# Supplementary material for: Nickel catalyzed C-N coupling of haloarenes with B2N4 reagents
Source: Nat Commun. 2025 Apr 3;16:3202. doi: 10.1038/s41467-025-58438-6 (PMC11968942; doi:10.1038/s41467-025-58438-6)
Supplement: Supplementary file 1 — Supplementary Information [file 41467_2025_58438_MOESM1_ESM.pdf]

# Supplementary Information

## Nickel catalyzed C-N coupling of haloarenes with $B_2N_4$ reagents

*Qianqian Chang*[1,‡], *Qini Li*[1,‡], *Yi-Hui Deng*[2,3,‡], *Tian-Yu Sun*[2,3,*], *Yun-Dong Wu*[2,3,4,*], *Leifeng Wang*[1,*]

[1]School of Pharmaceutical Sciences (Shenzhen), Shenzhen Campus of Sun Yat-Sen University, No.66, Gongchang Road, Shenzhen 518107, P. R. China.

[2]Key Laboratory of Computational Chemistry and Drug Design, State Key Laboratory of Chemical Oncogenomics, Shenzhen Key Laboratory of Chemical Genomics, School of Chemical Biology and Biotechnology, Peking University Shenzhen Graduate School, Shenzhen, Guangdong, PR China, 518055.

[3]Institute of Chemical Biology, Shenzhen Bay Laboratory, Shenzhen 518132, China.

[4]College of Chemistry and Molecular Engineering, Peking University, Beijing 100871, China.

[‡]These authors contributed equally.

*Corresponding E-mails: Tian-Yu_Sun@pku.edu.cn., wuyd@pkusz.edu.cn., wanglf33@mail.sysu.edu.cn

# Table of contents


1. General Information ........................................ 3

2. Optimization of the Reaction Conditions ........................................ 4

   2.1 Optimization of Reaction Conditions for Aryl Bromides ........................................ 4

3. Substrate Scope and Characterization Data ........................................ 5

4. Mechanistic Investigations ........................................ 42

   4.1 probing experiments and radical trapping experiments ........................................ 42

   4.2 Reaction of aryl bromide catalyzed by Ni(0) ........................................ 44

   4.3 Reaction of aryl bromide catalyzed by in-situ generated Ni(I) ........................................ 47

   4.4 Reaction of aryl bromide catalyzed by Ni(II) at room temperature ........................................ 48

   4.5 EPR study ........................................ 48

   4.6 Detecting the generation of Ni(III) intermediates in solution by HRMS ........................................ 50

   4.7 Detecting the key intermediates in solution by HRMS ........................................ 52

   4.8 Dimethylamine source confirmation ........................................ 54

   4.9 DFT calculation study ........................................ 55

5. NMR Spectroscopic Data ........................................ 59

6. References ........................................ 169

# 1. General Information

All reactions were performed in oven-dried 4 mL vials under the protection of argon in the glove box unless otherwise noted. Stirring was achieved by placing the assembled reactor on IKA C-MAG HS 7 control magnetic stir bars. (see the picture below for reaction setup).

Analytical TLC was performed on silica gel GF254 plates. The TLC plates were visualized by ultraviolet light (λ = 254 nm). Organic solutions were concentrated using a rotary evaporator with a diaphragm vacuum pump purchased from EYELA. Fresh silica gel chromatography was performed using 200-300 mesh silica gel (Qingdao, China).

Proton and carbon magnetic resonance spectra ($^{1}H$ NMR, $^{13}C$ NMR, $^{19}F$ NMR and $^{11}B$ NMR ) were recorded on a Bruker AVANCE III ($^{1}H$ NMR at 500 MHz, $^{13}C$ NMR at 126 MHz, $^{19}F$ NMR at 471 MHz, $^{11}B$ NMR at 161 MHz)

Spectrometer with solvent resonance as the internal standard ($^{1}H$ NMR: $CDCl_3$ at 7.26 ppm, DMSO-$d_6$ at 2.50 ppm; $^{13}C$ NMR: $CDCl_3$ at 77.16 ppm, DMSO-$d_6$ at 39.6 ppm). NMR yield using pyrazine as internal standard. $^{1}H$ NMR data are reported as follows: chemical shift, multiplicity (s = singlet, d = doublet, t = triplet, dd = doublet of doublets, ddd = doublet of doublets of doublets, ddddd = doublet of doublets of doublets of doublets of doublets, dt = doublet of triplets, ddt = doublet of doublets of triplets, td = triplet of doublets, tt = triplet of triplets, m = multiplet, q = quartet), coupling constants (Hz), and integration. High-resolution mass spectrometry (HRMS-ESI) was recorded on a Shimadzu LCMS-IT-TOF.

Commercially available reagents were purchased from Sigma-Aldrich, Adamas-beta, TCI, Bidepharm and were used as received unless otherwise noted. Super dry solvents such as acetonitrile (MeCN) dimethylformamide (DMF) were purchased from Adamas-beta. Other common solvents such as petroleum ether and ethyl acetate (EtOAc) are rectification grade for fresh silica gel chromatography purchased from General-reagent.

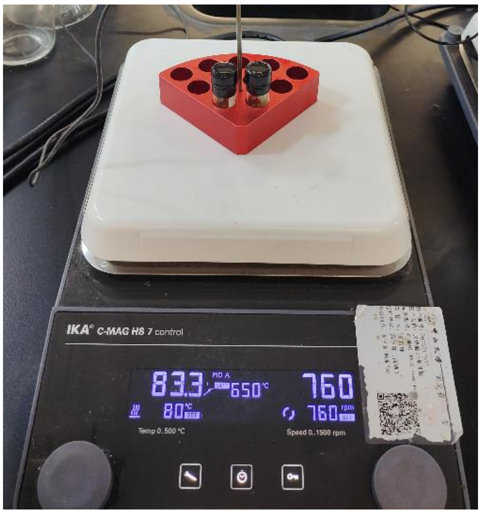


**Figure S1.** The reaction device.

## 2. Optimization of the Reaction Conditions

**General procedure (A):**

An oven-dried 4 mL vial was charged with a magnetic stir bar, substrate, Ni catalysts, $B_2(NMe_2)_4$, and solvent in the glove box. The vial was sealed with a plastic cap and then stirring was achieved by placing the assembled reactor on IKA C-MAG HS 7 control magnetic stir bars for 24 h. After reaction completion, the reaction was quenched with $H_2O$ and diluted with EtOAc. The resulting mixture was separated and extracted with EtOAc (three times). The combined organic layer was dried over anhydrous $Na_2SO_4$, filtered and concentrated *in vacuo*. The reaction mixture was purified by fresh silica gel chromatography to afford the desired product.

### 2.1 Optimization of Reaction Conditions for Aryl Bromides

**Table S1**. Optimization of reaction conditions for aryl bromides

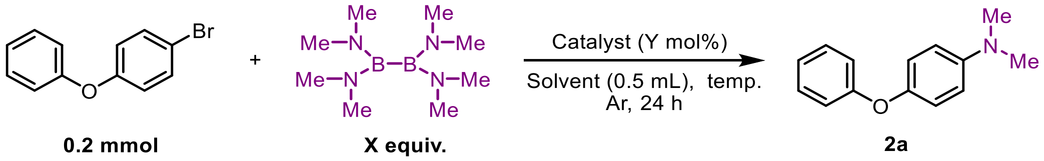


| Entry | $B_2(NMe_2)_4$ (X equiv.) | Catalyst (Y mol%) | Temperature | Solvent | Yield%[a] |
|---|---|---|---|---|---|
| 1 | 1.0 | $FeBr_3$ (10 mol%) | 60 °C | DMF | 0% |
| 2 | 1.0 | $Fe(OAc)_2$ (10 mol%) | 60 °C | DMF | 0% |
| 3 | 1.0 | $CuBr_2$ (10 mol%) | 60 °C | DMF | 0% |
| 4 | 1.0 | $Cu(OAc)_2$ (10 mol%) | 60 °C | DMF | 0% |
| 5 | 1.0 | $Pd(OAc)_2$ (10 mol%) | 60 °C | DMF | 0% |
| 6 | 1.0 | $NiCl_2$•$6H_2O$ (10 mol%) | 60 °C | DMF | 44% |
| 7 | 1.0 | $Ni(acac)_2$ (10 mol%) | 60 °C | DMF | 52% |
| 8 | 1.0 | $FeBr_3$ (10 mol%) | 80 °C | DMF | 0% |
| 9 | 1.0 | $Fe(OAc)_2$ (10 mol%) | 80 °C | DMF | 0% |
| 10 | 1.0 | $CuBr_2$ (10 mol%) | 80 °C | DMF | 0% |
| 11 | 1.0 | $Cu(OAc)_2$ (10 mol%) | 80 °C | DMF | Trace |
| 12 | 1.0 | $Pd(OAc)_2$ (10 mol%) | 80 °C | DMF | 0% |
| 13 | 1.0 | $NiCl_2$•$6H_2O$ (10 mol%) | 80 °C | DMF | 80% |
| 14 | 1.0 | $Ni(phen)Cl_2$ (10 mol%) | 80 °C | DMF | 15% |
| 15 | 1.0 | $NiBr_2(PPh_3)_2$ (10 mol%) | 80 °C | DMF | 55% |
| 16 | 1.0 | $Ni(dppp)Cl_2$ (10 mol%) | 80 °C | DMF | 74% |
| 17 | 1.0 | $Ni(COD)_2$ (10 mol%) | 80 °C | DMF | 0% |
| 18 | 1.0 | $Ni(OTf)_2$ (10 mol%) | 80 °C | DMF | 78% |
| 19 | 1.0 | $NiBr_2(DME)$ (10 mol%) | 80 °C | DMF | 45% |

| | | | | | |
|---|---|---|---|---|---|
| **20** | **1.0** | **$Ni(acac)_2$ (10 mol%)** | **80 °C** | **DMF** | **98%(94%)[b]** |
| 21 | 1.0 | $Ni(acac)_2$ (5 mol%) | 80 °C | DMF | 72% |
| 22 | 0.9 | $Ni(acac)_2$ (10 mol%) | 80 °C | DMF | 92% |
| 23 | 0.8 | $Ni(acac)_2$ (10 mol%) | 80 °C | DMF | 88% |
| 24 | 0.7 | $Ni(acac)_2$ (10 mol%) | 80 °C | DMF | 85% |
| 25 | 0.5 | $Ni(acac)_2$ (10 mol%) | 80 °C | DMF | 42% |
| 26 | 0.25 | $Ni(acac)_2$ (10 mol%) | 80 °C | DMF | 0% |
| 27 | 0 | $Ni(acac)_2$ (10 mol%) | 80 °C | DMF | 0% |
| 28 | 1.0 | none | 80 °C | DMF | 0% |
| 29 | 1.0 | $Ni(acac)_2$ (10 mol%) | 80 °C | DMAc | 89% |
| 30 | 1.0 | $Ni(acac)_2$ (10 mol%) | 80 °C | THF | 17% |
| 31 | 1.0 | $Ni(acac)_2$ (10 mol%) | 80 °C | MeCN | 32% |
| 32 | 1.0 | $Ni(acac)_2$ (10 mol%) | 80 °C | DMSO | Trace |

[a] Yield was determined by the $^1$H NMR analysis using pyrazine as an internal standard. [b] Isolated yield.

In summary, the optimized reaction condition for aryl bromides: substrates (0.2 mmol, 1.0 equiv.), $Ni(acac)_2$ (0.02 mmol, 10 mol%), $B_2(NMe_2)_4$ (0.2 mmol, 1.0 equiv.), DMF (0.5 mL), stirring at 80 °C on IKA C-MAG HS 7 control magnetic stir bars for 24 h.

# 3. Substrate Scope and Characterization Data

**General procedure (B):**

An oven-dried 4 mL vial was charged with a magnetic stir bar, aryl bromides or alkyl bromides (0.2 mmol, 1.0 equiv.), $Ni(acac)_2$ (0.02 mmol, 10 mol%), $B_2(NMe_2)_4$ (0.2 mmol, 1.0 equiv.), DMF (0.5 mL) in the glove box. The vial was sealed with a plastic cap and then stirring was achieved by placing the assembled reactor at 80 °C on IKA C-MAG HS 7 control magnetic stir bars for 24 h. After reaction completion, the reaction was quenched with $H_2O$ and diluted with EtOAc. The resulting mixture was separated and extracted with EtOAc (three times). The combined organic layer was dried over anhydrous $Na_2SO_4$, filtered and concentrated *in vacuo*. The reaction mixture was purified by fresh silica gel chromatography to afford the desired product.

***N,N*-Dimethyl-4-phenoxyaniline (2a)**

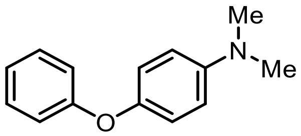


Following general procedure **(B)**, **2a** was obtained as pale yellow liquid in 94% isolated yield from 4-bromophenoxybenzene using 180:1 petroleum ether/EtOAc as eluent.

**$^1$H NMR** (500 MHz, $CDCl_3$) δ 7.30 (t, $J$ = 8.0 Hz, 2H), 7.03 (t, $J$ = 7.4 Hz, 1H), 7.00 (d, $J$ = 9.0 Hz, 2H), 6.97 (d, $J$ = 7.8 Hz, 2H), 6.77 (d, $J$ = 9.0 Hz, 2H), 2.96 (s, 6H).

$^{13}$**C NMR** (126 MHz, $CDCl_3$) δ 159.21, 147.76, 147.48, 129.62, 122.06, 121.04, 117.28, 114.12, 41.35. The NMR data were in consistent with the reported data.[1]

**_N,N_-dimethylaniline (2b)**

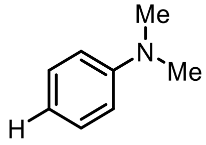

Following general procedure **(B)**, **2b** was obtained as colorless liquid in 84% isolated yield from bromobenzene using 80:1 petroleum ether/EtOAc as eluent.

$^{1}$**H NMR** (500 MHz, $CDCl_3$) δ 7.22 (dd, *J* = 9.0, 7.1 Hz, 2H), 6.71 (dd, *J* = 10.5, 7.5 Hz, 3H), 2.90 (s, 6H).The NMR data were in consistent with the reported data.[2]

**4-_tert_-Butyl-_N,N_-dimethylaniline (2c)**

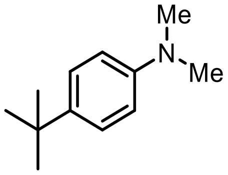

Following general procedure **(B)**, **2c** was obtained as pale yellow oil in 64% isolated yield from 1-bromo-4-*tert*-butylbenzene using 80:1 petroleum ether/EtOAc as eluent.

$^{1}$**H NMR** (500 MHz, $CDCl_3$) δ 7.37 (d, *J* = 8.8 Hz, 2H), 6.81 (d, *J* = 8.8 Hz, 2H), 3.00 (s, 6H), 1.39 (s, 9H).

$^{13}$**C NMR** (126 MHz, $CDCl_3$) δ 148.69, 139.51, 125.95, 112.77, 40.95, 33.86, 31.66. The NMR data were in consistent with the reported data.[1]

**_N,N_-dimethyl-4-(tetramethyl-l5-silyl)aniline (2d)**

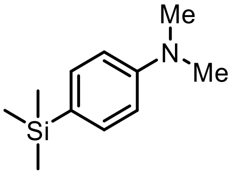

Following general procedure **(B)**, **2d** was obtained as colourless liquid in 85% isolated yield from (4-bromophenyl)trimethylsilane using 40:1 petroleum ether/EtOAc as eluent.

$^{1}$**H NMR** (500 MHz, $CDCl_3$) δ 7.32 (d, *J* = 8.4 Hz, 2H), 6.66 (d, *J* = 8.2 Hz, 2H), 2.87 (s, 6H), 0.15 (s, 9H).

$^{13}$**C NMR** (126 MHz, $CDCl_3$) δ 150.85, 134.25, 125.50, 111.89, 40.15.-0.96. The NMR data were in consistent with the reported data.[3]

**_N,N_-dimethyl-4-(methylthio)aniline (2e)**

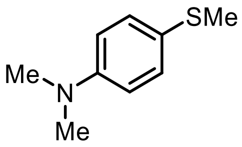

Following general procedure **(B)**, **2e** was obtained as colorless transparent liquid in 79% isolated

yield from (4-bromophenyl)(methyl)sulfane using 40:1 petroleum ether/EtOAc as eluent.

**$^1$H NMR** (500 MHz, $CDCl_3$) δ 7.29 (d, $J$ = 8.8 Hz, 2H), 6.69 (d, $J$ = 8.8 Hz, 2H), 2.94 (s, 6H), 2.42 (s, 3H).

**$^{13}$C NMR** (126 MHz, $CDCl_3$) δ 148.57, 130.47, 122.51, 112.28, 39.69, 18.30. The NMR data were in consistent with the reported data.[4]

**$N^1$,$N^1$,$N^4$,$N^4$-tetramethylbenzene-1,4-diamine (2f)**

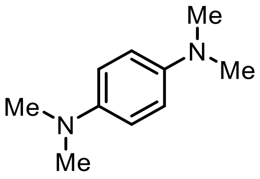


Following general procedure **(B)**, **2f** was obtained as gray solid in 71% isolated yield from 4-bromo-*N,N*-dimethylaniline using 5:1 petroleum ether/EtOAc as eluent.

**$^1$H NMR** (500 MHz, $CDCl_3$) δ 6.81 (s, 4H), 2.86 (s, 12H).

**$^{13}$C NMR** (126 MHz, $CDCl_3$) δ 144.14, 115.50, 42.17. The NMR data were in consistent with the reported data.[5]

***N,N*-Dimethyl-4-morpholinoaniline (2g)**

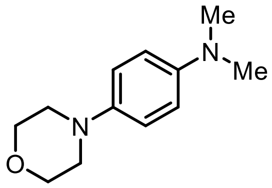


Following general procedure **(B)**, **2g** was obtained as white solid in 85% isolated yield from 4-bromophenyl-morpholine using 4:1 petroleum ether/EtOAc as eluent.

**$^1$H NMR** (500 MHz, $CDCl_3$) δ 6.91 (d, $J$ = 8.9 Hz, 2H), 6.78 (d, $J$ = 8.9 Hz, 2H), 3.91 – 3.83 (m, 4H), 3.05 (s, 4H), 2.89 (s, 6H).

**$^{13}$C NMR** (126 MHz, $CDCl_3$) δ 145.89, 143.26, 118.03, 114.51, 67.17, 51.12, 41.54. The NMR data were in consistent with the reported data.[6]

***N,N*-Dimethyl-4-(4,4,5,5-tetramethyl-1,3,2-dioxaborolan-2-yl)aniline (2h)**

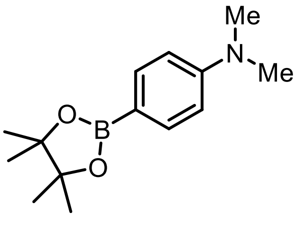


Following general procedure **(B)**, **2h** was obtained as white solid in 85% isolated yield from 4-bromophenyl pinacol borane using 60:1 petroleum ether/EtOAc as eluent.

**$^1$H NMR** (500 MHz, $CDCl_3$) δ 7.71 (d, $J$ = 8.7 Hz, 2H), 6.70 (d, $J$ = 8.7 Hz, 2H), 2.99 (s, 6H), 1.34 (s, 12H).

**$^{13}$C NMR** (126 MHz, $CDCl_3$) δ 152.66, 136.26, 111.36, 83.26, 40.22, 24.96.

**$^{11}$B NMR** (161 MHz, $CDCl_3$) δ 30.58. The NMR data were in consistent with the reported data.[7]

**$N,N$-dimethyl-4-nitroaniline (2i)**

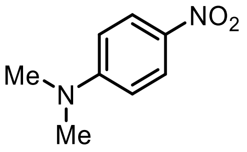


Following general procedure (B)**,** **2i** was obtained as light yellow solid in 85% isolated yield from 1-bromo-4-nitrobenzene using 10:1 petroleum ether/EtOAc as eluent.

**$^1$H NMR** (500 MHz, $CDCl_3$) δ 8.10 (d, $J$ = 9.4 Hz, 2H), 6.58 (d, $J$ = 9.4 Hz, 2H), 3.10 (s, 6H).

**$^{13}$C NMR** (126 MHz, $CDCl_3$) δ 153.21, 135.89, 125.07, 109.19, 39.22. The NMR data were in consistent with the reported data.[8]

**4-Cyano-$N,N$-dimethylaniline (2j)**

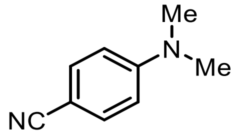


Following general procedure **(B)**, **2j** was obtained as white solid in 77% isolated yield from 4-bromobenzonitrile using 25:1 petroleum ether/EtOAc as eluent.

**$^1$H NMR** (400 MHz, $CDCl_3$) δ 7.44 (d, $J$ = 9.0 Hz, 2H), 6.62 (d, $J$ = 9.0 Hz, 2H), 3.02 (s, 6H).

**$^{13}$C NMR** (101 MHz, $CDCl_3$) δ 152.50, 133.47, 120.82, 111.55, 97.48, 40.07. The NMR data were in consistent with the reported data.[5]

**$N,N$-Dimethyl-4-(methylsulfonyl)aniline (2k)**

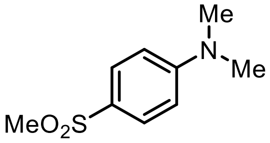


Following general procedure **(B)**, **2k** was obtained as beige solid in 87% isolated yield from 4-bromoohenyl methyl sulfone using 1:1 petroleum ether/EtOAc as eluent.

**$^1$H NMR** (500 MHz, $CDCl_3$) δ 7.70 (d, $J$ = 9.1 Hz, 2H), 6.67 (d, $J$ = 9.1 Hz, 2H), 3.03 (s, 6H), 2.97 (s, 3H).

**$^{13}$C NMR** (126 MHz, $CDCl_3$) δ 153.44, 129.04, 125.85, 111.01, 45.14, 40.12. The NMR data were in consistent with the reported data.[9]

**Ethyl 4-(dimethylamino)benzoate (2l)**

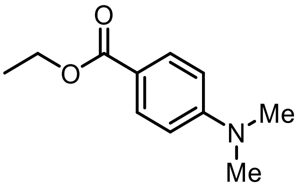


Following general procedure **(B)**, **2l** was obtained as white solid in 82% isolated yield from ethyl 4-bromobenzoate using 10:1 petroleum ether/EtOAc as eluent.

**$^1$H NMR** (500 MHz, $CDCl_3$) δ 7.91 (d, $J$ = 8.7 Hz, 2H), 6.63 (d, $J$ = 8.9 Hz, 2H), 4.32 (q, $J$ = 7.1

Hz, 2H), 3.02 (s, 6H), 1.36 (t, $J$ = 7.2 Hz, 3H).

**$^{13}$C NMR** (126 MHz, $CDCl_3$) δ 167.12, 153.33, 131.27, 117.42, 110.75, 60.18, 40.12, 14.57.

The NMR data were in consistent with the reported data.[10]

**_N'_-Acetyl-_N,N_-dimethyl-1,4-phenylenediamine (2m)**

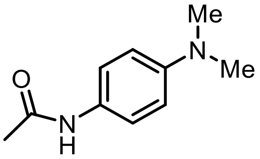


Following general procedure **(B)**, **2m** was obtained as beige solid in 77% isolated yield from 4-bromoacetanilide using 1:1 petroleum ether/EtOAc as eluent.

**$^1$H NMR** (500 MHz, DMSO-$d_6$) δ 9.59 (s, 1H), 7.46 – 7.17 (m, 2H), 6.75 – 6.54 (m, 2H), 2.82 (s, 6H), 1.97 (s, 3H).

**$^{13}$C NMR** (126 MHz, DMSO-$d_6$) δ 167.43, 146.96, 129.40, 120.55, 112.78, 40.63, 23.81.

The NMR data were in consistent with the reported data.[11]

**4-(Dimethylamino)benzamide (2n)**

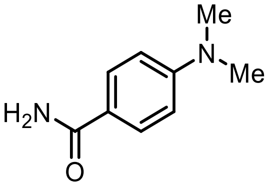


Following general procedure **(B)**, **2n** was obtained as off-white solid in 40% isolated yield from 4-bromobenzamide using 1:1 petroleum ether/EtOAc as eluent.

**$^1$H NMR** (500 MHz, DMSO-*d6*) δ 7.74 (d, $J$ = 8.8 Hz, 2H), 7.65 (s, 1H), 6.94 (s, 1H), 6.67 (d, $J$ = 8.8 Hz, 2H), 2.95 (s, 6H).

**$^{13}$C NMR** (126 MHz, DMSO-*d6*) δ 168.01, 152.15, 128.95, 120.95, 110.72, 39.73. The NMR data were in consistent with the reported data.[12]

**_N,N_-Dimethyl-4-biphenylamine (2o)**

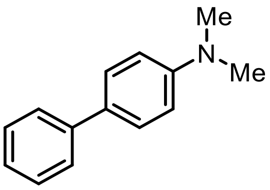


Following general procedure **(B)**, **2o** was obtained as white solid in 99% isolated yield from 4-bromobiphenyl using 60:1 petroleum ether/EtOAc as eluent.

**$^1$H NMR** (400 MHz, $CDCl_3$) δ 7.59 (dd, $J$ = 8.3, 1.1 Hz, 2H), 7.54 (d, $J$ = 8.9 Hz, 2H), 7.43 (t, $J$ = 7.7 Hz, 2H), 7.29 (t, $J$ = 7.4 Hz, 1H), 6.84 (d, $J$ = 8.9 Hz, 2H), 3.02 (s, 6H).

**$^{13}$C NMR** (151 MHz, $CDCl_3$) δ 150.09, 141.34, 129.37, 128.77, 127.83, 126.42, 126.11, 112.90, 40.72. The NMR data were in consistent with the reported data.[9]

**4'-Fluoro-_N,N_-dimethyl-[1,1'-biphenyl]-4-amine (2p)**

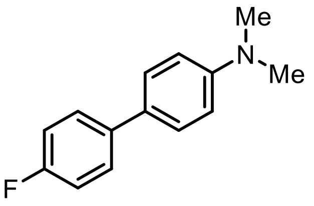

Following general procedure **(B)**, **2p** was obtained as white solid in 95% isolated yield from 4-bromo-4'-fluorobiphenyl using 60:1 petroleum ether/EtOAc as eluent.

**$^{1}$H NMR** (500 MHz, $CDCl_3$) δ 7.51 (dd, *J* = 8.6, 5.4 Hz, 2H), 7.47 (d, *J* = 8.8 Hz, 2H), 7.10 (t, *J* = 8.7 Hz, 2H), 6.82 (d, *J* = 8.7 Hz, 2H), 3.01 (s, 6H).

**$^{13}$C NMR** (126 MHz, $CDCl_3$) δ 161.87 (d, *J* = 244.5 Hz), 150.06, 137.51 (d, *J* = 3.2 Hz), 128.46, 127.82 (d, *J* = 7.8 Hz), 127.70, 115.54 (d, *J* = 21.3 Hz), 112.93, 40.69.

**$^{19}$F NMR** (471 MHz, $CDCl_3$) δ -117.71. The NMR data were in consistent with the reported data.[13]

**_N,N_-Dimethyl-4-(1_H_-pyrrol-1-yl)aniline (2q)**

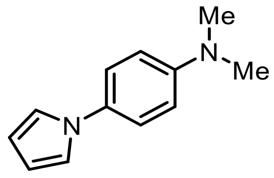

Following general procedure **(B)**, **2q** was obtained as white solid in 88% isolated yield from 1-(4-bromophenyl)-1*H*-pyrrole using 80:1 petroleum ether/EtOAc as eluent.

**$^{1}$H NMR** (500 MHz, $CDCl_3$) δ 7.30 – 7.26 (m, 2H), 6.99 (t, *J* = 2.2 Hz, 2H), 6.79 – 6.75 (m, 2H), 6.31 (t, *J* = 2.1 Hz, 2H), 2.97 (s, 6H).

**$^{13}$C NMR** (126 MHz, $CDCl_3$) δ 149.10, 131.43, 122.35, 119.90, 113.26, 109.46, 40.98. The NMR data were in consistent with the reported data.[14]

**_N,N_-Dimethyl-4-(naphthalen-2-yl)aniline (2r)**

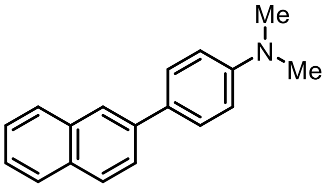

Following general procedure **(B)**, **2r** was obtained as white solid in 92% isolated yield from 2-(4-bromophenyl)naphthalene using 60:1 petroleum ether/EtOAc as eluent.

**$^{1}$H NMR** (400 MHz, $CDCl_3$) δ 8.01 (s, 1H), 7.87 (dd, J = 14.4, 8.2 Hz, 3H), 7.76 (dd, J = 8.5, 1.7 Hz, 1H), 7.67 (d, J = 8.8 Hz, 2H), 7.52 – 7.42 (m, 2H), 6.87 (d, J = 8.8 Hz, 2H), 3.03 (s, 6H).

**$^{13}$C NMR** (151 MHz, $CDCl_3$) δ 150.13, 138.66, 134.02, 132.18, 128.33, 128.11, 128.08, 127.73, 126.21, 125.45, 125.39, 124.32, 113.02, 40.75. The NMR data were in consistent with the reported data.[9]

**$N^1$,$N^1$,3,5-tetramethylbenzene-1,4-diamine (2s)**

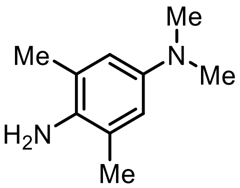


Following general procedure **(B)**, **2s** was obtained as yellow oil in 42% isolated yield from 4-bromo-2,6-dimethylaniline using 40:1 petroleum ether/EtOAc as eluent.

**$^1$H NMR** (500 MHz, $CDCl_3$) δ 6.52 (s, 2H), 3.26 (s, 2H), 2.82 (s, 6H), 2.19 (s, 6H).

**$^{13}$C NMR** (126 MHz, $CDCl_3$) δ 143.25, 133.77, 122.04, 114.15, 41.30, 17.14.The NMR data were in consistent with the reported data.[15]

**_N,N_-Dimethyl-4-hydroxymethylaniline (2t)**

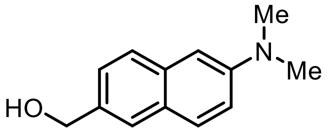


Following general procedure **(B)**, **2t** was obtained as white solid in 52% isolated yield from 4-bromobenzenemethanol using 4:1 petroleum ether/EtOAc as eluent.

**$^1$H NMR** (500 MHz, $CDCl_3$) δ 7.23 (d, $J$ = 8.6 Hz, 2H), 6.74 (d, $J$ = 8.7 Hz, 2H), 4.53 (s, 2H), 2.95 (s, 6H), 2.57 (s, 1H).

**$^{13}$C NMR** (126 MHz, $CDCl_3$) δ 150.28, 129.16, 128.57, 112.75, 65.01, 40.72. The NMR data were in consistent with the reported data.[16]

**4-(_N,N_-Dimethylamino)stilbene (2u)**

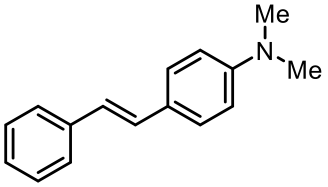


Following general procedure **(B)**, **2u** was obtained as white solid in 93% isolated yield from 1-bromo-4-styrylbenzene using 60:1 petroleum ether/EtOAc as eluent.

**$^1$H NMR** (500 MHz, $CDCl_3$) δ 7.51 (d, $J$ = 7.6 Hz, 2H), 7.45 (d, $J$ = 8.7 Hz, 2H), 7.36 (t, $J$ = 7.7 Hz, 2H), 7.24 (t, $J$ = 7.3 Hz, 1H), 7.09 (d, $J$ = 16.3 Hz, 1H), 6.96 (d, $J$ = 16.3 Hz, 1H), 6.75 (d, $J$ = 8.7 Hz, 2H), 3.01 (s, 6H).

**$^{13}$C NMR** (126 MHz, $CDCl_3$) δ 150.24, 138.31, 128.93, 128.70, 127.70, 126.80, 126.14, 125.93, 124.53, 112.60, 40.59. The NMR data were in consistent with the reported data.[17]

**_N,N_-dimethyl-4-(phenylethynyl)aniline (2v)**

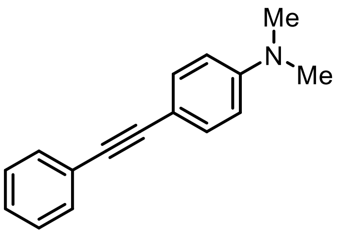

Following general procedure **(B)**, **2v** was obtained as white solid in 45% isolated yield from 1-bromo-4-(phenylethynyl)benzene using 80:1 petroleum ether/EtOAc as eluent.

**$^1$H NMR** (500 MHz, $CDCl_3$) δ 7.50 (d, *J* = 7.4 Hz, 2H), 7.41 (d, *J* = 8.4 Hz, 2H), 7.34 – 7.26 (m, 3H), 6.66 (d, *J* = 8.5 Hz, 2H), 2.99 (s, 6H).

**$^{13}$C NMR** (126 MHz, $CDCl_3$) δ 150.27, 132.87, 131.44, 128.38, 127.59, 124.31, 112.00, 110.23, 90.74, 87.48, 40.38.The NMR data were in consistent with the reported data.[18]

**Cyclopropyl(4-(dimethylamino)phenyl)methanone (2w)**

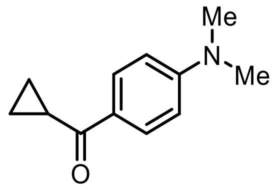


Following general procedure **(B)**, **2w** was obtained as beige solid in 90% isolated yield from 4-bromophenyl cyclopropyl ketone using 25:1 petroleum ether/EtOAc as eluent.

**$^1$H NMR** (500 MHz, $CDCl_3$) δ 7.96 (d, *J* = 9.0 Hz, 2H), 6.67 (d, *J* = 9.0 Hz, 2H), 3.05 (s, 6H), 2.64 – 2.59 (m, 1H), 1.19 – 1.14 (m, 2H), 0.96 – 0.91 (m, 2H).

**$^{13}$C NMR** (126 MHz, $CDCl_3$) δ 198.39, 153.37, 130.23, 126.05, 110.75, 40.12, 16.14, 10.68. The NMR data were in consistent with the reported data.[19]

**(4-(Dimethylamino)phenyl)(phenyl)methanone (2x)**

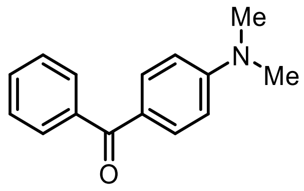


Following general procedure **(B)**, **2x** was obtained as yellow solid in 75% isolated yield from 4-bromobenzophenone using 25:1 petroleum ether/EtOAc as eluent.

**$^1$H NMR** (500 MHz, $CDCl_3$) δ 7.80 (d, J = 8.9 Hz, 2H), 7.72 (d, *J* = 7.3 Hz, 2H), 7.52 (t, *J* = 7.3 Hz, 1H), 7.45 (t, *J* = 7.5 Hz, 2H), 6.67 (d, *J* = 8.9 Hz, 2H), 3.06 (s, 6H).

**$^{13}$C NMR** (126 MHz, $CDCl_3$) δ 195.22, 153.37, 139.39, 132.81, 131.19, 129.51, 128.09, 124.80, 110.62, 40.12. The NMR data were in consistent with the reported data.[9]

***N,N*-dimethyl-2-nitroaniline (2y)**

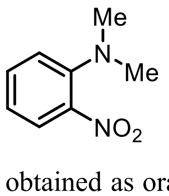


Following general procedure **(B)**, **2y** was obtained as orange liquid in 93% isolated yield from 1-bromo-2-nitrobenzene using 40:1 petroleum ether/EtOAc as eluent.

**$^1$H NMR** (500 MHz, $CDCl_3$) δ 7.76 (d, *J* = 8.2 Hz, 1H), 7.39 (t, *J* = 7.9 Hz, 1H), 7.02 (d, *J* = 8.5 Hz, 1H), 6.81 (t, *J* = 7.6 Hz, 1H), 2.88 (s, 6H).

**$^{13}$C NMR** (126 MHz, $CDCl_3$) δ 146.36, 139.49, 133.34, 126.83, 118.21, 118.01, 42.55. The NMR

data were in consistent with the reported data.[20]

**2-*N,N*-Dimethylaminobenzonitrile (2z)**

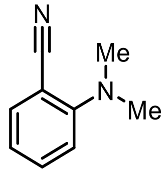


Following general procedure **(B)**, **2z** was obtained as pale yellow oil in 70% isolated yield from 2-bromobenzonitrile using 25:1 petroleum ether/EtOAc as eluent.

**$^1$H NMR** (500 MHz, $CDCl_3$) δ 7.47 (dd, *J* = 7.8, 1.8 Hz, 1H), 7.39 (ddd, *J* = 8.8, 7.3, 1.8 Hz, 1H), 6.87 (d, *J* = 8.5 Hz, 1H), 6.82 (t, *J* = 7.5 Hz, 1H), 3.02 (s, 6H).

**$^{13}$C NMR** (126 MHz, $CDCl_3$) δ 155.30, 134.93, 133.48, 119.71, 119.13, 116.76, 101.19, 43.00. The NMR data were in consistent with the reported data.[21]

***N,N*-Dimethyl-2-phenoxyaniline (2aa)**

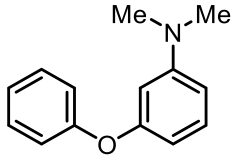


Following general procedure **(B)**, **2aa** was obtained as colorless liquid in 37% isolated yield from 1-bromo-2-phenoxybenzene using 180:1 petroleum ether/EtOAc as eluent.

**$^1$H NMR** (500 MHz, $CDCl_3$) δ 7.35 (t, *J* = 7.9 Hz, 2H), 7.17 – 6.99 (m, 5H), 6.95 (d, *J* = 4.1 Hz, 2H), 2.88 (s, 6H).

**$^{13}$C NMR** (126 MHz, $CDCl_3$) δ 157.58, 148.41, 144.92, 129.59, 124.43, 122.54, 121.69, 120.80, 118.56, 117.70, 43.07.

**IR (neat)**: ν = 3436.17, 3079.87, 2971.73, 2866.31, 2837.76, 2801.58, 2786.43, 1596.73, 1587.39, 1571.28, 1491.00, 1473.81, 1449.32, 1432.55, 1329.90, 1287.97, 1229.00, 1163.17, 1097.73, 949.00, 866.08, 751.50, 688.71 $cm^{-1}$.

**HRMS (ESI-TOF) m/z**: $[M+H]^+$ Calcd. for $C_{14}H_{15}NO$ 214.1226; Found 214.1236.

**3-*N,N*-Dimethylaminobenzonitrile (2ab)**

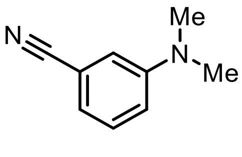


Following general procedure **(B)**, **2ab** was obtained as colorless liquid in 82% isolated yield from 3-bromobenzonitrile using 25:1 petroleum ether/EtOAc as eluent.

**$^1$H NMR** (500 MHz, $CDCl_3$) δ 7.30 – 7.24 (m, 1H), 6.94 (d, *J* = 7.5 Hz, 1H), 6.88 (d, *J* = 8.0 Hz, 2H), 2.97 (s, 6H).

**$^{13}$C NMR** (126 MHz, $CDCl_3$) δ 150.24, 129.75, 119.83, 119.35, 116.25, 114.74, 112.73, 40.14. The NMR data were in consistent with the reported data.[22]

***N,N*-Dimethyl-3-phenoxyaniline (2ac)**

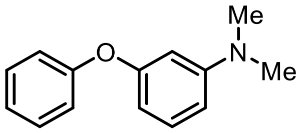

Following general procedure **(B)**, **2ac** was obtained as colorless liquid in 71% isolated yield from 1-bromo-3-phenoxybenzene using 180:1 petroleum ether/EtOAc as eluent.

**$^{1}$H NMR** (500 MHz, $CDCl_3$) δ 7.32 – 7.22 (m, 2H), 7.14 (t, *J* = 8.2 Hz, 1H), 7.02 (dd, *J* = 15.3, 7.6 Hz, 3H), 6.45 (dd, *J* = 8.3, 1.9 Hz, 1H), 6.41 (t, *J* = 2.2 Hz, 1H), 6.35 – 6.28 (m, 1H), 2.87 (s, 6H).

**$^{13}$C NMR** (126 MHz, $CDCl_3$) δ 158.10, 157.69, 152.17, 130.02, 129.63, 122.82, 118.64, 107.79, 106.97, 103.59, 40.49. The NMR data were in consistent with the reported data.[23]

**_N,N_-Dimethylbenzo[_d_][1,3]dioxol-5-amine (2ad)**

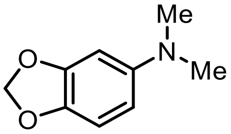

Following general procedure **(B)**, **2ad** was obtained as pale yellow liquid in 85% isolated yield from 1,2-(methylenedioxy)-4-bromobenzene using 45:1 petroleum ether/EtOAc as eluent.

**$^{1}$H NMR** (400 MHz, $CDCl_3$) δ 6.74 (d, *J* = 8.5 Hz, 1H), 6.45 (d, *J* = 2.5 Hz, 1H), 6.19 (dd, *J* = 8.5, 2.5 Hz, 1H), 5.88 (s, 2H), 2.88 (s, 6H).

**$^{13}$C NMR** (151 MHz, $CDCl_3$) δ 148.39, 147.31, 139.48, 108.38, 105.27, 100.67, 96.56, 41.90. The NMR data were in consistent with the reported data.[1]

**Ethyl 3-(dimethylamino)-4-fluorobenzoate (2ae)**

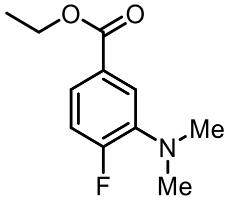

Following general procedure **(B)**, **2ae** was obtained as colorless transparent liquid in 78% isolated yield from ethyl 3-bromo-4-fluorobenzoate using 10:1 petroleum ether/EtOAc as eluent.

**$^{1}$H NMR** (500 MHz, $CDCl_3$) δ 7.72 (dd, *J* = 8.5, 2.0 Hz, 1H), 7.64 (dd, *J* = 14.5, 2.0 Hz, 1H), 6.78 (t, *J* = 8.8 Hz, 1H), 4.33 (q, *J* = 7.1 Hz, 2H), 2.97 (d, *J* = 1.6 Hz, 6H), 1.37 (t, *J* = 7.1 Hz, 3H).

**$^{13}$C NMR** (126 MHz, $CDCl_3$) δ 164.88 (d, *J* = 2.5 Hz), 152.82, 143.12 (d, *J* = 7.9 Hz), 125.39 (d, *J* = 2.7 Hz), 120.17 (d, *J* = 7.1 Hz), 116.39 (d, *J* = 23.1 Hz), 115.12 (d, *J* = 4.0 Hz), 59.64, 41.18 (d, *J* = 5.5 Hz), 13.36.

**$^{19}$F NMR** (471 MHz, $CDCl_3$) δ -123.03.

**IR (ATR):** ν = 2939.26, 2825.45, 2784.54, 1710.25, 1612.75, 1594.12, 1565.83, 1522.13, 1442.88, 1388.29, 1365.38, 1292.78, 1256.51, 1234.92, 1157.83, 1118.72, 1041.62, 1024.91, 984.86, 956.87, 929.24, 909.53, 816.69, 764.34, 717.24, 686.48, 631.60, 576.73, 475.39, 446.44, 424.59 $cm^{-1}$.

**HRMS (ESI-TOF) m/z**: $[M+Na]^+$ Calcd. for $C_{11}H_{14}FNO_2$ 234.0901; Found 234.0902.

**4-Fluoro-_N,N_-dimethyl-2-nitroaniline (2af)**

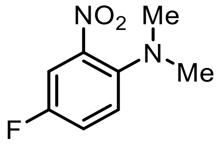


Following general procedure **(B)**, **2af** was obtained as orange solid in 49% isolated yield from 2-bromo-5-fluoronitrobenzene using 100:1 petroleum ether/EtOAc as eluent.

**$^{1}$H NMR** (500 MHz, $CDCl_3$) δ 7.51 (dd, *J* = 8.2, 3.0 Hz, 1H), 7.18 (ddd, *J* = 10.2, 7.3, 3.0 Hz, 1H), 7.03 (dd, *J* = 9.2, 4.6 Hz, 1H), 2.84 (s, 6H).

**$^{13}$C NMR** (126 MHz, $CDCl_3$) δ 154.77 (d, *J* = 241.9 Hz), 143.50 (d, *J* = 2.3 Hz), 139.48 (d, *J* = 6.4 Hz), 120.96 (d, *J* = 22.5 Hz), 120.20 (d, *J* = 7.5 Hz), 113.07 (d, *J* = 26.7 Hz), 43.10.

**$^{19}$F NMR** (471 MHz, $CDCl_3$) δ -123.94. The NMR data were in consistent with the reported data.[24]

**_N,N_-Dimethyl-2-nitro-4-(trifluoromethyl)aniline (2ag)**

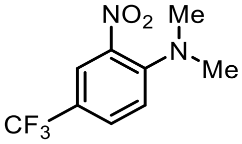


Following general procedure **(B)**, **2ag** was obtained as yellow solid in 87% isolated yield from 1-bromo-4-trifluoromethyl-2-nitrobenzene using 100:1 petroleum ether/EtOAc as eluent.

**$^{1}$H NMR** (500 MHz, $CDCl_3$) δ 8.02 (d, *J* = 2.3 Hz, 1H), 7.55 (dd, *J* = 9.0, 2.3 Hz, 1H), 7.05 (d, *J* = 9.0 Hz, 1H), 2.95 (s, 6H).

**$^{13}$C NMR** (126 MHz, $CDCl_3$) δ 147.85, 137.01, 129.57 (q, *J* = 3.2 Hz), 124.84 (q, *J* = 4.1 Hz), 123.77 (q, *J* = 270.6 Hz), 118.56 (q, *J* = 34.3 Hz), 117.82, 42.16.

**$^{19}$F NMR** (471 MHz, $CDCl_3$) δ -61.67. The NMR data were in consistent with the reported data.[25]

**4-Chloro-_N,N_-dimethyl-2-nitroaniline (2ah)**

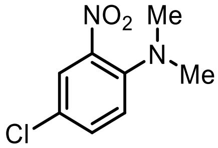


Following general procedure **(B)**, **2ah** was obtained as orange solid in 79% isolated yield from 2-chloro-5-bromonitrobenzene using 100:1 petroleum ether/EtOAc as eluent.

**$^{1}$H NMR** (500 MHz, $CDCl_3$) δ 7.72 (t, *J* = 2.4 Hz, 1H), 7.31 (dt, *J* = 9.0, 2.2 Hz, 1H), 6.94 (d, *J* = 9.1 Hz, 1H), 2.86 (s, 6H).

**$^{13}$C NMR** (126 MHz, $CDCl_3$) δ 144.94, 138.79, 133.24, 126.19, 122.31, 119.38, 42.45. The NMR data were in consistent with the reported data.[26]

**2-Chloro-_N,N_-dimethyl-4-nitroaniline (2ai)**

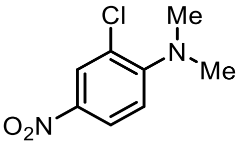

Following general procedure **(B)**, **2ai** was obtained as yellow solid in 80% isolated yield from 1-bromo-2-chloro-4-nitrobenzene using 100:1 petroleum ether/EtOAc as eluent.

**$^{1}$H NMR** (500 MHz, $CDCl_3$) δ 8.20 (d, *J* = 2.4 Hz, 1H), 8.04 (dd, *J* = 9.1, 2.4 Hz, 1H), 6.96 (d, *J* = 9.1 Hz, 1H), 3.00 (s, 6H).

**$^{13}$C NMR** (126 MHz, $CDCl_3$) δ 155.56, 140.87, 127.26, 124.99, 123.43, 117.95, 43.09. The NMR data were in consistent with the reported data.[27]

**5-Chloro-*N,N*-dimethyl-m-toluidine (2aj)**

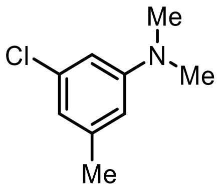


Following general procedure **(B)**, **2aj** was obtained as colorless oil in 65% isolated yield from 1-bromo-3-chloro-5-methylbenzene using 140:1 petroleum ether/EtOAc as eluent.

**$^{1}$H NMR** (500 MHz, $CDCl_3$) δ 6.55 (d, *J* = 10.8 Hz, 2H), 6.43 (s, 1H), 2.95 (s, 6H), 2.32 (s, 3H).

**$^{13}$C NMR** (126 MHz, $CDCl_3$) δ 151.51, 140.15, 134.77, 117.24, 111.40, 109.63, 40.50, 21.79. The NMR data were in consistent with the reported data.[28]

**Dimethyl 5-(*N,N*-dimethylamino)isophthalate (2ak)**

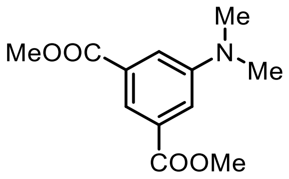


Following general procedure **(B)**, **2ak** was obtained as white solid in 94% isolated yield from dimethyl 5-bromoisophthalate using 15:1 petroleum ether/EtOAc as eluent.

**$^{1}$H NMR** (500 MHz, $CDCl_3$) δ 7.98 (s, 1H), 7.53 (d, *J* = 1.2 Hz, 2H), 3.91 (s, 6H), 3.02 (s, 6H).

**$^{13}$C NMR** (126 MHz, $CDCl_3$) δ 167.15, 150.47, 131.20, 118.33, 117.08, 52.33, 40.58. The NMR data were in consistent with the reported data.[26]

**3,5-Dimethyl-*N,N*-dimethylaniline (2al)**

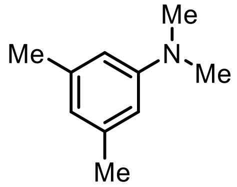


Following general procedure **(B)**, **2al** was obtained as yellow liquid in 94% isolated yield from 5-bromo-1,3-xylene using 80:1 petroleum ether/EtOAc as eluent.

**$^{1}$H NMR** (500 MHz, $CDCl_3$) δ 6.46 (d, *J* = 5.8 Hz, 3H), 2.98 (s, 6H), 2.35 (s, 6H).

**$^{13}$C NMR** (126 MHz, $CDCl_3$) δ 151.02, 138.68, 118.91, 110.89, 40.87, 21.87. The NMR data were in consistent with the reported data.[29]

***N,N*-Dimethylnaphthalen-2-amine (2am)**

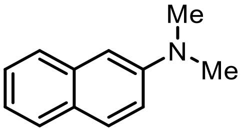


Following general procedure **(B)**, **2am** was obtained as white solid in 86% isolated yield from 2-bromonaphthalene using 80:1 petroleum ether/EtOAc as eluent.

**$^1$H NMR** (400 MHz, $CDCl_3$) δ 7.81 – 7.70 (m, 3H), 7.44 (t, *J* = 7.5 Hz, 1H), 7.28 (t, *J* = 7.5 Hz, 1H), 7.23 (dd, *J* = 9.0, 2.3 Hz, 1H), 7.00 (s, 1H), 3.10 (s, 6H).

**$^{13}$C NMR** (151 MHz, $CDCl_3$) δ 148.73, 135.06, 128.78, 127.55, 126.97, 126.30, 122.19, 116.58, 106.64, 41.02. The NMR data were in consistent with the reported data.[9]

**_N,N_-Dimethyl-1-naphthalenamine (2an)**

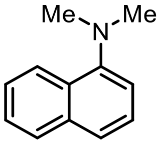


Following general procedure **(B)**, **2an** was obtained as white solid in 86% isolated yield from 1-bromonaphthalene using 80:1 petroleum ether/EtOAc as eluent.

**$^1$H NMR** (500 MHz, $CDCl_3$) δ 8.36 (d, *J* = 8.3 Hz, 1H), 7.92 (d, *J* = 7.6 Hz, 1H), 7.64 – 7.52 (m, 3H), 7.49 (t, *J* = 7.8 Hz, 1H), 7.16 (d, *J* = 7.4 Hz, 1H), 2.99 (s, 6H).

**$^{13}$C NMR** (126 MHz, $CDCl_3$) δ 150.97, 134.93, 128.93, 128.44, 125.87, 125.79, 125.21, 124.26, 122.97, 114.01, 45.29. The NMR data were in consistent with the reported data.[9]

**4-methoxy-_N,N_-dimethylnaphthalen-1-amine(2ao)**

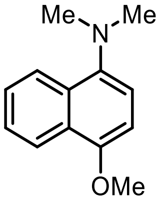


Following general procedure **(B)**, **2ao** was obtained as light yellow liquid in 86% isolated yield from 1-bromo-4-methoxynaphthalene using 10:1 petroleum ether/EtOAc as eluent.

**$^1$H NMR** (500 MHz, Chloroform-*d*) δ 8.24 (dd, *J* = 11.8, 8.3 Hz, 2H), 7.56 – 7.45 (m, 2H), 7.02 (d, *J* = 8.1 Hz, 1H), 6.74 (d, *J* = 8.1 Hz, 1H), 3.98 (s, 3H), 2.84 (d, *J* = 1.1 Hz, 6H).

**$^{13}$C NMR** (126 MHz, $CDCl_3$) δ 151.75, 144.23, 130.04, 126.66, 126.05, 125.33, 123.85, 122.41, 114.01, 103.48, 55.72, 45.73. The NMR data were in consistent with the reported data.[30]

**(6-(dimethylamino)naphthalen-2-yl)methanol (2ap)**

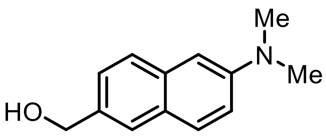


Following general procedure **(B)**, **2ap** was obtained as white solid in 68% isolated yield from (6-bromonaphthalen-2-yl)methanol using 5:1 petroleum ether/EtOAc as eluent.

**$^1$H NMR** (500 MHz, $CDCl_3$) δ 7.71 – 7.60 (m, 3H), 7.37 (dd, *J* = 8.4, 1.8 Hz, 1H), 7.17 (dd, *J* = 9.1,

2.6 Hz, 1H), 6.93 (d, $J$ = 2.6 Hz, 1H), 4.75 (s, 2H), 3.05 (s, 6H), 2.01 (s, 1H).

**$^{13}$C NMR** (126 MHz, $CDCl_3$) δ 147.69, 133.47, 133.42, 127.65, 125.63, 125.56, 124.81, 124.52, 115.65, 105.44, 64.66, 39.85. The NMR data were in consistent with the reported data.[31]

**_N,N_-Dimethylbenzo[_b_]thiophen-2-amine (2aq)**

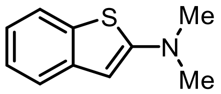


Following general procedure **(B)**, **2aq** was obtained as yellow oil in 57% isolated yield from 2-bromo-1-benzothiophene using 90:1 petroleum ether/EtOAc as eluent.

**$^{1}$H NMR** (500 MHz, $CDCl_3$) δ 7.55 (d, $J$ = 7.9 Hz, 1H), 7.40 (d, $J$ = 7.9 Hz, 1H), 7.23 – 7.17 (m, 1H), 7.04 – 6.99 (m, 1H), 5.95 (s, 1H), 2.97 (s, 6H).

**$^{13}$C NMR** (126 MHz, $CDCl_3$) δ 157.87, 141.56, 132.52, 124.55, 121.50, 120.44, 120.22, 96.65, 42.59. The NMR data were in consistent with the reported data.[17]

**_N,N_-Dimethylbenzo[_b_]thiophen-6-amine (2ar)**

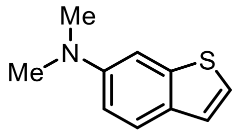


Following general procedure **(B)**, **2ar** was obtained as yellow solid in 83% isolated yield from 6-bromobenzothiophene using 60:1 petroleum ether/EtOAc as eluent.

**$^{1}$H NMR** (500 MHz, $CDCl_3$) δ 7.61 (d, $J$ = 8.8 Hz, 1H), 7.16 – 7.10 (m, 2H), 7.07 (d, $J$ = 5.4 Hz, 1H), 6.87 (dd, $J$ = 8.8, 2.3 Hz, 1H), 2.93 (s, 6H).

**$^{13}$C NMR** (126 MHz, $CDCl_3$) δ 148.57, 141.96, 130.93, 123.72, 123.40, 121.67, 112.63, 104.71, 41.22. The NMR data were in consistent with the reported data.[32]

**_N,N_-dimethylbenzofuran-7-amine (2as)**

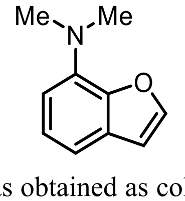


Following general procedure **(B)**, **2as** was obtained as colorless transparent liquid in 63% isolated yield from 7-bromobenzofuran using 40:1 petroleum ether/EtOAc as eluent.

**$^{1}$H NMR** (500 MHz, Chloroform-$d$) δ 7.63 (d, $J$ = 2.2 Hz, 1H), 7.16 (s, 2H), 6.76 (d, $J$ = 2.2 Hz, 1H), 6.70 (dd, $J$ = 5.8, 3.1 Hz, 1H), 3.08 (s, 6H).

**$^{13}$C NMR** (126 MHz, Chloroform-$d$) δ 145.35, 142.87, 137.34, 127.60, 122.56, 111.69, 109.34, 105.91, 41.30.

**IR (ATR):** ν = 2949.17, 2877.03, 2838.69, 2791.81, 1605.69, 1589.12, 1550.07, 1457.90, 1427.21, 1352.20, 1318.99, 1243.85, 1199.26, 1141.85, 1126.04, 1047.86, 917.73, 871.38, 825.34, 786.61, 725.82, 647.34, 617.18, 571.34, 547.50, 485.95 $cm^{-1}$.

**HRMS (ESI-TOF) m/z**: $[M+H]^+$ Calcd. for $C_{10}H_{11}NO$ 162.0914; Found 162.0908.

**1-Methyl-4-(dimethylamino)-1_H_-pyrrolo[2,3-_b_]pyridine (2at)**

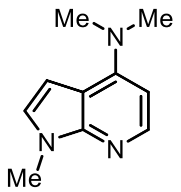

Following general procedure **(B)**, **2at** was obtained as off-white solid in 85% isolated yield from 4-bromo-1-methyl-1*H*-pyrrolo[2,3-*b*]pyridine using 2:1 petroleum ether/EtOAc as eluent.

**$^1$H NMR** (500 MHz, $CDCl_3$) δ 8.03 (d, *J* = 5.7 Hz, 1H), 6.90 (d, *J* = 3.7 Hz, 1H), 6.55 (d, *J* = 3.6 Hz, 1H), 6.14 (d, *J* = 5.7 Hz, 1H), 3.79 (s, 3H), 3.18 (s, 6H).

**$^{13}$C NMR** (126 MHz, $CDCl_3$) δ 150.97, 149.36, 144.09, 124.27, 108.50, 100.14, 99.03, 41.57, 31.51. The NMR data were in consistent with the reported data.[33]

**5-(*N,N*-Dimethylamino)-1-methylindole (2au)**

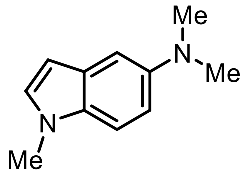

Following general procedure **(B)**, **2au** was obtained as white solid in 62% isolated yield from 5-bromo-1-methyl-1*H*-indole using 10:1 petroleum ether/EtOAc as eluent.

**$^1$H NMR** (500 MHz, $CDCl_3$) δ 7.23 (d, *J* = 8.9 Hz, 1H), 7.06 (d, *J* = 2.4 Hz, 1H), 6.99 (d, *J* = 3.0 Hz, 1H), 6.96 (dd, *J* = 8.9, 2.4 Hz, 1H), 6.39 (d, *J* = 3.0 Hz, 1H), 3.76 (s, 3H), 2.94 (s, 6H).

**$^{13}$C NMR** (126 MHz, $CDCl_3$) δ 145.99, 131.64, 129.24, 129.09, 112.96, 109.65, 105.40, 100.22, 43.19, 32.98. The NMR data were in consistent with the reported data.[34]

**6-(Dimethylamino)nicotinonitrile (2av)**

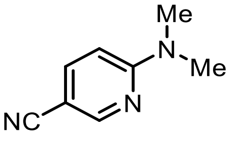

Following general procedure **(B)**, **2av** was obtained as yellow solid in 96% isolated yield from 2-bromo-5-cyanopyridine using 6:1 petroleum ether/EtOAc as eluent.

**$^1$H NMR** (500 MHz, $CDCl_3$) δ 8.35 (d, *J* = 2.3 Hz, 1H), 7.53 (dd, *J* = 9.0, 2.4 Hz, 1H), 6.45 (d, *J* = 9.0 Hz, 1H), 3.12 (s, 6H).

**$^{13}$C NMR** (126 MHz, $CDCl_3$) δ 159.65, 152.65, 139.28, 119.09, 105.20, 95.14, 37.97. The NMR data were in consistent with the reported data.[35]

***N,N*-Dimethyl-5-nitropyridin-2-amine (2aw)**

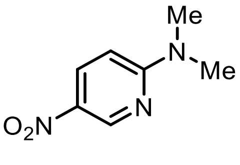

Following general procedure **(B)**, **2aw** was obtained as yellow solid in 83% isolated yield from 2-bromo-5-nitropyridine using 6:1 petroleum ether/EtOAc as eluent.

**$^{1}$H NMR** (500 MHz, $CDCl_3$) δ 8.99 (d, *J* = 2.8 Hz, 1H), 8.14 (dd, *J* = 9.5, 2.8 Hz, 1H), 6.43 (d, *J* = 9.5 Hz, 1H), 3.20 (s, 6H).

**$^{13}$C NMR** (126 MHz, $CDCl_3$) δ 160.89, 146.57, 134.57, 132.62, 104.21, 38.47. The NMR data were in consistent with the reported data.[12]

**6-(*N,N*-Dimethylamino)-2*H*-chromen-2-one (2ax)**

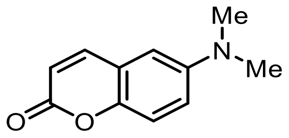


Following general procedure **(B)**, **2ax** was obtained as yellow solid in 43% isolated yield from 6-bromo-2*H*-chromen-2-one using 4:1 petroleum ether/EtOAc as eluent.

**$^{1}$H NMR** (500 MHz, $CDCl_3$) δ 7.63 (d, *J* = 9.5 Hz, 1H), 7.20 (d, *J* = 9.1 Hz, 1H), 6.95 (dd, *J* = 9.1, 3.0 Hz, 1H), 6.66 (d, *J* = 3.0 Hz, 1H), 6.37 (d, *J* = 9.5 Hz, 1H), 2.96 (s, 6H).

**$^{13}$C NMR** (126 MHz, $CDCl_3$) δ 161.56, 147.76, 146.30, 143.92, 119.32, 117.47, 117.33, 116.82, 109.30, 41.08. The NMR data were in consistent with the reported data.[36]

***N,N*-dimethylquinoxalin-2-amine (2ay)**

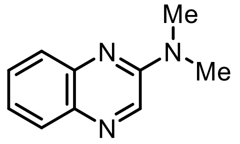


Following general procedure **(B)**, **2ay** was obtained as pale yellow solid in 70% isolated yield from 2-bromoquinoxaline using 20:1 petroleum ether/EtOAc as eluent.

**$^{1}$H NMR** (500 MHz, $CDCl_3$) δ 8.51 (s, 1H), 7.87 (d, *J* = 8.2 Hz, 1H), 7.69 (d, *J* = 8.3 Hz, 1H), 7.56 (t, *J* = 7.7 Hz, 1H), 7.35 (t, *J* = 7.4 Hz, 1H), 3.28 (s, 6H).

**$^{13}$C NMR** (126 MHz, $CDCl_3$) δ 151.44, 140.96, 135.32, 134.13, 128.96, 127.68, 125.27, 123.00, 36.77.The NMR data were in consistent with the reported data.[37]

***N,N*-Dimethylquinolin-7-amine (2az)**

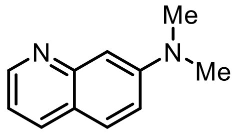


Following general procedure **(B)**, **2az** was obtained as yellow solid in 59% isolated yield from 7-bromoquinoline using 3:1 petroleum ether/EtOAc as eluent.

**$^{1}$H NMR** (500 MHz, $CDCl_3$) δ 8.72 (dd, *J* = 4.4, 1.8 Hz, 1H), 7.94 (dd, *J* = 8.0, 1.8 Hz, 1H), 7.61 (d, *J* = 9.8 Hz, 1H), 7.18 – 7.12 (m, 2H), 7.07 (dd, *J* = 8.1, 4.3 Hz, 1H), 3.07 (s, 6H).

**$^{13}$C NMR** (126 MHz, $CDCl_3$) δ 151.28, 150.66, 150.14, 135.50, 128.41, 121.05, 117.26, 116.37, 107.00, 40.54. The NMR data were in consistent with the reported data.[38]

***N,N*-dimethylisoquinolin-1-amine(2ba)**

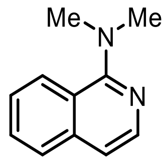

Following general procedure **(B)**, **2ba** was obtained as pale yellow oil in 55% isolated yield from 1-bromoisoquinoline using 10:1 petroleum ether/EtOAc as eluent.

**$^1$H NMR** (500 MHz, $CDCl_3$) δ 8.16 – 8.07 (m, 2H), 7.72 (d, *J* = 8.2 Hz, 1H), 7.62 – 7.55 (m, 1H), 7.51 – 7.44 (m, 1H), 7.16 (d, *J* = 5.7 Hz, 1H), 3.11 (s, 6H).

**$^{13}$C NMR** (126 MHz, $CDCl_3$) δ 162.12, 140.60, 138.45, 129.59, 127.08, 126.30, 125.67, 121.46, 114.81, 43.22.The NMR data were in consistent with the reported data.[39]

**6-Chloro-*N,N*-dimethylpyrazin-2-amine (2bb)**

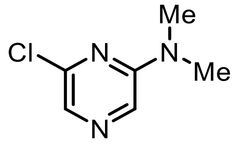

Following general procedure **(B)**, **2bb** was obtained as white solid in 76% isolated yield from 2-bromo-6-chloropyrazine using 6:1 petroleum ether/EtOAc as eluent.

**$^1$H NMR** (500 MHz, $CDCl_3$) δ 7.85 (s, 1H), 7.74 (s, 1H), 3.11 (s, 6H).

**$^{13}$C NMR** (126 MHz, $CDCl_3$) δ 154.43, 146.69, 129.45, 127.20, 37.75. The NMR data were in consistent with the reported data.[12]

**5-(*N,N*-Dimethylamino)-2-chloropyrimidine (2bc)**

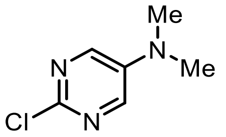

Following general procedure **(B)**, **2bc** was obtained as off-white solid in 60% isolated yield from 5-bromo-2-chloropyrimidine using 3:1 petroleum ether/EtOAc as eluent.

**$^1$H NMR** (500 MHz, $CDCl_3$) δ 8.02 (s, 2H), 2.98 (s, 6H).

**$^{13}$C NMR** (126 MHz, $CDCl_3$) δ 148.02, 142.46, 39.73. The NMR data were in consistent with the reported data.[12]

**Methyl-2-((*tert*-butoxycarbonyl)amino)-3-(4-(dimethylamino)phenyl)propanoate (3a)**

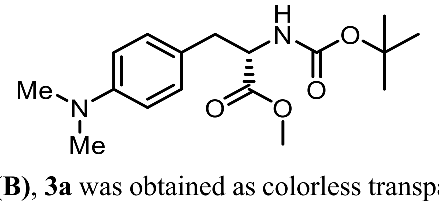

Following general procedure **(B)**, **3a** was obtained as colorless transparent oil in 85% isolated yield from methyl (S)-3-(4-bromophenyl)-2-((*tert*-butoxycarbonyl)amino)propanoate using 10:1 petroleum ether/EtOAc as eluent.

**$^1$H NMR** (500 MHz, $CDCl_3$) δ 6.98 (d, *J* = 8.5 Hz, 2H), 6.67 (d, *J* = 8.7 Hz, 2H), 4.97 (d, *J* = 8.3 Hz, 1H), 4.51 (dt, *J* = 8.4, 5.8 Hz, 1H), 3.71 (s, 3H), 2.99 (t, *J* = 5.2 Hz, 2H), 2.91 (s, 6H), 1.42 (s,

9H).

**$^{13}$C NMR** (126 MHz, $CDCl_3$) δ 172.73, 155.28, 149.72, 130.01, 123.71, 112.93, 79.84, 54.71, 52.19, 40.76, 37.33, 28.41.

**IR (ATR):** ν = 3343.25, 2976.78, 1741.70, 1709.50, 1674.77, 1612.30, 1437.29, 1364.60, 1249.78, 1216.24, 1160.68, 1113.24, 1053.84, 1018.04, 808.17, 732.80, 552.58, 416.64 $cm^{-1}$.

**HRMS (ESI-TOF) m/z**: $[M+Na]^+$ Calcd. for $C_{17}H_{26}N_2O_4$ 345.1785; Found 345.1786.

The enantiomeric excess value was determined by HPLC analysis (chiralcel AD-H column, 90:1 hexane/ i-PrOH, 1mL/min, 254nm, 25 °C) tR = 14.246 min (minor), tR = 15.607 min (major), ee = 95 %.

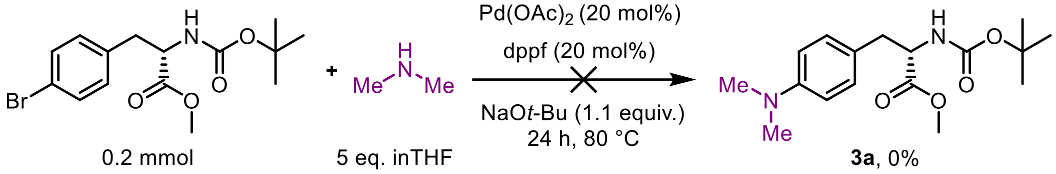


**Figure S2.** Reaction under classic Buchwald-Hartwig C-N coupling conditions

Compared with the standard conditions we had built up, only decomposition was detected instead of desired *ee* retention product under classic Buchwald-Hartwig C-N coupling conditions.

**2-chloro-*N*-(4-(dimethylamino)phenyl)acetamide (3b)**

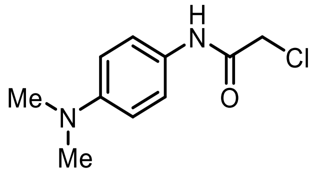

Following general procedure **(B)**, **3b** was obtained as gary solid in 57% isolated yield from *N*-(4-bromophenyl)-2-chloroacetamide using 20:1 petroleum ether/EtOAc as eluent.

**Melting point**:150.89-151.33°C

**$^{1}$H NMR** (500 MHz, $CDCl_3$) δ 8.15 – 8.00 (m, 1H), 7.37 (d, *J* = 9.1 Hz, 2H), 6.71 (d, *J* = 9.0 Hz, 2H), 4.17 (s, 2H), 2.94 (s, 6H).

**$^{13}$C NMR** (126 MHz, $CDCl_3$) δ 163.65, 148.72, 126.34, 122.22, 112.99, 43.03, 40.91.

**IR (neat)**: ν = 3746.96, 3272.41, 3119.59, 2886.78, 2806.11, 1867.43, 1659.97, 1614.04, 1605,47, 1540.78, 1521.64, 1433.25, 1407.92, 1339.76, 1295.90, 1253.70, 1227.72, 1062.34, 946.97, 816.56 $cm^{-1}$.

**HRMS (ESI-TOF) m/z**: $[M+H]^+$ Calcd. for $C_{10}H_{13}ClN_2O$ 213.0789; Found 213.0791.

**Ethyl 2-(dimethylamino)oxazole-4-carboxylate (3c)**

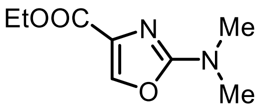

Following general procedure **(B)**, **3c** was obtained as off-white solid in 79% isolated yield from ethyl 2-bromooxazole-4-carboxylate using 2:1 petroleum ether/EtOAc as eluent.

**$^{1}$H NMR** (500 MHz, $CDCl_3$) δ 7.69 (s, 1H), 4.27 (q, *J* = 7.1 Hz, 2H), 3.02 (s, 6H), 1.28 (t, *J* = 7.1

Hz, 3H).

**$^{13}$C NMR** (126 MHz, $CDCl_3$) δ 162.33, 162.07, 137.74, 133.44, 60.78, 37.77, 14.29. The NMR data were in consistent with the reported data.[12]

**_N,N_-Dimethylbenzo[_d_]thiazol-2-amine (3d)**

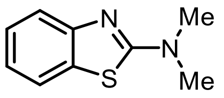

Following general procedure **(B)**, **3d** was obtained as white solid in 83% isolated yield from ethyl 2-bromooxazole-4-carboxylate using 6:1 petroleum ether/EtOAc as eluent.

**$^{1}$H NMR** (500 MHz, $CDCl_3$) δ 7.58 (dd, $J$ = 11.1, 8.2 Hz, 2H), 7.28 (t, $J$ = 7.7 Hz, 1H), 7.05 (t, $J$ = 7.6 Hz, 1H), 3.19 (s, 6H).

**$^{13}$C NMR** (126 MHz, $CDCl_3$) δ 168.88, 153.36, 131.23, 126.03, 120.99, 120.72, 118.87, 40.28. The NMR data were in consistent with the reported data.[42]

**_N,N_,1-trimethyl-1_H_-indazol-3-amine (3e)**

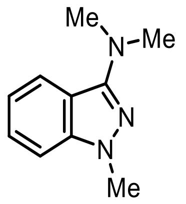

Following general procedure **(B)**, **3e** was obtained as white solid in 53% isolated yield from 3-bromo-1-methyl-1H-indazole using 20:1 petroleum ether/EtOAc as eluent.

**$^{1}$H NMR** (500 MHz, $CDCl_3$) δ 7.76 (d, $J$ = 8.2 Hz, 1H), 7.35 – 7.29 (m, 1H), 7.21 (d, $J$ = 8.6 Hz, 1H), 7.01 – 6.95 (m, 1H), 3.88 (d, $J$ = 2.0 Hz, 3H), 3.09 (d, $J$ = 2.0 Hz, 6H).

**$^{13}$C NMR** (126 MHz, $CDCl_3$) δ 142.32, 126.52, 121.92, 118.22, 115.28, 108.78, 41.96, 34.99.

**IR (ATR):** v = 3424.76, 2947.01, 2851.96, 2791.63, 1611.83, 1573.24, 1534.65, 1495.16, 1450.03, 1351.10, 1295.39, 1265.09, 1224.03, 1164.67, 1128.73, 1080.01, 1002.69, 966.96, 925.60, 834.65, 765.68, 736.15, 666.40, 624.63, 609.30, 555.17, 429.48 $cm^{-1}$.

**HRMS (ESI-TOF) m/z**: $[M+H]^+$ Calcd. for $C_{10}H_{13}N_3$ 176.1182; Found 176.1180.

**Metronidazole derivative (3f)**

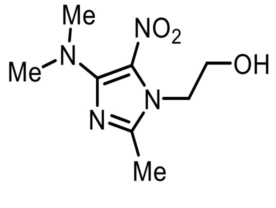

2-(4-bromo-2-methyl-5-nitro-1_H_-imidazol-1-yl)ethan-1-ol was synthesized according to literature precedent.[43] This product was obtained as white solid. The NMR data were in consistent with the reported data.[43]

**$^{1}$H NMR** (500 MHz, $CDCl_3$) δ 4.15 (t, $J$ = 5.1 Hz, 2H), 4.03 (t, $J$ = 5.1 Hz, 2H), 2.53 (s, 3H).

Following general procedure **(B)**, **3f** was obtained as light yellow solid in 70% isolated yield from 2-(4-bromo-2-methyl-5-nitro-1_H_-imidazol-1-yl)ethan-1-ol using 2:1 petroleum ether/EtOAc as

eluent.

**Melting point:** 154.16-154.23 °C

**$^1$H NMR** (500 MHz, $CDCl_3$) δ 3.98 (t, *J* = 5.0 Hz, 2H), 3.90 (t, *J* = 5.3 Hz, 3H), 2.78 (s, 6H), 2.38 (s, 3H).

**$^{13}$C NMR** (126 MHz, $CDCl_3$) δ 141.58, 141.18, 138.40, 61.27, 46.03, 42.11, 14.41.

**IR (neat)**: ν = 3468.15, 3273.50, 2950.45, 1878.97, 1575.02, 1539.08, 1497.26, 1414.53, 1381.72, 1365.66, 1062.39 $cm^{-1}$.

**HRMS (ESI-TOF) m/z**: $[M+H]^+$ Calcd. for $C_8H_{14}N_4O_3$ 215.1139; Found 215.1139.

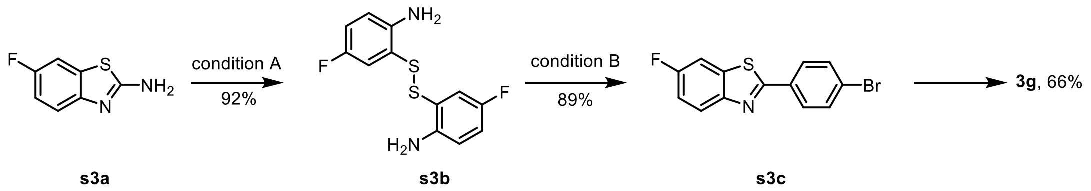


**Figure S3.** three-step synthesis of PET tracer analog 3g

**Procedure:**

2,2'-disulfanediylbis(4-fluoroaniline) **(s3b)** (92% isolated yield) was synthesized according to literature precedent: [44]A suspension of 6-fluorobenzo[d]thiazol-2-amine **(s3a)** (1.5 g, 10 mmol) and ethylene glycol (2.2 mL, 40 mmol) in aqueous KOH (3.0 M, 100 mL) was stirred at 140 °C for 24 h. After cooling of the mixture, it was neutralized with concentrated HCl on an ice bath, and the precipitate was filtered off and washed with EtOAc. The filtrate was partitioned between EtOAc (200 mL) and $H_2O$ (200 mL). The water layer was extracted with EtOAc (three times), and the combined organic layer was dried over anhydrous $Na_2SO_4$, filtered and concentrated *in vacuo*. The reaction mixture was purified by fresh silica gel chromatography to afford the desired product.

**$^1$H NMR** (500 MHz, $CDCl_3$) δ 6.97 – 6.87 (m, 4H), 6.67 (dd, *J* = 8.6, 4.8 Hz, 2H), 4.17 (s, 4H). The NMR data were in consistent with the reported data.[44]

**Procedure:**

2-(4-bromophenyl)-6-fluorobenzo[d]thiazole **(s3c)** (91% isolated yield) was synthesized according to literature precedent:[45] 2,2'-disulfanediylbis(4-fluoroaniline) **(s3b)** (3.2 mmol), 4-Bromobenzaldehyde (3.26 mmol) and sodium metabisulfite (3.26 mmol) were dissolved in anhydrous DMSO (18.6 mL). The resulting reaction mixture was stirred at 120 °C for 2 h and the formation of the desired compound was monitored by TLC analysis. On completion of reaction the mixture was cooled to room temperature, water was added and the resulting precipitate was collected by vacuum filtration. The precipitate was then washed with excess water, re-dissolved in DCM (30 mL) and the remaining traces of sodium metabisulfite were removed by washing with brine (30 mL). the combined organic layer was dried over anhydrous $Na_2SO_4$, filtered and concentrated *in vacuo*. The reaction mixture was purified by fresh silica gel chromatography to afford the desired product.

**$^1$H NMR** (500 MHz, $CDCl_3$) δ 8.00 (dd, *J* = 9.0, 4.8 Hz, 1H), 7.96 – 7.89 (m, 2H), 7.63 (d, *J* = 8.5 Hz, 2H), 7.59 (dd, *J* = 8.1, 2.6 Hz, 1H), 7.23 (dd, *J* = 8.9, 2.7 Hz, 1H). The NMR data were in

consistent with the reported data.[45]

**4-(6-fluorobenzo[d]thiazol-2-yl)-*N,N*-dimethylaniline (3g)**

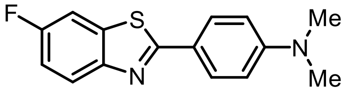


Following general procedure **(B)**, **3g** was obtained as white solid in 80% isolated yield from 2-(4-bromophenyl)-6-fluorobenzothiazole using 30:1 petroleum ether/EtOAc as eluent.

**$^1$H NMR** (500 MHz, $CDCl_3$) δ 7.97 – 7.85 (m, 3H), 7.52 (dd, *J* = 8.2, 2.7 Hz, 1H), 7.16 (td, *J* = 8.9, 2.6 Hz, 1H), 6.74 (d, *J* = 8.9 Hz, 2H), 3.06 (s, 6H).

**$^{13}$C NMR** (126 MHz, $CDCl_3$) δ 161.09 (d, *J* = 3.2 Hz), 159.12 (d, *J* = 242.3 Hz), 152.38, 151.22 (d, *J* = 1.88 Hz), 135.66 (d, *J* = 11.1 Hz), 128.92, 123.15 (d, *J* = 9.3 Hz), 121.34, 114.49 (d, *J* = 24.5 Hz), 111.88, 107.80 (d, *J* = 26.8 Hz), 40.32.

**$^{19}$F NMR** (471 MHz, $CDCl_3$) δ -117.63.The NMR data were in consistent with the reported data.[46]

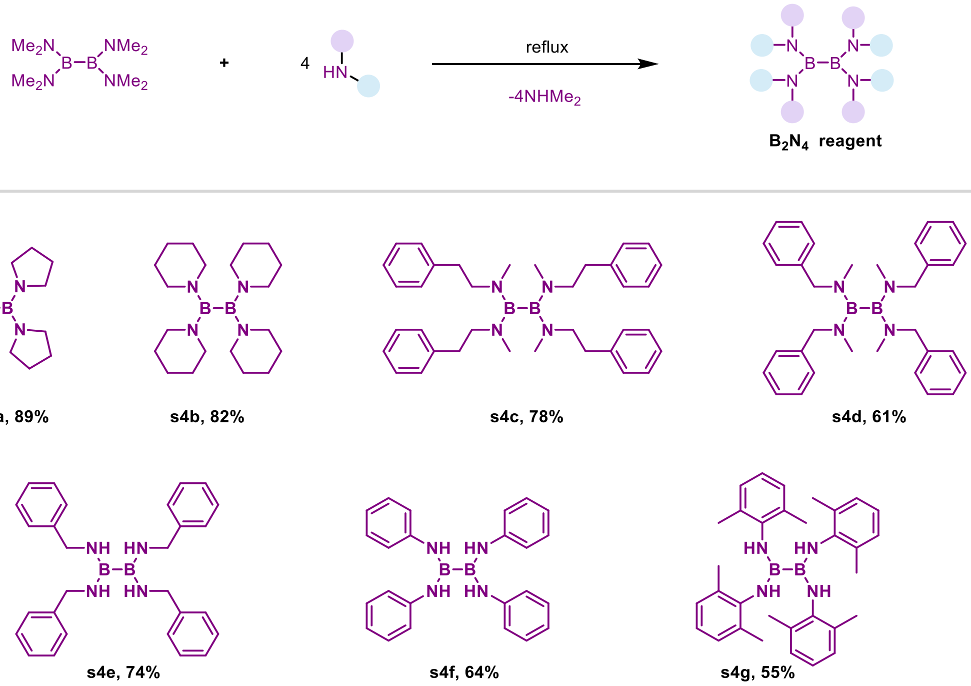


**Figure S4.** $B_2N_4$ reagents preparation from $B_2(NMe_2)_4$.

**General procedure (C):**

In an oven-dried 100 mL round-bottom flask, $B_2(NMe_2)_4$ (1.0 equiv., 20 mmol), amine (4.95 equiv., 99 mmol) and a magnetic stir bar were added under an argon atmosphere. Then the mixture was heated to gentle reflux for 24 h. After completion of the reaction, the reaction mixture was allowed to stand and cool at room temperature, and solids precipitated out. Subsequently, it was filtered and then rotary evaporated to obtain the target product.

***Notes*:**

All experiments were performed under strictly anhydrous and oxygen-free conditions.

**General procedure (D):**

A solution of $B_2(NMe_2)_4$ (1.0 equiv., 20 mmol) and amine (4.95 equiv., 99 mmol) in toluene (50 mL)

was refluxed under argon atmosphere for 24 h. After completion of the reaction, the solvent in the reaction system was removed by a rotary evaporator. Add hexane into the mixture and then solids precipitated out. The reaction mixture was filtered and the solid product was dried under vacuum to give tetra-(amine)-diborane.

***Notes*:**

All experiments were performed under strictly anhydrous and oxygen-free conditions.

**General procedure (E):**

An oven-dried 4 mL vial was charged with a magnetic stir bar, aryl bromides or aryl chlorides (0.2 mmol, 1.0 equiv.), $Ni(acac)_2$ (0.02 mmol, 10 mol%), $B_2N_4$ diboron reagents (0.2 mmol, 1.0 equiv.), DMF (0.5 mL) in the glove box. The vial was sealed with a plastic cap and then stirring was achieved by placing the assembled reactor at 80 °C on IKA C-MAG HS 7 control magnetic stir bars for 24 h. After reaction completion, the reaction was quenched with $H_2O$ and diluted with EtOAc. The resulting mixture was separated and extracted with EtOAc (three times). The combined organic layer was dried over anhydrous $Na_2SO_4$, filtered and concentrated *in vacuo*. The reaction mixture was purified by fresh silica gel chromatography to afford the desired product.

**Tetra-(pyrrolidine)-diborane (s4a)**

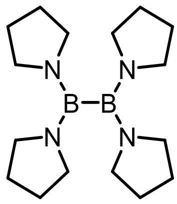


Following general procedure **(C)**, **s4a** was obtained as white solid in 89% isolated yield**.**

**$^1$H NMR** (500 MHz, $CDCl_3$) δ 3.23 (s, 16H), 1.65 (d, $J$ = 6.2 Hz, 16H).

**$^{13}$C NMR** (126 MHz, $CDCl_3$) δ 49.27, 26.41.

**$^{11}$B NMR** (161 MHz, $CDCl_3$) δ 34.94.The NMR data were in consistent with the reported data.[47]

**Tetra-(piperidine)-diborane (s4b)**

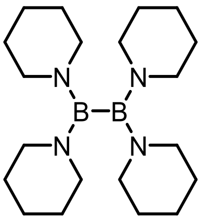


Following general procedure **(C), s4b** was obtained as white solid in 82% isolated yield**.**

**$^1$H NMR** (500 MHz, $CDCl_3$) δ 2.99 (t, $J$ = 5.3 Hz, 16H), 1.57 (p, $J$ = 5.8 Hz, 8H), 1.40 (p, $J$ = 5.7 Hz, 16H).

**$^{13}$C NMR** (126 MHz, $CDCl_3$) δ 50.06, 28.20, 25.83.

**$^{11}$B NMR** (161 MHz, $CDCl_3$) δ 34.45.The NMR data were in consistent with the reported data.[47]

**Tetra-(*N*-methyl-2-phenylethan-1-amine)-diboron (s4c)**

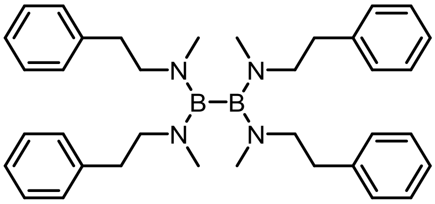

Following general procedure **(D)**, **s4c** was obtained as pale yellow solid in 78% isolated yield**.**

**Melting point:** 186.38-186.41 °C

**$^{1}$H NMR** (500 MHz, DMSO-$d_6$) δ 7.33 – 7.20 (m, 20H), 2.87 (t, $J$ = 8.0 Hz, 8H), 2.77 (t, $J$ = 7.8 Hz, 8H), 2.41 (d, $J$ = 1.9 Hz, 12H).

**$^{13}$C NMR** (126 MHz, DMSO-$d_6$) δ 139.84, 129.08, 128.83, 126.59, 52.38, 35.37, 34.74.

**$^{11}$B NMR** (161 MHz, DMSO-$d_6$) δ 1.50.

**IR (neat):** ν = 3414.33, 3020.52, 2948.67, 2786.85, 2437.68, 1481.25, 1454.67, 1436.53, 1359.01, 1053.83, 946.42, 979.02, 879.02, 813.31, 750.07, 696.49, 582.82, 510.20 cm$^{-1}$.

**HRMS (ESI-TOF) m/z**: [M+K]$^+$ Calcd. for $C_{36}H_{48}B_2N_4$ 597.3697; Found 597.3704.

**Tetra-(*N*-Methylbenzylamine)-diboron (s4d)**

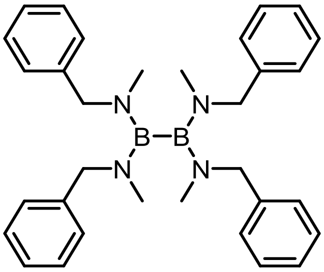

Following general procedure **(D)**, **s4d** was obtained as pale yellow solid in 61% isolated yield**.**

**Melting point:** 175.77-179.18 °C

**$^{1}$H NMR** (500 MHz, DMSO-$d_6$) δ 7.37 (s, 20H), 3.83 (s, 8H), 2.38 (s, 12H).

**$^{13}$C NMR** (126 MHz, DMSO-$d_6$) δ 137.58, 129.17, 128.83, 128.02, 54.04, 34.65.

**$^{11}$B NMR** (161 MHz, DMSO-$d_6$) δ 1.50.

**IR (neat)**: ν = 3412.4, 3030.94, 3019.07, 2934.5, 2835.45, 2791.42, 2757.62, 2699.18, 2535.46, 2412.2, 1646, 1424.54, 1350.4, 1212.62, 1074.55, 1023.67, 961.45, 856.84, 751.79, 698.88 cm$^{-1}$

**HRMS (ESI-TOF) m/z**: [M+K]$^+$ Calcd. for $C_{32}H_{40}B_2N_4$ 525.3331; Found 525.3325.

**Tetra-(Benzylamine)-diboron (s4e)**

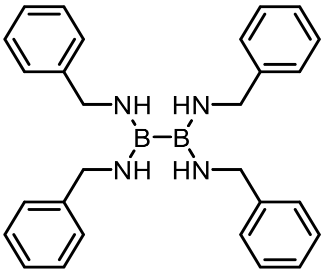

Following general procedure **(D), s4e** was obtained as pale yellow solid in 74% isolated yield**.**

**$^{1}$H NMR** (500 MHz, DMSO-$d_6$) δ 7.37 – 7.21 (m, 20H), 4.44 (s, 4H), 3.78 (s, 8H).

**$^{13}$C NMR** (126 MHz, DMSO-$d_6$) δ 142.17, 128.67, 127.92, 127.18, 45.25.

**$^{11}$B NMR** (161 MHz, DMSO-$d_6$) δ 33.12.The NMR data were in consistent with the reported data.[48]

**Tetra-(aniline)-diboron (s4f)**

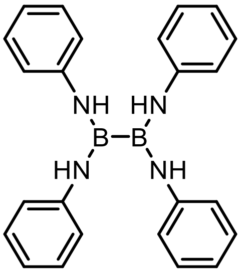


Following general procedure **(D)**, **s4f** was obtained as white solid in 64% isolated yield**.**

**$^1$H NMR** (500 MHz, DMSO-$d_6$) δ 7.16 – 6.91 (m, 20H), 6.68 (s, 4H).

**$^{13}$C NMR** (126 MHz, DMSO-$d_6$) δ 146.38, 129.52, 120.03, 117.26.

**$^{11}$B NMR** (161 MHz, DMSO-$d_6$) δ 33.61.The NMR data were in consistent with the reported data.[48]

**Tetra-(2,6-Dimethylaniline)-diboron (s4g)**

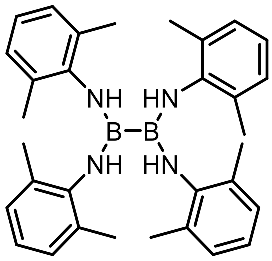


Following general procedure **(D)**, **s4g** was obtained as white solid in 55% isolated yield**.**

**$^1$H NMR (**500 MHz, DMSO-$d_6$**)** δ 6.89 (d, $J$ = 7.4 Hz, 8H), 6.50 (t, $J$ = 7.4 Hz, 4H), 4.57 (s, 4H), 2.17 (s, 24H).

**$^{13}$C NMR (**126 MHz, DMSO**)** δ 144.59, 128.19, 120.99, 116.24, 18.26.

**$^{11}$B NMR** (161 MHz, DMSO-$d_6$) δ 32.94.The NMR data were in consistent with the reported data.[48]

**1-phenylpyrrolidine (4a)**

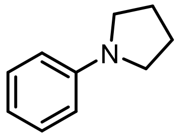


Following general procedure **(E)**, **4a** was obtained as pale yellow oil in 81% isolated yield from bromobenzene using 40:1 petroleum ether/EtOAc as eluent.

**$^1$H NMR** (500 MHz, $CDCl_3$) δ 7.24 – 7.18 (m, 2H), 6.67 – 6.62 (m, 1H), 6.58 – 6.53 (m, 2H), 3.31 – 3.21 (m, 4H), 2.02 – 1.93 (m, 4H).

**$^{13}$C NMR** (126 MHz, $CDCl_3$) δ 147.96, 129.11, 115.35, 111.62, 47.55, 25.46.The NMR data were in consistent with the reported data.[49]

**1-(4-(methylthio)phenyl)pyrrolidine (4b)**

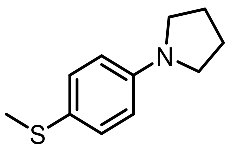

Following general procedure **(E)**, **4b** was obtained as light yellow solid in 78% isolated yield from (4-bromophenyl)(methyl)sulfane using 40:1 petroleum ether/EtOAc as eluent.

**$^{1}$H NMR** (500 MHz, $CDCl_3$) δ 7.34 – 7.28 (m, 2H), 6.53 (dt, *J* = 8.9, 2.8 Hz, 2H), 3.29 (tt, *J* = 6.5, 2.5 Hz, 4H), 2.43 (dd, *J* = 3.1, 1.3 Hz, 3H), 2.01 (td, *J* = 6.5, 2.5 Hz, 4H).

**$^{13}$C NMR** (126 MHz, $CDCl_3$) δ 146.96, 132.16, 121.67, 112.21, 47.60, 25.45, 19.84.The NMR data were in consistent with the reported data.[50]

**phenyl(3-(pyrrolidin-1-yl)phenyl)methanone (4c)**

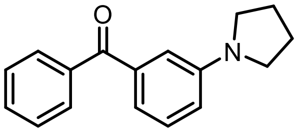

Following general procedure **(E)**, **4c** was obtained as light yellow oil in 88% isolated yield from (3-bromophenyl)(phenyl)methanone using 40:1 petroleum ether/EtOAc as eluent.

**$^{1}$H NMR** (500 MHz, $CDCl_3$) δ 7.83 (d, *J* = 6.9 Hz, 2H), 7.58 – 7.53 (m, 1H), 7.46 (t, *J* = 7.7 Hz, 2H), 7.29 (dd, *J* = 8.5, 7.2 Hz, 1H), 7.01 – 6.97 (m, 2H), 6.77 (ddd, *J* = 8.3, 2.5, 1.1 Hz, 1H), 3.36 – 3.26 (m, 4H), 2.06 – 1.98 (m, 4H).

**$^{13}$C NMR** (126 MHz, $CDCl_3$) δ 197.62, 147.80, 138.38, 138.13, 132.12, 130.10, 128.73, 128.07, 117.64, 115.55, 112.49, 47.71, 25.48.The NMR data were in consistent with the reported data.[49]

**2-(piperidin-1-yl)quinoxaline (4d)**

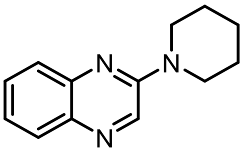

Following general procedure **(E)**, **4d** was obtained as light yellow oil in 65% isolated yield from 2-bromoquinoxaline 40:1 petroleum ether/EtOAc as eluent.

**$^{1}$H NMR** (500 MHz, $CDCl_3$) δ 8.58 (s, 1H), 7.85 (dd, *J* = 8.3, 1.5 Hz, 1H), 7.66 (dd, *J* = 8.4, 1.4 Hz, 1H), 7.55 (ddd, *J* = 8.4, 6.9, 1.5 Hz, 1H), 7.39 – 7.33 (m, 1H), 3.77 (t, *J* = 4.2 Hz, 4H), 1.72 (d, *J* = 2.5 Hz, 6H).

**$^{13}$C NMR** (126 MHz, $CDCl_3$) δ 152.48, 141.91, 136.46, 136.05, 129.93, 128.58, 126.35, 124.30, 45.88, 25.68, 24.63. The NMR data were in consistent with the reported data.[51]

***N*-benzyl-*N*-methyl-4-nitroaniline (4e)**

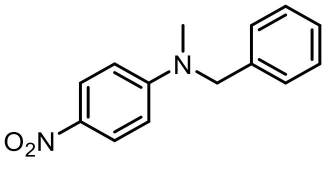

Following general procedure **(E)**, **4e** was obtained as light yellow solid in 56% isolated yield from 1-bromo-4-nitrobenzene using 20:1 petroleum ether/EtOAc as eluent.

**$^1$H NMR** (500 MHz, $CDCl_3$) δ 8.03 (dt, *J* = 9.3, 2.3 Hz, 2H), 7.34 – 7.19 (m, 3H), 7.09 (d, *J* = 7.5 Hz, 2H), 6.59 (dt, *J* = 9.3, 2.2 Hz, 2H), 4.60 (s, 2H), 3.12 (d, *J* = 1.3 Hz, 3H).

**$^{13}$C NMR** (126 MHz, $CDCl_3$) δ 152.83, 136.37, 135.64, 127.94, 126.53, 125.25, 125.20, 109.56, 55.12, 38.12.The NMR data were in consistent with the reported data.[52]

**_N_-methyl-_N_-phenethylbenzo[d]thiazol-2-amine (4f)**

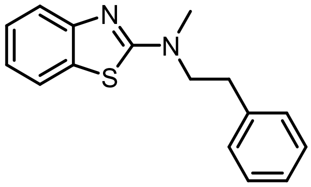


Following general procedure **(E)**, **4f** was obtained as red brown oil in 85% isolated yield from 2-bromobenzo[d]thiazole using 20:1 petroleum ether/EtOAc as eluent.

**$^1$H NMR** (500 MHz, $CDCl_3$) δ 7.60 – 7.55 (m, 2H), 7.28 (dt, *J* = 7.9, 6.3 Hz, 3H), 7.24 – 7.19 (m, 3H), 7.06 – 7.01 (m, 1H), 3.74 – 3.66 (m, 2H), 3.06 (s, 3H), 3.00 – 2.94 (m, 2H).

**$^{13}$C NMR** (126 MHz, $CDCl_3$) δ 167.79, 153.24, 138.62, 130.94, 128.83, 128.61, 126.51, 125.86, 120.87, 120.57, 118.79, 55.27, 38.64, 33.53.The NMR data were in consistent with the reported data.[53]

**2-((6-chloro-3-methyl-2,4-dioxo-3,4-dihydropyrimidin-1(2*H*)-yl)methyl)benzonitrile (4g)**

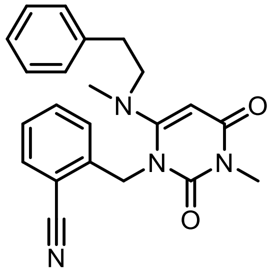


Following general procedure **(E)**, **4g** was obtained as pale yellow oil in 90% isolated yield from 2-((6-chloro-3-methyl-2,4-dioxo-3,4-dihydropyrimidin-1(2*H*)-yl)methyl)benzonitrile using 1 ： 1 petroleum ether/EtOAc as eluent.

**$^1$H NMR** (500 MHz, $CDCl_3$) δ 7.65 (d, *J* = 7.7 Hz, 1H), 7.51 (d, *J* = 7.8 Hz, 1H), 7.36 (s, 1H), 7.23 – 7.17 (m, 2H), 7.15 (d, *J* = 7.2 Hz, 1H), 7.08 (d, *J* = 8.0 Hz, 1H), 6.97 (d, *J* = 7.4 Hz, 2H), 5.38 (d, *J* = 1.5 Hz, 1H), 5.19 (s, 2H), 3.31 (d, *J* = 1.5 Hz, 3H), 3.10 (t, *J* = 7.8 Hz, 2H), 2.81 – 2.61 (m, 6H).

**$^{13}$C NMR** (126 MHz, $CDCl_3$) δ 161.92, 158.62, 151.85, 139.74, 136.81, 132.15, 131.99, 127.61, 127.39, 126.80, 125.69, 125.56, 116.05, 110.09, 89.78, 54.95, 45.63, 39.31, 31.75, 26.94.

**IR (ATR):** ν = 2924.05, 2222.29, 1697.72, 1643.12, 1598.11, 1436.45, 1361.08, 1311.15, 1280.68, 1200.74, 1118.83, 1066.49, 1030.44, 949.05, 807.15, 762.20, 700.84, 647.81, 609.09, 556.18, 515.27, 416.69 $cm^{-1}$.

**HRMS (ESI-TOF) m/z**: $[M+H]^+$ Calcd. for $C_{22}H_{22}N_4O_2$ 375.1816; Found 375.1801.

**methyl 4-(phenylamino)benzoate (4h)**

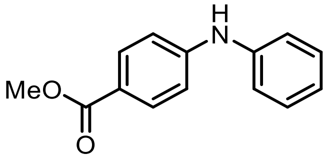

Following general procedure **(E)**, **4h** was obtained as yellow oil in 54% isolated yield from methyl 4-bromobenzoate using 40:1 petroleum ether/EtOAc as eluent.

**$^1$H NMR** (500 MHz, $CDCl_3$) δ 7.66 (t, *J* = 2.0 Hz, 1H), 7.52 (dt, *J* = 7.6, 1.4 Hz, 1H), 7.25 – 7.17 (m, 4H), 7.05 – 7.01 (m, 2H), 6.91 (tt, *J* = 7.4, 1.1 Hz, 1H), 5.78 (s, 1H), 4.30 (q, *J* = 7.1 Hz, 2H), 1.32 (t, *J* = 7.1 Hz, 3H).

**$^{13}$C NMR** (126 MHz, $CDCl_3$) δ 166.67, 143.52, 142.48, 131.72, 129.49, 129.30, 121.79, 121.68, 121.38, 118.33, 118.31, 61.03, 14.35.The NMR data were in consistent with the reported data.[54]

**4-phenoxy-*N*-phenylaniline (4i)**

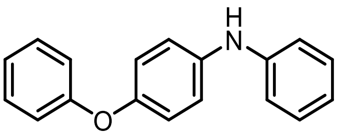

Following general procedure **(E)**, **4i** was obtained as yellow solid in 70% isolated yield from 1-bromo-4-phenoxybenzene using 30:1 petroleum ether/EtOAc as eluent.

**$^1$H NMR** (500 MHz, $CDCl_3$) δ 7.24 (t, *J* = 7.8 Hz, 2H), 7.17 (t, *J* = 7.8 Hz, 2H), 7.02 – 6.97 (m, 3H), 6.95 – 6.85 (m, 6H), 6.82 (t, *J* = 7.4 Hz, 1H), 5.53 (s, 1H).

**$^{13}$C NMR** (126 MHz, $CDCl_3$) δ 157.15, 150.20, 142.92, 137.75, 128.62, 128.35, 121.61, 119.48, 119.35, 116.92, 115.84. The NMR data were in consistent with the reported data.[55]

***N*-phenylbenzo[b]thiophen-5-amine (4j)**

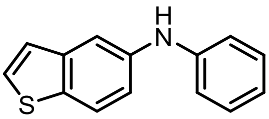

Following general procedure **(E)**, **4j** was obtained as yellow oil in 63% isolated yield from 5-bromobenzo[b]thiopheneusing 40:1 petroleum ether/EtOAc as eluent.

**$^1$H NMR** (500 MHz, $CDCl_3$) δ 7.77 (d, *J* = 8.6 Hz, 1H), 7.56 (d, *J* = 2.2 Hz, 1H), 7.44 (d, *J* = 5.4 Hz, 1H), 7.33 – 7.27 (m, 2H), 7.23 (d, *J* = 5.4 Hz, 1H), 7.13 (dd, *J* = 8.7, 2.2 Hz, 1H), 7.11 – 7.06 (m, 2H), 6.94 (t, *J* = 7.4 Hz, 1H), 5.75 (s, 1H).

**$^{13}$C NMR** (126 MHz, $CDCl_3$) δ 142.86, 139.72, 139.00, 131.98, 128.35, 126.32, 122.43, 122.05, 119.64, 116.92, 116.16, 111.26.The NMR data were in consistent with the reported data.[56]

**3-((2,6-dimethylphenyl)amino)phenol (4k)**

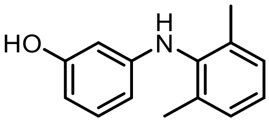

Following general procedure **(E)**, **4k** was obtained as white solid in 47% isolated yield from 3-bromophenol using 40:1 petroleum ether/EtOAc as eluent.

**Melting point:** 97.13-97.46 °C

**$^1$H NMR** (500 MHz, $CDCl_3$) δ 7.11 (q, $J$ = 5.4 Hz, 3H), 7.02 (t, $J$ = 8.0 Hz, 1H), 6.19 (ddd, $J$ = 25.6, 8.0, 2.3 Hz, 2H), 5.91 (t, $J$ = 2.3 Hz, 1H), 5.17 (s, 1H), 4.56 (s, 1H), 2.22 (s, 6H).

**$^{13}$C NMR** (126 MHz, $CDCl_3$) δ 155.69, 147.06, 136.91, 135.20, 129.21, 127.51, 125.00, 105.61, 104.02, 99.05, 17.28.

**IR (neat):** ν = 3387.49, 3283.76, 3033.10, 2918.85, 1616.30, 1589.28, 1472.49, 1438.38, 1376.22, 1330.07, 1229.50, 1212.03, 1174.77, 1156.82, 1094.95, 969.98, 832.47, 764.51, 688.88 $cm^{-1}$.

**HRMS (ESI-TOF) m/z**: $[M+H]^+$ Calcd. for $C_{14}H_{15}NO$ 214.1226; Found 214.1224.

**Gallic acid derivative (5a)**

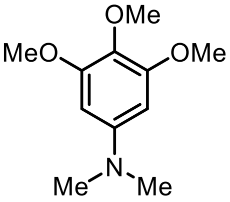


Following general procedure **(B)**, **5a** was obtained as white solid in 69% isolated yield from tert-butyl (4-bromophenethyl)carbamate using 10:1 petroleum ether/EtOAc as eluent.

**$^1$H NMR** (500 MHz, $CDCl_3$) δ 5.96 (s, 2H), 3.86 (s, 6H), 3.77 (s, 3H), 2.92 (s, 6H).

**$^{13}$C NMR** (126 MHz, $CDCl_3$) δ 153.73, 147.74, 130.07, 91.03, 61.12, 56.09, 41.26.

The NMR data were in consistent with the reported data.[57]

**Pirfenidone derivative (5b)**

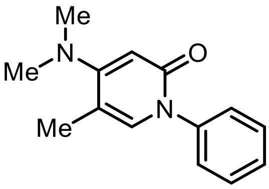


Following general procedure **(B)**, **5b** was obtained as yellowish-brown oil in 85% isolated yield from 4-bromo-5-methyl-1-phenylpyridin-2(1*H*)-one using 3:1 petroleum ether/EtOAc as eluent.

**$^1$H NMR** (500 MHz, $CDCl_3$) δ 7.44 (dd, $J$ = 8.6, 6.9 Hz, 2H), 7.39 – 7.32 (m, 3H), 6.77 – 6.72 (m, 1H), 6.48 (s, 1H), 2.88 (s, 6H), 2.07 (s, 3H).

**$^{13}$C NMR** (126 MHz, $CDCl_3$) δ 158.34, 143.14, 141.81, 129.16, 128.01, 126.86, 126.53, 121.79, 114.65, 41.78, 17.75.

**IR (ART):** ν = 3041.89, 2944.72, 2860.87, 2781.83, 2230.65, 1658.33, 1606.64, 1587.06, 1544.18, 1490.90, 1454.63, 1402.65, 1266.30, 1203.74, 1141.45, 1100.44, 1049.66, 1010.87, 911.15, 894.76, 816.84, 779.02, 756.42, 726.67, 694.15, 655.62, 632.86, 578.76, 532.15, 500.90, 478.19 $cm^{-1}$.

**HRMS (ESI-TOF) m/z**: $[M+K]^+$ Calcd. for $C_{14}H_{16}N_2O$ 267.0894; Found 267.0894

**Thymol derivative (5c)**

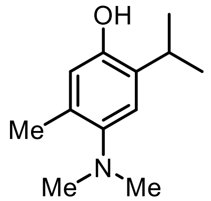

Following general procedure **(B)**, **5c** was obtained as white solid in 40% isolated yield from 4-bromo-2-isopropyl-5-methylphenol using 10:1 petroleum ether/EtOAc as eluent.

**$^1$H NMR** (500 MHz, $CDCl_3$) δ 6.92 (s, 1H), 6.56 (s, 1H), 4.76 (s, 1H), 3.17 (p, *J* = 6.9 Hz, 1H), 2.66 (s, 6H), 2.24 (s, 3H), 1.26 (d, *J* = 6.9 Hz, 6H).

**$^{13}$C NMR** (126 MHz, $CDCl_3$) δ 148.45, 146.19, 132.07, 131.15, 117.96, 116.87, 45.08, 27.44, 22.85, 17.66.

**IR (ATR):** ν = 2958.34, 2869.13, 2775.63, 1719.55, 1612.58, 1511.23, 1452.15, 1410.33, 1339.37, 1245.70, 1168.08, 1133.99, 1095.21, 1034.68, 1003.55, 926.24, 879.40, 855.96, 780.97, 740.00, 683.32, 644.48, 601.21, 539.43, 494.08, 476.73, 448.29 $cm^{-1}$.

**HRMS (ESI-TOF) m/z**: $[M+H]^+$ Calcd. for $C_{12}H_{19}NO$ 194,1539; Found 194.1532.

**Thymol derivative (5d)**

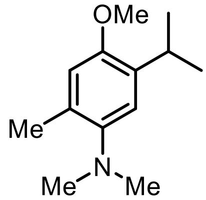


Following general procedure **(B)**, **5d** was obtained as colorless transparent liquid in 62% isolated yield from 1-bromo-5-isopropyl-4-methoxy-2-methylbenzene using 40:1 petroleum ether/EtOAc as eluent.

**$^1$H NMR** (500 MHz, $CDCl_3$) δ 6.98 (s, 1H), 6.71 (s, 1H), 3.82 (s, 3H), 3.29 (h, *J* = 6.9 Hz, 1H), 2.69 (s, 6H), 2.34 (s, 3H), 1.24 (d, *J* = 7.0 Hz, 6H).

**$^{13}$C NMR** (126 MHz, $CDCl_3$) δ 152.62, 145.83, 134.85, 130.66, 116.83, 113.56, 55.85, 45.05, 27.06, 22.99, 18.05.

**IR (ATR): ν =** 2933.83, 2866.64, 2823.31, 2775.08, 1504.13, 1451.90, 1396.39, 1342.08, 1242.75, 1172.66, 1149.13, 1095.20, 1064.31, 1010.08, 927.52, 879.69, 842.59, 805.29, 779.19, 740.08, 638.85, 609.55 $cm^{-1}$.

**HRMS (ESI-TOF) m/z**: $[M+H]^+$ Calcd. for $C_{13}H_{21}NO$ 208.1696; Found 208.1697.

**Estradiol derivative (5e)**

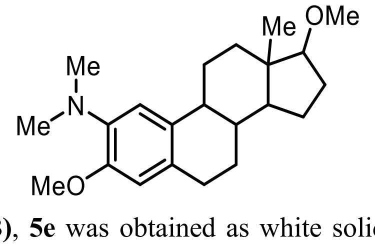


Following general procedure **(B)**, **5e** was obtained as white solid in 52% isolated yield from 2-bromo-3,17-dimethoxy-13-methyl-7,8,9,11,12,13,14,15,16,17-decahydro-6*H*-cyclopenta[a]phenanthrene using 10:1 petroleum ether/EtOAc as eluent.

**Melting point:** 139.92-140.13 °C

**$^1$H NMR** (500 MHz, $CDCl_3$) δ 6.90 (s, 1H), 6.57 (s, 1H), 3.85 (s, 3H), 3.38 (s, 3H), 3.32 (t, *J* = 8.3 Hz, 1H), 2.81 (d, *J* = 2.2 Hz, 2H), 2.76 (s, 6H), 2.34 – 2.26 (m, 1H), 2.21 (ddd, *J* = 14.8, 10.9, 4.0 Hz, 1H), 2.13 – 2.00 (m, 2H), 1.88 (ddt, *J* = 12.4, 5.9, 2.1 Hz, 1H), 1.77 – 1.64 (m, 1H), 1.58 – 1.23

(m, 7H), 0.80 (s, 3H).

**$^{13}$C NMR** (126 MHz, $CDCl_3$ δ 150.57, 140.24, 132.13, 130.76, 115.53, 111.50, 90.91, 57.98, 55.41, 50.40, 44.36, 43.75, 43.36, 38.79, 38.23, 29.51, 27.88, 27.48, 26.70, 23.16, 11.70.

**IR (neat)** : ν = 3441.6, 2953.5, 2933.8, 2872.1,2832.5, 2778.5, 1607.4, 1511.3, 1459.5, 1336.3, 1299.3, 1252.4, 1232.7, 1134.1, 1114.4, 1099.6, 1028.1, 992.68, 917.17, 870.33, 798.84, 739.67. $cm^{-1}$

**HRMS (ESI-TOF) m/z**: $[M+H]^+$ Calcd. for $C_{22}H_{33}NO_2$ 344.2584; Found 344.2579.

**Neostigmine derivative (5f)**

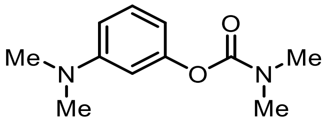


Following general procedure **(B)**, **5f** was obtained as light yellow oil in 80% isolated yield from 3-bromophenyl dimethylcarbamate using 10:1 petroleum ether/EtOAc as eluent.

**$^1$H NMR** (500 MHz, $CDCl_3$) δ 7.19 (dd, $J$ = 9.0, 7.4 Hz, 1H), 6.58 – 6.53 (m, 1H), 6.46 (dd, $J$ = 8.7, 1.7 Hz, 2H), 3.09 (s, 3H), 3.01 (s, 3H), 2.94 (s, 6H).

**$^{13}$C NMR** (126 MHz, $CDCl_3$) δ 155.28, 152.69, 151.78, 129.53, 109.74, 109.62, 106.04, 40.60, 36.75, 36.55.The NMR data were in consistent with the reported data.[58]

**Cinromide derivative (5g)**

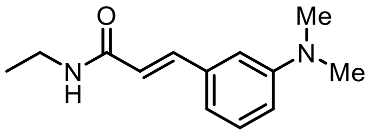


Following general procedure **(B)**, **5g** was obtained as yellow solid in 70% isolated yield from (E)-3-(3-bromophenyl)-*N*-ethylacrylamide using 2:1 petroleum ether/EtOAc as eluent.

**Melting point**: 150.08-150.38 °C

**$^1$H NMR** (500 MHz, $CDCl_3$) δ 7.58 (d, $J$ = 15.6 Hz, 1H), 7.18 (t, $J$ = 7.9 Hz, 1H), 6.87 (d, $J$ = 7.5 Hz, 1H), 6.79 (s, 1H), 6.70 (dd, $J$ = 8.3, 2.6 Hz, 1H), 6.43 (d, $J$ = 15.5 Hz, 1H), 6.18 (s, 1H), 3.46 – 3.35 (m, 2H), 2.92 (s, 6H), 1.19 (t, $J$ = 7.3 Hz, 3H).

**$^{13}$C NMR** (126 MHz, $CDCl_3$) δ 166.27, 150.81, 141.66, 135.67, 129.44, 120.63, 115.79, 113.86, 112.17, 40.56, 34.64, 14.93.

**IR (neat)**: ν = 3466.96, 3243.70, 3054.46, 2965.86, 2889.95, 1651.35, 1612.88, 1599.50, 1548.70, 1358.99, 997.01, 755.84 $cm^{-1}$.

**HRMS (ESI-TOF) m/z**: $[M+Na]^+$ Calcd. for $C_{13}H_{18}N_2O$ 241.1311, found: 241.1320.

**Levodropropizine derivative (5h)**

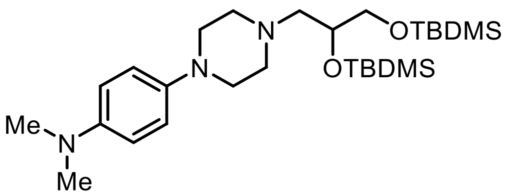


Following general procedure **(B)**, **5h** was obtained as colorless transparent oli in 69% isolated yield

from 1-(2,3-bis((*tert*-butyldimethylsilyl)oxy)propyl)-4-(4-bromophenyl)piperazine using 10:1 petroleum ether/EtOAc as eluent.

**$^{1}$H NMR** (500 MHz, $CDCl_3$) δ 6.91 (d, *J* = 9.1 Hz, 2H), 6.76 (d, *J* = 9.0 Hz, 2H), 3.81 (p, *J* = 5.5 Hz, 1H), 3.62 (dd, *J* = 10.0, 5.6 Hz, 1H), 3.53 (dd, *J* = 10.1, 5.6 Hz, 1H), 3.06 (t, *J* = 5.0 Hz, 4H), 2.87 (s, 6H), 2.65 (ddt, *J* = 32.5, 10.9, 4.9 Hz, 4H), 2.50 (dd, *J* = 13.0, 4.8 Hz, 1H), 2.38 (dd, *J* = 13.0, 6.1 Hz, 1H), 0.90 (d, *J* = 4.6 Hz, 18H), 0.11 – 0.03 (m, 12H).

**$^{13}$C NMR** (126 MHz, $CDCl_3$) δ 145.67, 143.71, 118.23, 114.70, 71.94, 66.39, 62.17, 54.55, 50.96, 41.74, 26.15, 26.07, 18.52, 18.34, -4.35, -5.12.

**IR (neat)**: v = 3436.46, 2952.07, 2928.15, 2883.37, 2855.72, 1519.94, 1471.83, 1460.57, 1322.58, 1296.98, 1255.94, 1248.79, 1234.97, 1143.99,1124.55, 1107.64, 723.15, 668.63 $cm^{-1}$.

**HRMS (ESI-TOF) m/z**: $[M+H]^+$ Calcd. for $C_{27}H_{53}N_3O_2Si_2$ 508.3749; Found 508.3749.

**Tyramine derivative (5i)**

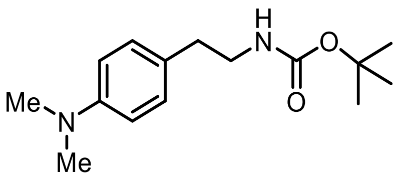


Following general procedure **(B)**, **5i** was obtained as white solid in 68% isolated yield from *tert*-butyl (4-bromophenethyl)carbamate using 10:1 petroleum ether/EtOAc as eluent.

**Melting point:** 69.35-69.44 ℃

**$^{1}$H NMR** (500 MHz, $CDCl_3$) δ 7.07 (d, *J* = 8.2 Hz, 2H), 6.72 (d, *J* = 8.2 Hz, 2H), 4.58 (s, 1H), 3.34 (q, *J* = 6.7 Hz, 2H), 2.93 (s, 6H), 2.70 (t, *J* = 7.1 Hz, 2H), 1.44 (s, 9H).

**$^{13}$C NMR** (126 MHz, $CDCl_3$) δ 156.02, 149.41, 129.52, 127.10, 113.18, 79.12, 42.11, 40.94, 35.18, 28.53.

**IR (neat):** v = 3851.69, 3747.73, 3358.21, 3008.76, 2080.78, 2932.23, 2861.15, 1686.13, 1618.76, 1537.53, 1522.20, 1365.27, 1351.82, 1305.65, 1268.55, 1250.78, 1180.63, 811.77 $cm^{-1}$.

**HRMS (ESI-TOF) m/z**: $[M+H]^+$ Calcd. for $C_{15}H_{24}N_2O_2$ 265.1911; Found 265.1917.

**Atomoxetine derivative (5j)**

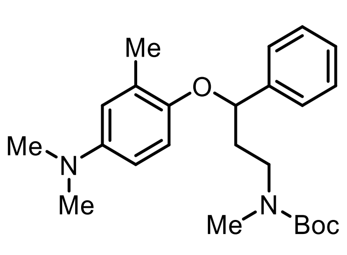


Following general procedure **(B)**, **5j** was obtained as colorless transparent oil in 75% isolated yield from *tert*-butyl (3-(4-bromo-2-methylphenoxy)-3-phenylpropyl)(methyl)carbamate using 6:1 petroleum ether/EtOAc as eluent.

**$^{1}$H NMR** (500 MHz, $CDCl_3$) δ 7.38 – 7.22 (m, 5H), 6.65 (d, *J* = 3.1 Hz, 1H), 6.50 (d, *J* = 8.9 Hz, 1H), 6.40 (dd, *J* = 8.8, 3.0 Hz, 1H), 5.04 (dd, *J* = 8.7, 4.2 Hz, 1H), 3.50 – 3.36 (m, 2H), 2.86 (s, 3H), 2.81 (s, 6H), 2.33 (s, 3H), 2.23 – 2.04 (m, 2H), 1.41 (s, 9H).

**$^{13}$C NMR** (126 MHz, $CDCl_3$) δ 155.83, 148.55, 145.28, 142.36, 128.64, 127.48, 125.87, 125.85, 117.03, 113.81, 111.63, 79.35, 46.15, 41.84, 37.30, 34.55, 28.48, 17.05.

**IR (ATR):** ν = 2975.69, 2244.93, 1682.68, 1503.68, 1450.43, 1394.48, 1365.28, 1303.15, 1234.75, 1171.09, 1142.87, 1050.98, 1009.33, 955.72, 910.61, 879.95, 795.39, 729.92, 701.53, 647.76, 536.43, 444.32, 416.87 $cm^{-1}$.

**HRMS (ESI-TOF) m/z**: $[M+H]^+$ Calcd. for $C_{24}H_{34}N_2O_3$ 399.2642; Found 399.2646.

**Gemfibrozil derivative (5k)**

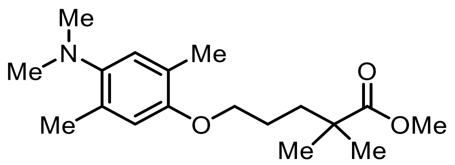


Bromo-gefibrozil methyl ester was synthesized according to literature precedent.[59] This product was obtained as transparent colorless liquid. The NMR data were in consistent with the reported data.[59] Following general procedure **(B)**, **5k** was obtained as light yellow oli in 67% isolated yield from methyl 5-(4-bromo-2,5-dimethylphenoxy)-2,2-dimethylpentanoate using 40:1 petroleum ether/EtOAc as eluent.

**$^1$H NMR** (500 MHz, $CDCl_3$) δ 6.89 (s, 1H), 6.65 (s, 1H), 3.91 (t, *J* = 3.0 Hz, 2H), 3.69 (s, 3H), 2.66 (s, 6H), 2.32 (s, 3H), 2.22 (s, 3H), 1.73 (d, *J* = 3.1 Hz, 4H), 1.25 (s, 6H).

**$^{13}$C NMR** (126 MHz, $CDCl_3$) δ 178.41, 152.95, 130.71, 124.43, 121.35, 114.25, 68.52, 51.80, 45.05, 42.21, 37.26, 25.41, 25.29, 18.04, 16.03.

**IR (ATR):** ν = 2931.64, 2862.68, 2776.53, 1729.07, 1607.61, 1511.10, 1473.20, 1450.90, 1388.95, 1365.83, 1319.97, 1248.02, 1195.59, 1172.95, 1143.25, 1089.29, 1043.02, 993.78, 940.91, 872.07, 823.53, 771.60, 663.76, 594.05, 470.48 $cm^{-1}$.

**HRMS (ESI-TOF) m/z**: $[M+H]^+$ Calcd. for $C_{18}H_{29}NO_3$ 308.2220; Found 308.2229.

**Canagliflozin intermediate derivative (5l)**

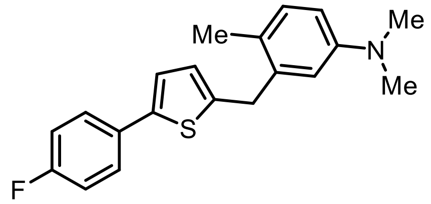


Following general procedure **(B)**, **5l** was obtained as off-white solid in 81% isolated yield from 2-(5-bromo-2-methylbenzyl)-5-(4-fluorophenyl)thiophene using 80:1 petroleum ether/EtOAc as eluent.

**Melting point:** 87.01-87.27 °C

**$^1$H NMR** (500 MHz, $CDCl_3$) δ 7.53 – 7.43 (m, 2H), 7.09 – 6.98 (m, 4H), 6.68 (dd, *J* = 8.8, 3.0 Hz, 2H), 6.62 (dd, *J* = 8.3, 2.5 Hz, 1H), 4.09 (s, 2H), 2.92 (s, 6H), 2.23 (s, 3H).

**$^{13}$C NMR** (126 MHz, $CDCl_3$) δ 162.17 (d, *J* = 246.5 Hz), 149.53, 144.15, 141.44, 138.69, 131.17, 131.12 (d, *J* = 3.3 Hz), 127.22 (d, *J* = 7.9 Hz), 125.87, 124.65, 122.74, 115.80 (d, *J* = 21.8 Hz), 114.72, 111.84, 41.10, 34.84, 18.44.

**$^{19}$F NMR** (471 MHz, $CDCl_3$) δ -115.37.

**IR (neat):** ν = 3431.73, 3077.17, 3036.35, 2913.45, 2805.09, 1617.3, 1508.8, 1449.7, 1356, 1230.3, 1158.8, 1112, 1094.7, 855.54, 828.42, 796.37, 710.09, 675.57 cm$^{-1}$.

**HRMS (ESI-TOF) m/z**: $[M+H]^+$ Calcd. for $C_{20}H_{20}FNS$ 326.1373; Found 326.1381.

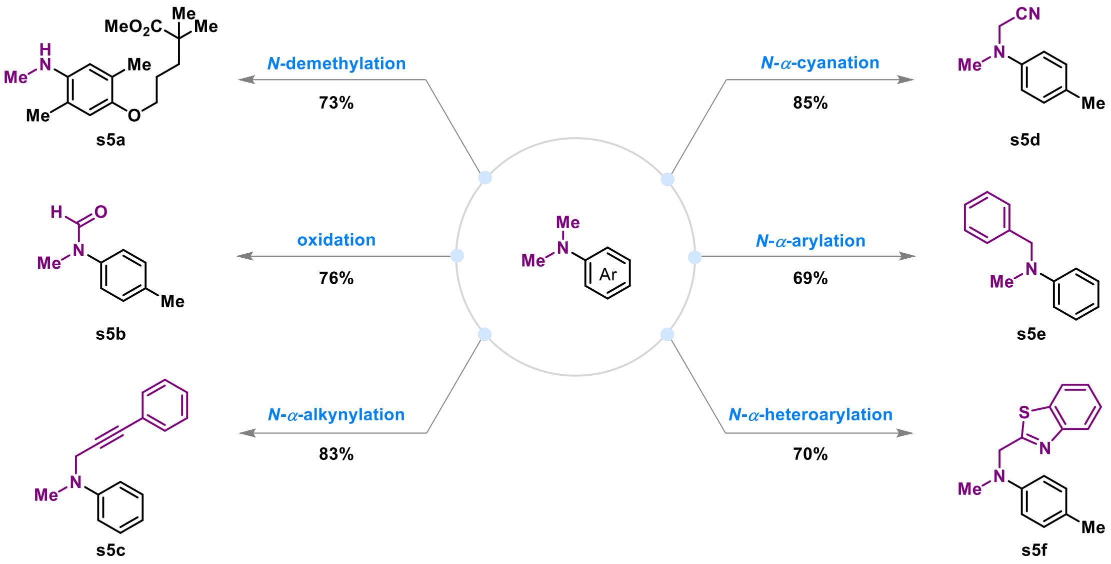


**Figure S5.** Various synthetic transformations of *N,N*-dimethyl derivatives

**Methyl 5-(2,5-dimethyl-4-(methylamino)phenoxy)-2,2-dimethylpentanoate (s5a)**

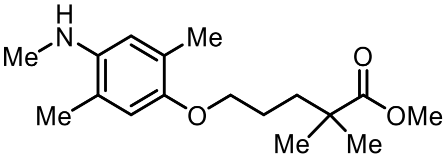

methyl 5-(2,5-dimethyl-4-(methylamino)phenoxy)-2,2-dimethylpentanoate **(s5a)** (42.84 mg, 73% isolated yield) was synthesized according to literature precedent:[60] Into a 4 mL vial containing a magnetic stirring bar were added aequentially methyl 5-(4-(dimethylamino)-2,5-dimethylphenoxy)-2,2-dimethylpentanoate (0.2 mmol, 1.0 equiv.), $Na_2HPO_4$ (0.4 mmol, 2.0 equiv.) and PIDA (0.3 mmol, 1.5 equiv.). And then dry DMSO (2.0 mL, 0.1 M) was added to dissolve the substrate in the glove box filled with argon gas. The resulting mixture was stirred at room temperature under irradiation with a 40 W blue LEDs light (Kessil PR160L-427 nm lamp) for 24 h. The mixture was extracted three times with ethyl acetate. The combined organic layers were washed with brine and dried over anhydrous $Na_2SO_4$, filtered and concentrated in vacuo. The desired demethylated products were purified by flash chromatography on silica gel using 15:1 petroleum ether/EtOAc as eluent. This product was obtained as transparent colorless oil.

**$^1$H NMR** (500 MHz, $CDCl_3$) δ 6.61 (s, 1H), 6.44 (s, 1H), 3.91 – 3.80 (m, 2H), 3.66 (s, 3H), 2.85 (s, 3H), 2.21 (s, 3H), 2.10 (s, 3H), 1.70 (s, 4H), 1.21 (s, 6H).

**$^{13}$C NMR** (126 MHz, $CDCl_3$) δ 178.40, 149.01, 141.27, 125.38, 120.24, 115.80, 112.66, 69.68, 51.72, 42.12, 37.19, 31.57, 25.45, 25.19, 17.32, 16.09.

**IR (ATR):** ν = 3428.07, 2924.06, 2854.87, 1727.51, 1518.46, 1469.94, 1403.46, 1388.61, 1318.33, 1217.82, 1195.50, 1142.74, 1056.35, 988.05, 855.02, 771.58, 646.81, 447.66, 416.66 cm$^{-1}$.

**HRMS (ESI-TOF) m/z**: $[M+H]^+$ Calcd. for $C_{17}H_{27}NO_3$ 294.2064; Found 294.2060.

**$N$-methyl-$N$-($p$-tolyl)formamide (s5b)**

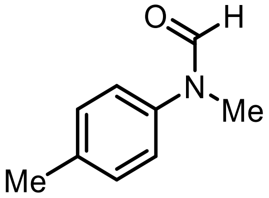

$N$-methyl-$N$-($p$-tolyl)formamide **(s5b)** (34.02 mg, 76% isolated yield) was synthesized according to literature precedent:[61] An oven-dried 4 mL vial equipped with a magnetic stirrer bar was charged with *N,N*-dimethylaniline (36.35 mg, 0.30 mmol) and DME (2.0 mL). The reaction vessel was exposed to LED (380-385 nm, 1.5 W) irradiation at room temperature in air with stirring for 24 h. After completion of the reaction, the mixture was concentrated to yield the crude product, the desired products were purified by flash chromatography on silica gel using 5:1 petroleum ether/EtOAc as eluent. This product was obtained as colorless liquid.

**$^1$H NMR** (500 MHz, $CDCl_3$) δ 8.41 (s, 1H), 7.20 (d, $J$ = 7.9 Hz, 2H), 7.05 (d, $J$ = 8.4 Hz, 2H), 3.28 (s, 3H), 2.35 (s, 3H). The NMR data were in consistent with the reported data.[61]

**$N$-methyl-$N$-(3-phenylprop-2-yn-1-yl)aniline (s5c)**

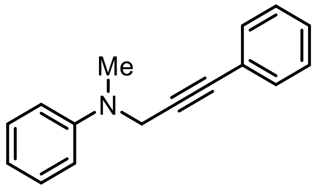

$N$-methyl-$N$-(3-phenylprop-2-yn-1-yl)aniline **(s5c)** (36.74mg, 83% isolated yield) was synthesized according to literature precedent:[62] An oven-dried 4 mL vial was charged with a magnetic stir bar, CuBr (1.43 mg, 0.01 mmol), *N,N*-dimethylaniline (0.051 mL, 0.4 mmol) and phenylacetylene (0.022 mL, 0.2 mmol) in the glove box filled with argon gas. Then added *tert*-butyl hydroperoxide (0.04 mL, 5-6M in decane) into the vial over 30 seconds at room temperature. The vial was sealed with a plastic cap and removed from the glove box. And then stirred for 3 h at 100 °C on magnetic stir bars. After cooling to room temperature, the reaction was quenched with $H_2O$ and diluted with EtOAc. The resulting mixture was separated and extracted with ethyl acetate (three times). The combined organic layer was dried over anhydrous $Na_2SO_4$, filtered and concentrated *in vacuo*. The pure product was obtained by flash column chromatography on silica gel using petroleum ether as eluent. This product was obtained as light yellow liquid.

**$^1$H NMR** (500 MHz, $CDCl_3$) δ 7.29 (dd, $J$ = 6.9, 2.9 Hz, 2H), 7.24 – 7.15 (m, 5H), 6.84 (d, $J$ = 8.2 Hz, 2H), 6.74 (t, $J$ = 7.3 Hz, 1H), 4.19 (s, 2H), 2.96 (s, 3H).

**$^{13}$C NMR** (126 MHz, $CDCl_3$) δ 148.30, 130.72, 128.06, 127.16, 127.05, 122.00, 117.13, 113.36, 83.95, 83.12, 42.29, 37.67. The NMR data were in consistent with the reported data.[62]

**2-(methyl($p$-tolyl)amino)acetonitrile (s5d)**

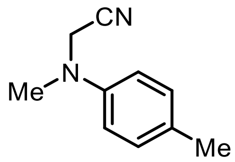


2-(methyl(*p*-tolyl)amino)acetonitrile **(s5d)** (136.08 mg, 85% isolated yield） was synthesized according to literature precedent:[63] An oven-dried 4 mL vial was charged with a magnetic stir bar, the iron(II) chloride (12.68 mg), tertiary amine (1.0 mmol), trimethylsilyl cyanide (2.0 mmol, 0.27 mL), and MeOH (2.0 mL) were added successively in the glove box filled with argon gas. To the mixture was added dropwise *tert*-butyl hydroperoxide (2.5 mmol, 0.470 mL, 5.5 M solution in decane) over a period of 5 min. The mixture was stirred at room temperature for the 10 hours. At the end of the reaction, the reaction mixture was poured into a saturated aqueous NaCl solution (20 mL) and extracted with $CH_2Cl_2$ (3 × 20 mL). The organic phases were combined, and the volatile components were evaporated in a rotary evaporator. The pure product was obtained by flash column chromatography on silica gel using 20:1 petroleum ether/EtOAc as eluent. This product was obtained as transparent colorless liquid.

**$^1$H NMR** (500 MHz, $CDCl_3$) δ 7.15 (d, *J* = 8.2 Hz, 2H), 6.82 (d, *J* = 8.6 Hz, 2H), 4.12 (s, 2H), 2.97 (s, 3H), 2.32 (s, 3H).

**$^{13}$C NMR** (126 MHz, $CDCl_3$) δ 144.69, 128.94, 128.74, 114.53, 114.35, 41.70, 38.38, 19.34.The NMR data were in consistent with the reported data.[63]

**N-benzyl-N-methylaniline (s5e)**

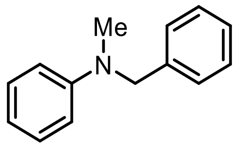


*N*-benzyl-*N*-methylaniline **(s5e)** (68.06 mg, 69% isolated yield) was synthesized according to literature precedent:[64] An oven-dried 10 mL round-bottom flask was charged with a magnetic stir bar, $Ni(acac)_2$ (12.8 mg, 0.05 mmol), $PPh_3$ (13.1 mg, 0.05 mmol), $K_3PO_4$ (106.2 mg, 0.5 mmol) $PhB(OH)_2$ (0.5 mmol) and THF (3 mL) in the glove box filled with argon gas. After stirring for 5 min, DTBP (0.6 mmol) was injected into the round-bottom flask. The reaction was then heated up to 100 °C and kept stirring for 16 hours. After completion of the reaction, the mixture was quenched with diluted hydrochloric acid. The solution was extracted with ethyl acetate (3 × 5 mL). The organic layers were combined and dried over anhydrous $Na_2SO_4$, The pure product was obtained by flash column chromatography on silica gel using 100:1 petroleum ether/EtOAc as eluent. This product was obtained as transparent colorless liquid.

**$^1$H NMR** (500 MHz, $CDCl_3$) δ 7.22 (d, *J* = 7.5 Hz, 2H), 7.19 – 7.09 (m, 5H), 6.65 (dd, *J* = 17.1, 7.7 Hz, 3H), 4.44 (s, 2H), 2.92 (d, *J* = 1.7 Hz, 3H). The NMR data were in consistent with the reported data.[64]

**N-(benzo[d]thiazol-2-ylmethyl)-N,4-dimethylaniline (s5f)**

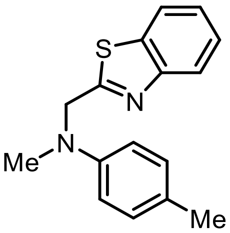


*N*-(benzo[d]thiazol-2-ylmethyl)-*N*,4-dimethylaniline **(s5f)** (37.53mg, 70% isolated yield) was synthesized according to literature precedent:[65] In a 4 ml vial were introduced the cobaloxime (0.8 mg, 2.0 μmol, 1 mol%), the iridium photocatalyst (2.2 mg, 2.0 μmol, 1 mol%) and DABCO (22.2 mg, 0.2 mmol, 1 equiv.). The vial was closed using a cap with a rubber septum and purged with argon for 15 minutes. DMF (2.0 mL), the heterocycle (0.2 mmol, 1.0 equiv.) and the amine (0.6 mmol, 3.0 equiv.) were subsequently added and the mixture was stirred under blue LEDs irradiation for 48 hours. After this time, The mixture was extracted three times with ethyl acetate. The combined organic layers were washed with brine and dried over anhydrous $Na_2SO_4$, filtered and concentrated in vacuo. The crude material was purified by column chromatography to afford the titled compounds.

**$^1$H NMR** (500 MHz, $CDCl_3$) δ 8.02 (d, *J* = 8.2 Hz, 1H), 7.81 (d, *J* = 8.1 Hz, 1H), 7.48 (t, *J* = 7.6 Hz, 1H), 7.36 (t, *J* = 7.5 Hz, 1H), 7.09 (d, *J* = 8.2 Hz, 2H), 6.79 (d, *J* = 8.6 Hz, 2H), 4.86 (s, 2H), 3.12 (s, 3H), 2.28 (s, 3H).

**$^{13}$C NMR** (126 MHz, $CDCl_3$) δ 172.21, 152.57, 145.74, 134.04, 128.79, 126.42, 124.87, 123.79, 121.63, 120.74, 112.40, 55.55, 38.52, 19.25. The NMR data were in consistent with the reported data.[65]

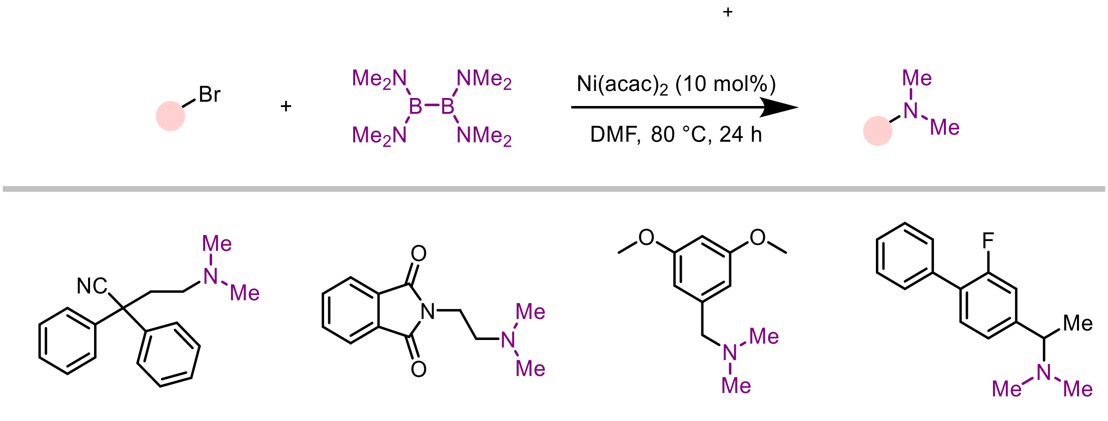


**s6a**, 43% **s6b**, 71% **s6c**, 50% **s6d**, 65%

**Figure S6.** Exploration of C-N bond formation with alkyl bromides

**4-(dimethylamino)-2,2-diphenylbutanenitrile (s6a)**

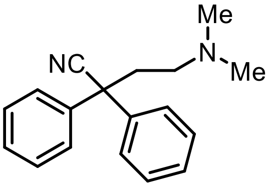


Following general procedure **(B)**, **s6a** was obtained as light yellow oil in 43% isolated yield from 4-bromo-2,2-diphenylbutanenitrile using 3:1 petroleum ether/EtOAc as eluent.

**$^1$H NMR** (400 MHz, $CDCl_3$) δ 7.52 – 7.27 (m, 10H), 2.67 – 2.56 (m, 2H), 2.49 – 2.36 (m, 2H), 2.25 (s, 6H).

**$^{13}$C NMR** (151 MHz, $CDCl_3$) δ 139.97, 128.99, 128.00, 126.76, 122.08, 55.83, 50.02, 45.57, 37.37. The NMR data were in consistent with the reported data.[66]

**1-(2-fluoro-[1,1'-biphenyl]-4-yl)-*N,N*-dimethylethan-1-amine (s6b)**

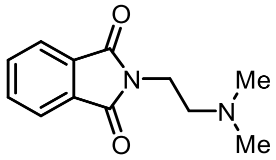


Following general procedure **(B)**, **s6b** was obtained as light yellow oil in 71% isolated yield from 2-(2-bromoethyl)isoindoline-1,3-dione using 3:1 petroleum ether/EtOAc as eluent.

**$^1$H NMR** (400 MHz, $CDCl_3$) δ 7.82 (dd, *J* = 5.5, 3.0 Hz, 2H), 7.69 (dd, *J* = 5.5, 3.0 Hz, 2H), 3.79 (t, *J* = 6.6 Hz, 2H), 2.58 (t, *J* = 6.6 Hz, 2H), 2.27 (d, *J* = 2.4 Hz, 6H).

**$^{13}$C NMR** (151 MHz, $CDCl_3$) δ 168.52, 133.95, 132.36, 123.33, 57.24, 45.59, 36.08. The NMR data were in consistent with the reported data.[67]

**1-(3,5-dimethoxyphenyl)-*N,N*-dimethylmethanamine (s6c)**

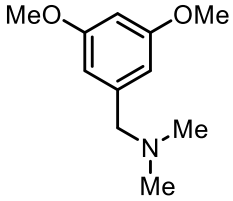


Following general procedure **(B)**, **s6c** was obtained as light yellow oil in 50% isolated yield from 1-(bromomethyl)-3,5-dimethoxybenzene using 3:1 petroleum ether/EtOAc as eluent.

**$^1$H NMR** (400 MHz, $CDCl_3$) δ 6.48 (d, *J* = 2.3 Hz, 2H), 6.35 (t, *J* = 2.3 Hz, 1H), 3.77 (s, 6H), 3.35 (s, 2H), 2.23 (s, 6H).

**$^{13}$C NMR** (101 MHz, $CDCl_3$) δ 160.73, 141.37, 106.77, 99.27, 64.62, 55.30, 45.44.The NMR data were in consistent with the reported data.[68]

**2-(2-(dimethylamino)ethyl)isoindoline-1,3-dione (s6d)**

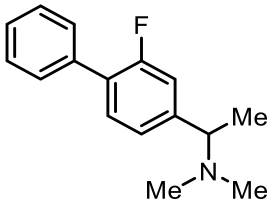


Following general procedure **(B)**, **s6d** was obtained as light yellow oil in 65% isolated yield from 4-(1-bromoethyl)-2-fluoro-1,1'-biphenyl using 3:1 petroleum ether/EtOAc as eluent.

**$^1$H NMR** (400 MHz, $CDCl_3$) δ 7.61 – 7.49 (m, 2H), 7.48 – 7.31 (m, 4H), 7.19 – 7.09 (m, 2H), 3.30 (d, *J* = 6.7 Hz, 1H), 2.24 (s, 6H), 1.39 (d, *J* = 6.6 Hz, 3H).

**$^{13}$C NMR** (151 MHz, $CDCl_3$) δ 159.85 (d, *J* = 247.8 Hz), 146.14 (d, *J* = 6.9 Hz), 135.93, 130.52 (d, *J* = 3.8 Hz), 129.10 (d, *J* = 2.9 Hz), 128.54, 127.63, 127.51, 123.55 (d, *J* = 3.2 Hz), 115.14 (d, *J* = 23.0 Hz), 65.38, 43.27, 20.07.

$^{19}$F NMR (471 MHz, $CDCl_3$) δ -118.40.The NMR data were in consistent with the reported data.[69]

# 4. Mechanistic Investigations

## 4.1 probing experiments and radical trapping experiments

### 4.1.1 probing experiments

**Table S2**. probing experiments

| Entry | Deviation from standard conditions | Yield (%)[a] |
|---|---|---|
| 1 | none | 94% |
| 2 | tris(dimethylamino)borane instead of $B_2(NMe_2)_4$ | 0% |
| 3 | Dimethylamine in THF instead of $B_2(NMe_2)_4$ | 0% |
| 5 | Dimethylamine in $H_2O$ instead of $B_2(NMe_2)_4$ | 0% |
| 6 | *N*,*N*-dimethylformamide instead of $B_2(NMe_2)_4$ | 0% |

[a]isolated yields.

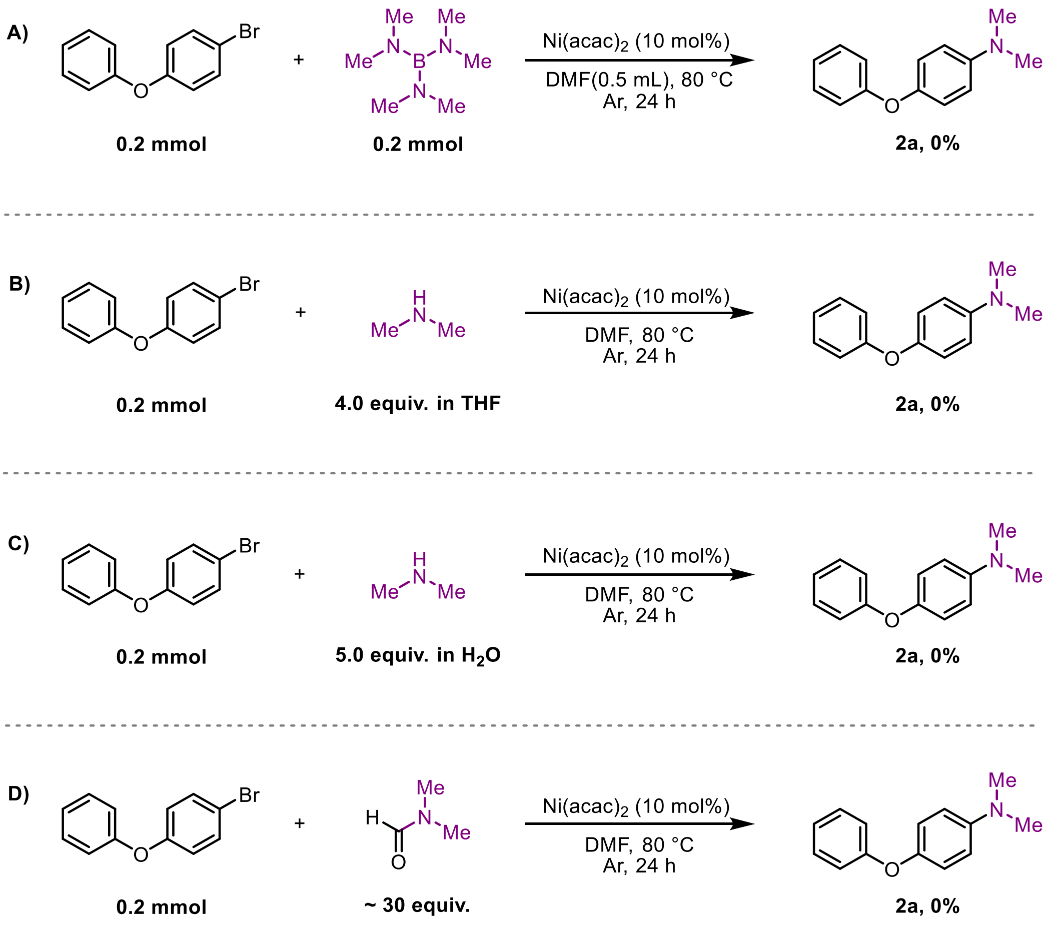


**Figure S7.** probing experiments

#### 4.1.2 radical trapping experiments

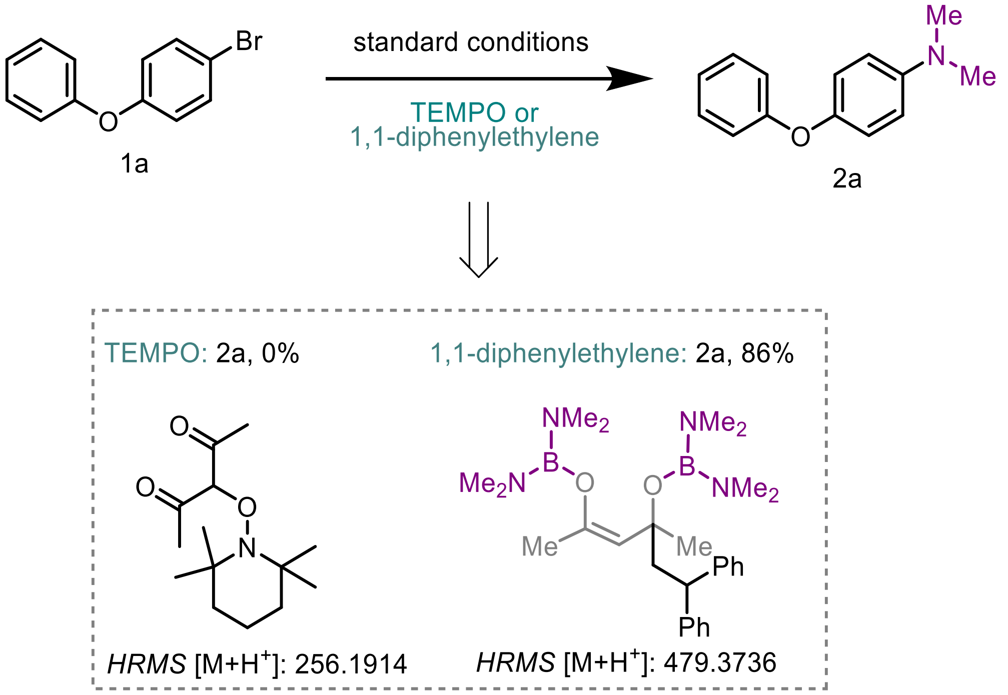


**Figure S8.** radical trapping experiments

**Procedure:**

An oven-dried 4 mL vial was charged with a magnetic stir bar, 1-bromo-4-phenoxybenzene (0.2 mmol, 1.0 equiv.), $Ni(acac)_2$ (0.02 mmol, 10 mol%), $B_2(NMe_2)_4$ (0.2 mmol, 1.0 equiv.), TEMPO (2.5 equiv.) or 1,1-diphenylethylene (2.5 equiv.), DMF (0.5 mL) in the glove box. The vial was sealed with a plastic cap and then stirring was achieved by placing the assembled reactor at 80 °C on IKA C-MAG HS 7 control magnetic stir bars for 24 h. Then use a micropipette to draw up 1 μL of the reaction solution and dilute it into 1 mL of MeCN for the detection of high-resolution data. The experimental results show that the C-N coupling reaction was completely suppressed using TEMPO as a radical inhibitor, and the adduct of TEMPO-acac was detected by HRMS (*HRMS* $[M+H^+]$: 256.1914). Additionally, no obvious inhibition of C-N bond formation using 1,1-diphenylethylene as a neutral radical scavenger, indicating that no radical species was involved in the Ni(I)/Ni(III) redox cycle. In this trapping experiment, radical intermediate was captured by 1,1-diphenylethylene (*HRMS* $[M+H^+]$: 479.3736).

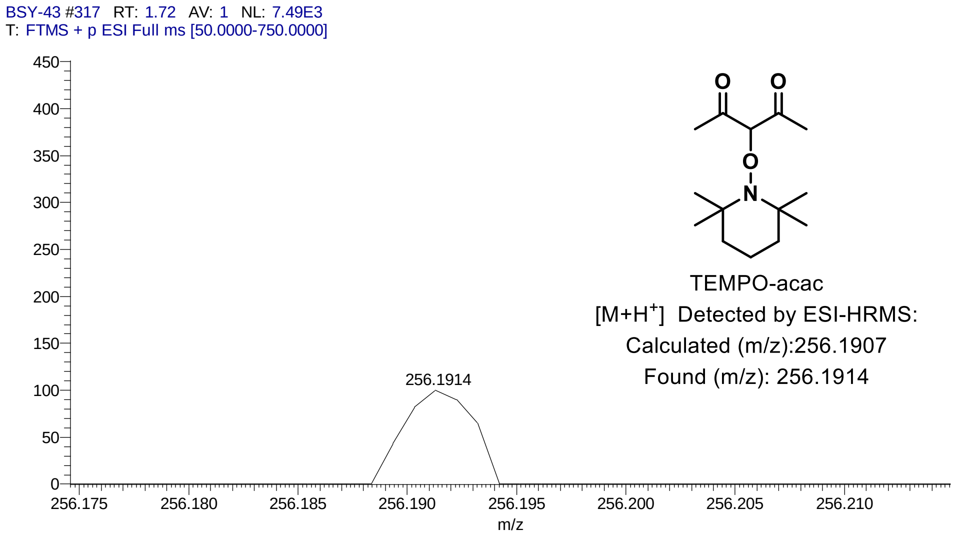


**Figure S9.** TEMPO adduct detected by HRMS

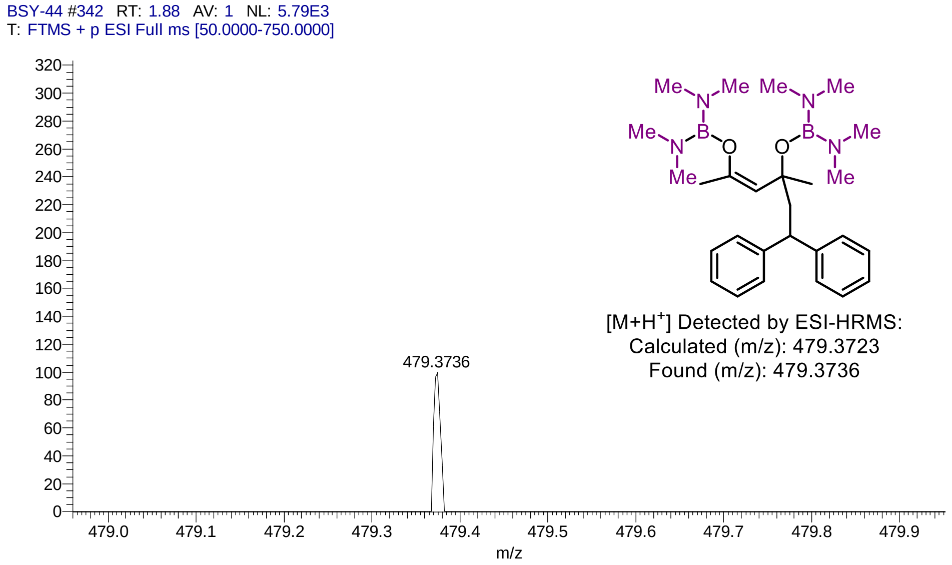


**Figure S10.** 1,1-diphenylethylene adduct detected by HRMS

## 4.2 Reaction of aryl bromide catalyzed by Ni(0)

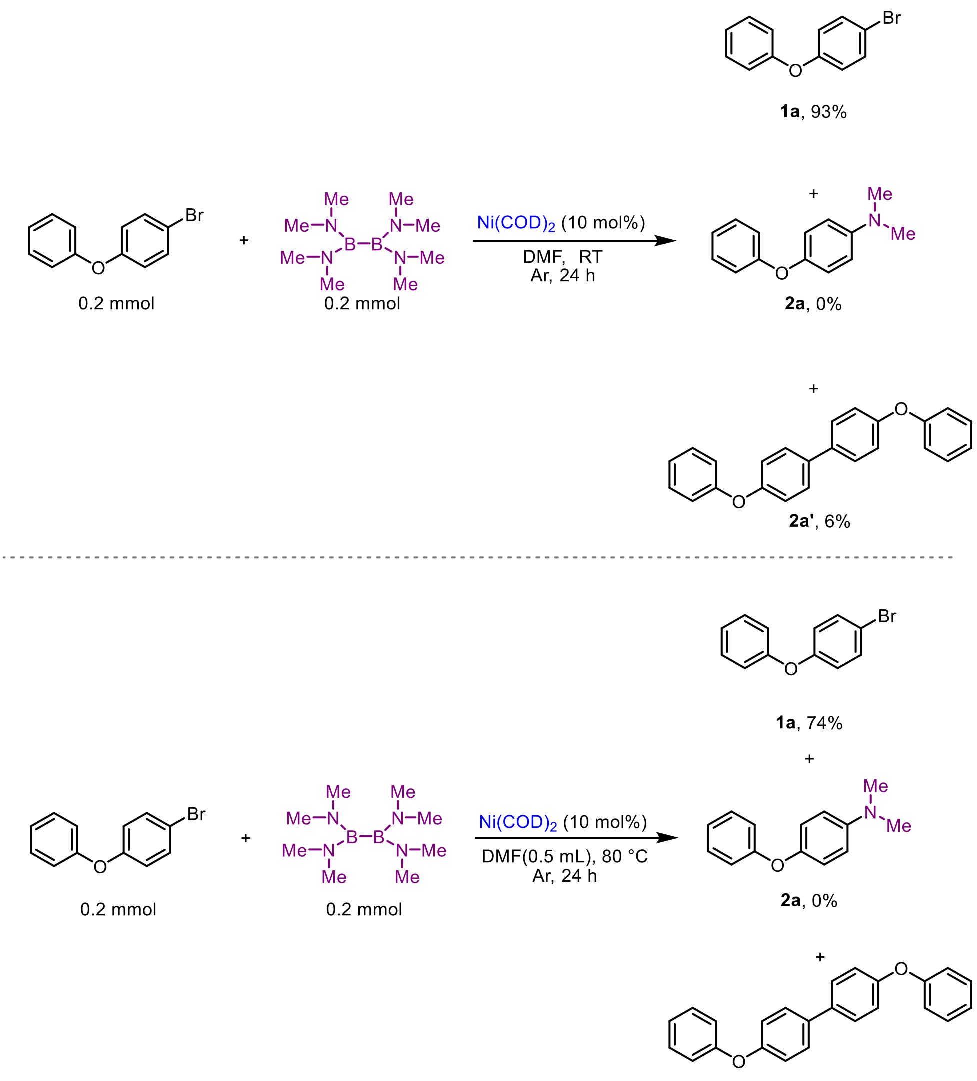


**Figure 11.** Reaction of aryl bromide catalyzed by Ni(0)

**Procedure :**

An oven-dried 4 mL vial was charged with a magnetic stir bar, aryl bromides (0.2 mmol, 1.0 equiv.), $Ni(COD)_2$ (0.02 mmol, 10 mol%), $B_2(NMe_2)_4$ (0.2 mmol, 1.0 equiv.), DMF (0.5 mL) in the glove box. The vial was sealed with a plastic cap and then stirring was achieved by placing the assembled reactor at RT or 80 °C on IKA C-MAG HS 7 control magnetic stir bars for 24 h. Then the reaction was quenched with $H_2O$ and diluted with EtOAc. The resulting mixture was separated and extracted with EtOAc (three times). The combined organic layer was dried over anhydrous $Na_2SO_4$, filtered and concentrated *in vacuo*. The reaction mixture was purified by fresh silica gel chromatography to afford the desired product. **$^1$H NMR** (500 MHz, $CDCl_3$) δ 7.53 (d, $J$ = 8.7 Hz, 4H), 7.36 (t, $J$ = 7.7 Hz, 4H), 7.12 (t, $J$ = 7.4 Hz, 2H), 7.09 – 6.97 (m, 8H). **$^{13}$C NMR** (126 MHz, $CDCl_3$) δ 157.31, 156.78, 135.80, 129.94, 128.32, 123.51, 119.24, 119.12.

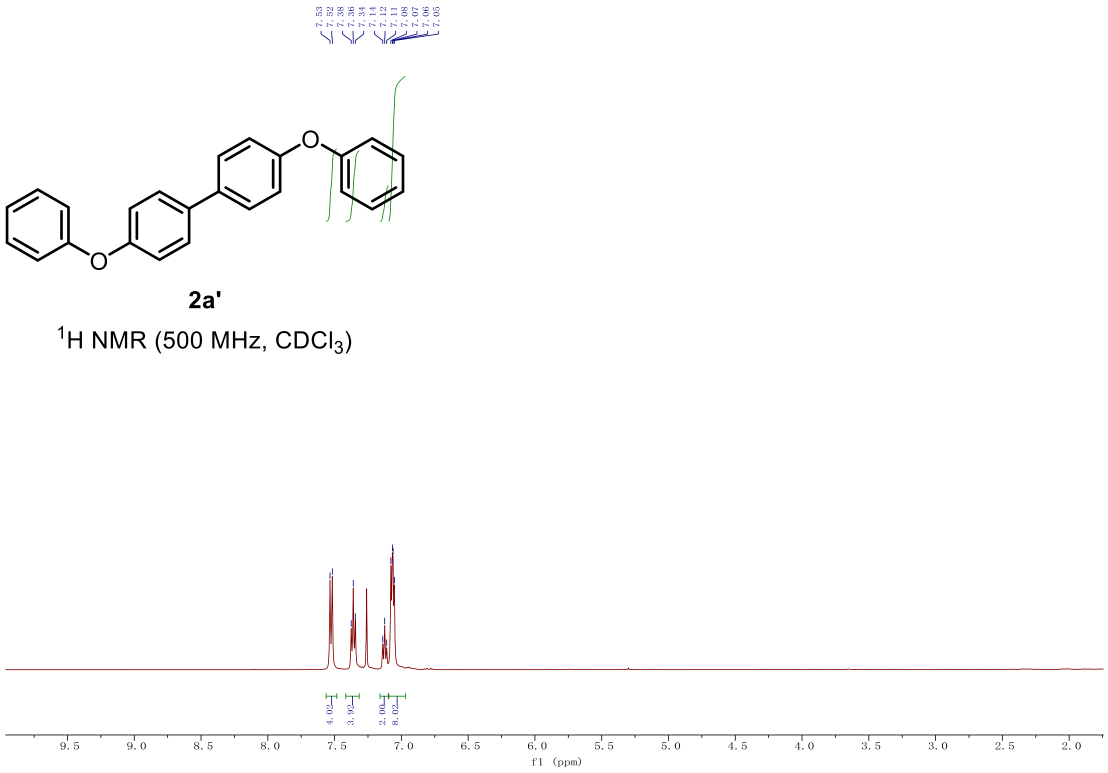


**Figure S12.** $^{1}$H NMR for homo-coupling product 2a’

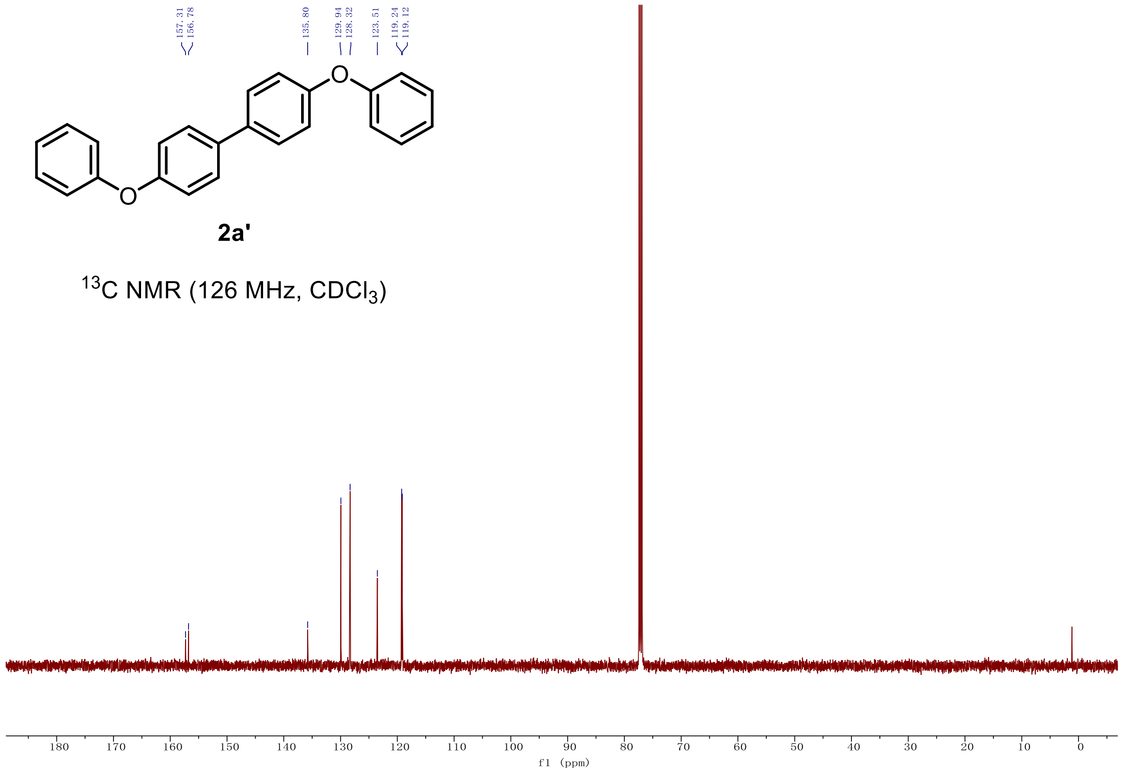


**Figure S13**. $^{13}$C NMR for homo-coupling product 2a’

## 4.3 Reaction of aryl bromide catalyzed by in-situ generated Ni(I)

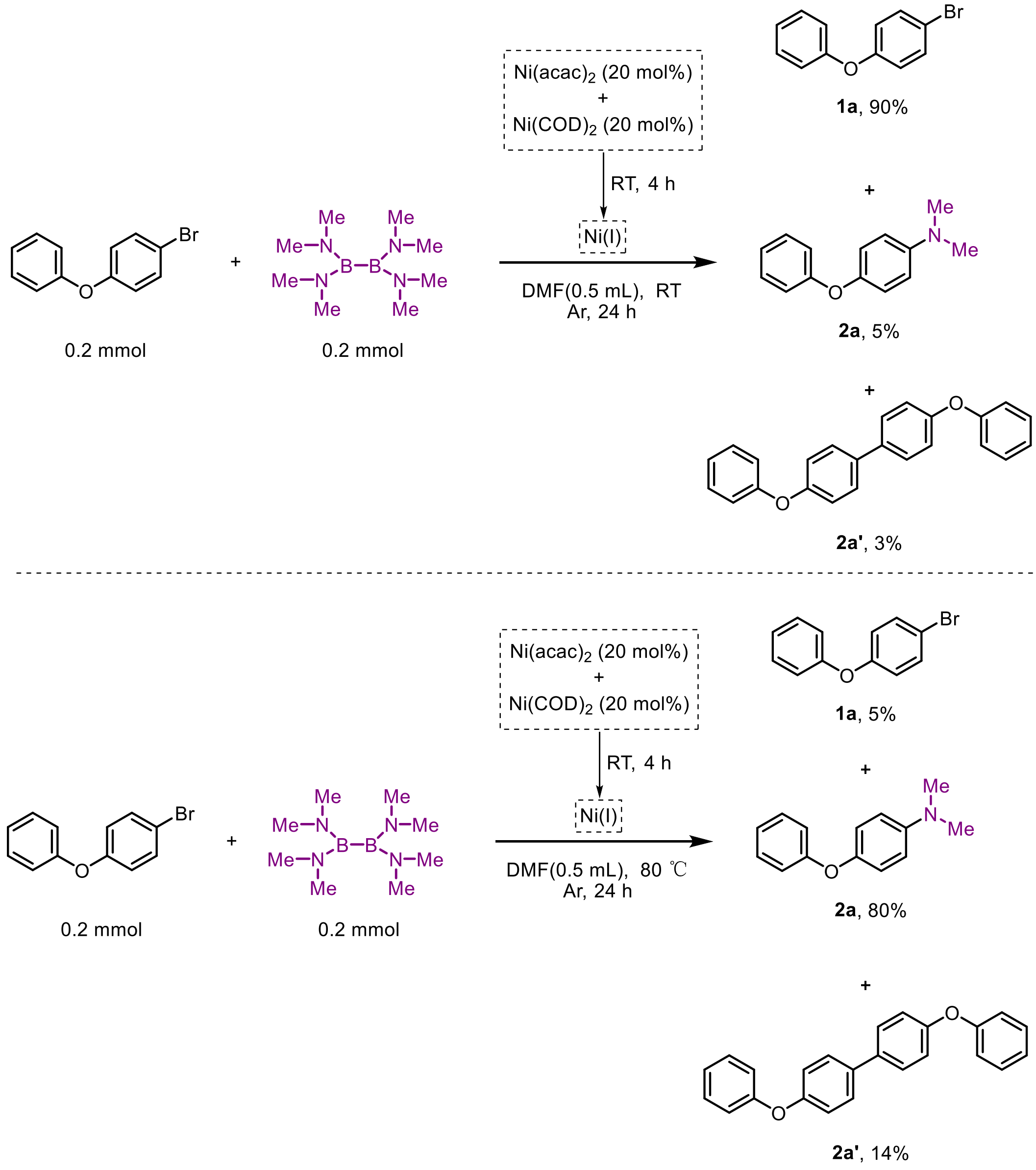


**Figure S14.** Reaction of aryl bromide catalyzed by in-situ generated Ni(I)

**Procedure :**

An oven-dried 4 mL vial was charged with a magnetic stir bar, $Ni(COD)_2$ (0.04 mmol, 20 mol%), $Ni(acac)_2$ (0.04 mmol, 20 mol%), DMF (0.5 mL) in glove box. The vial was sealed with a plastic cap and stirred at room temperature for 4 h and 1-bromo-4-phenoxybenzene (0.2 mmol, 1.0 equiv.), $B_2(NMe_2)_4$ (0.2 mmol, 1.0 equiv.) were added in succession. The vial was sealed with a plastic cap and removed from the glove box. And then stirred for 24 h at room temperature or 80 °C on IKA C-MAG HS 7 control magnetic stir bars. Then the reaction was quenched with $H_2O$ and diluted with EtOAc. The resulting mixture was separated and extracted with EtOAc (three times). The combined organic layer was dried over anhydrous $Na_2SO_4$, filtered and concentrated *in vacuo*. The residue was analyzed by $^1H$ NMR with $CDCl_3$ as a solvent to give the yield of the reaction. Yield was determined by $^1H$ NMR using pyrazine as the internal standard.

## 4.4 Reaction of aryl bromide catalyzed by Ni(II) at room temperature

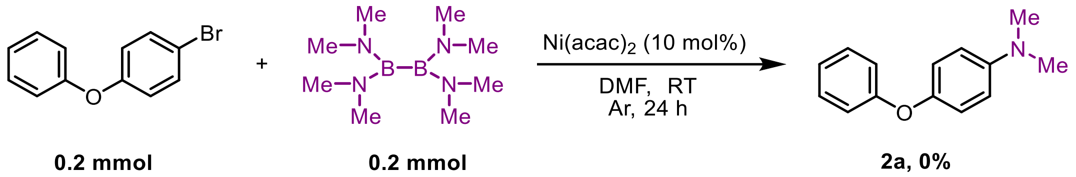


**Figure S15.** Reaction of aryl bromide catalyzed by Ni(II) at room temperature

**Procedure :**

An oven-dried 4 mL vial was charged with a magnetic stir bar, aryl bromides (0.2 mmol, 1.0 equiv.), $Ni(acac)_2$ (0.02 mmol, 10 mol%), $B_2(NMe_2)_4$ (0.2 mmol, 1.0 equiv.), DMF (0.5 mL) in the glove box. The vial was sealed with a plastic cap and then stirring was achieved by placing the assembled reactor at room temperature on IKA C-MAG HS 7 control magnetic stir bars for 24 h. Then the reaction was monitored by TLC, which showed no substrate consumption.

## 4.5 EPR study

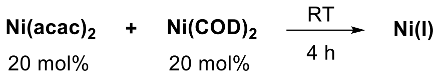


**Procedure:**

An oven-dried 4 mL vial was charged with a magnetic stir bar, $Ni(COD)_2$ (0.04 mmol, 20 mol%), $Ni(acac)_2$ (0.04 mmol, 20 mol%), DMF (0.5 mL) in glove box. The vial was sealed with a plastic cap and stirred at room temperature for 4 h. And the reaction mixture was transferred to an EPR tube. The EPR spectrum was collected on a frozen solution at 100 K.

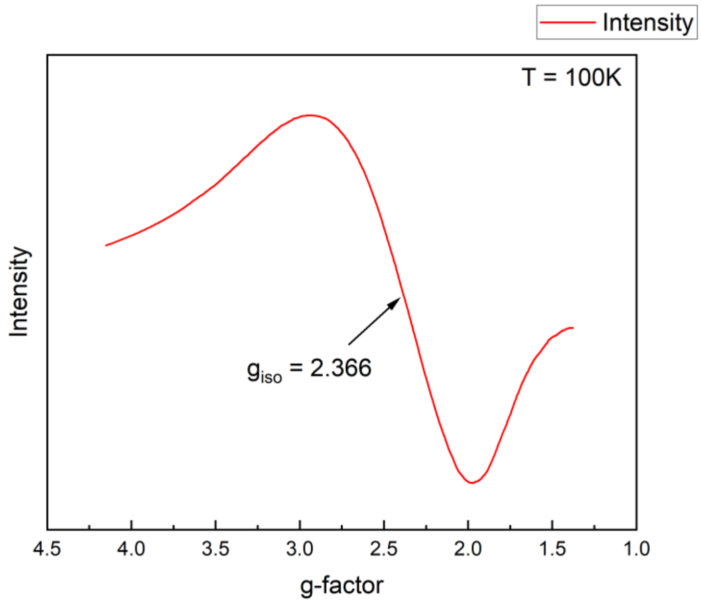


**Figure S16.** Ni(I) species was evidenced from the comproportionation reaction by EPR

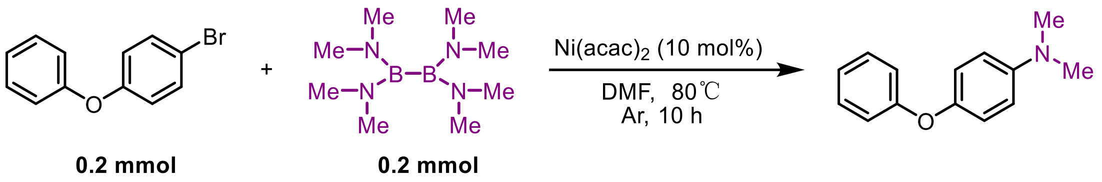


**Procedure:**

An oven-dried 4 mL vial was charged with a magnetic stir bar, aryl bromides (0.2 mmol, 1.0 equiv.), $Ni(acac)_2$ (0.02 mmol, 10 mol%), $B_2(NMe_2)_4$ (0.2 mmol, 1.0 equiv.), DMF (0.5 mL) in the glove box. The vial was sealed with a plastic cap and then stirring was achieved by placing the assembled reactor at 80 °C on IKA C-MAG HS 7 control magnetic stir bars for 10 h. And the reaction mixture was transferred to an EPR tube. The EPR spectrum was collected on a frozen solution at 100 K.

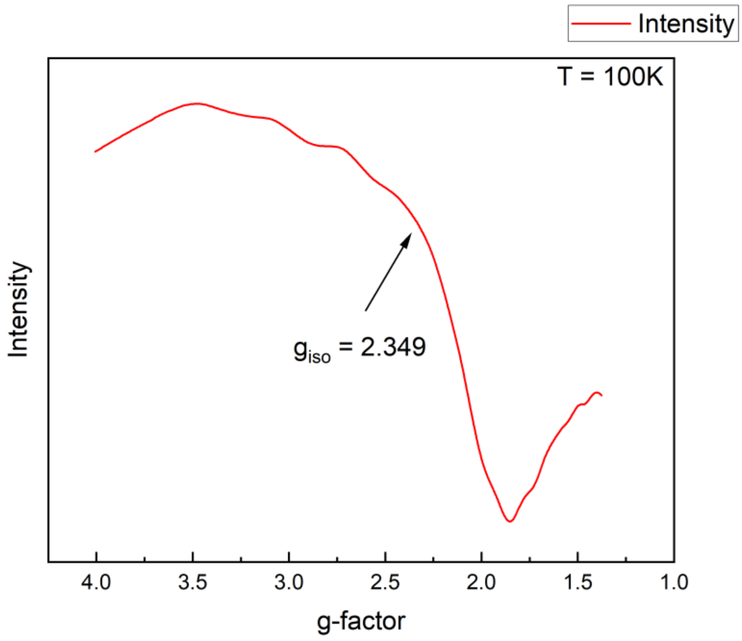


**Figure S17.** Ni(I) species was evidenced from standard reaction by EPR

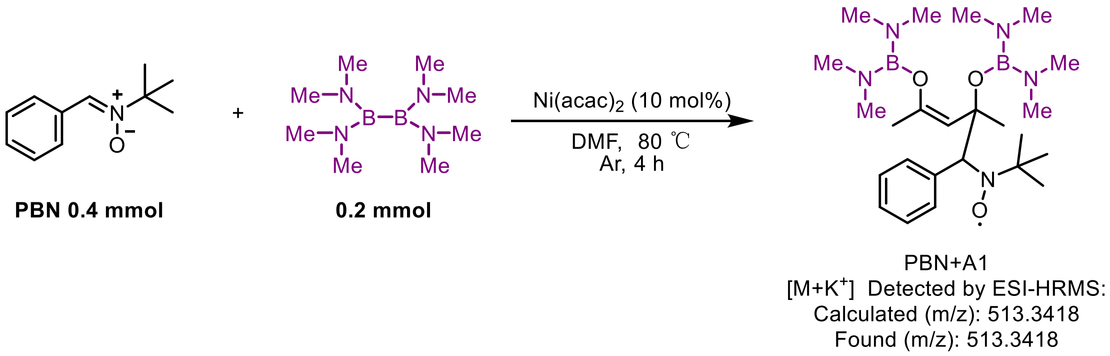


**Procedure:**

An oven-dried 4 mL vial was charged with a magnetic stir bar, aryl bromides (0.2 mmol, 1.0 equiv.), $Ni(acac)_2$ (0.02 mmol, 10 mol%), $B_2(NMe_2)_4$ (0.2 mmol, 1.0 equiv.), phenyl *tert*-butyl nitrone (PBN) (0.4 mmol), DMF (0.5 mL) in the glove box. The vial was sealed with a plastic cap and then stirring

was achieved by placing the assembled reactor at 80 °C on IKA C-MAG HS 7 control magnetic stir bars for 4 h. Then the reaction mixture was transferred to an EPR tube. The resulting mixture was analyzed by EPR at 80 °C. A g-factor in 2.005 indicated the existence of an un paired electron for organic radicals. this result strongly confirmed the radical nature of the reaction. And the adduct of PBN+A1 was detected by HRMS (*m/z*) ratio of 513.3418 (calc. 513.3418).

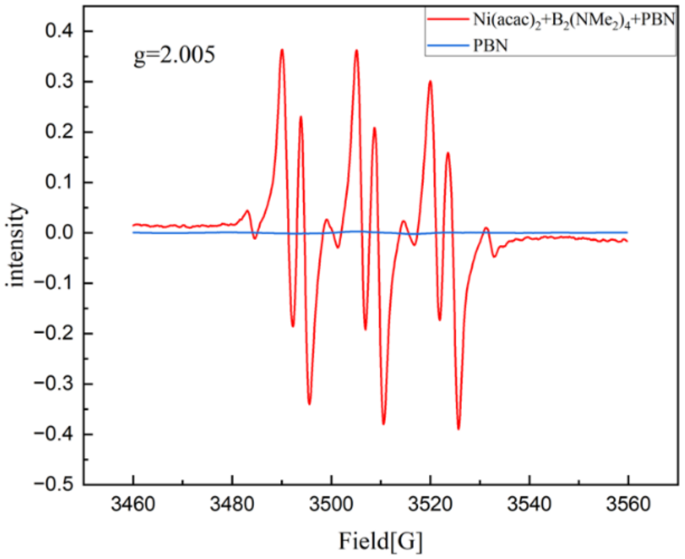


**Figure S18.** EPR experiment with phenyl *tert*-butyl nitrone (PBN) used as spin trapping reagent.

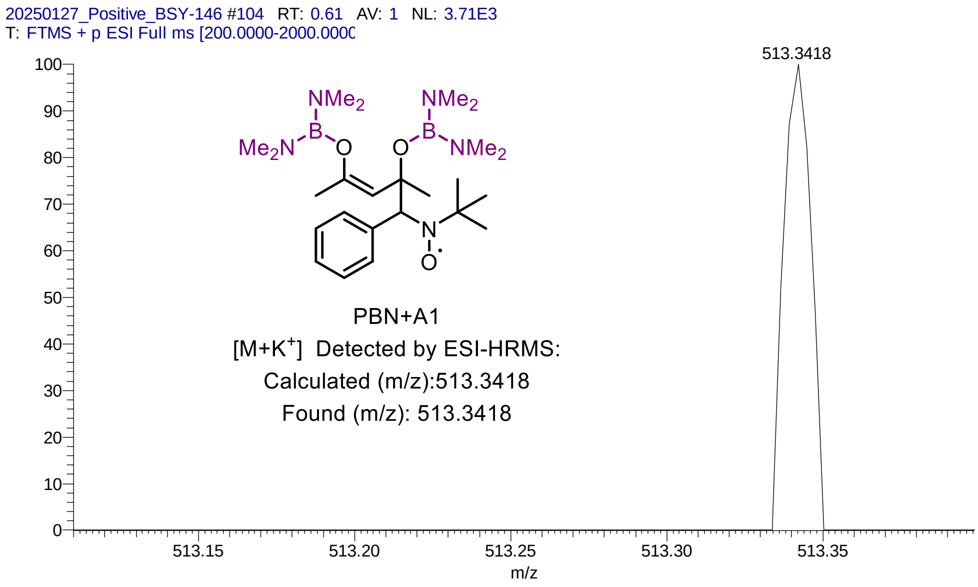


**Figure S19.** ESI-HRMS for PBN+A1

## 4.6 Detecting the generation of Ni(III) intermediates in solution by HRMS

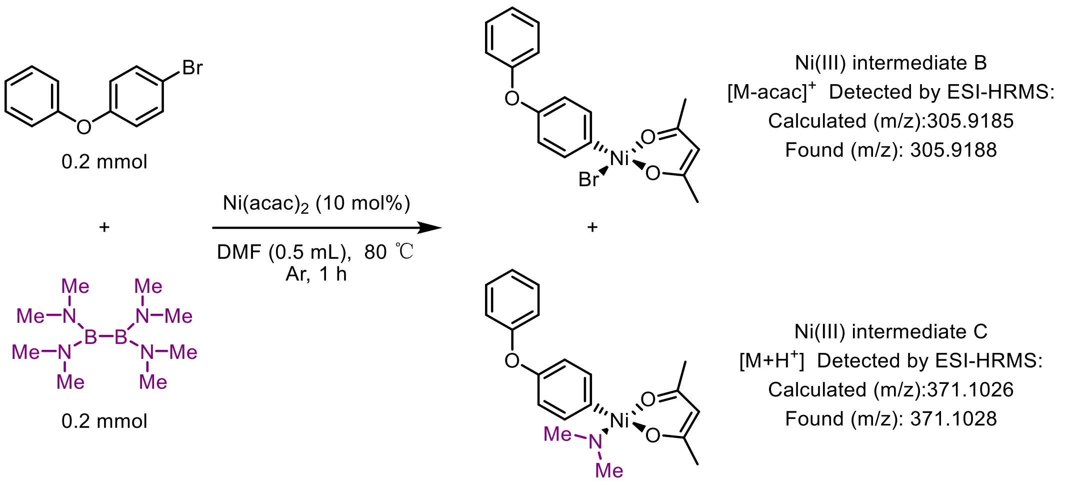


**Figure S20.** Detecting the generation of Ni(III) intermediate in solution

**Procedure :**

An oven-dried 4 mL vial was charged with a magnetic stir bar, aryl bromides (0.2 mmol, 1.0 equiv.), $Ni(acac)_2$ (0.02 mmol, 10 mol%), $B_2(NMe_2)_4$ (0.2 mmol, 1.0 equiv.), DMF (0.5 mL) in the glove box filled with argon gas. The vial was sealed with a plastic cap and then stirring was achieved by placing the assembled reactor at 80 °C on IKA C-MAG HS 7 control magnetic stir bars for 1 h. The ESI-HRMS analysis of the reaction solution exhibits a significant ion peak at a mass-to-charge (*m/z*) ratio of 305.9188 (calc. 305.9185), suggesting the generation of Ni(III) intermediate B. The ESI-HRMS analysis of the reaction solution exhibits a significant ion peak at a mass-to-charge (*m/z*) ratio of 371.1028 (calc. 371.1026), suggesting the generation of Ni(III) intermediate C.

***Note:***

After the reaction was heated and stirred for 1 hour, and HRMS sample was prepared. In order to ensure the oxygen-free HRMS condition, the whole process was carried out in the glove box filled with argon gas.

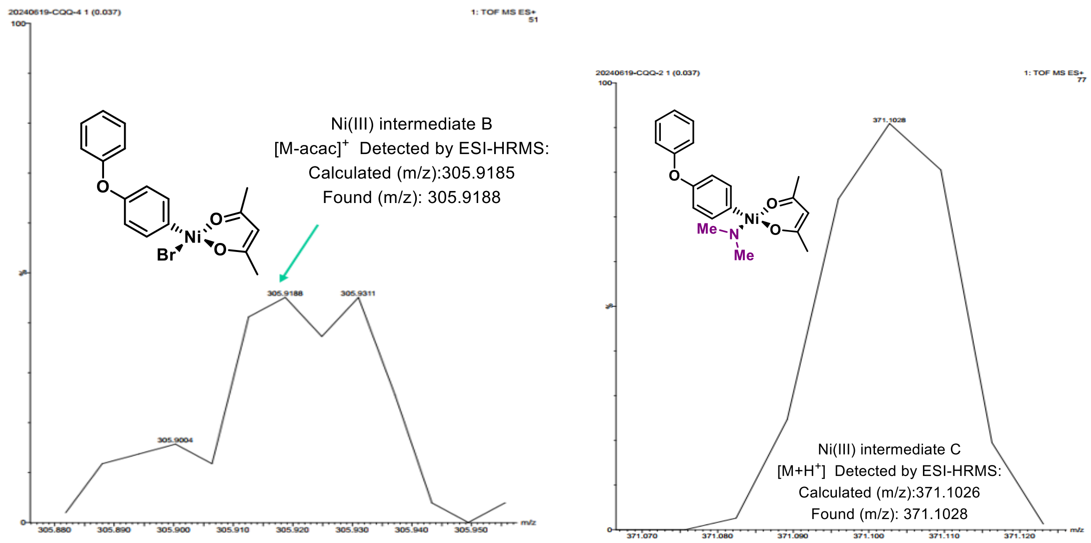


**Figure S21**. ESI-HRMS for Ni(III) intermediates

## 4.7 Detecting the key intermediates in solution by HRMS

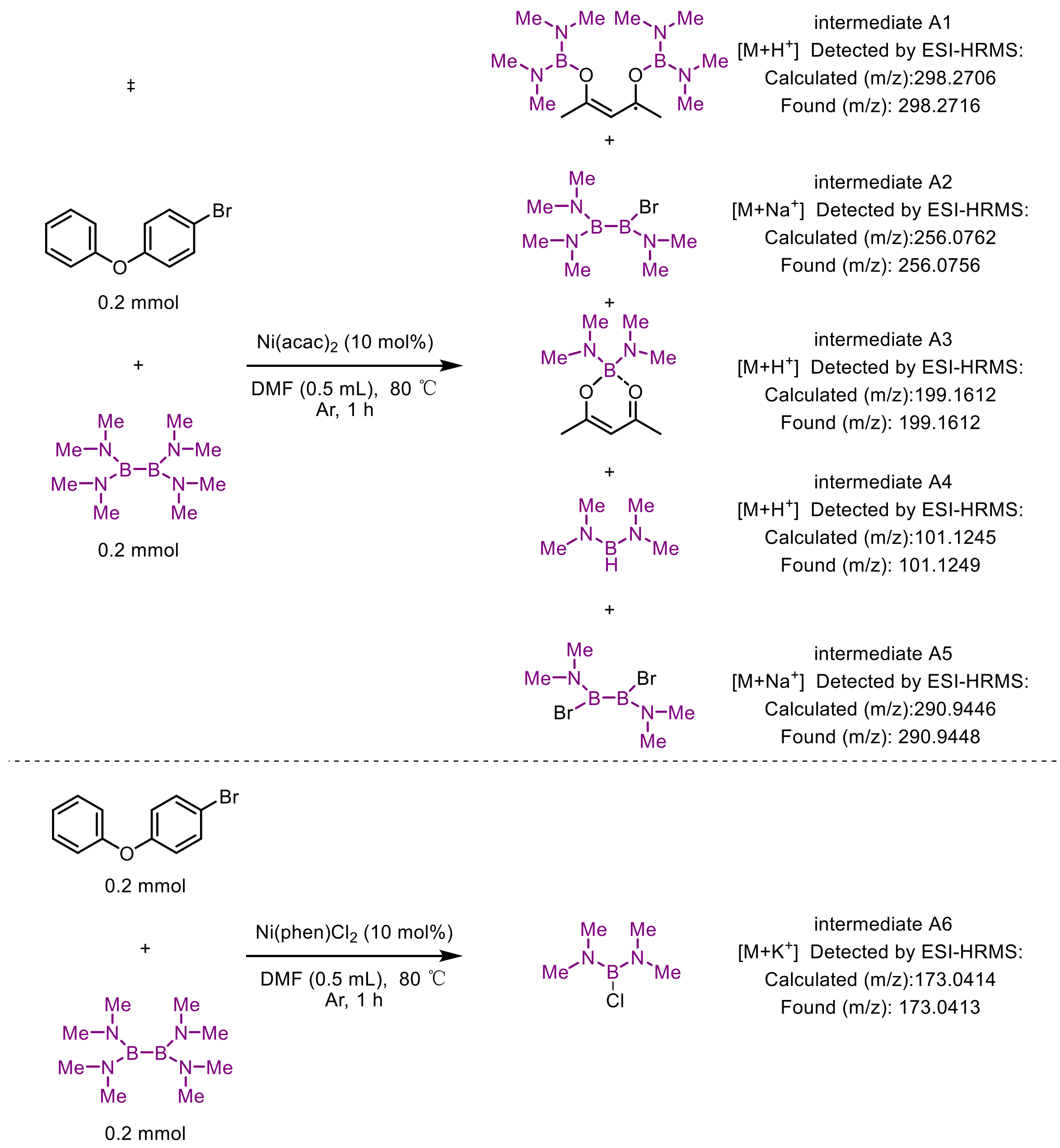


**Figure S22.** Detecting the key intermediates in solution by HRMS

**Procedure :**

An oven-dried 4 mL vial was charged with a magnetic stir bar, aryl bromides (0.2 mmol, 1.0 equiv.), $Ni(acac)_2$ or $Ni(phen)Cl_2$ (0.02 mmol, 10 mol%), $B_2(NMe_2)_4$ (0.2 mmol, 1.0 equiv.), DMF (0.5 mL) in the glove box. The vial was sealed with a plastic cap and then stirring was achieved by placing the assembled reactor at 80 ℃ on IKA C-MAG HS 7 control magnetic stir bars for 1 h. The ESI-HRMS analysis of the reaction solution exhibits a significant ion peak at a mass-to-charge (*m/z*) ratio of 298.2716 (calc. 298.2706), suggesting the generation of intermediate A1. The ESI-HRMS analysis of the reaction solution exhibits a significant ion peak at a mass-to-charge (*m/z*) ratio of 256.0762 (calc. 256.0756), suggesting the generation of intermediate A2. The ESI-HRMS analysis

of the reaction solution exhibits a significant ion peak at a mass-to-charge (*m/z*) ratio of 199.1612 (calc. 199.1612), suggesting the generation of intermediate A3. The ESI-HRMS analysis of the reaction solution exhibits a significant ion peak at a mass-to-charge (*m/z*) ratio of 101.1245 (calc. 101.1249), suggesting the generation of intermediate A4. The ESI-HRMS analysis of the reaction solution exhibits a significant ion peak at a mass-to-charge (*m/z*) ratio of 290.9448 (calc. 290.9446), suggesting the generation of intermediate A5. The ESI-HRMS analysis of the reaction solution exhibits a significant ion peak at a mass-to-charge (*m/z*) ratio of 173.0413 (calc. 173.0414), suggesting the generation of intermediate A6.

***Note:***

After the reaction was heated and stirred for 1 hour, and HRMS sample was prepared. In order to ensure the oxygen-free HRMS condition, the whole process was carried out in the glove box filled with argon gas.

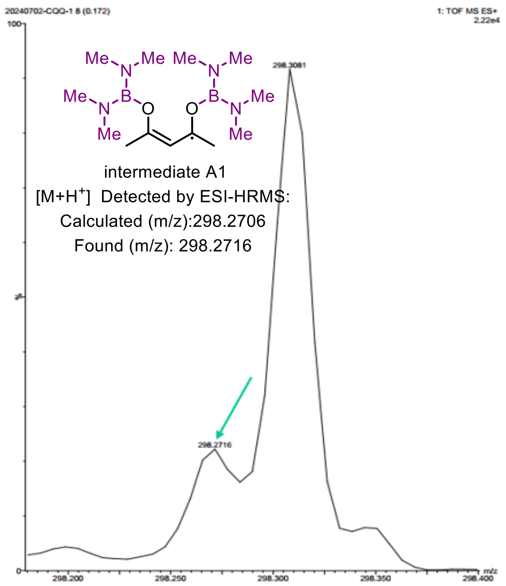


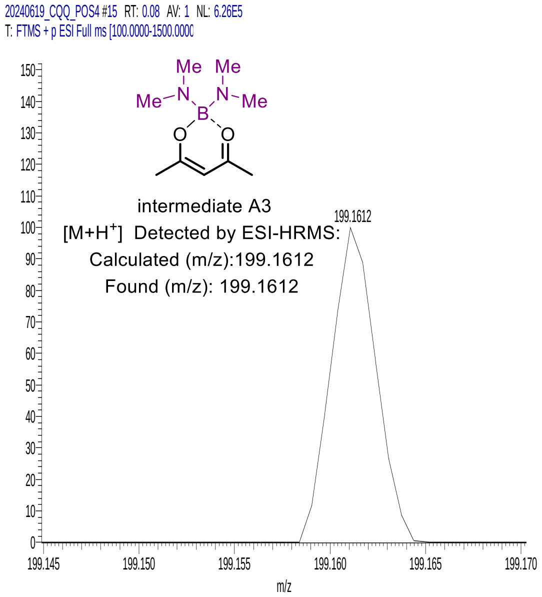


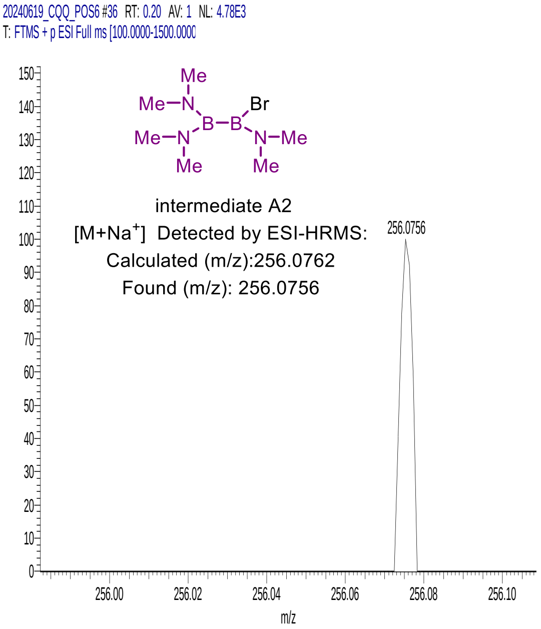


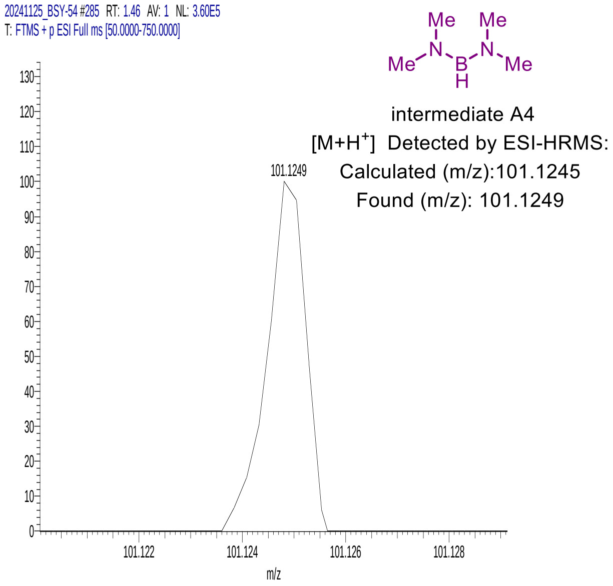

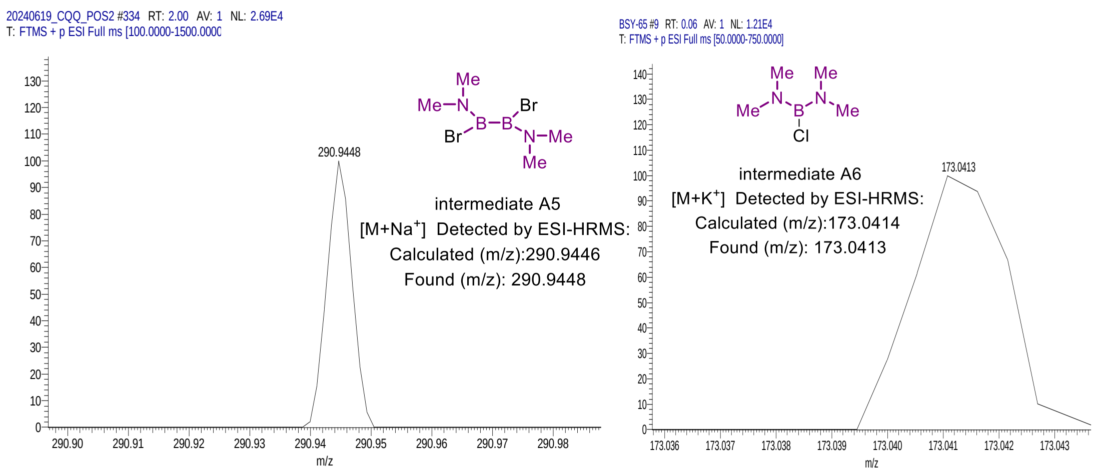


**Figure S23**. ESI-HRMS for the key intermediates

## 4.8 Dimethylamine source confirmation

We examined the impact of $B_2(NMe_2)_4$ loading. No desired product was detected with 0.25 equivalents of $B_2(NMe_2)_4$, suggesting that a portion of $B_2(NMe_2)_4$ is consumed during the initial single-electron transfer (SET) process. The yield increased to 42% with 0.5 equivalents of $B_2(NMe_2)_4$ and significantly improved to 85% with 0.7 equivalents. Notably, yields of 88% were achieved with 0.8 equivalents of $B_2(NMe_2)_4$, indicating that, in addition to $B_2(NMe_2)_4$, other dimethylamine sources participate in the C-N bond formation reaction. We also captured the by product (**A5**: *HRMS* [M+Na$^+$]: 290.9448) of **A2**, showcasing the fact that **A2** is one of the dimethylamine sources.

**A: dominant pathway**

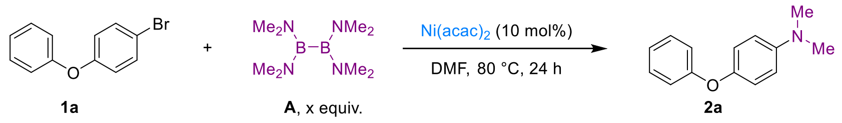


| $B_2(NMe_2)_4$/equiv. | 0.25 | 0.5 | 0.7 | 0.8 | 0.9 | 1.0 |
|---|---|---|---|---|---|---|
| yield of **2a** | <5% | 42% | 85% | 88% | 92% | 94% |

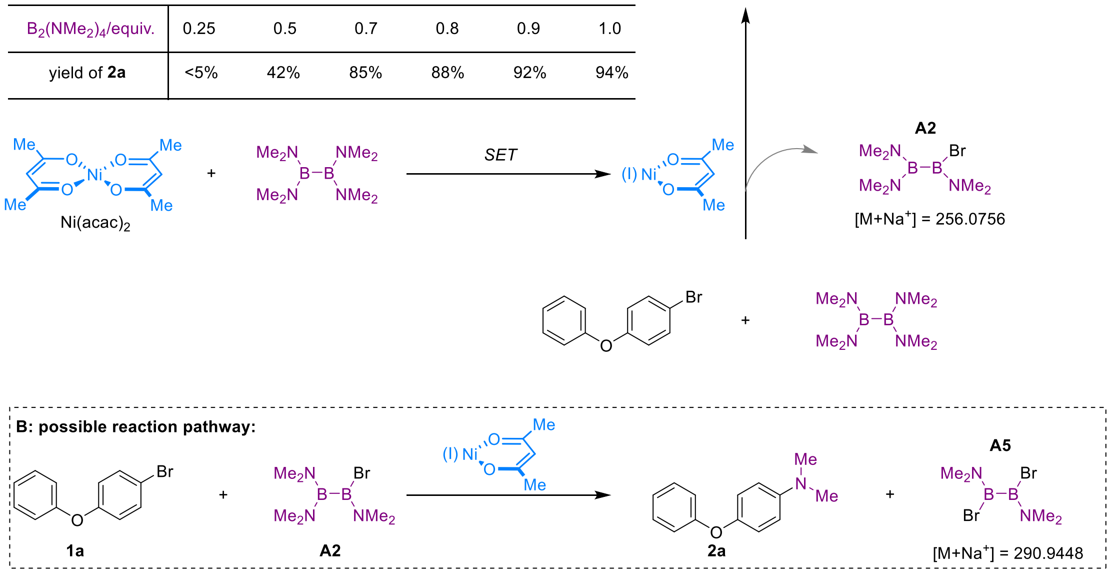


**Figure S24**. Dimethylamine source confirmation

## 4.9 DFT calculation study

All density functional theory (DFT) calculations were conducted with the Gaussian 16 program.[70] Geometry optimization was performed with dispersion-corrected M06L-D3 functional.[71] SDD basis set with ECP was used for Ni, the 6-31G(d,p) basis set was used for other atoms.[72] Frequency analysis was conducted at the same level of theory to verify the stationary points to be energy minimum or a transition state and to obtain the thermal energy corrections. Single point energies were calculated with M06L-D3 and a mixed basis set SDD for Ni and 6-311+G(d,p) for the other atoms. Solvent effect (Solvent= *N,N*-dimethylformamide) was calculated by using SMD solvation model.[73] The relative energies with ZPE corrections and free energies are in kcal/mol.

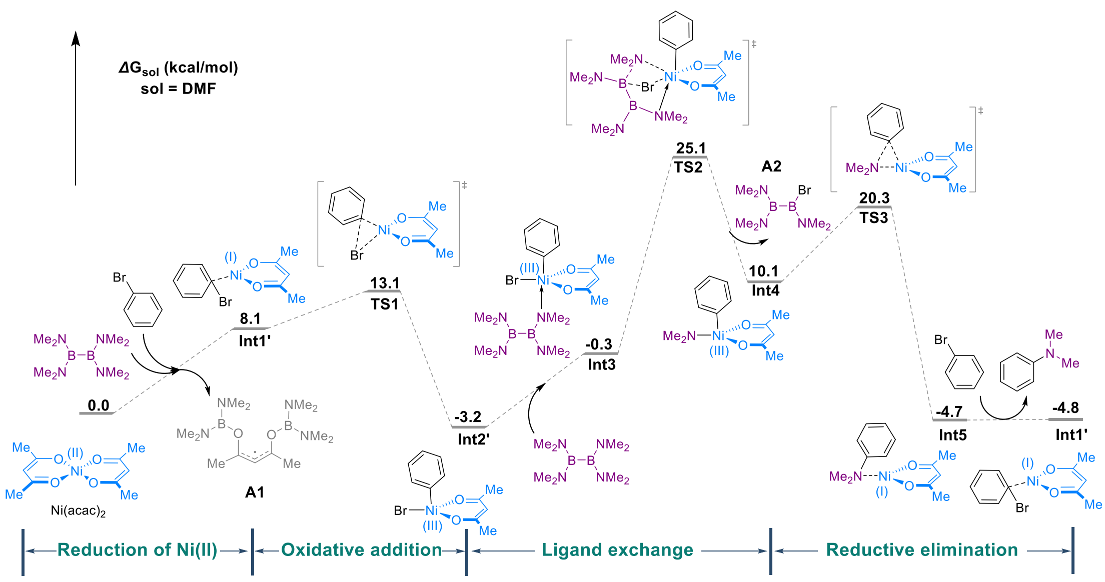


**Figure S25.** Energy profile of reduction of Ni(II) species and subsequent Ni(I)/Ni(III) catalytic cycle excluding explicit solvent DMF.

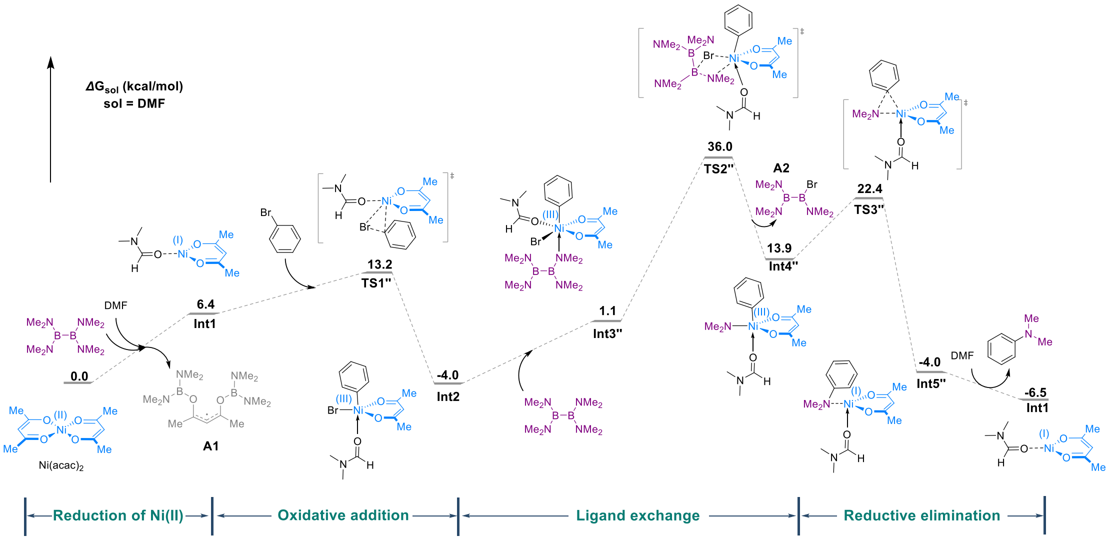


**Figure S26**. Energy profile of reduction of Ni(II) species and subsequent Ni(I)/Ni(III) catalytic cycle considering explicit solvent DMF for every step.

We have conducted a thorough reassessment of the solvents' role in our reaction, with a particular

focus on their influence on the stabilization of boronyl radical species and nickel coordination. In the main text, we have considered both **Figure S25** and **Figure S26**, and selected the structures with the lowest energy as the potential energy surface.

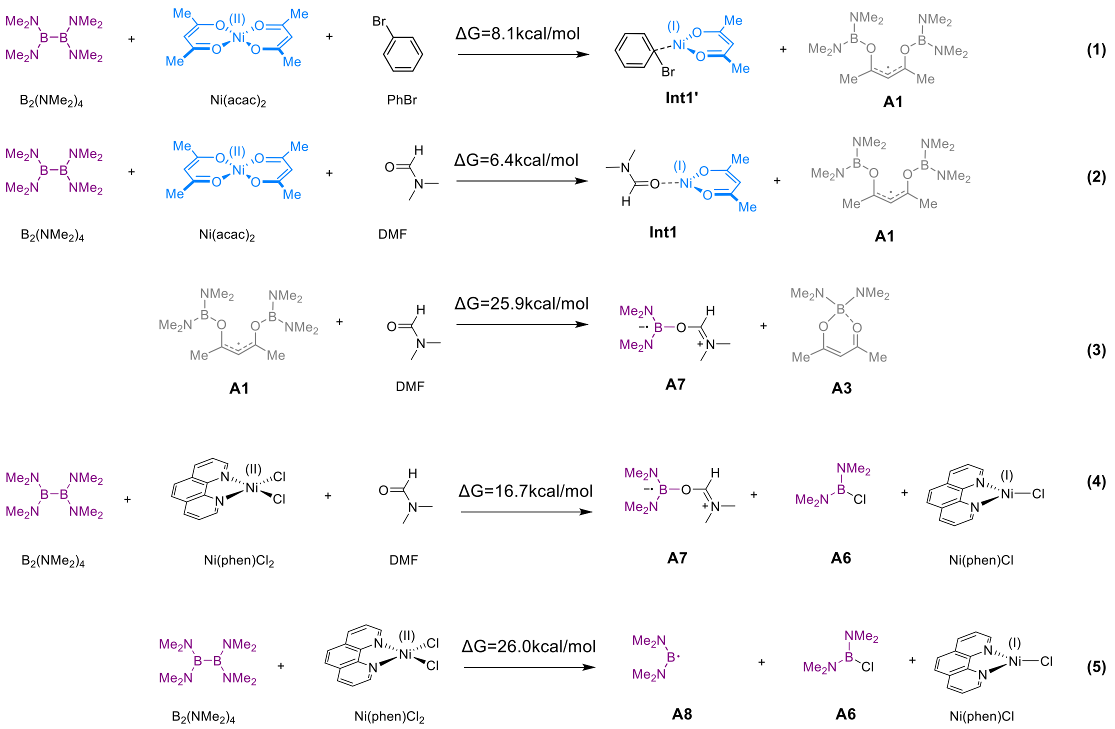


**Figure S27**. Reaction energies of reduction of Ni(II) species and plausible stablizing effect of DMF and acac ligand.

After conducting further calculations for Ni(acac)$_2$, we examined the interaction between DMF and the boronyl radical, ultimately determining that no further energy reduction could be attained through this coordination, as an additional 25.9 kcal/mol is required for DMF to coordinate with the boronyl radical in **A1** (**Figure S27 (3)**). Nevertheless, for the SET process of Ni(phen)Cl$_2$, the stabilizing effect of DMF on the boronyl radical significantly reduces the reaction energy from 26.0 kcal/mol to 16.7 kcal/mol (**Figure S27 (4)&(5)**). It is possible that the acac ligand confers greater stability to the boronyl species, as the conjugated structures within the acac ligand can potentially enhance the stability of radical species **A1**. Our findings also revealed that the coordination of the solvent to nickel does enhance the stability of the nickel intermediates. Specifically, the ΔG of the intermediate **Int1** is 6.4 kcal/mol, while the energy ΔG of the intermediate **Int1'** is 8.1 kcal/mol (**Figure S27 (1)&(2)**).

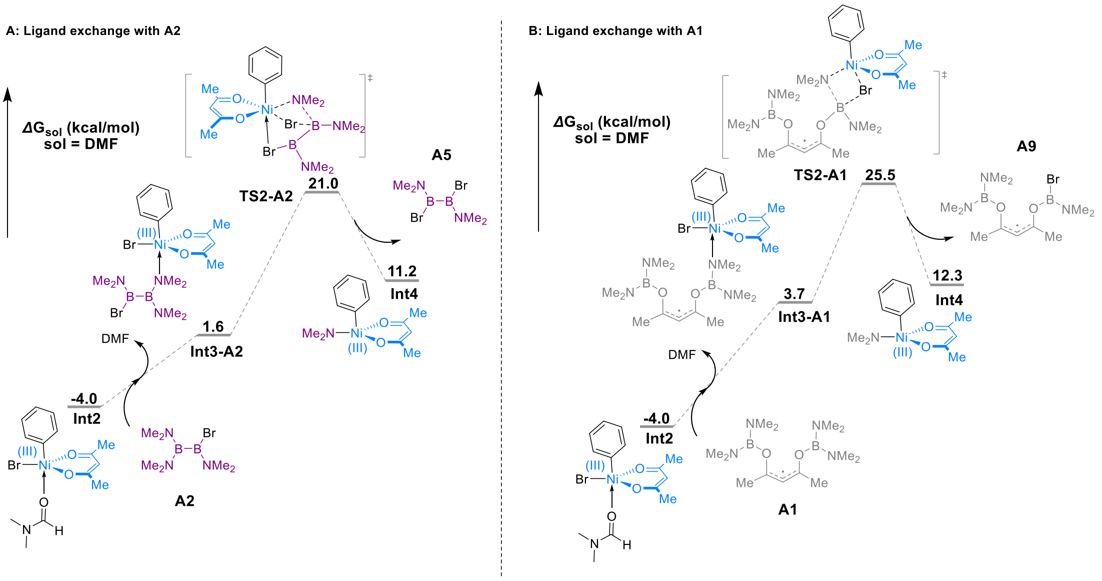


**Figure S28**. Energy profile of ligand exchange process considering **A1** and **A2**.

We calculated the likelihood of **A1** and **A2** participating in the ligand exchange process. The DFT results indicate that **A2**'s involvement in the reaction reduces the energy of the rate-determining step from 25.1 kcal/mol to 21.0 kcal/mol (**Figure S28A**). Conversely, **A1**'s participation slightly increases the energy of the rate-determining step, from 25.1 kcal/mol to 25.5 kcal/mol (**Figure S28B**). Combining the DFT results and the experimental findings from the $B_2(NMe_2)_4$ loading experiments and *HRMS*, it is highly plausible that **A2** acts as an additional dimethylamine source in the reaction.

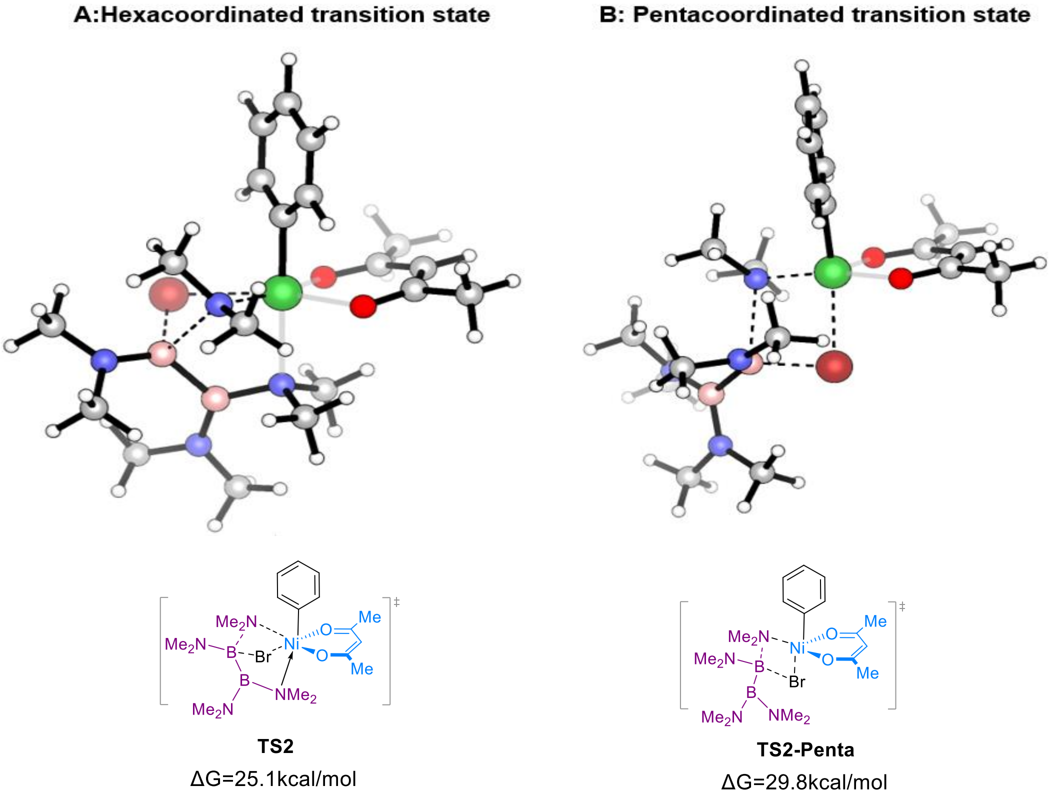

**Figure S29**. Comparison of energy and structure between hexacoordinated transition state and pentacoordinated transition state.

The comparison between the hexacoordinated and pentacoordinated transition states reveals that the hexacoordinated transition state is more stable. This increased stability can be attributed to the coordination interaction between another dimethylamine group and the nickel center, making the hexacoordinated transition state more stable compared to the pentacoordinated one.

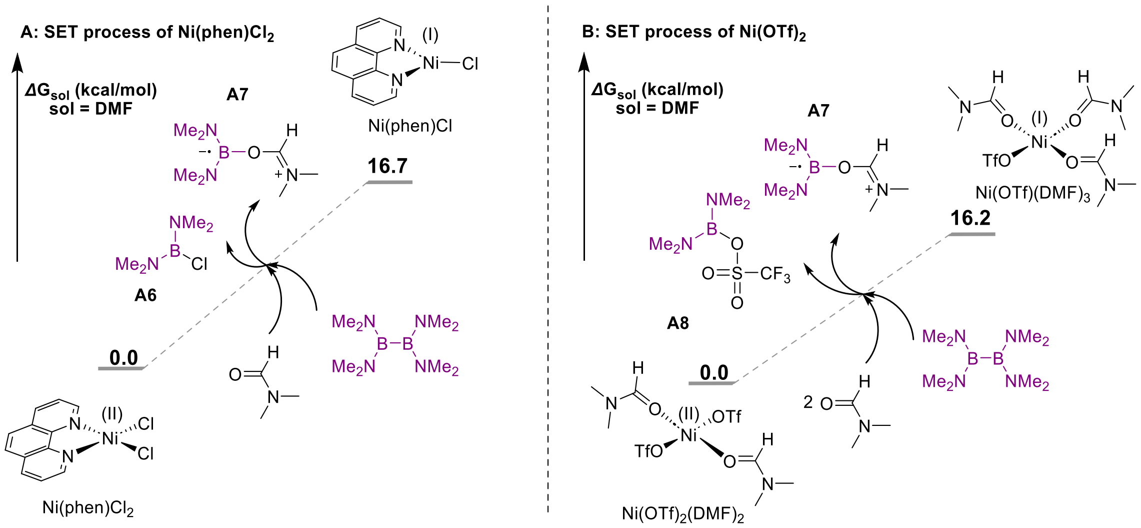


**Figure S30.** Energy Profiles of SET Processes for Ni(phen)$Cl_2$ and Ni$(OTf)_2$.

The acac ligand plays a crucial role in the formation of Ni(I) through the single-electron transfer (SET) process, facilitating the generation of an intermediate **Int1**. Notably, other nickel catalysts without the acac ligand can also undergo reactions. To gain further insights, we conducted new calculations employing Ni(phen)$Cl_2$ and Ni$(OTf)_2(DMF)_2$ as model catalysts, focusing on the difference of the SET process, and DMF solvent was also considered in stabilizing the boronyl radical and nickel complex through coordination. Our computational results revealed that for Ni(phen)$Cl_2$, the reaction is endothermic by 16.7 kcal/mol, while for Ni$(OTf)_2(DMF)_2$, it is endothermic by 16.2 kcal/mol. Although these values are higher than the energy required for SET in Ni$(acac)_2$ (6.4 kcal/mol), they are accessible at the reaction temperature (80 °C).

# 5. NMR Spectroscopic Data

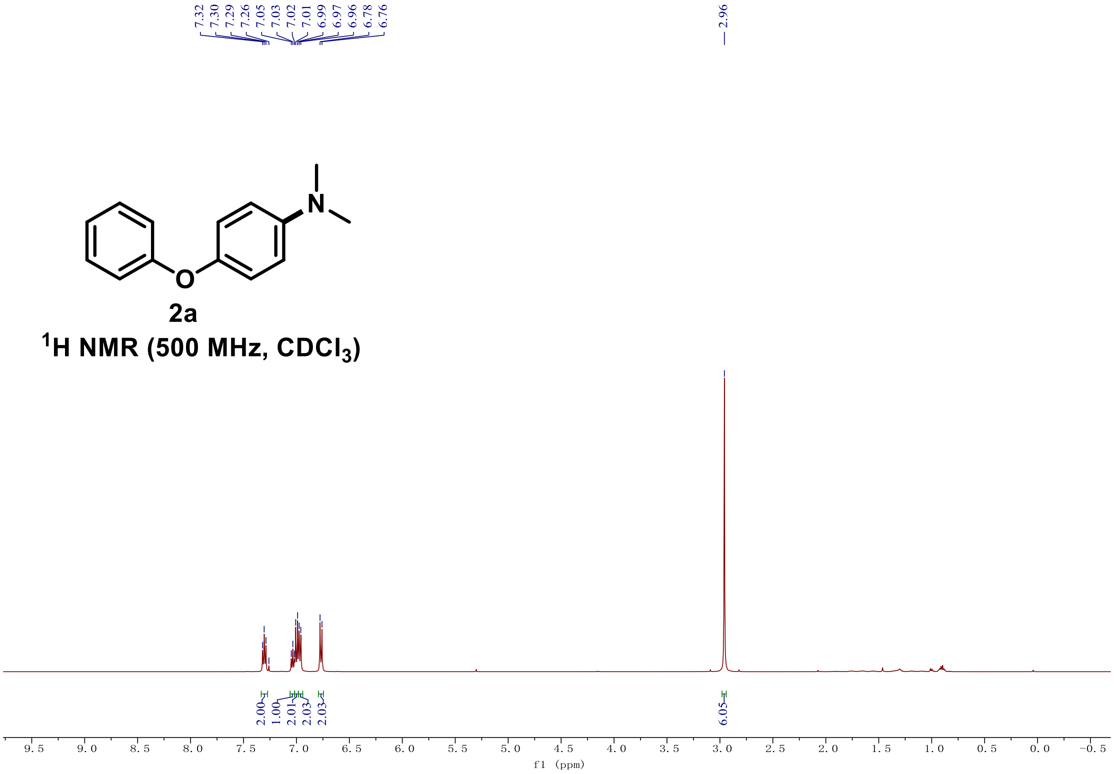


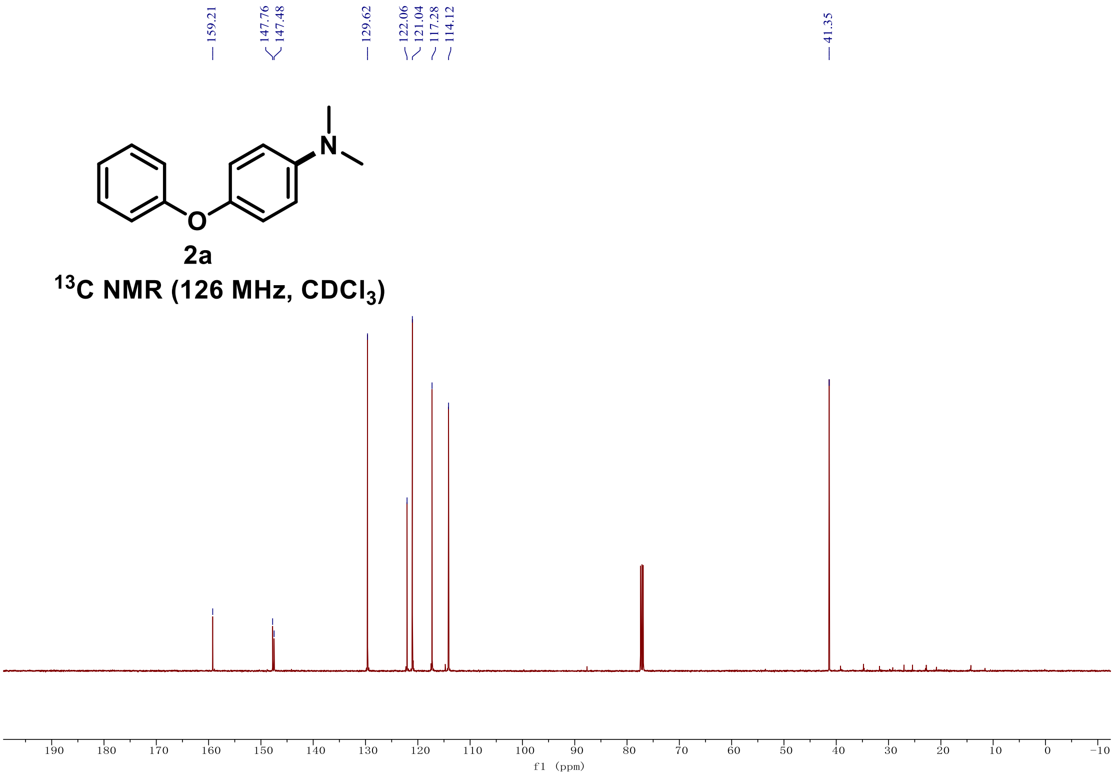

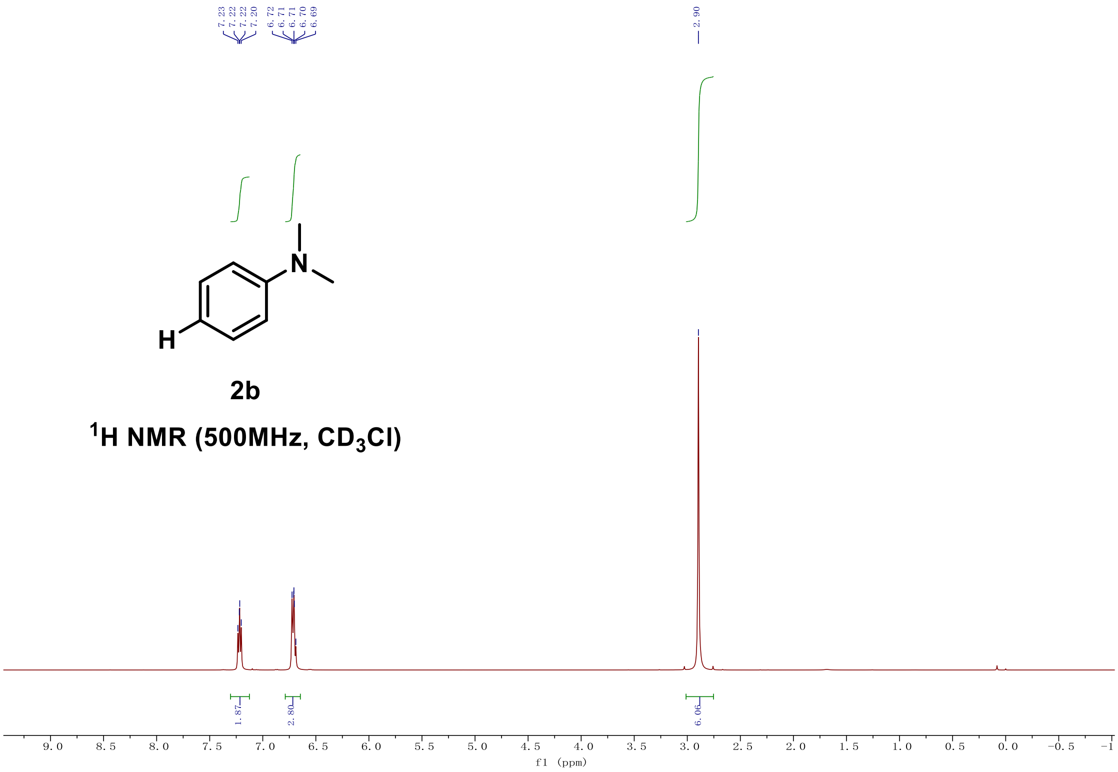
7.23
7.22
7.22
7.20
6.72
6.71
6.71
6.70
6.69
2.90
H
N
2b
$^1$H NMR (500MHz, $CD_3Cl$)
1.87
2.80
6.06
f1 (ppm)

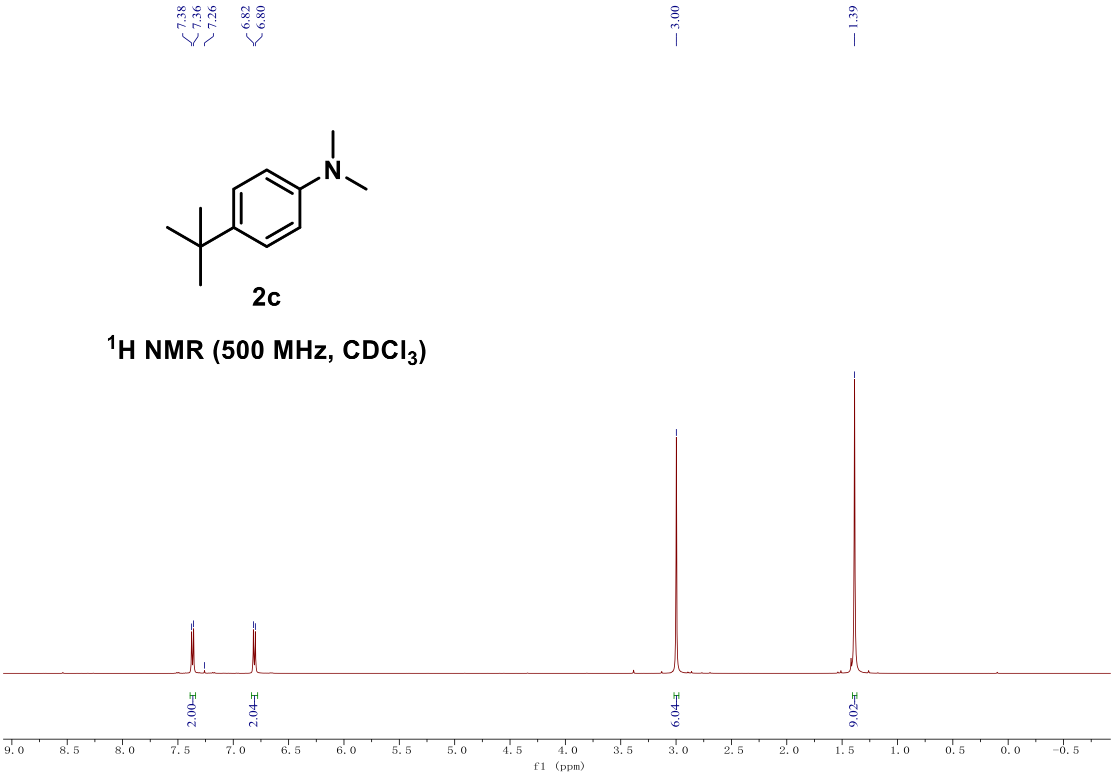
7.38
7.36
7.26
6.82
6.80
3.00
1.39
N
2c
$^1$H NMR (500 MHz, $CDCl_3$)
2.00
2.04
6.04
9.02
f1 (ppm)

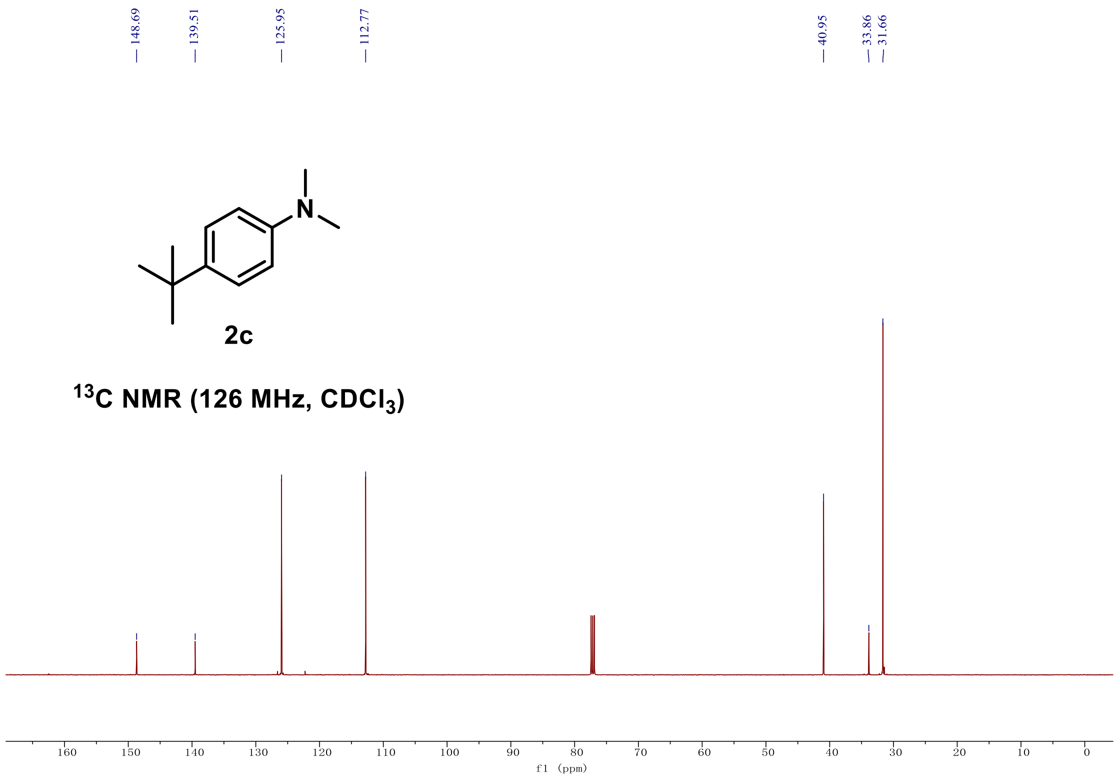
148.69
139.51
125.95
112.77
40.95
33.86
31.66
2c
$^{13}$C NMR (126 MHz, CDCl$_3$)
f1 (ppm)

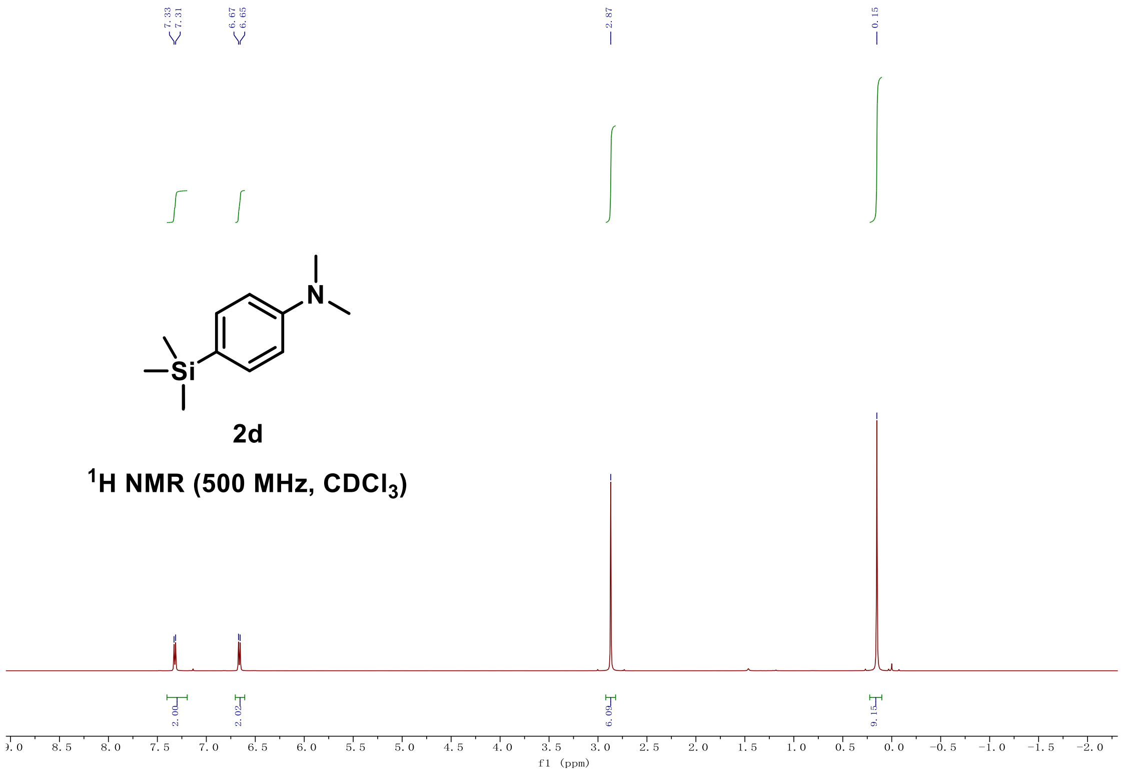
7.33
7.31
6.67
6.65
2.87
0.15
2d
$^{1}$H NMR (500 MHz, CDCl$_3$)
2.00
2.02
6.09
9.15
f1 (ppm)

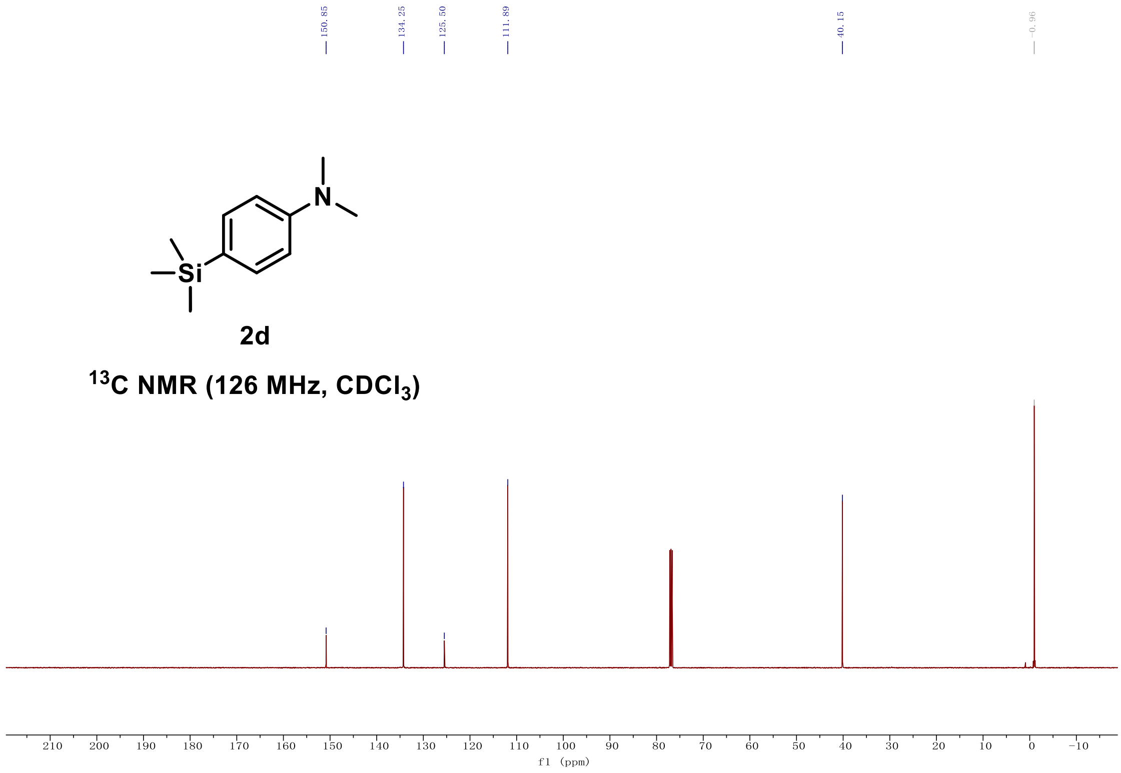


**2d**

**$^{13}$C NMR (126 MHz, CDCl$_3$)**

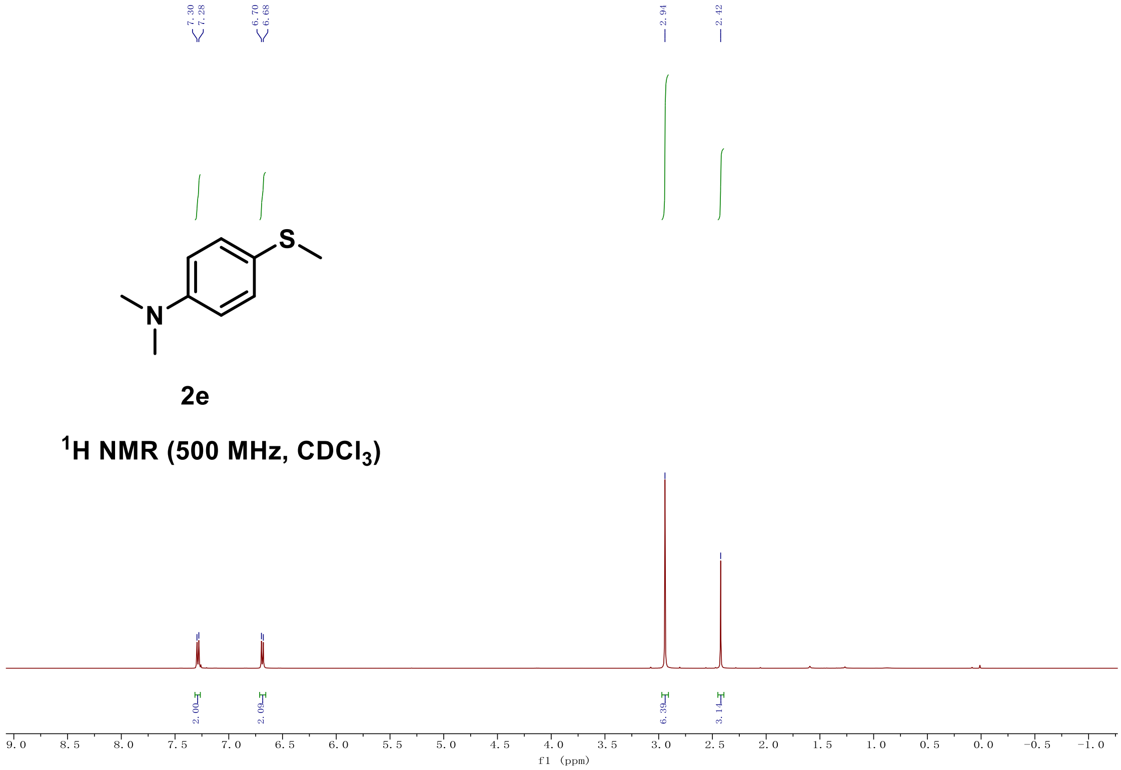


**2e**

**$^{1}$H NMR (500 MHz, CDCl$_3$)**

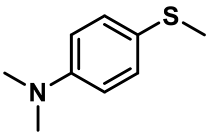

**2e**

**$^{13}$C NMR (126 MHz, CDCl$_3$)**

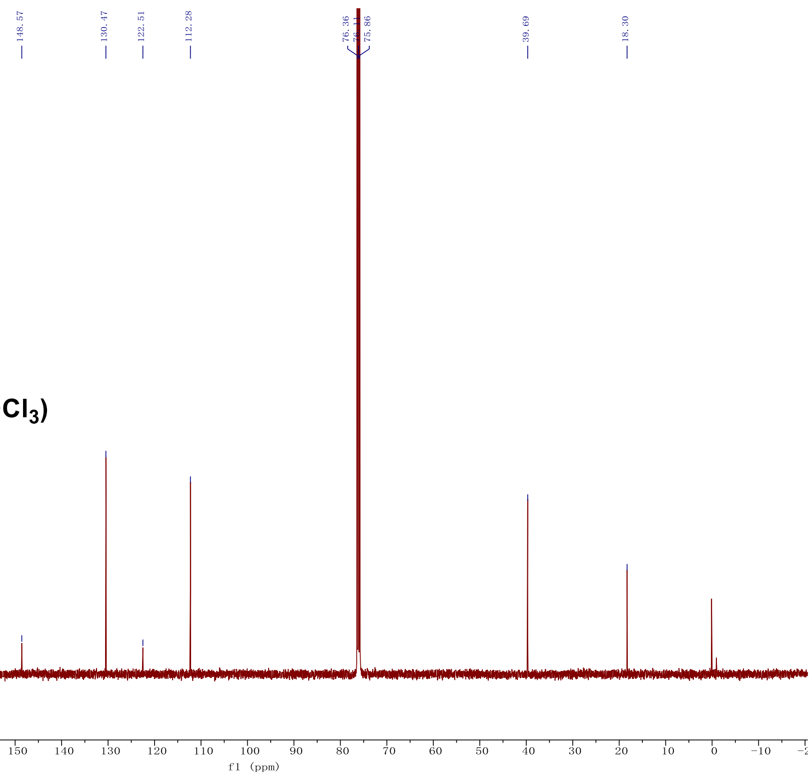


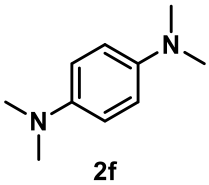

**2f**

**$^{1}$H NMR (500 MHz, CDCl$_3$)**

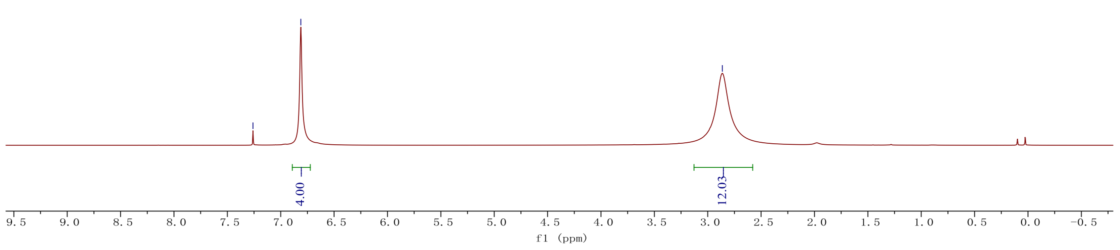

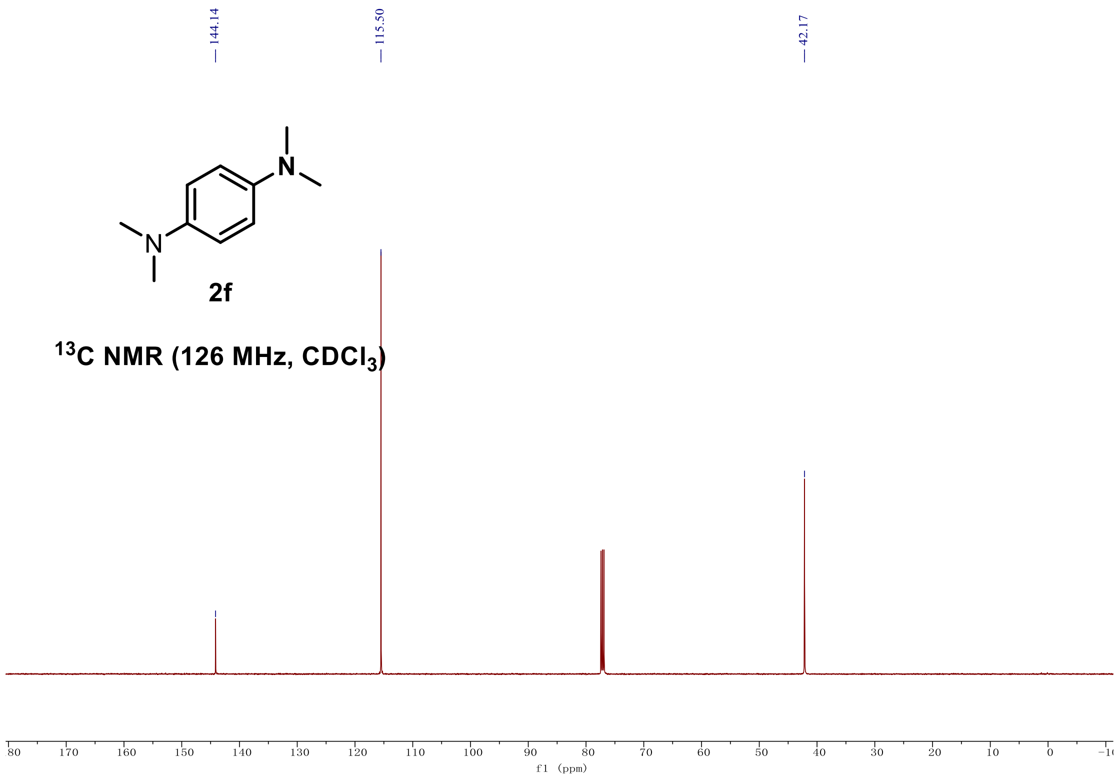
144.14
115.50
42.17
N
N
2f
13C NMR (126 MHz, CDCl3)
80 170 160 150 140 130 120 110 100 90 80 70 60 50 40 30 20 10 0
f1 (ppm)

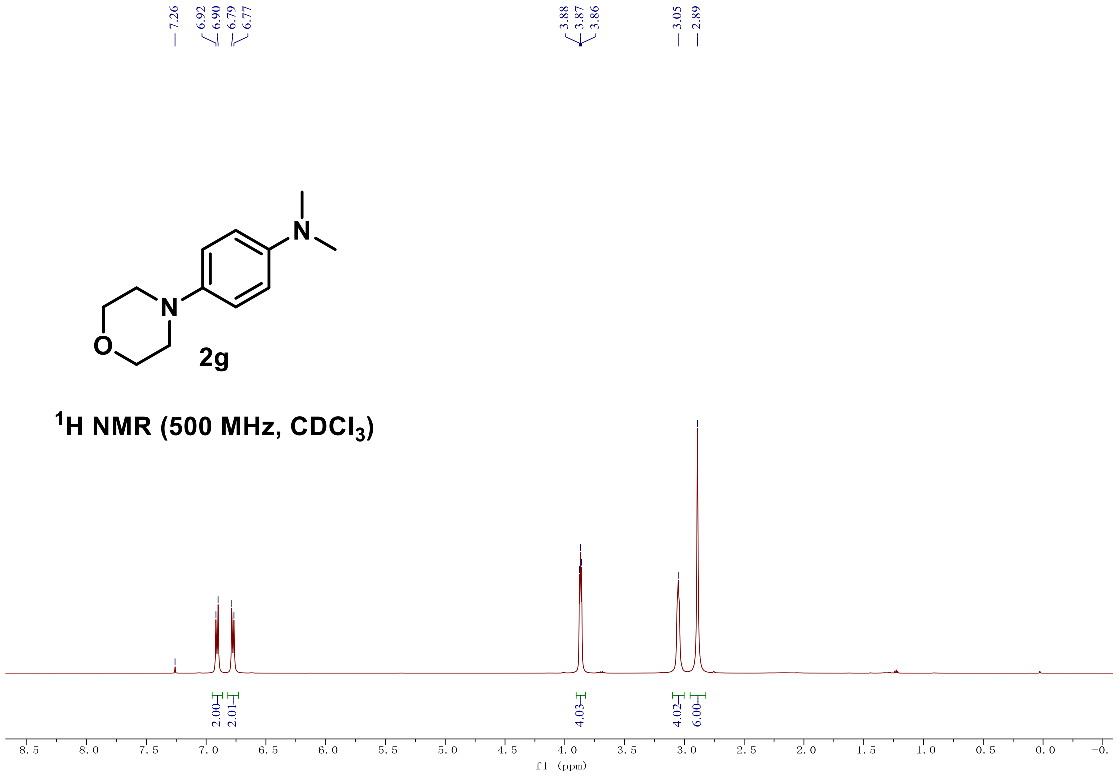
7.26
6.92
6.90
6.79
6.77
3.88
3.87
3.86
3.05
2.89
N
N
O
2g
1H NMR (500 MHz, CDCl3)
2.00
2.01
4.03
4.02
6.00
8.5 8.0 7.5 7.0 6.5 6.0 5.5 5.0 4.5 4.0 3.5 3.0 2.5 2.0 1.5 1.0 0.5 0.0
f1 (ppm)

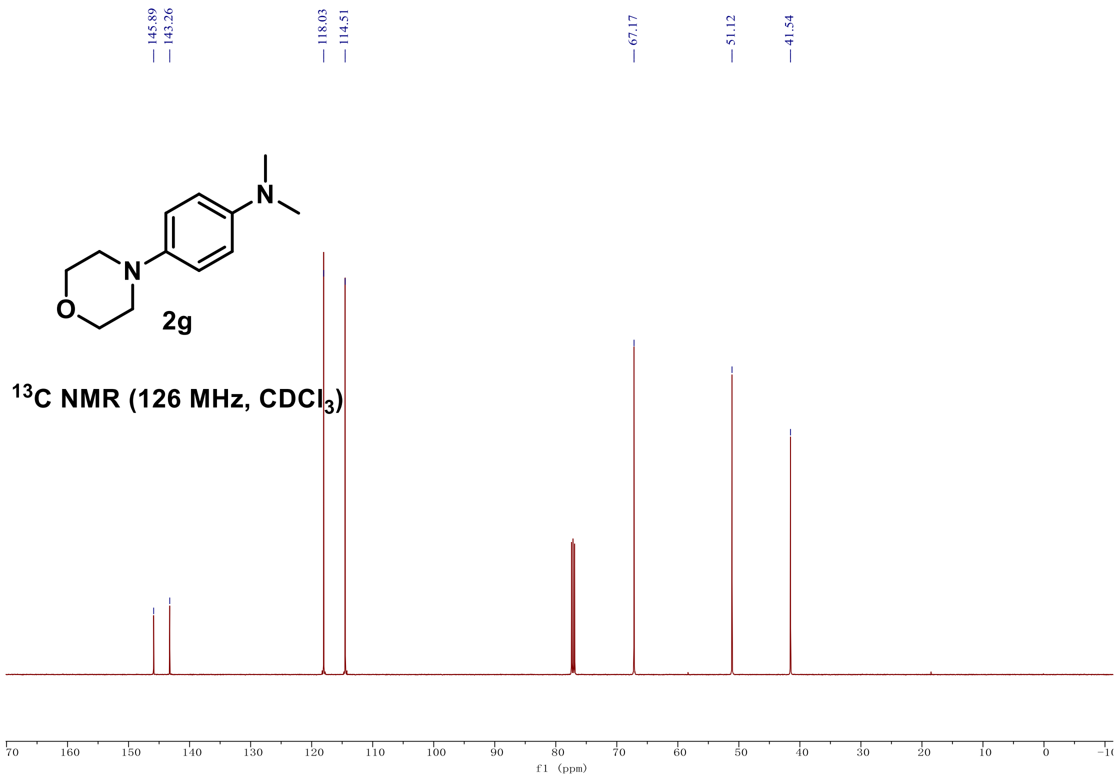
145.89
143.26
118.03
114.51
67.17
51.12
41.54
2g
13C NMR (126 MHz, CDCl3)
f1 (ppm)

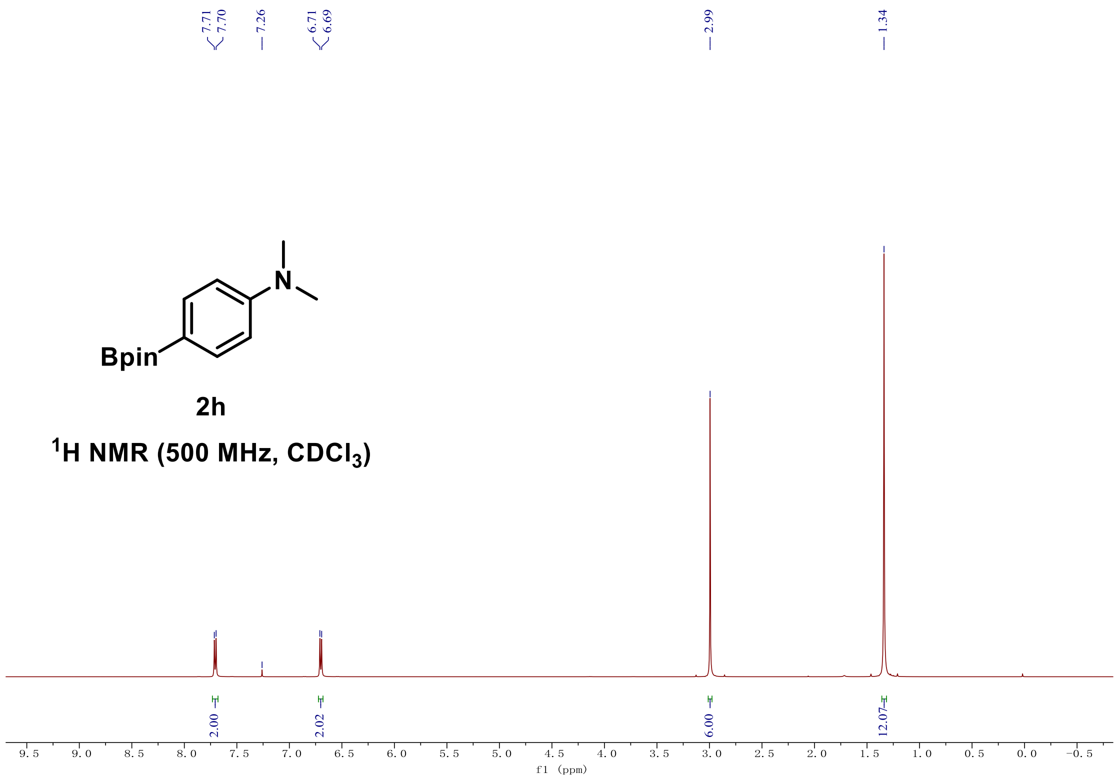
7.71
7.70
7.26
6.71
6.69
2.99
1.34
Bpin
2h
1H NMR (500 MHz, CDCl3)
2.00
2.02
6.00
12.07
f1 (ppm)

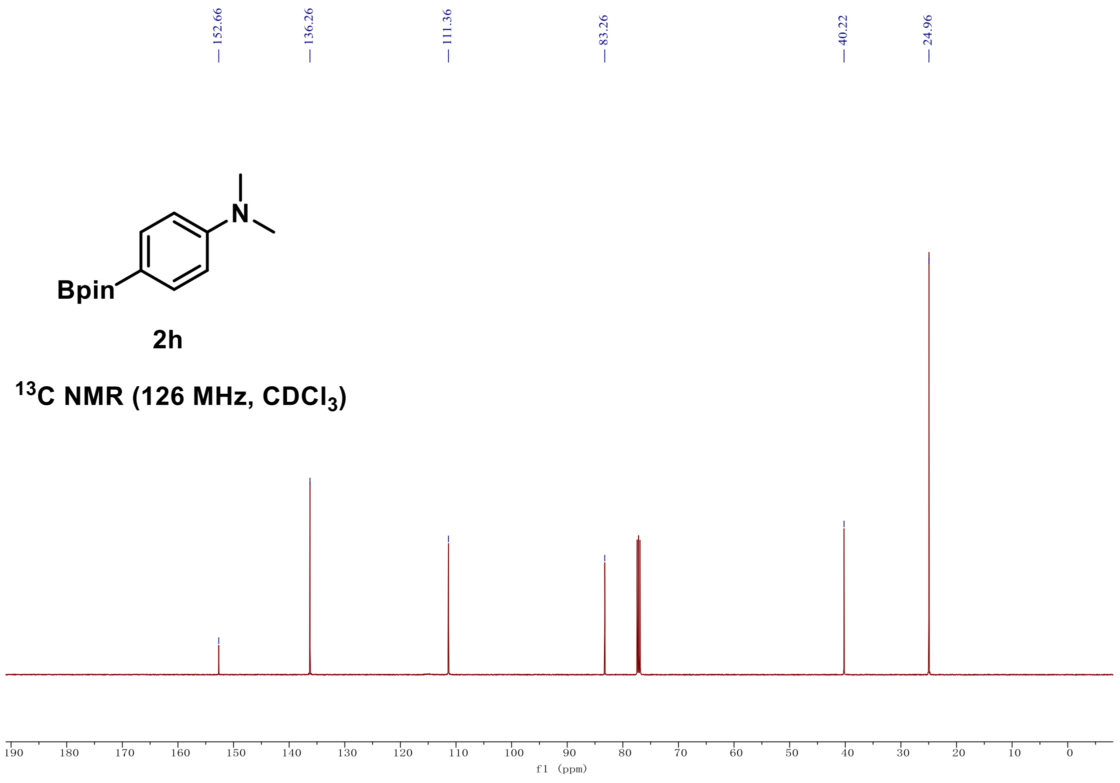
152.66
136.26
111.36
83.26
40.22
24.96
Bpin
N
2h
$^{13}C$ NMR (126 MHz, $CDCl_3$)
f1 (ppm)

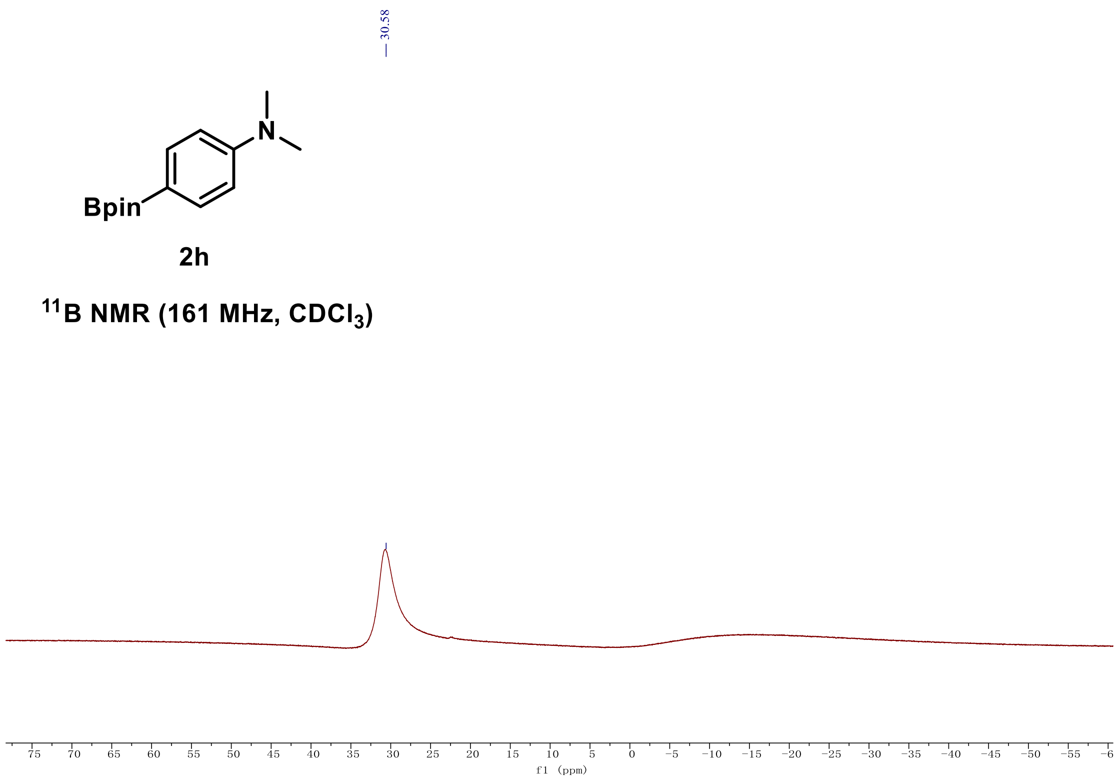
30.58
Bpin
N
2h
$^{11}B$ NMR (161 MHz, $CDCl_3$)
f1 (ppm)

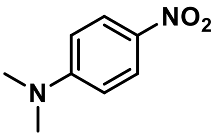

**2i**

$^{1}H$ NMR (500 MHz, $CDCl_3$)

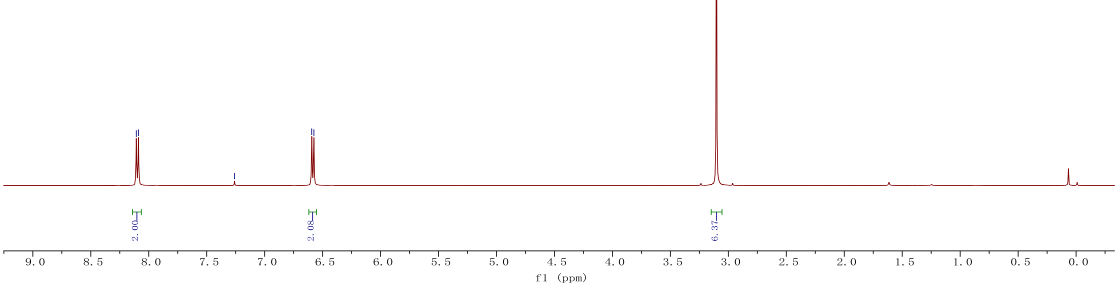


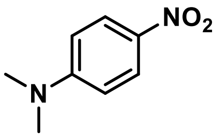

**2i**

$^{13}C$ NMR (126 MHz, $CDCl_3$)

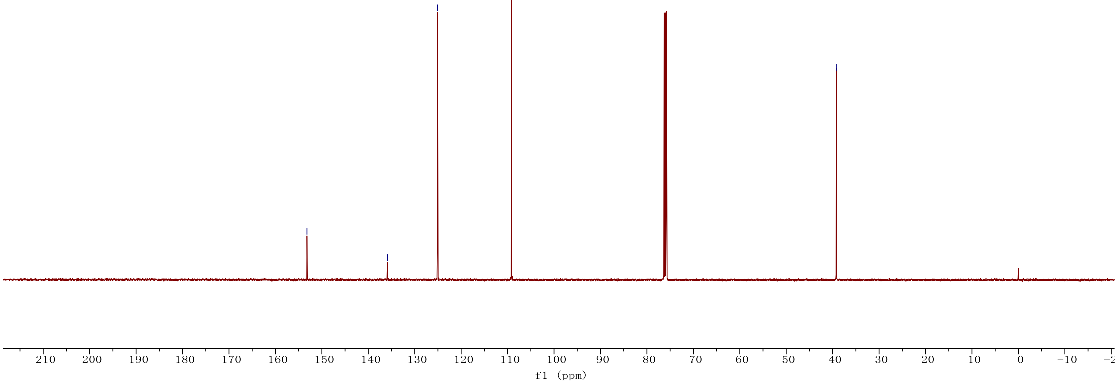

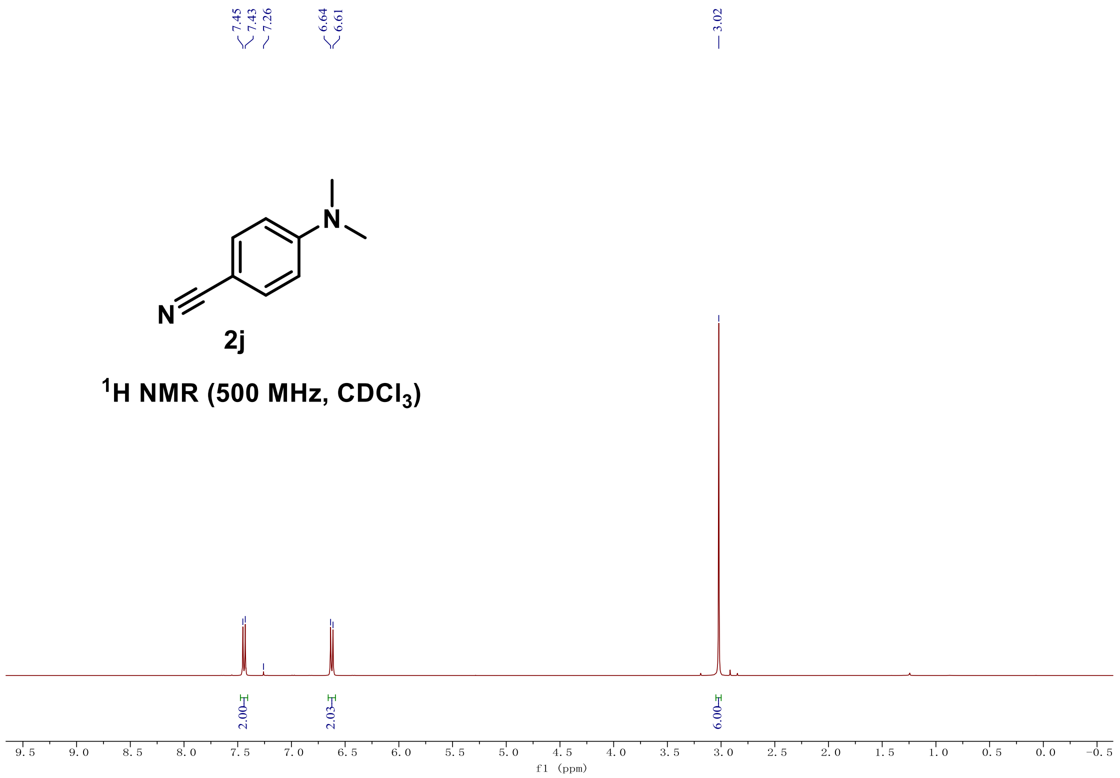
7.45
7.43
7.26
6.64
6.61
3.02
N
N
2j
$^{1}$H NMR (500 MHz, $CDCl_3$)
2.00
2.03
6.00
9.5 9.0 8.5 8.0 7.5 7.0 6.5 6.0 5.5 5.0 4.5 4.0 3.5 3.0 2.5 2.0 1.5 1.0 0.5 0.0 -0.5
f1 (ppm)

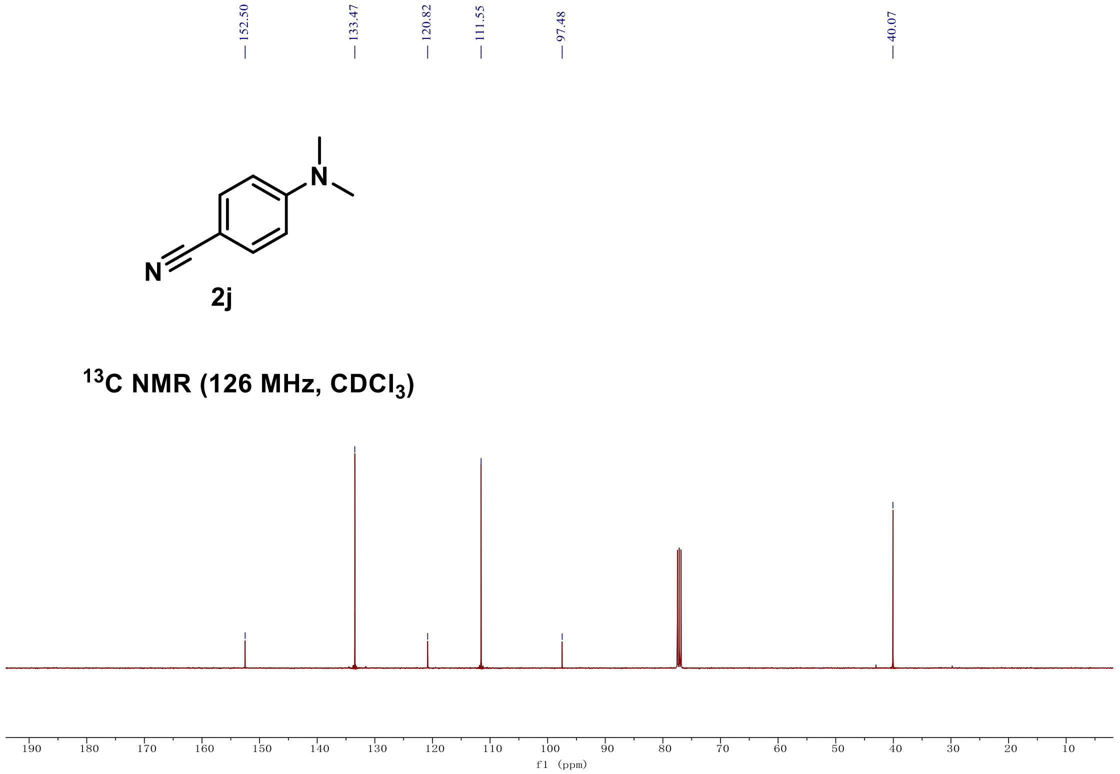
152.50
133.47
120.82
111.55
97.48
40.07
N
N
2j
$^{13}$C NMR (126 MHz, $CDCl_3$)
190 180 170 160 150 140 130 120 110 100 90 80 70 60 50 40 30 20 10
f1 (ppm)

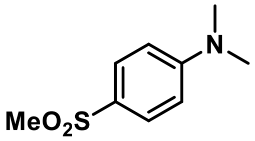

**2k**

**$^{1}H$ NMR (500 MHz, $CDCl_3$)**

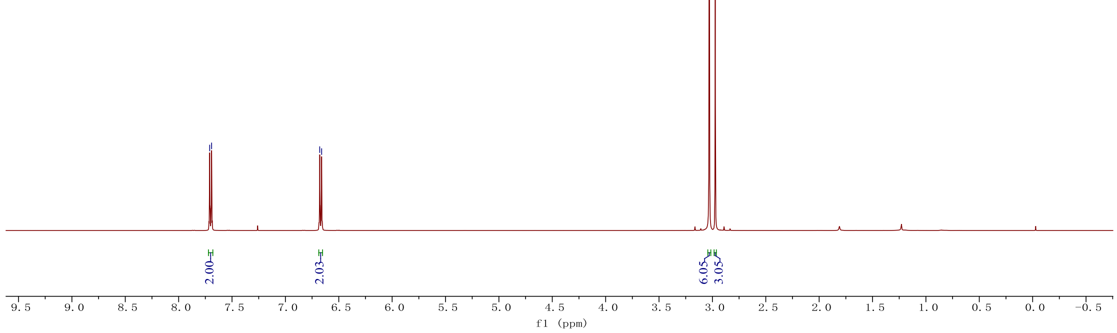


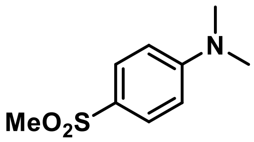

**2k**

**$^{13}C$ NMR (126 MHz, $CDCl_3$)**

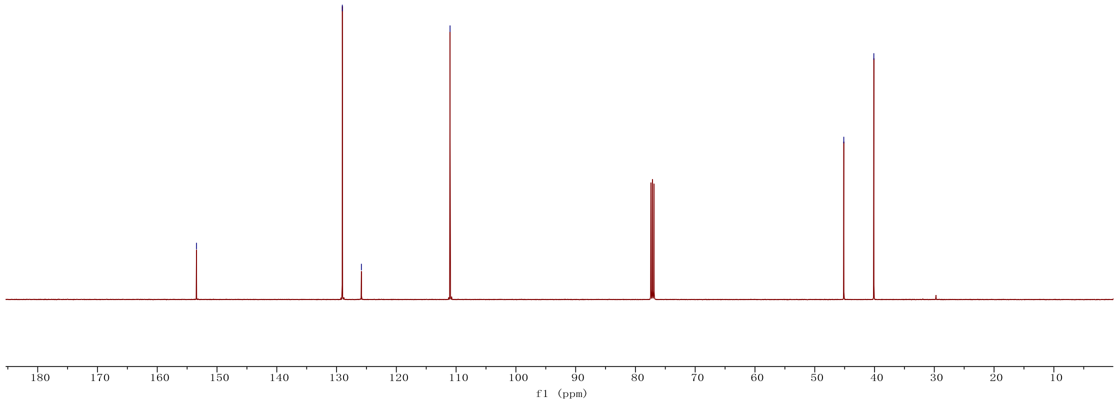

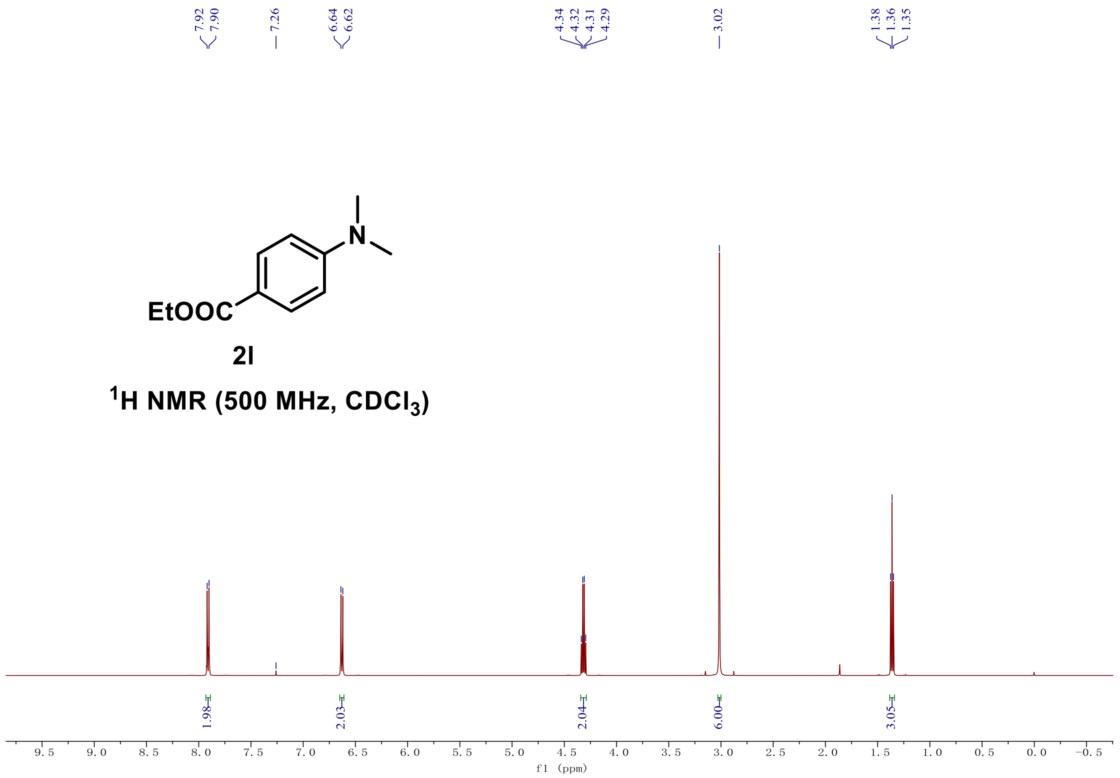
7.92
7.90
7.26
6.64
6.62
4.34
4.32
4.31
4.29
3.02
1.38
1.36
1.35
EtOOC
N
2l
1H NMR (500 MHz, CDCl3)
1.98
2.03
2.04
6.00
3.05
9.5 9.0 8.5 8.0 7.5 7.0 6.5 6.0 5.5 5.0 4.5 4.0 3.5 3.0 2.5 2.0 1.5 1.0 0.5 0.0 -0.5
f1 (ppm)

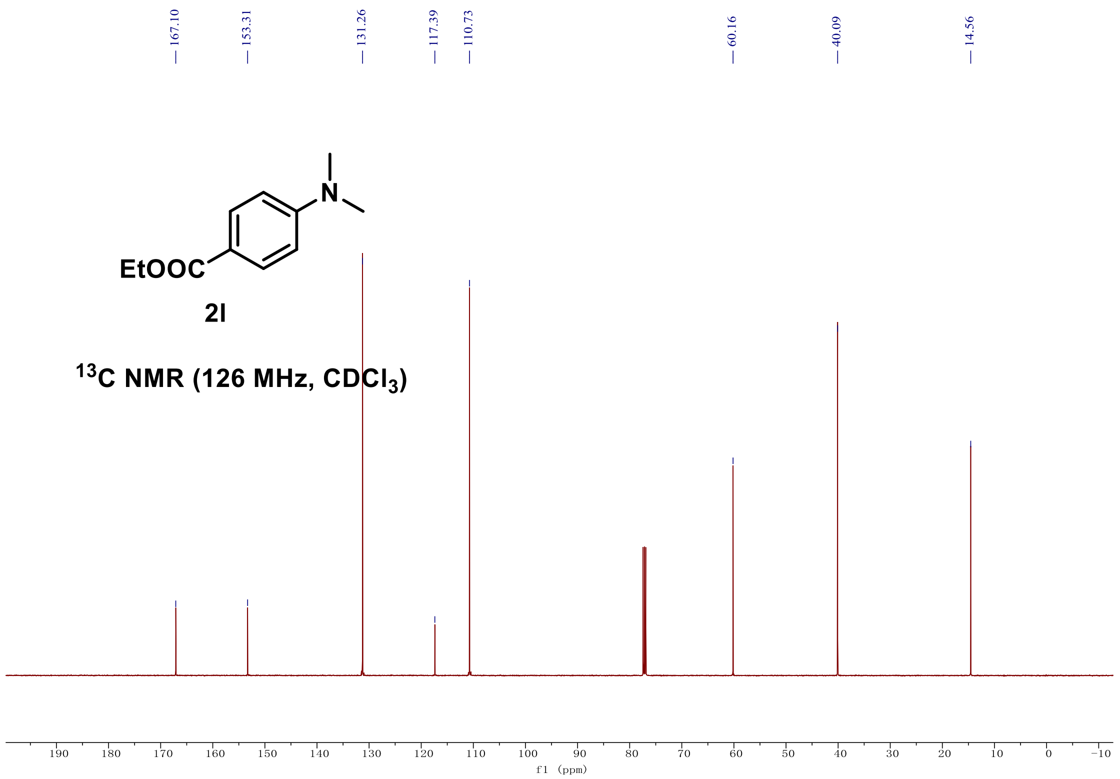
167.10
153.31
131.26
117.39
110.73
60.16
40.09
14.56
EtOOC
N
2l
13C NMR (126 MHz, CDCl3)
190 180 170 160 150 140 130 120 110 100 90 80 70 60 50 40 30 20 10 0 -10
f1 (ppm)

2m

$^{1}$H NMR (500 MHz, DMSO-d$_6$)

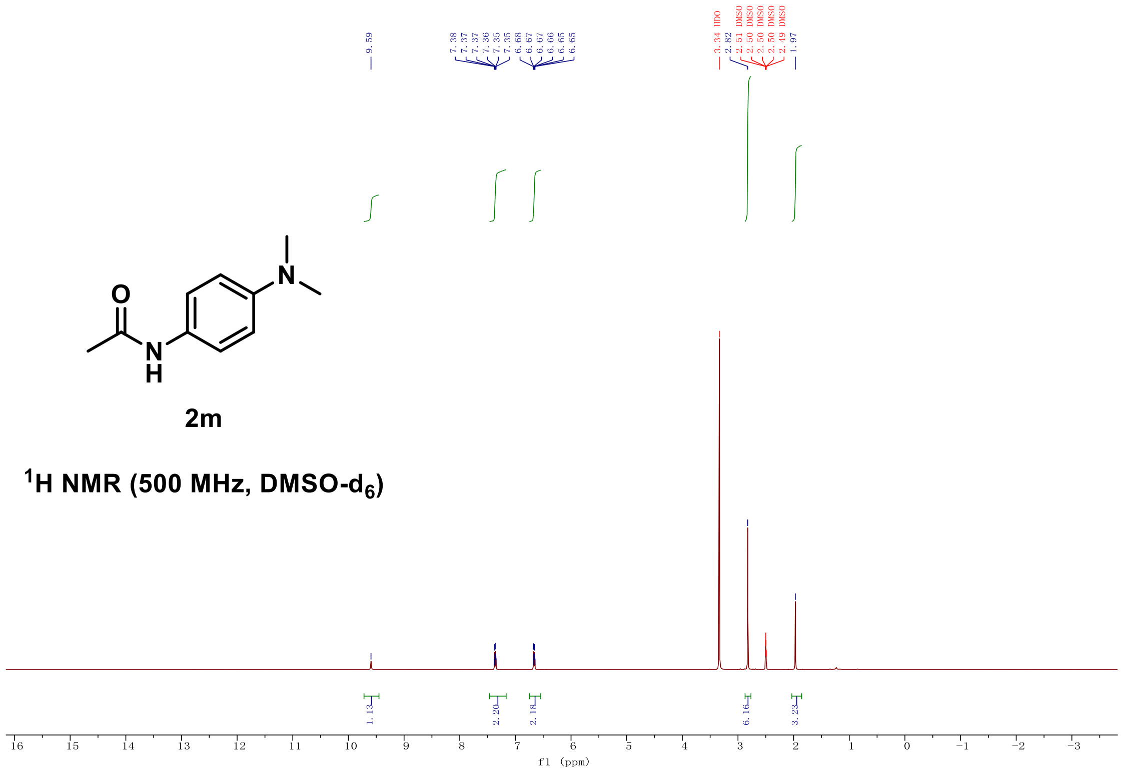


2m

$^{13}$C NMR (126 MHz, DMSO-d$_6$)

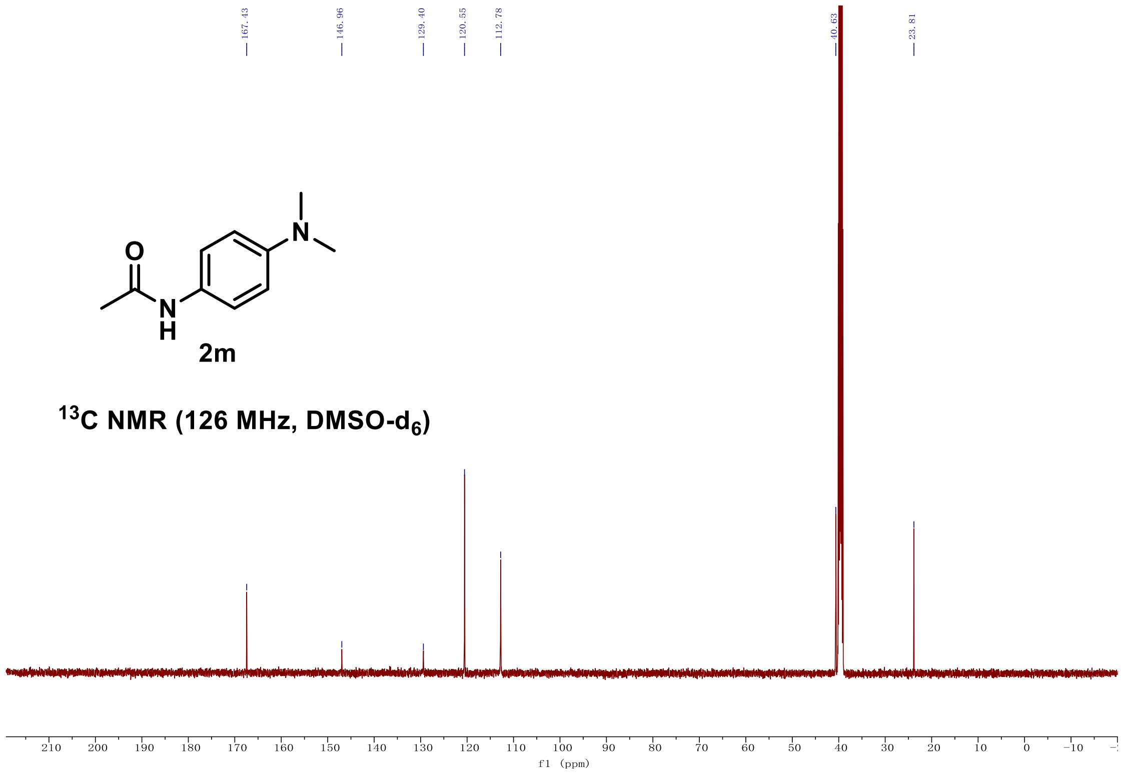

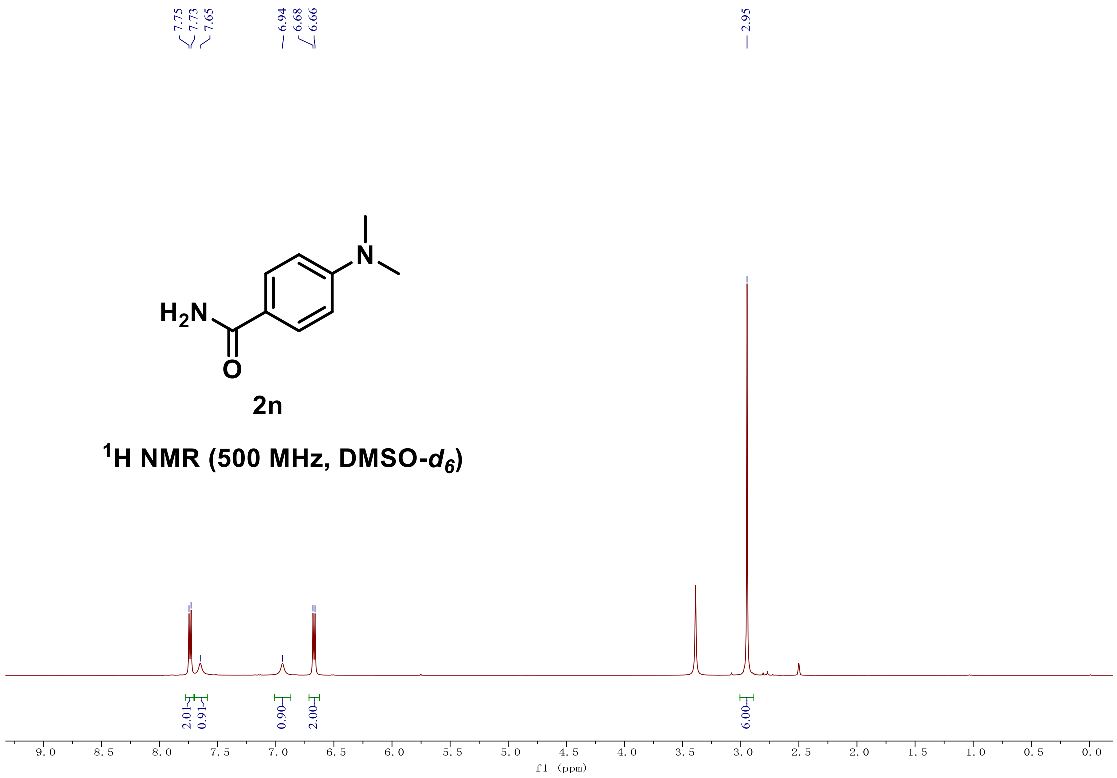
7.75
7.73
7.65
6.94
6.68
6.66
2.95
H2N
N
O
2n
1H NMR (500 MHz, DMSO-d6)
2.01
0.91
0.90
2.00
6.00
f1 (ppm)

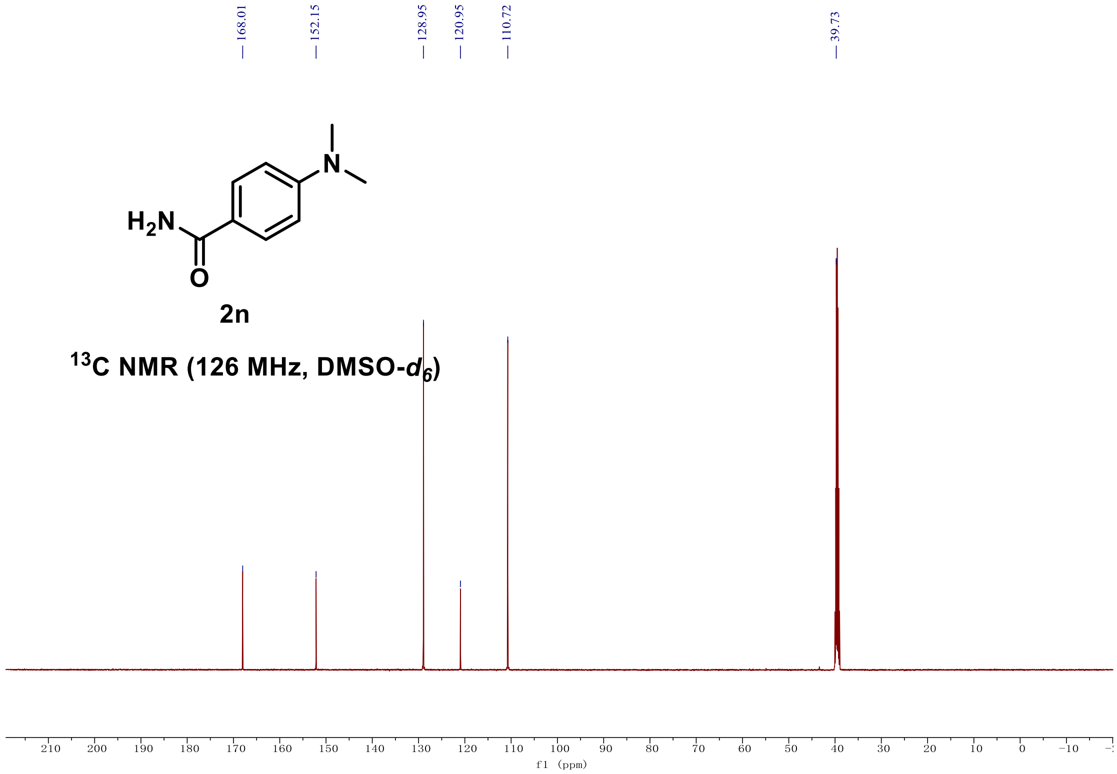
168.01
152.15
128.95
120.95
110.72
39.73
H2N
N
O
2n
13C NMR (126 MHz, DMSO-d6)
f1 (ppm)

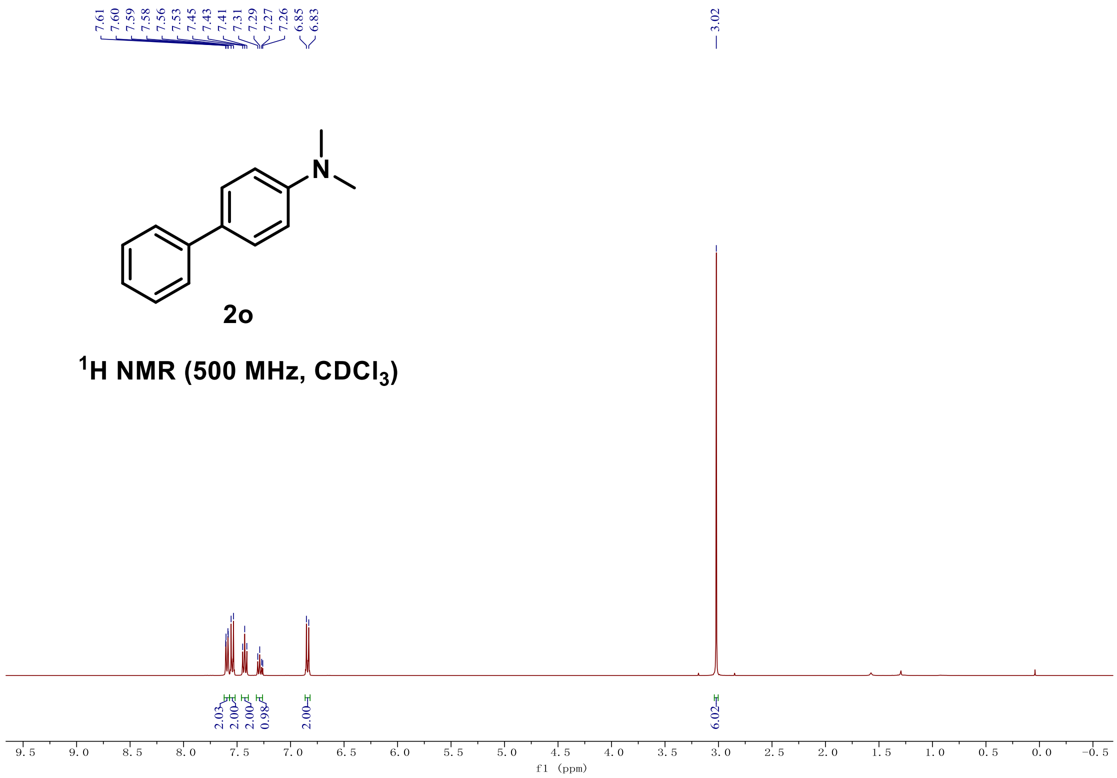


**2o**

$^{1}$H NMR (500 MHz, $CDCl_3$)

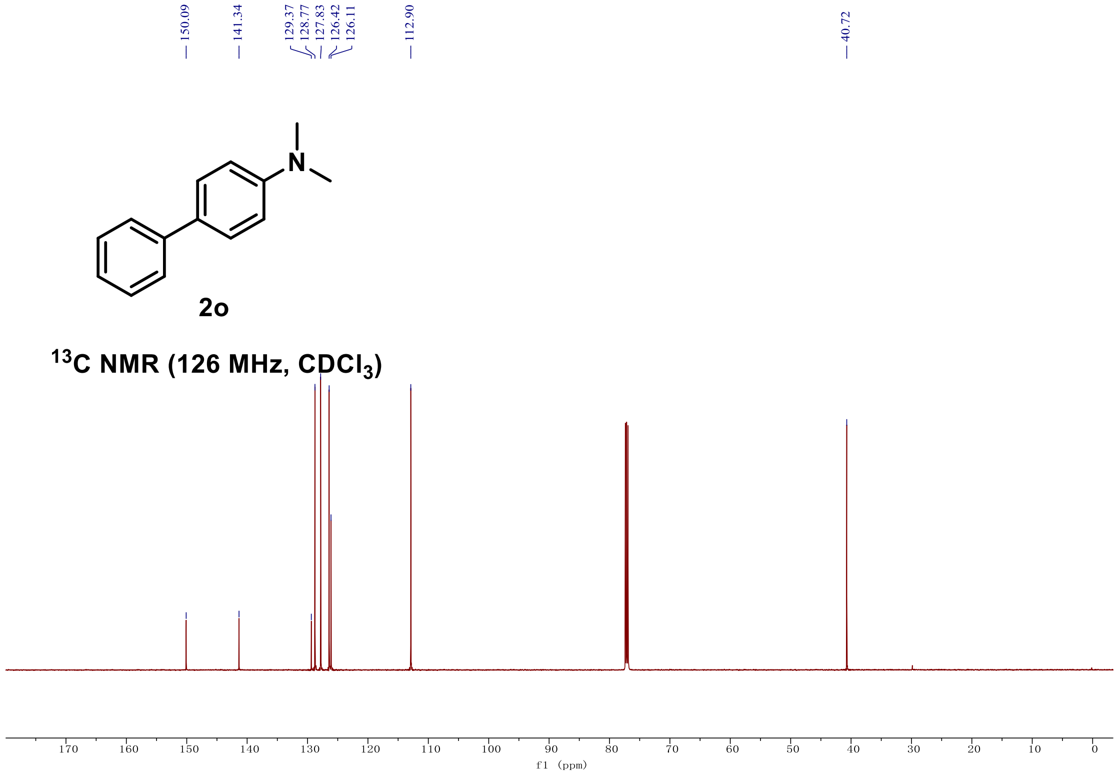


**2o**

$^{13}$C NMR (126 MHz, $CDCl_3$)

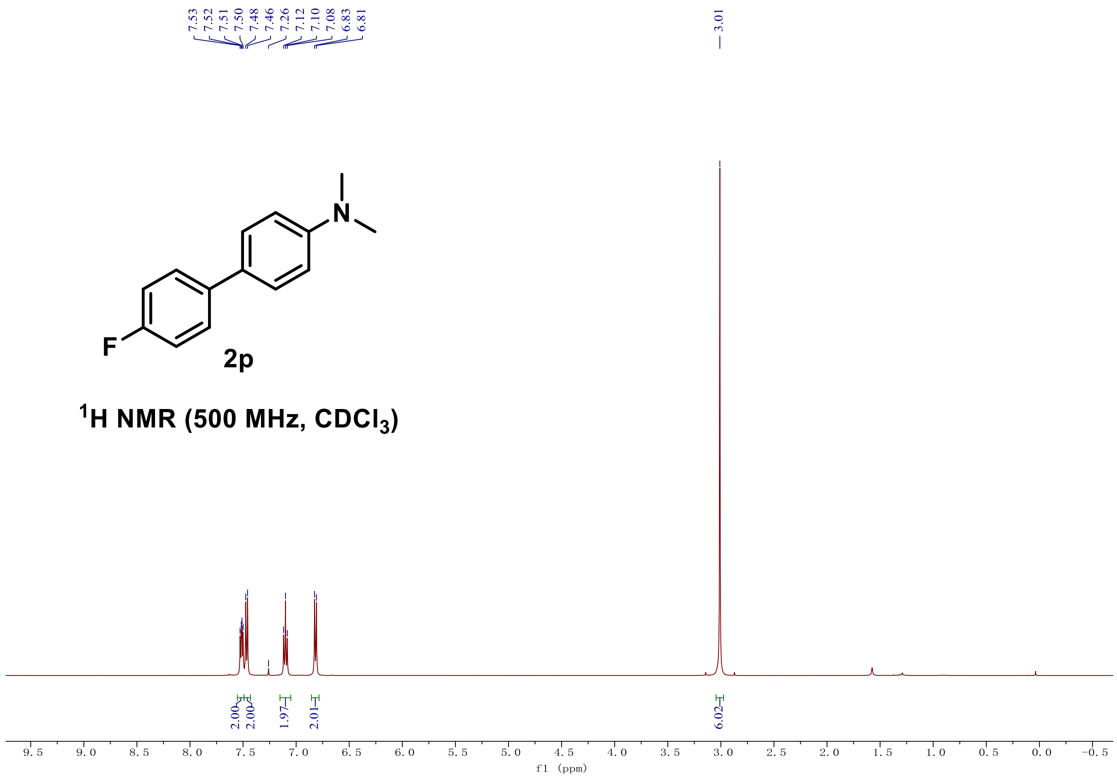
7.53
7.52
7.51
7.50
7.48
7.46
7.26
7.12
7.10
7.08
6.83
6.81
3.01
F
N
2p
1H NMR (500 MHz, CDCl3)
2.00
2.00
1.97
2.01
6.02
f1 (ppm)

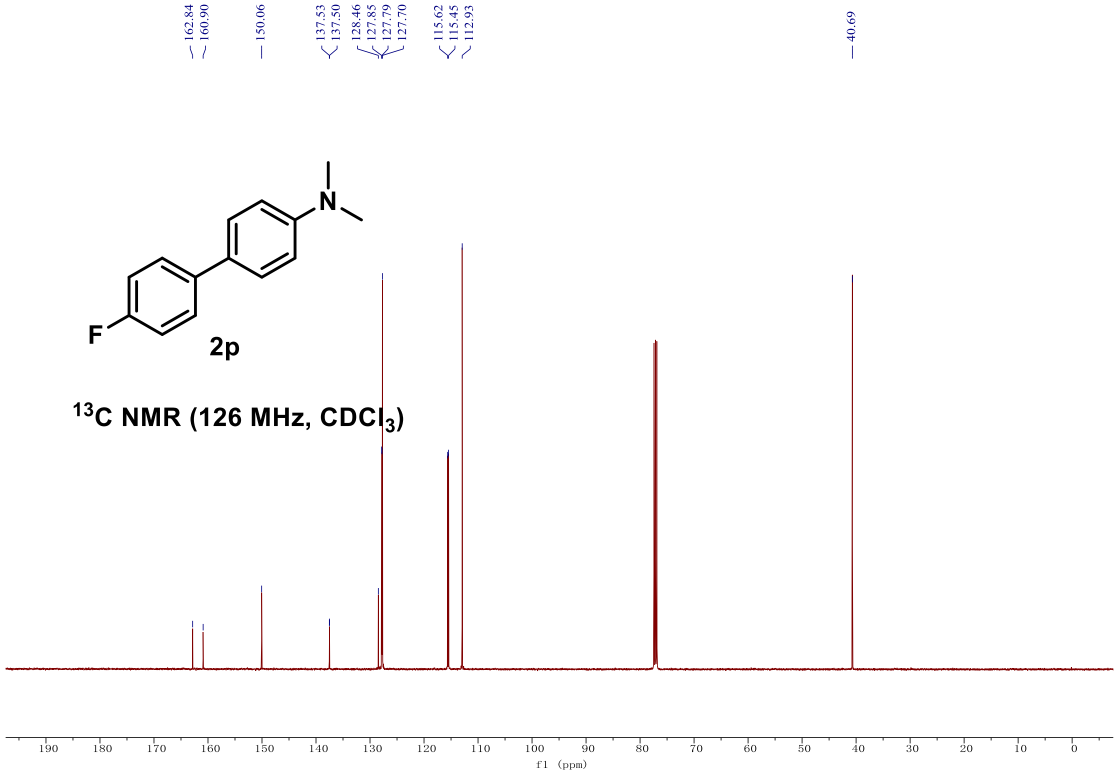
162.84
160.90
150.06
137.53
137.50
128.46
127.85
127.79
127.70
115.62
115.45
112.93
40.69
F
N
2p
13C NMR (126 MHz, CDCl3)
f1 (ppm)

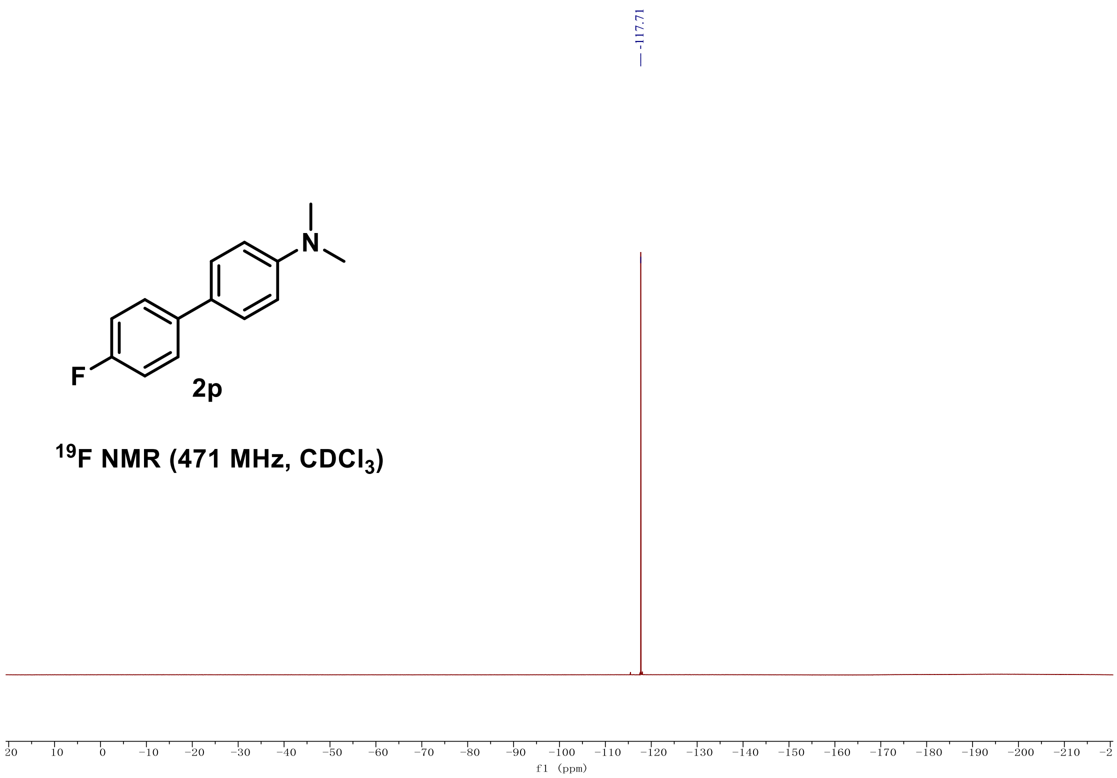

**2p**

**$^{19}$F NMR (471 MHz, $CDCl_3$)**

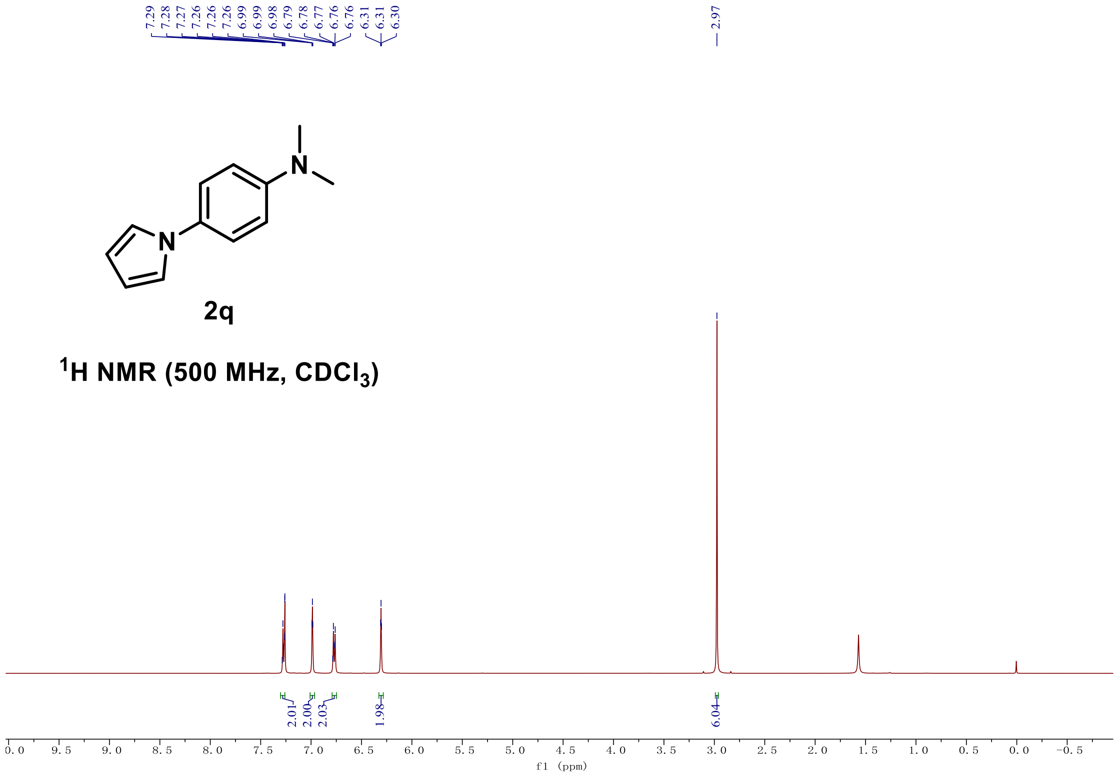

**2q**

**$^{1}$H NMR (500 MHz, $CDCl_3$)**

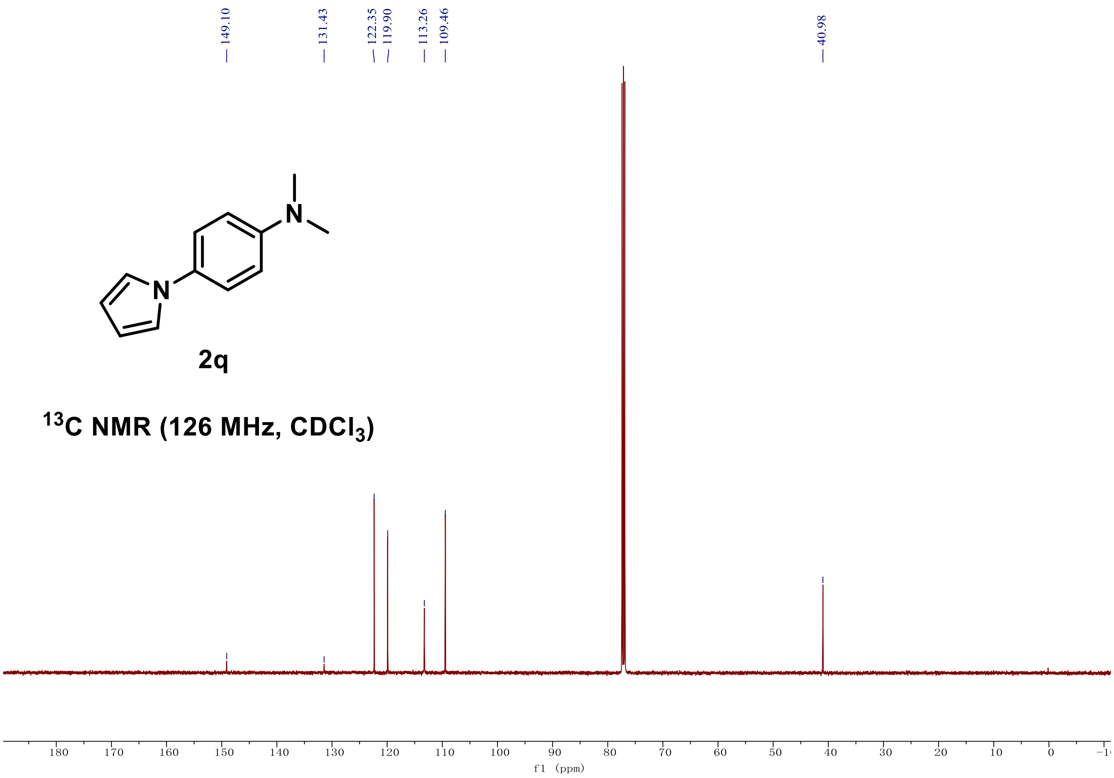
149.10
131.43
122.35
119.90
113.26
109.46
40.98
N
N
2q
$^{13}$C NMR (126 MHz, CDCl$_3$)
180 170 160 150 140 130 120 110 100 90 80 70 60 50 40 30 20 10 0
f1 (ppm)

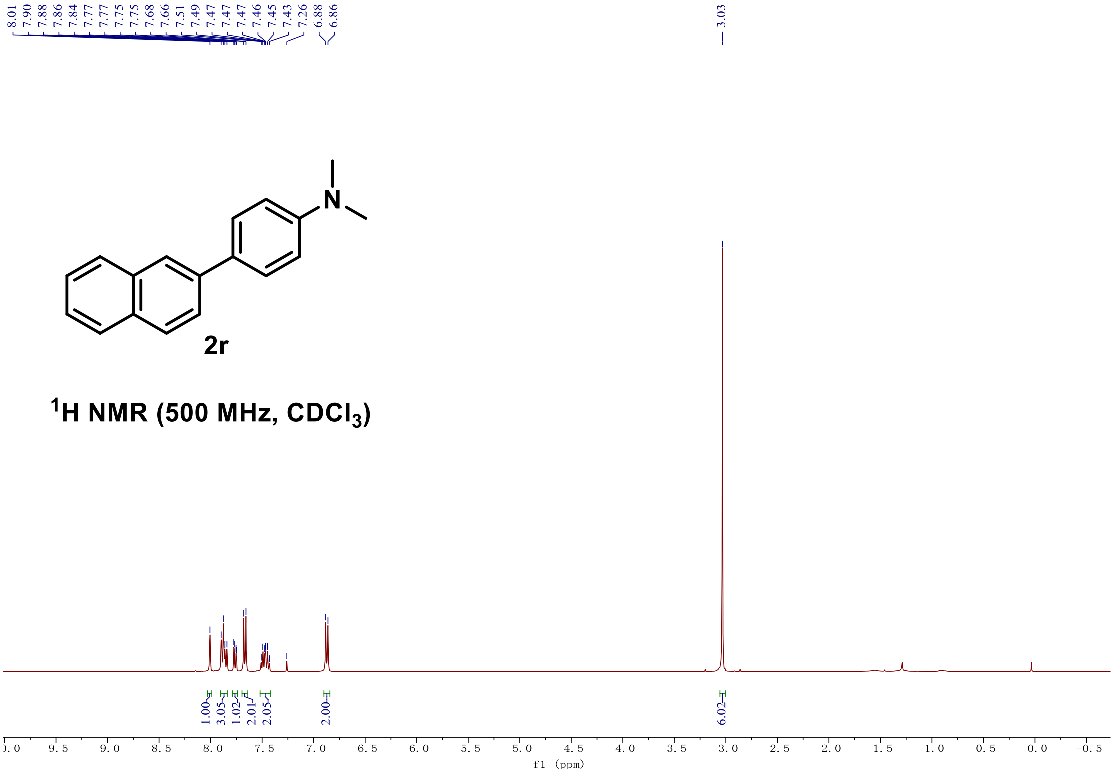
8.01 7.90 7.88 7.86 7.84 7.77 7.77 7.75 7.75 7.68 7.66 7.51 7.49 7.47 7.47 7.47 7.46 7.45 7.43 7.26 6.88 6.86
3.03
N
2r
$^{1}$H NMR (500 MHz, CDCl$_3$)
1.00 3.05 1.02 2.01 2.05 2.00
6.02
9.5 9.0 8.5 8.0 7.5 7.0 6.5 6.0 5.5 5.0 4.5 4.0 3.5 3.0 2.5 2.0 1.5 1.0 0.5 0.0 -0.5
f1 (ppm)

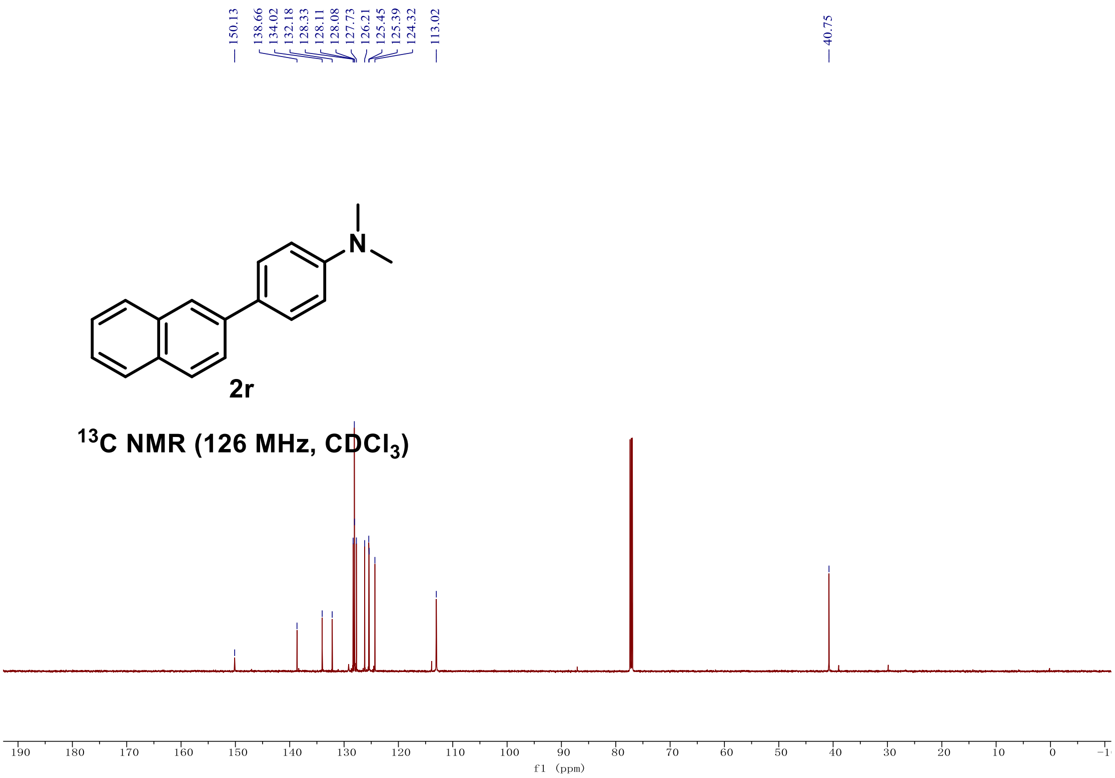
150.13
138.66
134.02
132.18
128.33
128.11
128.08
127.73
126.21
125.45
125.39
124.32
113.02
40.75
N
2r
13C NMR (126 MHz, CDCl3)
f1 (ppm)

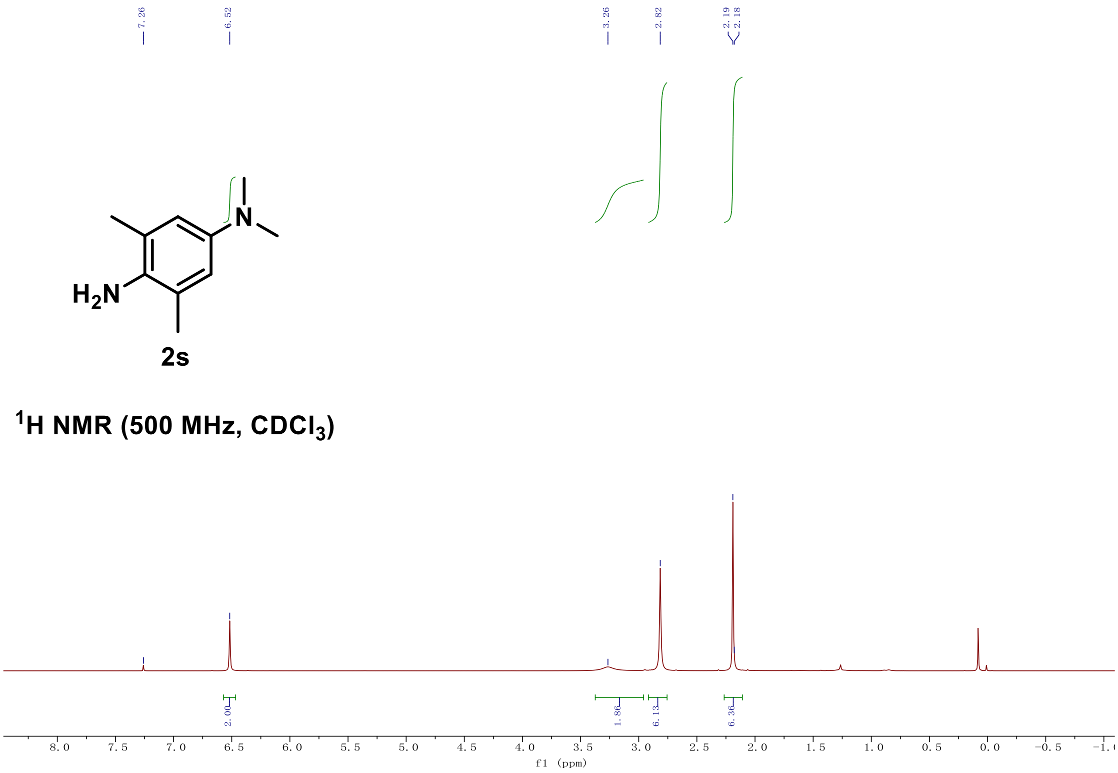
7.26
6.52
3.26
2.82
2.19
2.18
N
H2N
2s
1H NMR (500 MHz, CDCl3)
2.00
1.86
6.13
6.36
f1 (ppm)

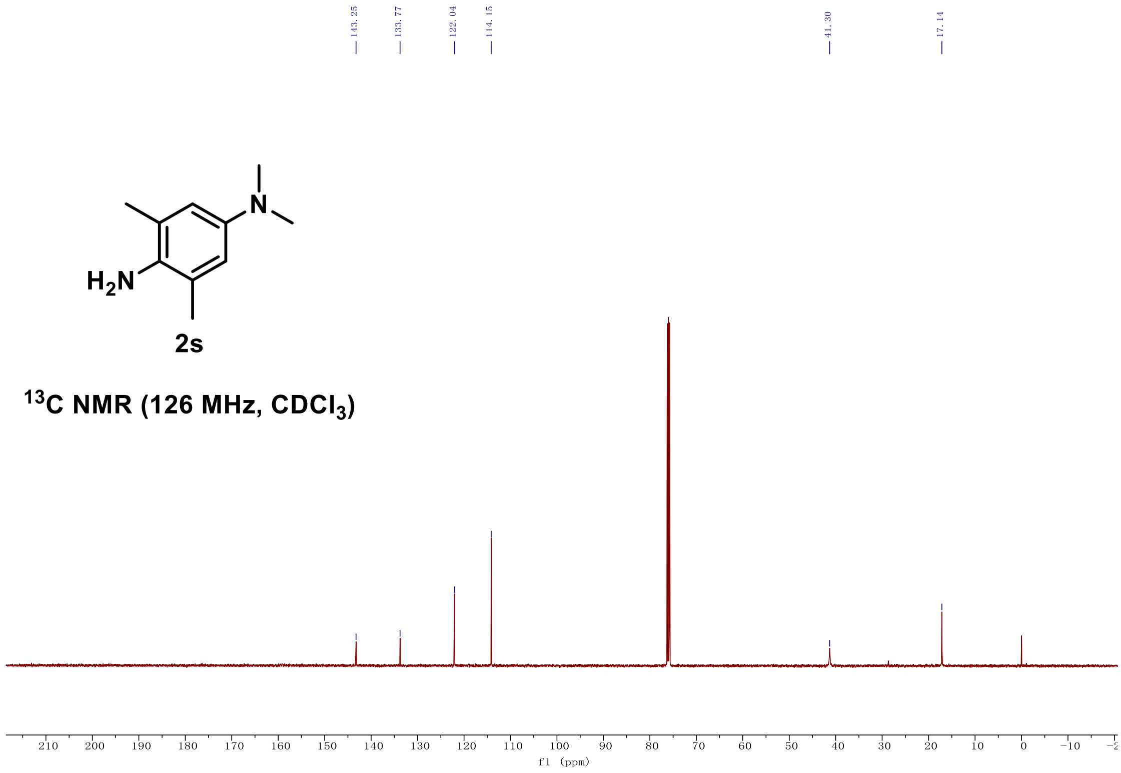
143.25
133.77
122.04
114.15
41.30
17.14
2s
$^{13}$C NMR (126 MHz, $CDCl_3$)
f1 (ppm)

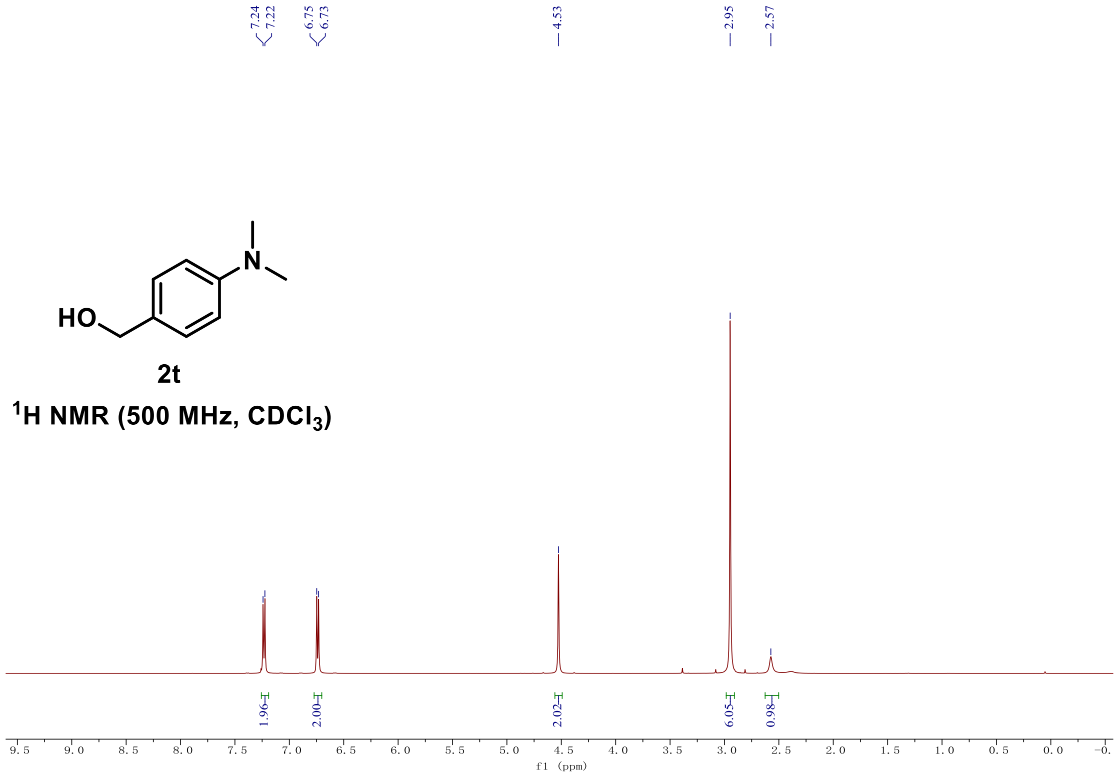
7.24
7.22
6.75
6.73
4.53
2.95
2.57
HO
N
2t
$^{1}$H NMR (500 MHz, $CDCl_3$)
1.96
2.00
2.02
6.05
0.98
f1 (ppm)

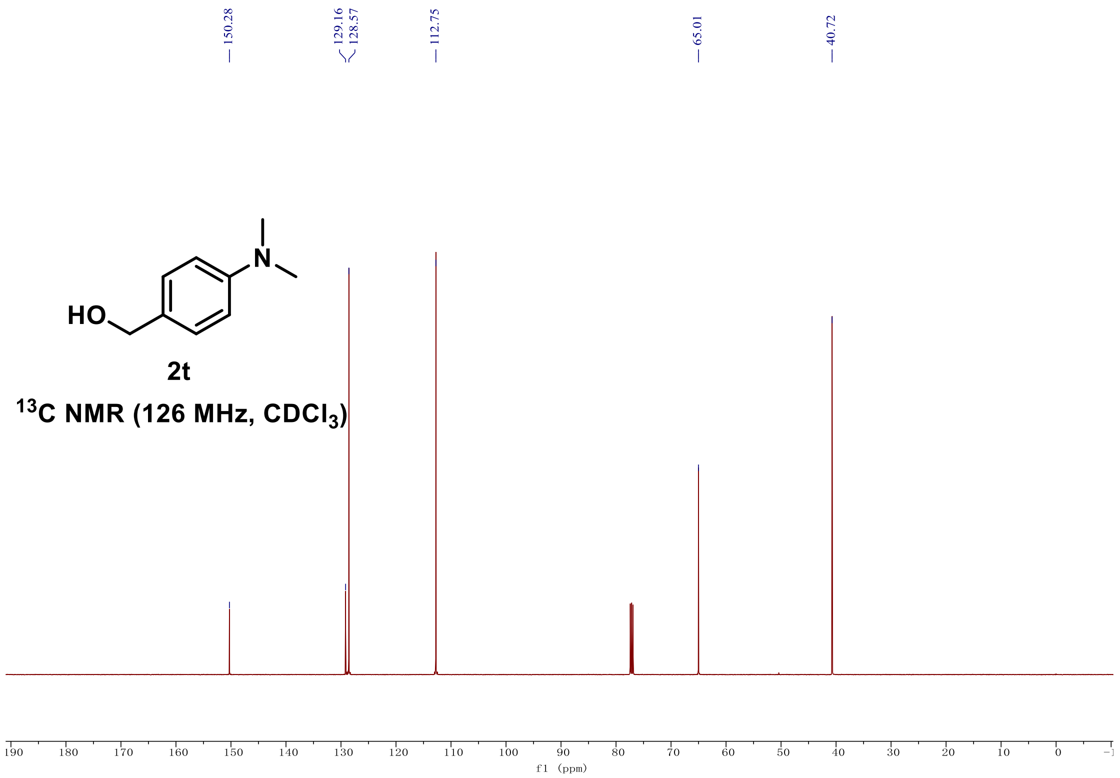
150.28
129.16
128.57
112.75
65.01
40.72
HO
N
2t
$^{13}$C NMR (126 MHz, $CDCl_3$)
f1 (ppm)

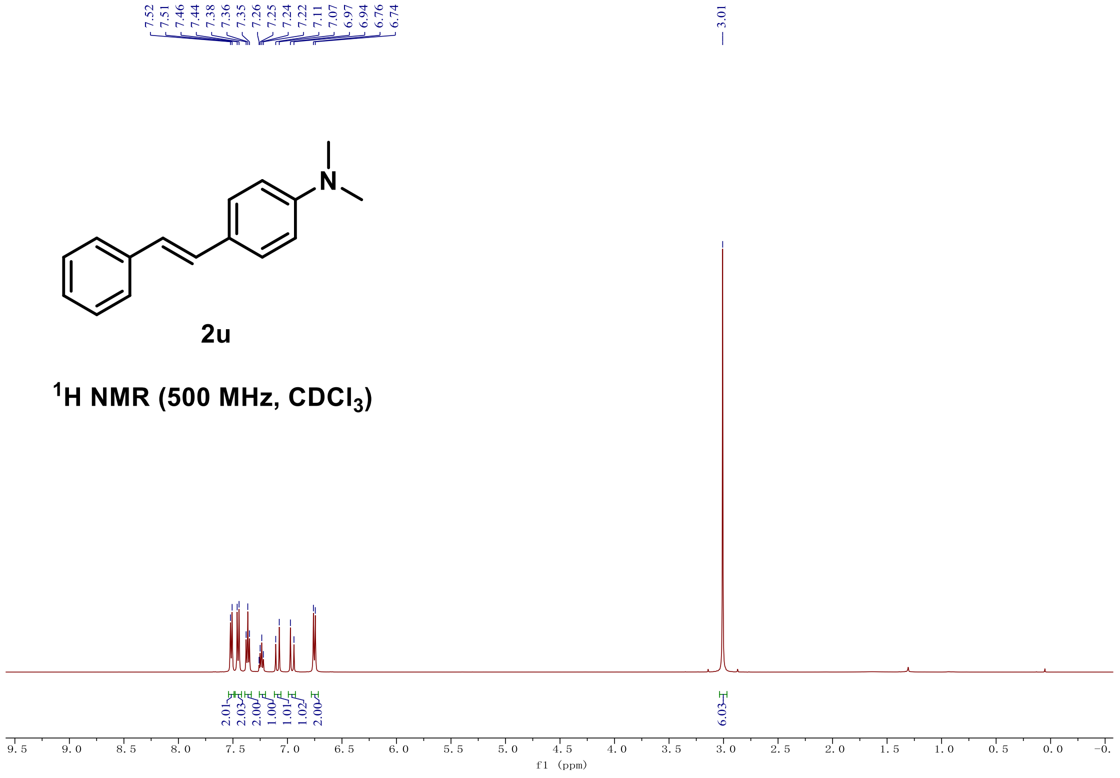
7.52
7.51
7.46
7.44
7.38
7.36
7.35
7.26
7.25
7.24
7.22
7.11
7.07
6.97
6.94
6.76
6.74
3.01
N
2u
$^{1}$H NMR (500 MHz, $CDCl_3$)
2.01
2.03
2.00
1.00
1.01
1.02
2.00
6.03
f1 (ppm)

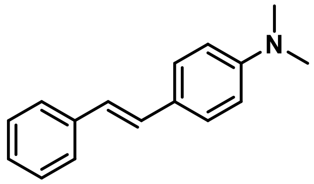


**2u**

**$^{13}$C NMR (126 MHz, CDCl$_3$)**

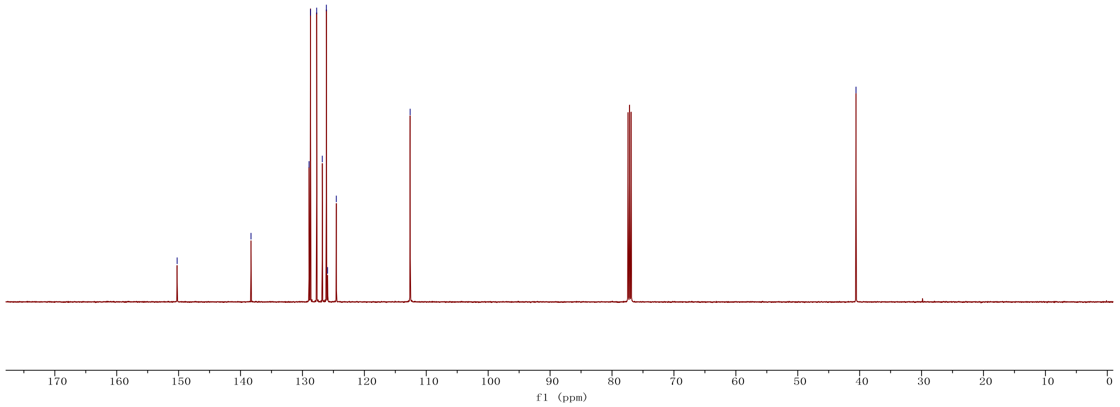


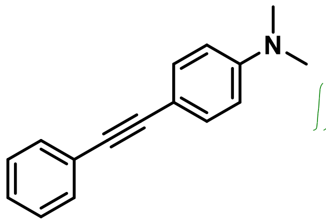


**2v**

**$^1$H NMR (500 MHz, CDCl$_3$)**

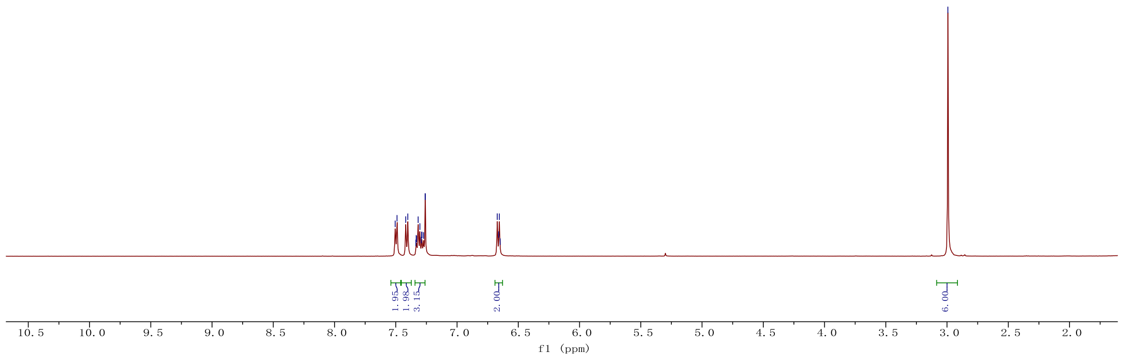

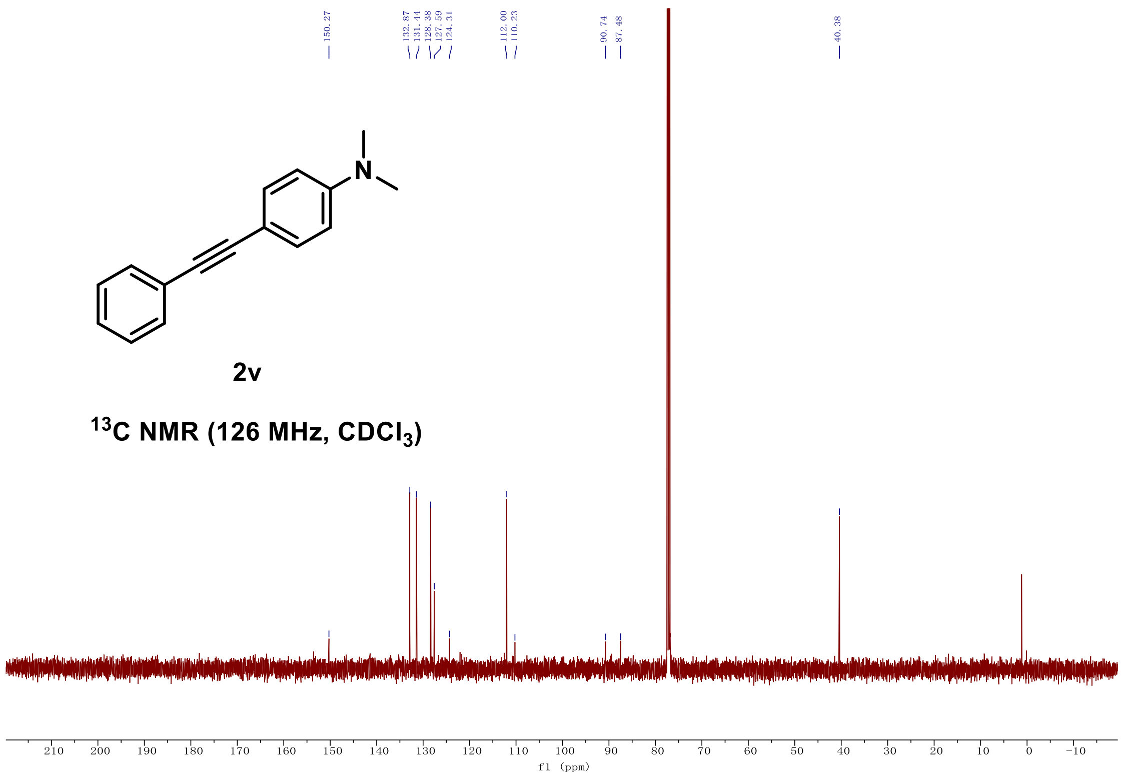
150.27
132.87
131.44
128.38
127.59
124.31
112.00
110.23
90.74
87.48
40.38
2v
$^{13}$C NMR (126 MHz, CDCl$_3$)
210 200 190 180 170 160 150 140 130 120 110 100 90 80 70 60 50 40 30 20 10 0 −10
f1 (ppm)

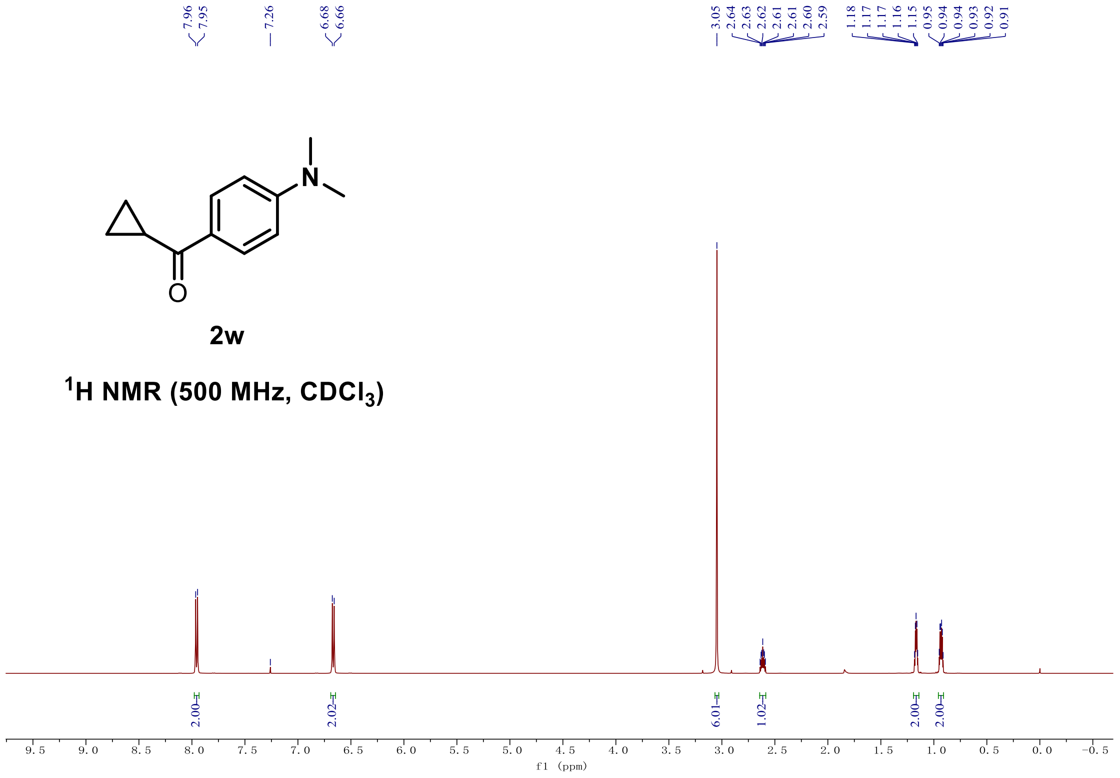
7.96
7.95
7.26
6.68
6.66
3.05
2.64
2.63
2.62
2.61
2.61
2.60
2.59
1.18
1.17
1.17
1.16
1.15
0.95
0.94
0.94
0.93
0.92
0.91
O
N
2w
$^{1}$H NMR (500 MHz, CDCl$_3$)
2.00
2.02
6.01
1.02
2.00
2.00
9.5 9.0 8.5 8.0 7.5 7.0 6.5 6.0 5.5 5.0 4.5 4.0 3.5 3.0 2.5 2.0 1.5 1.0 0.5 0.0 −0.5
f1 (ppm)

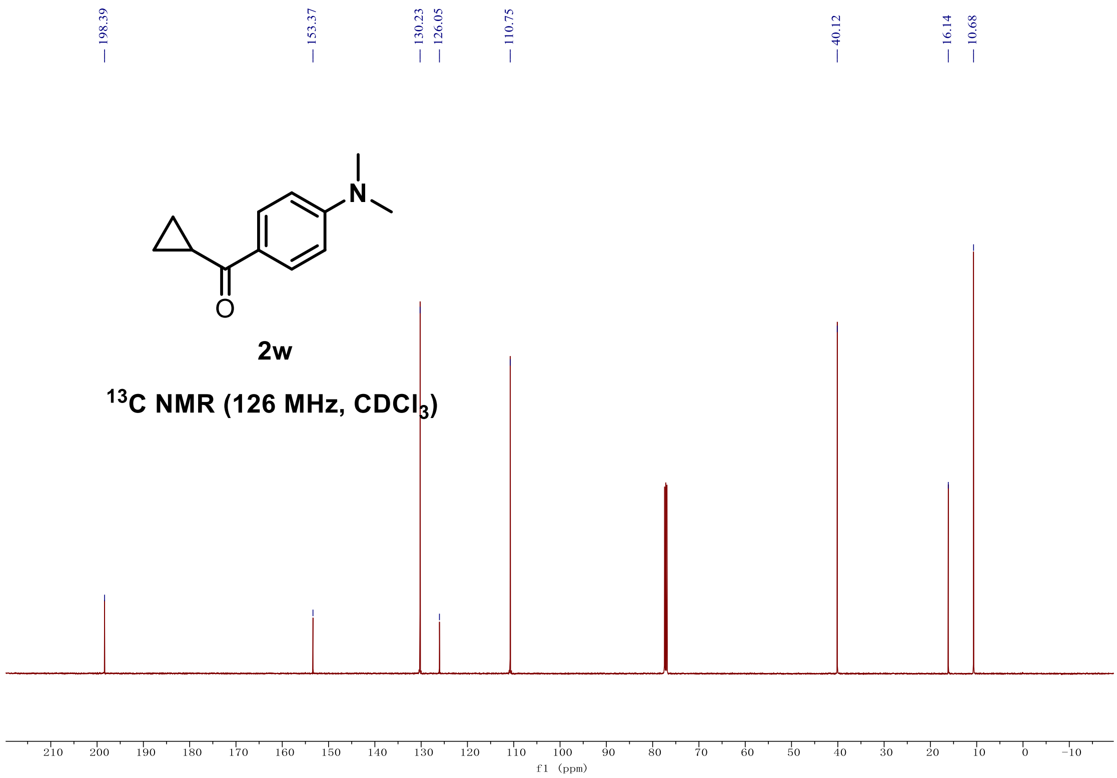
198.39
153.37
130.23
126.05
110.75
40.12
16.14
10.68
N
O
2w
$^{13}$C NMR (126 MHz, CDCl$_3$)
f1 (ppm)

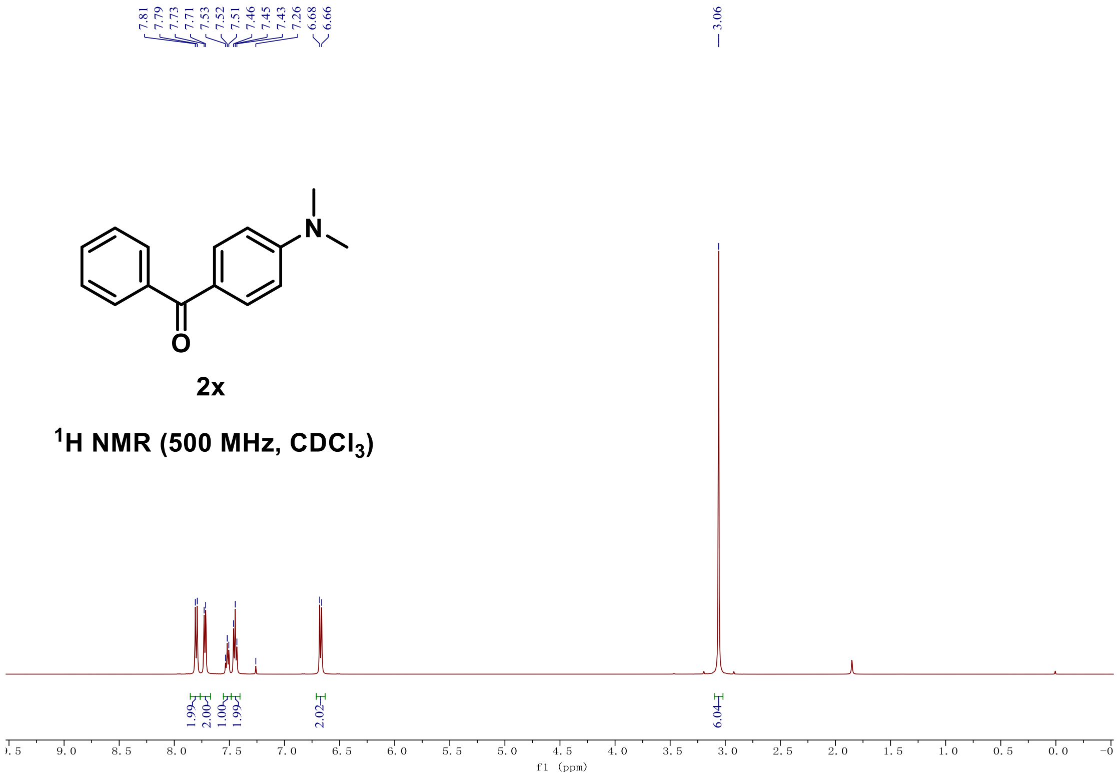
7.81
7.79
7.73
7.71
7.53
7.52
7.51
7.46
7.45
7.43
7.26
6.68
6.66
3.06
N
O
2x
$^{1}$H NMR (500 MHz, CDCl$_3$)
1.99
2.00
1.00
1.99
2.02
6.04
f1 (ppm)

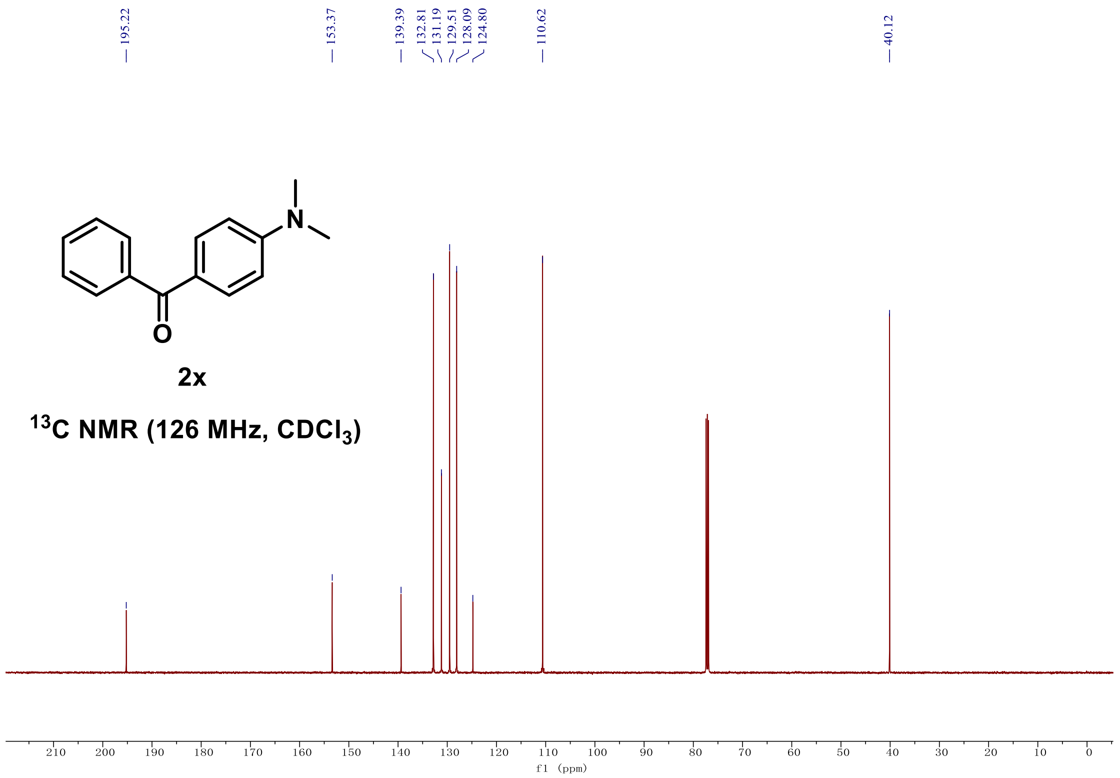
195.22
153.37
139.39
132.81
131.19
129.51
128.09
124.80
110.62
40.12
N
O
2x
$^{13}$C NMR (126 MHz, CDCl$_3$)
210 200 190 180 170 160 150 140 130 120 110 100 90 80 70 60 50 40 30 20 10 0
f1 (ppm)

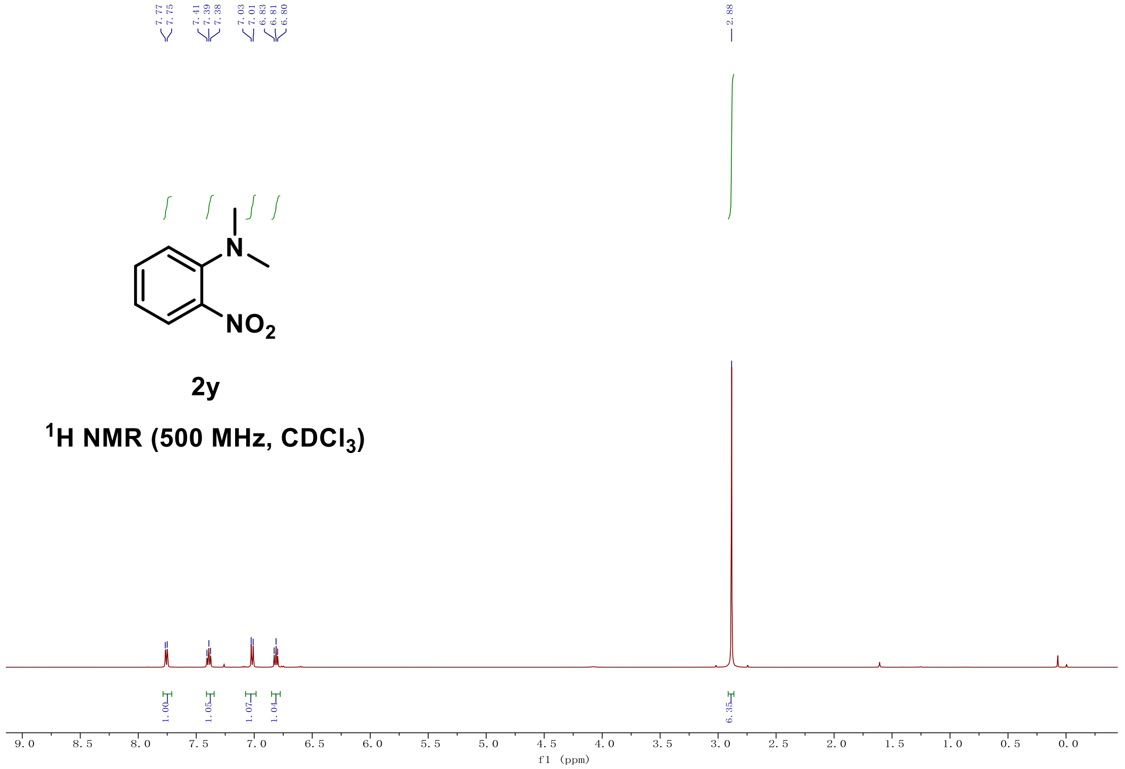
7.77
7.75
7.41
7.39
7.38
7.03
7.01
6.83
6.81
6.80
2.88
N
NO$_2$
2y
$^1$H NMR (500 MHz, CDCl$_3$)
1.00
1.05
1.07
1.04
6.35
9.0 8.5 8.0 7.5 7.0 6.5 6.0 5.5 5.0 4.5 4.0 3.5 3.0 2.5 2.0 1.5 1.0 0.5 0.0
f1 (ppm)

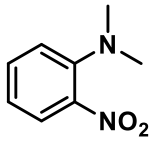

**2y**

**$^{13}$C NMR (126 MHz, CDCl$_3$)**

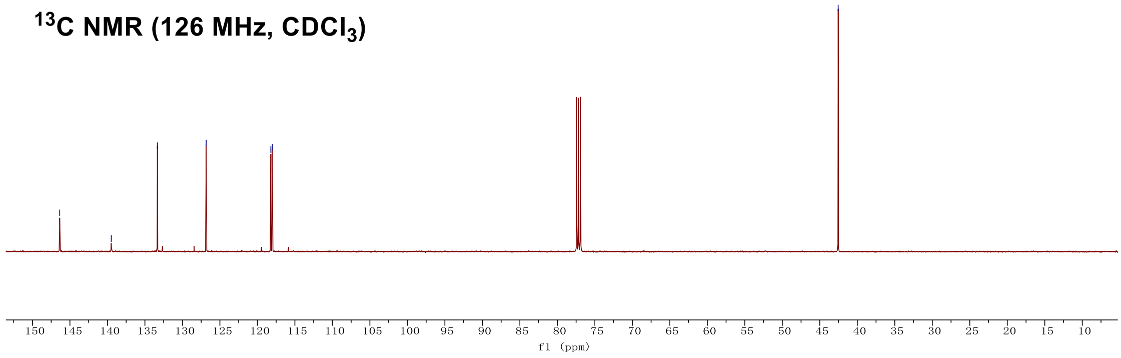


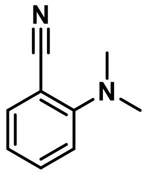

**2z**

**$^{1}$H NMR (500 MHz, CDCl$_3$)**

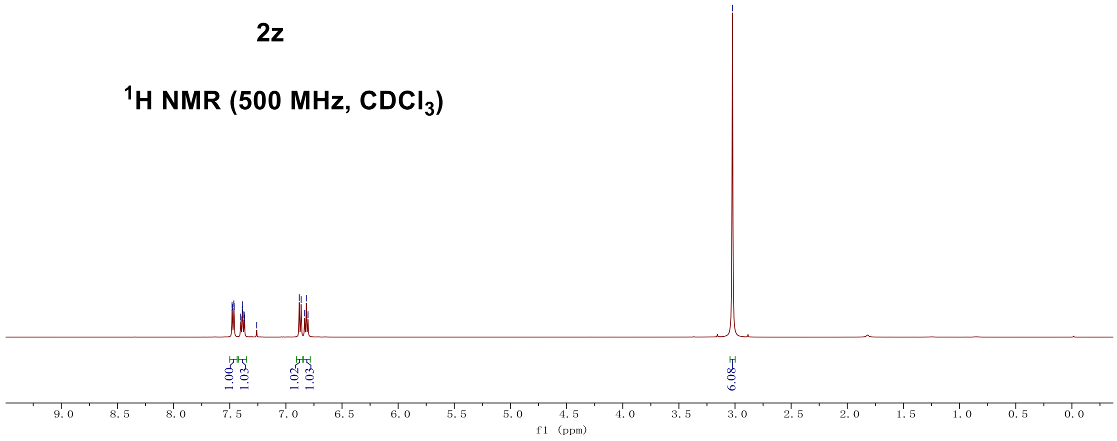

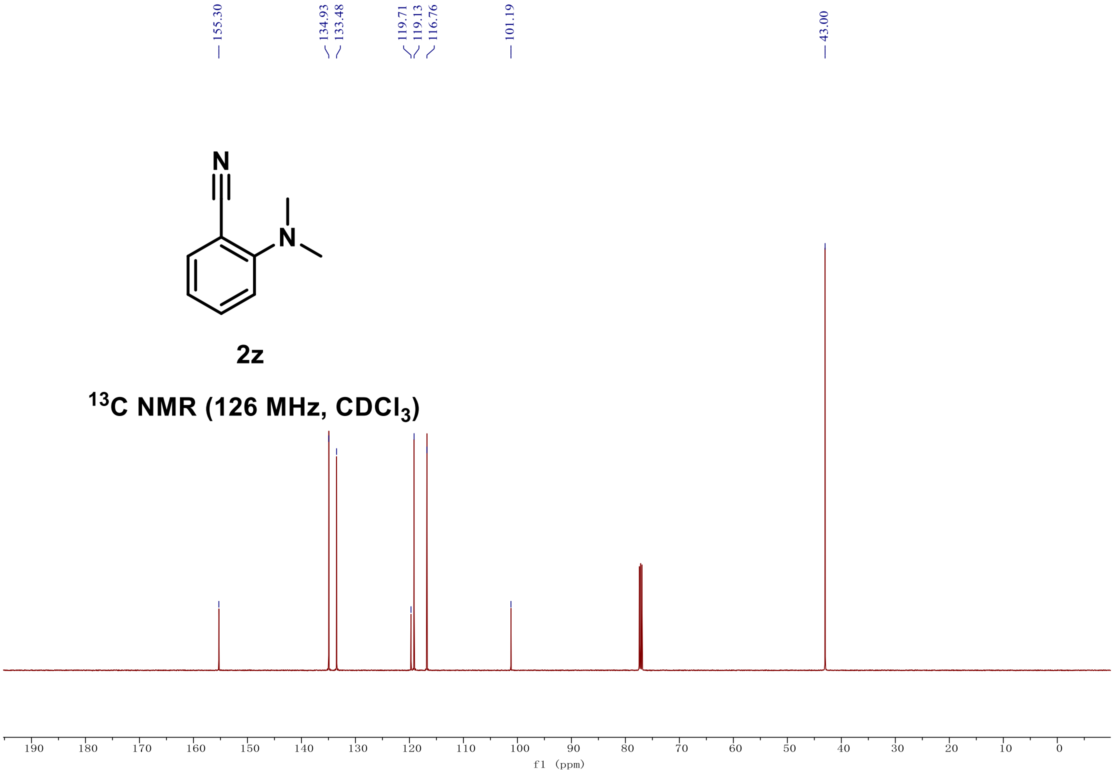


2z

$^{13}$C NMR (126 MHz, $CDCl_3$)

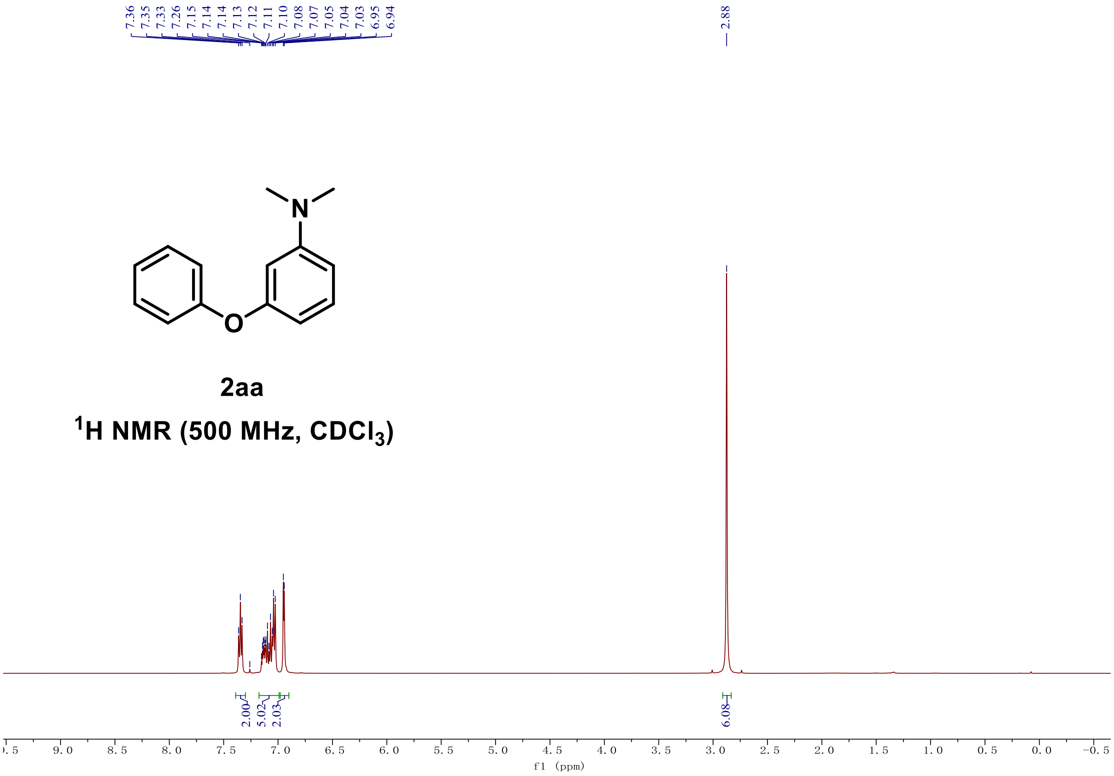


2aa

$^{1}$H NMR (500 MHz, $CDCl_3$)

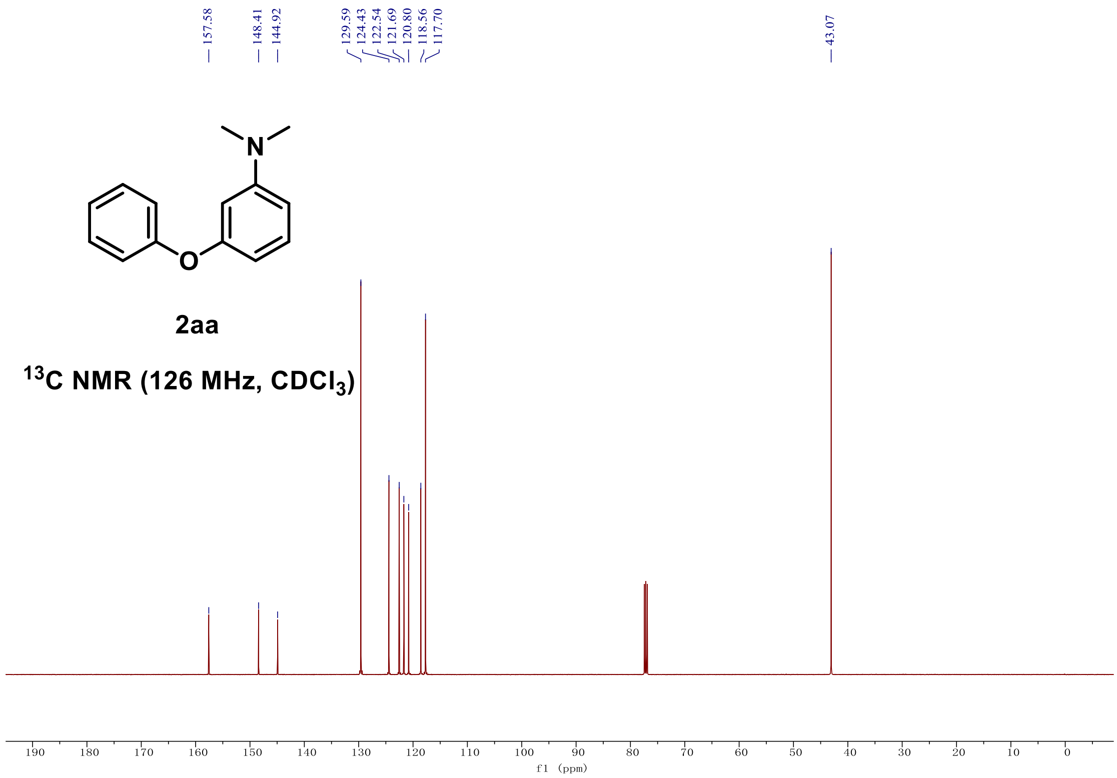
157.58
148.41
144.92
129.59
124.43
122.54
121.69
120.80
118.56
117.70
43.07
2aa
$^{13}$C NMR (126 MHz, CDCl$_3$)
190 180 170 160 150 140 130 120 110 100 90 80 70 60 50 40 30 20 10 0
f1 (ppm)

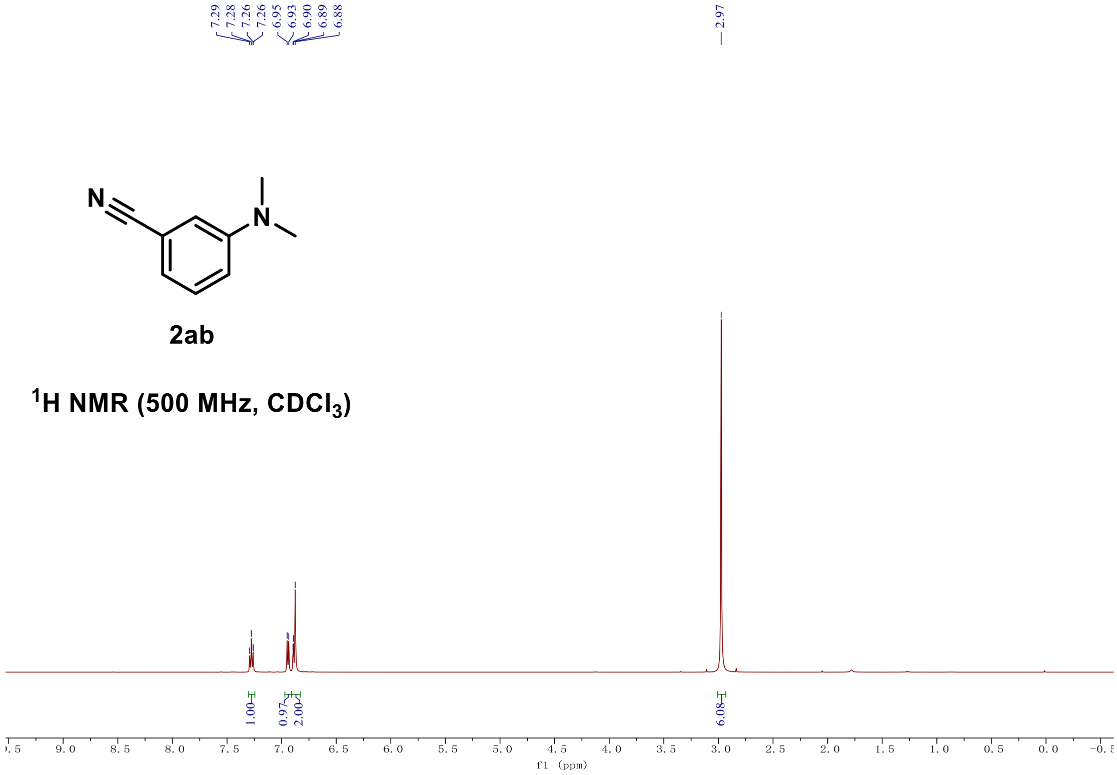
7.29
7.28
7.26
7.26
6.95
6.93
6.90
6.89
6.88
2.97
2ab
$^1$H NMR (500 MHz, CDCl$_3$)
1.00
0.97
2.00
6.08
9.0 8.5 8.0 7.5 7.0 6.5 6.0 5.5 5.0 4.5 4.0 3.5 3.0 2.5 2.0 1.5 1.0 0.5 0.0
f1 (ppm)

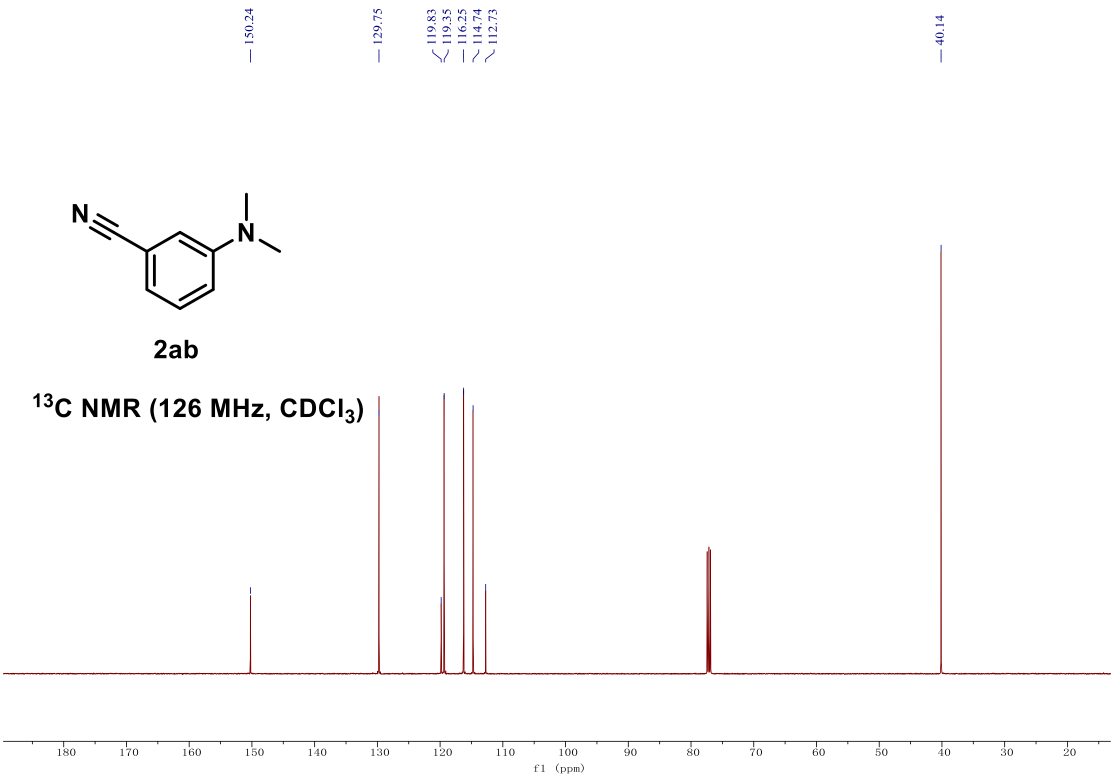
150.24
129.75
119.83
119.35
116.25
114.74
112.73
40.14
N
N
2ab
$^{13}$C NMR (126 MHz, CDCl$_3$)
180 170 160 150 140 130 120 110 100 90 80 70 60 50 40 30 20
f1 (ppm)

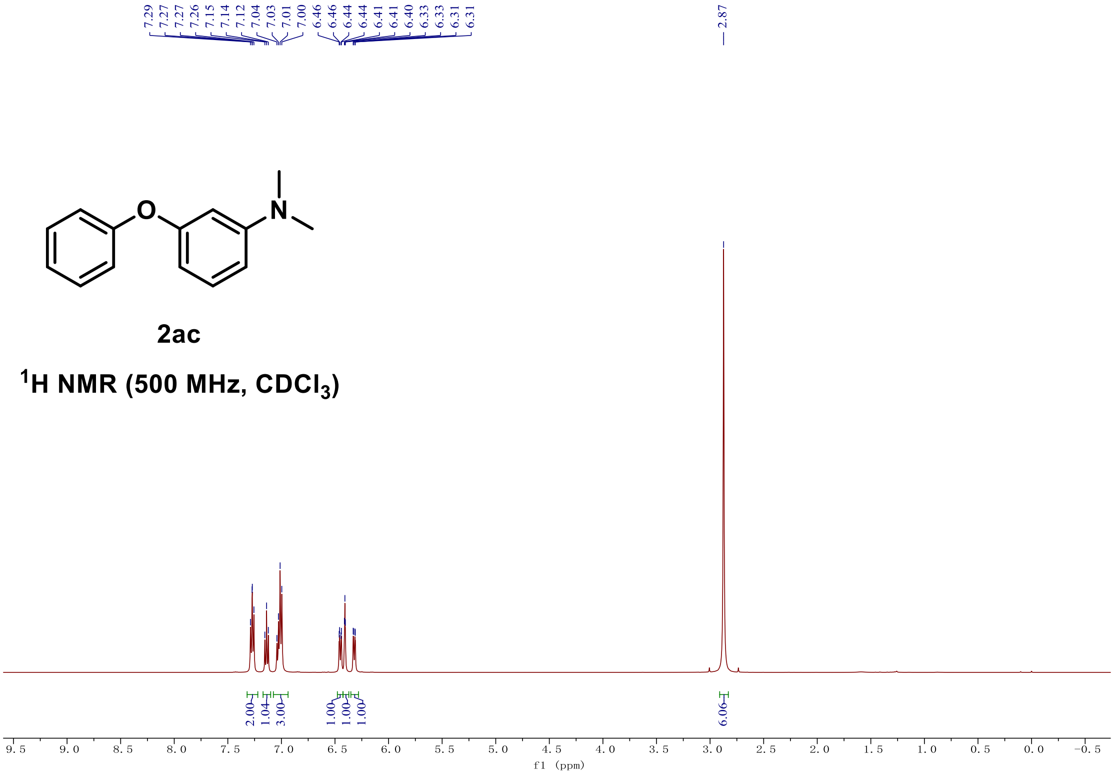
7.29
7.27
7.27
7.26
7.15
7.14
7.12
7.04
7.03
7.01
7.00
6.46
6.46
6.44
6.44
6.41
6.41
6.40
6.33
6.33
6.31
6.31
2.87
O
N
2ac
$^{1}$H NMR (500 MHz, CDCl$_3$)
2.00
1.04
3.00
1.00
1.00
1.00
6.06
9.5 9.0 8.5 8.0 7.5 7.0 6.5 6.0 5.5 5.0 4.5 4.0 3.5 3.0 2.5 2.0 1.5 1.0 0.5 0.0 -0.5
f1 (ppm)

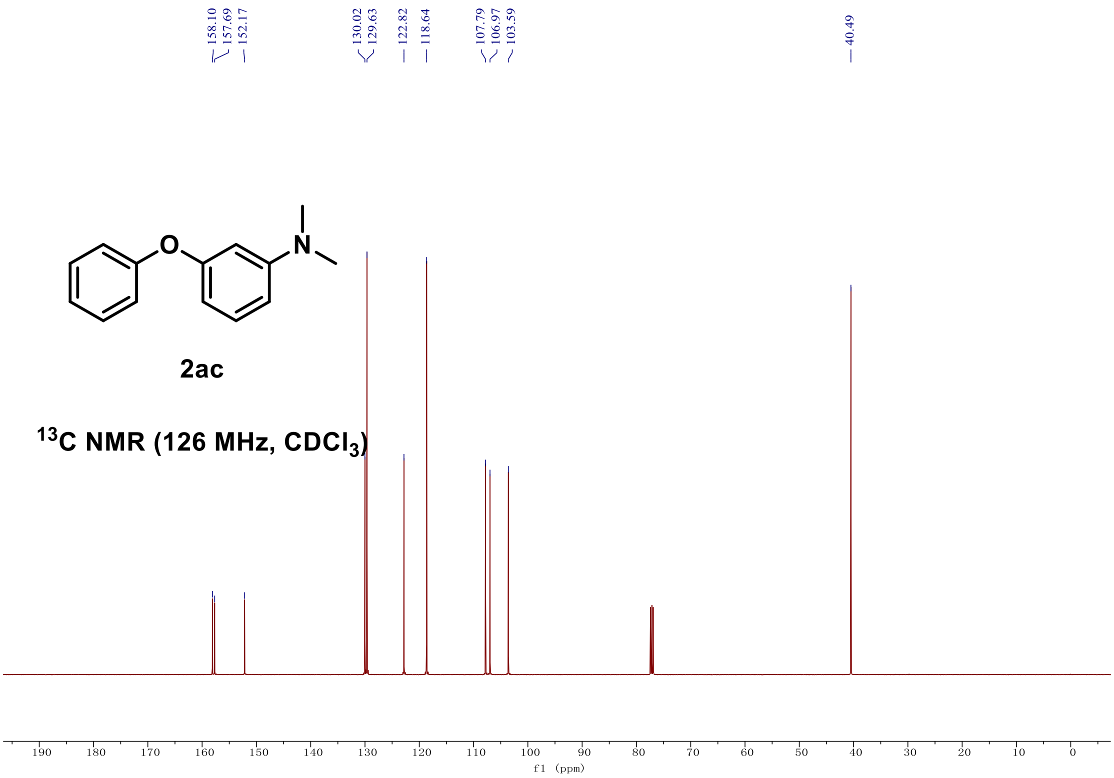


**2ac**

**$^{13}$C NMR (126 MHz, CDCl$_3$)**

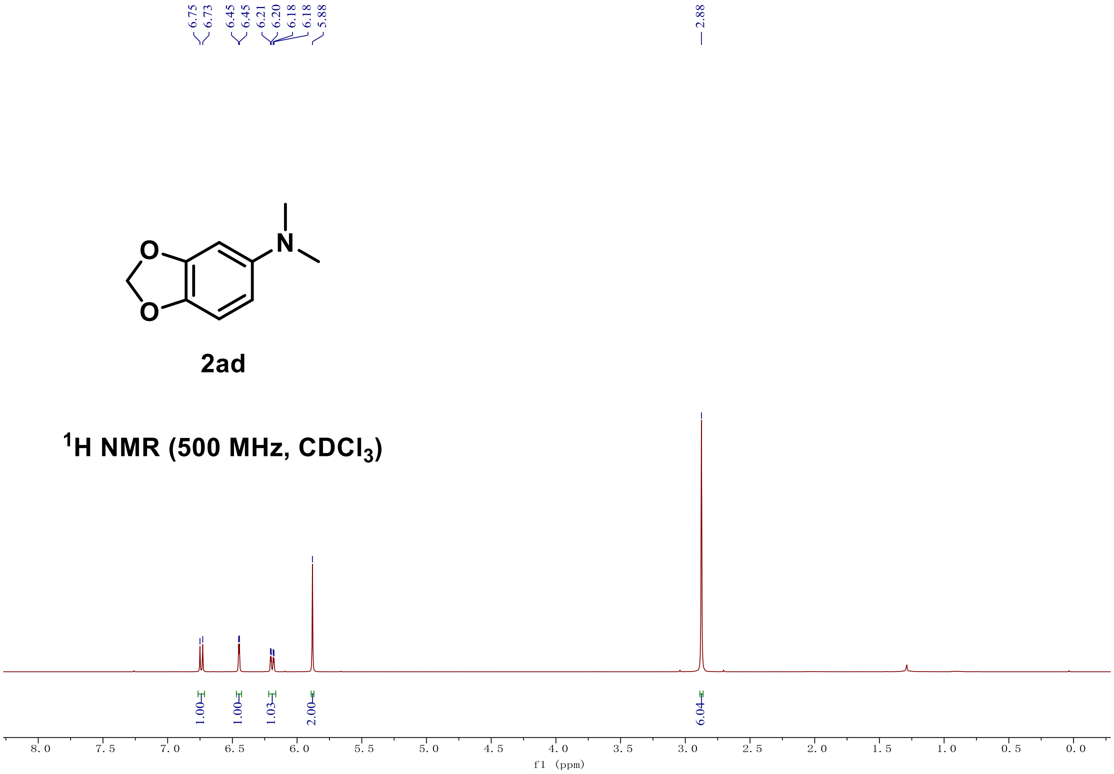


**2ad**

**$^{1}$H NMR (500 MHz, CDCl$_3$)**

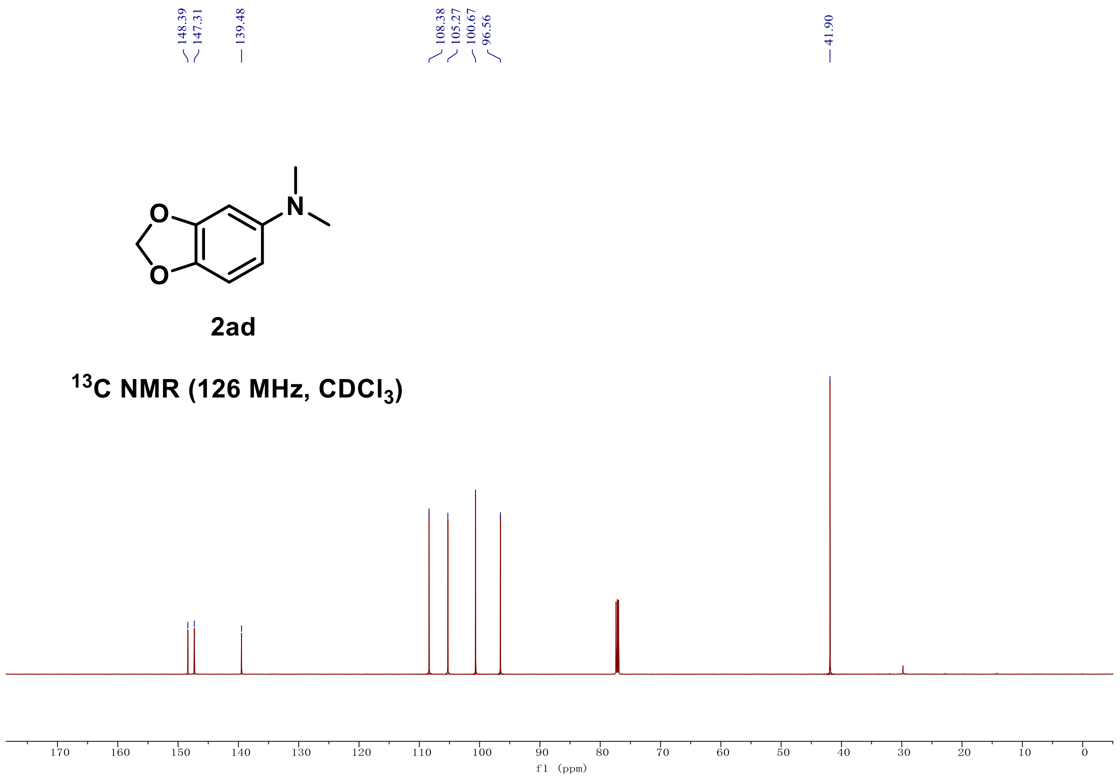
148.39
147.31
139.48
108.38
105.27
100.67
96.56
41.90
2ad
$^{13}$C NMR (126 MHz, $CDCl_3$)
f1 (ppm)

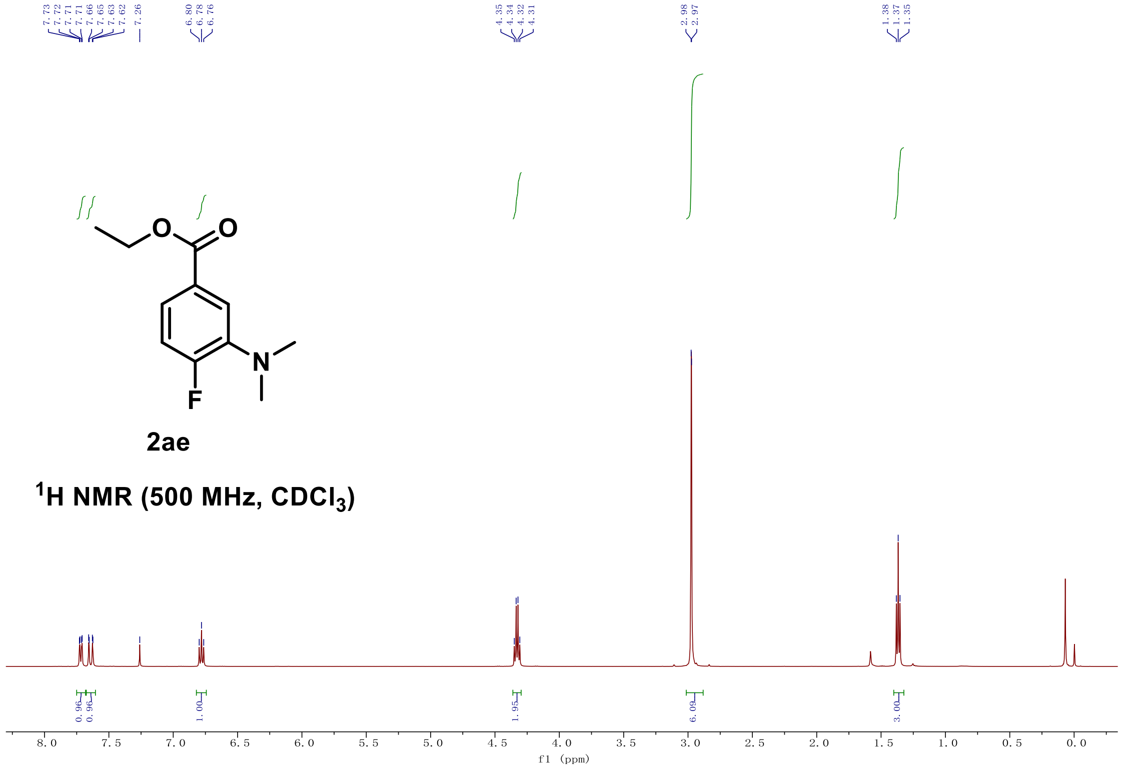
7.73
7.72
7.71
7.71
7.66
7.65
7.63
7.62
7.26
6.80
6.78
6.76
4.35
4.34
4.32
4.31
2.98
2.97
1.38
1.37
1.35
2ae
$^{1}$H NMR (500 MHz, $CDCl_3$)
0.96
0.96
1.00
1.95
6.09
3.00
f1 (ppm)

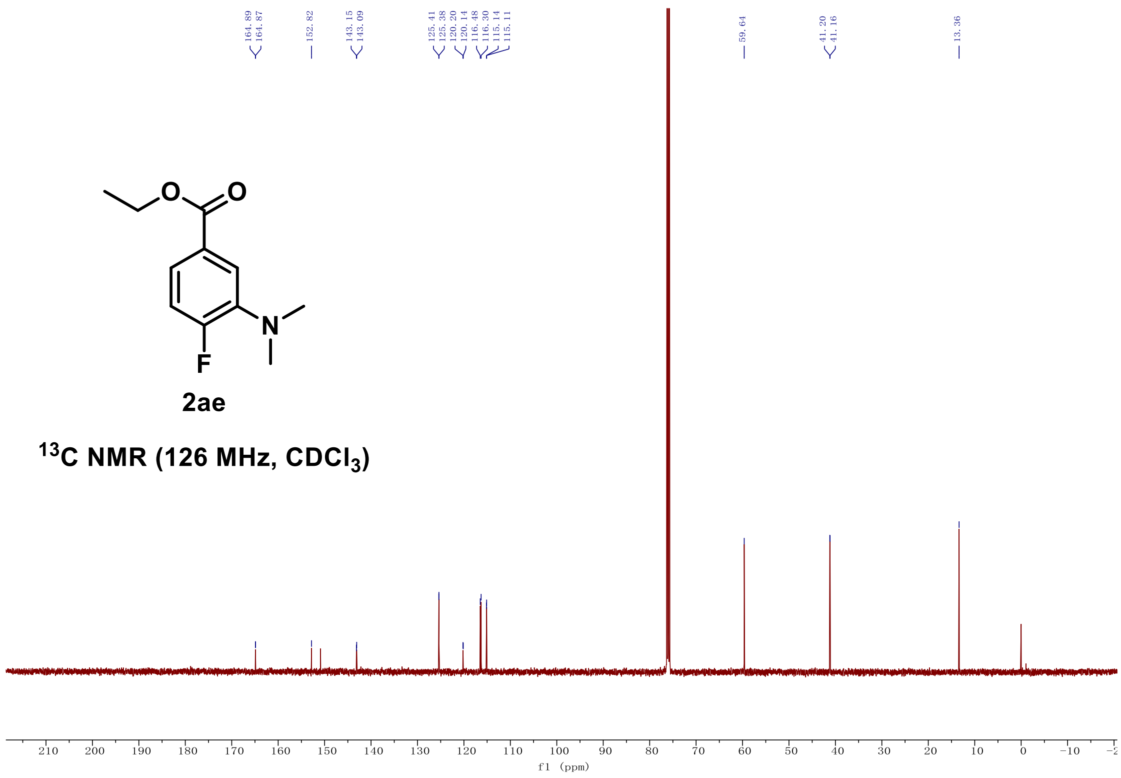


2ae

$^{13}C$ NMR (126 MHz, $CDCl_3$)

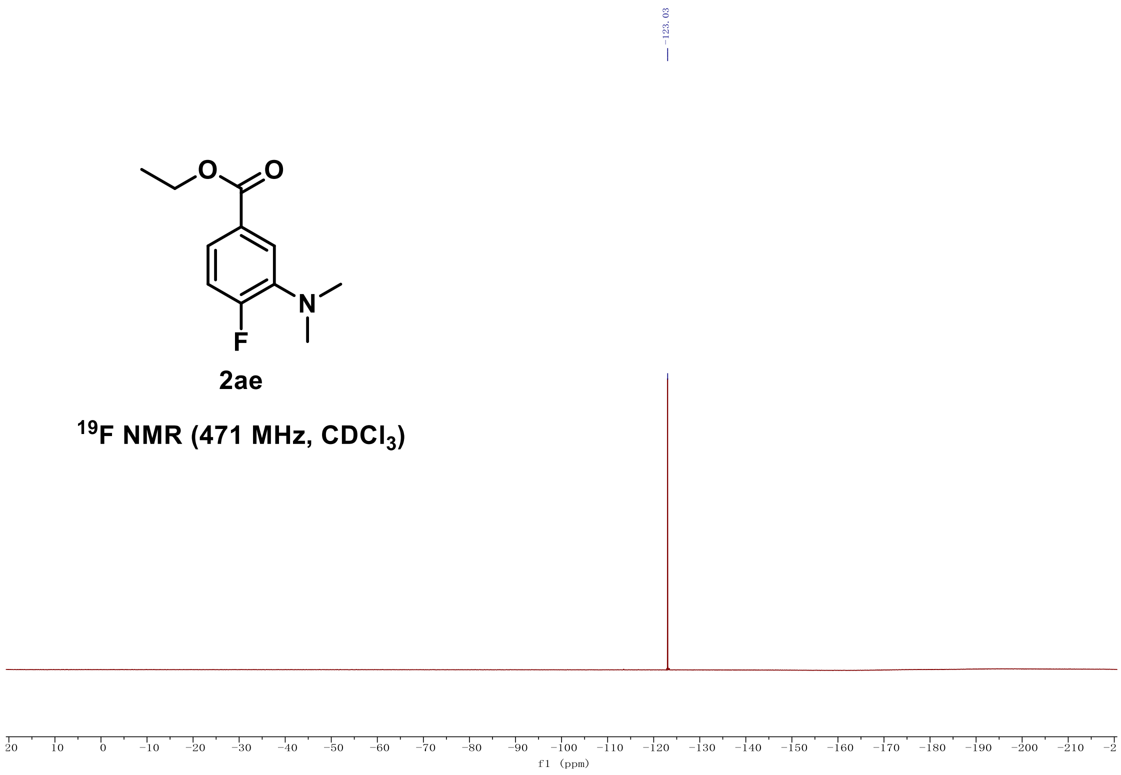


2ae

$^{19}F$ NMR (471 MHz, $CDCl_3$)

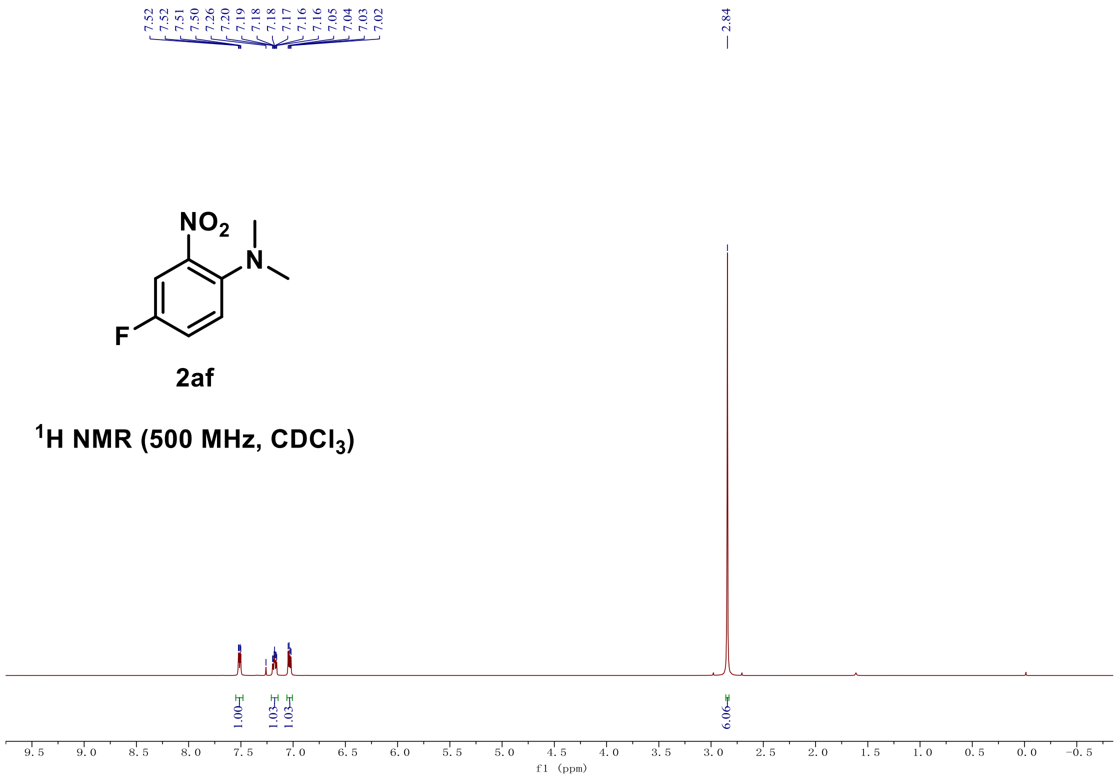

7.52 7.52 7.51 7.50 7.26 7.20 7.19 7.18 7.18 7.17 7.16 7.16 7.05 7.04 7.03 7.02
2.84
$NO_2$
N
F
2af
$^1H$ NMR (500 MHz, $CDCl_3$)
1.00
1.03
1.03
6.06
f1 (ppm)


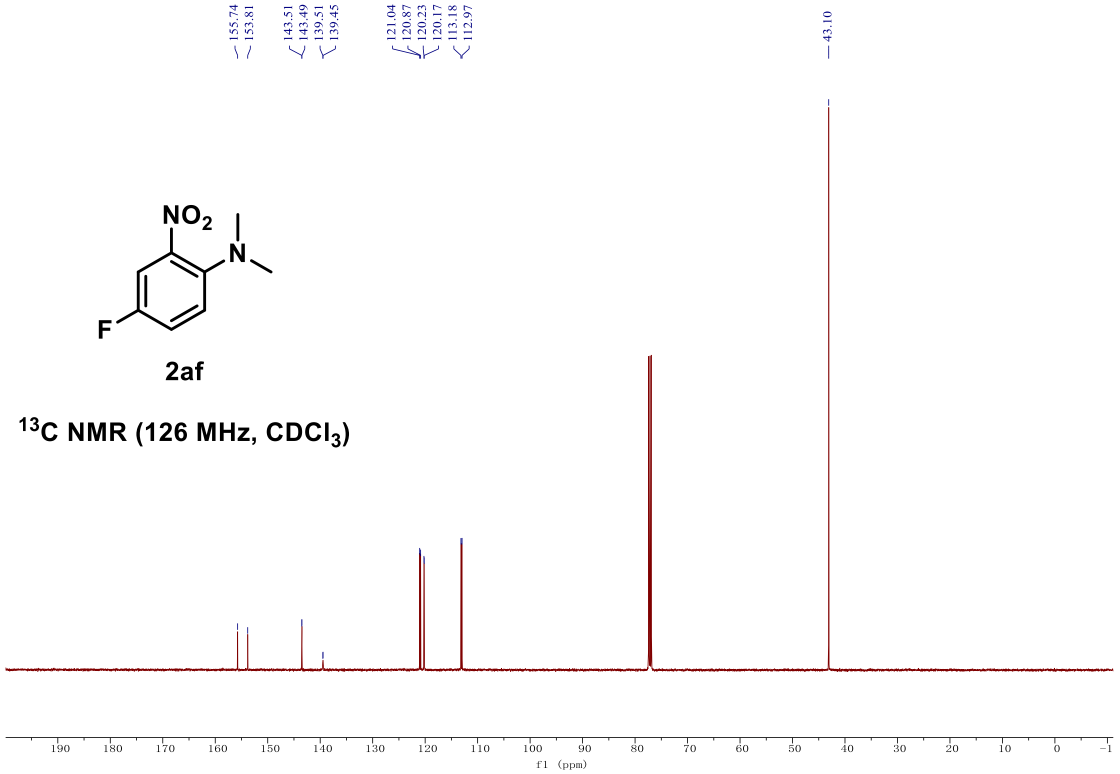

155.74 153.81 143.51 143.49 139.51 139.45 121.04 120.87 120.23 120.17 113.18 112.97
43.10
$NO_2$
N
F
2af
$^{13}C$ NMR (126 MHz, $CDCl_3$)
f1 (ppm)

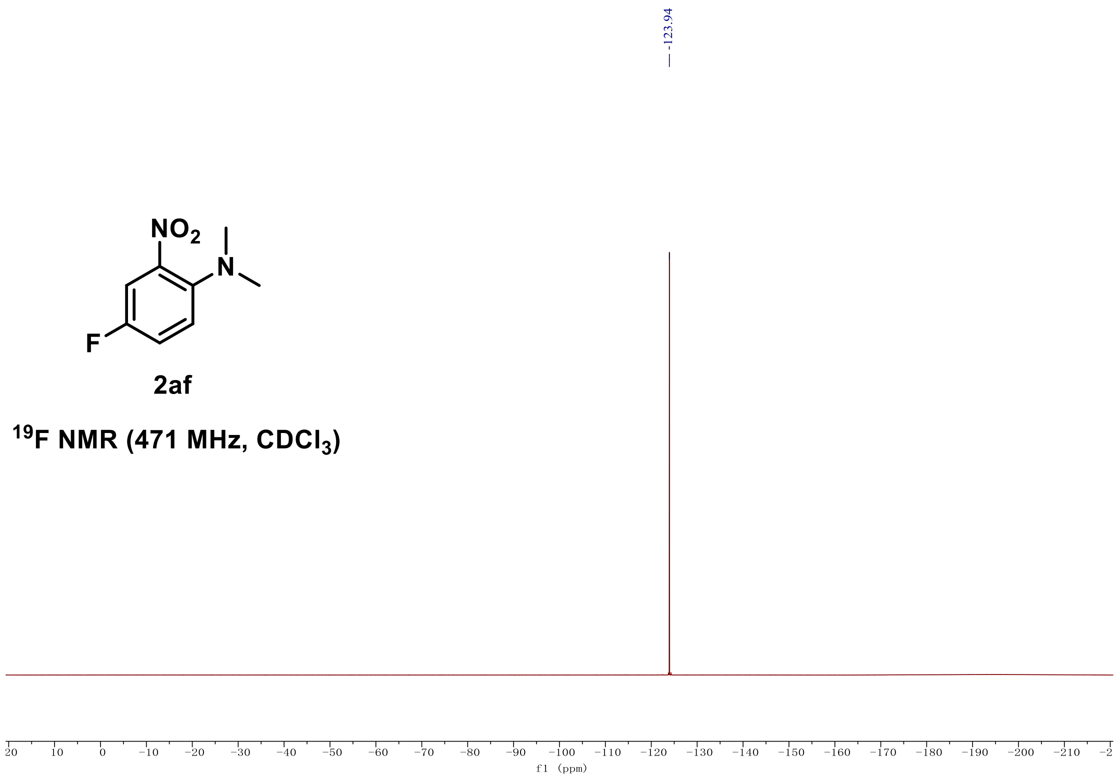


**2af**

**$^{19}$F NMR (471 MHz, $CDCl_3$)**

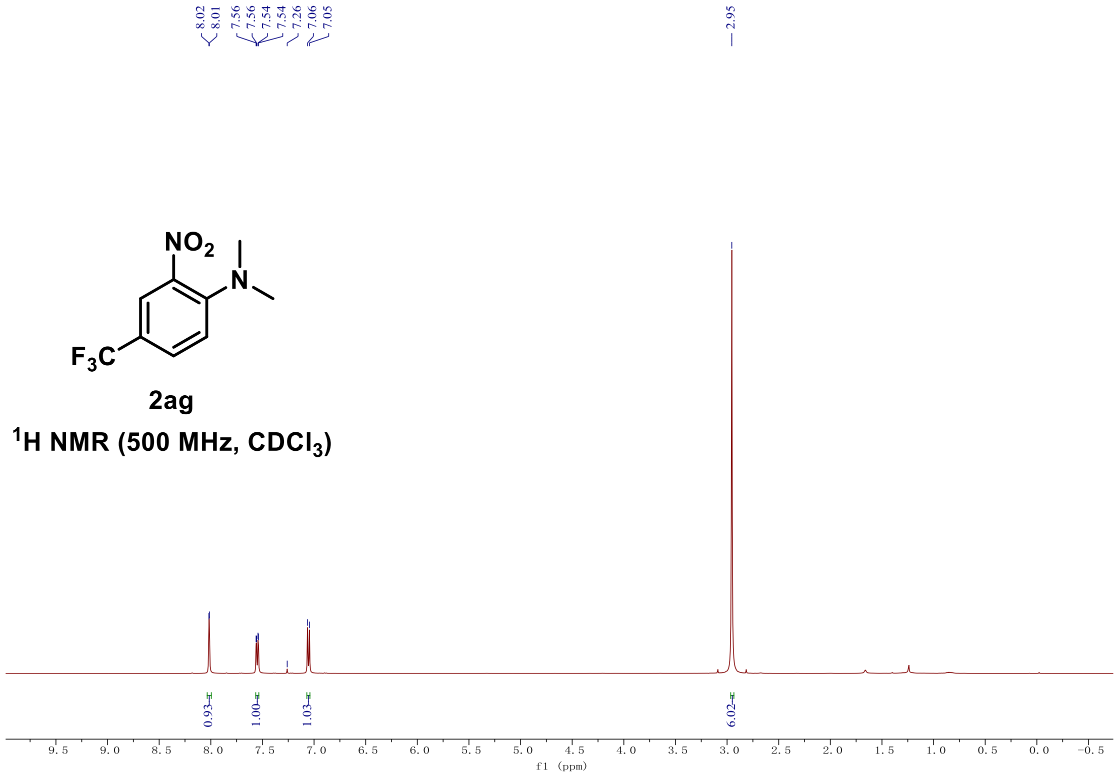


**2ag**

**$^{1}$H NMR (500 MHz, $CDCl_3$)**

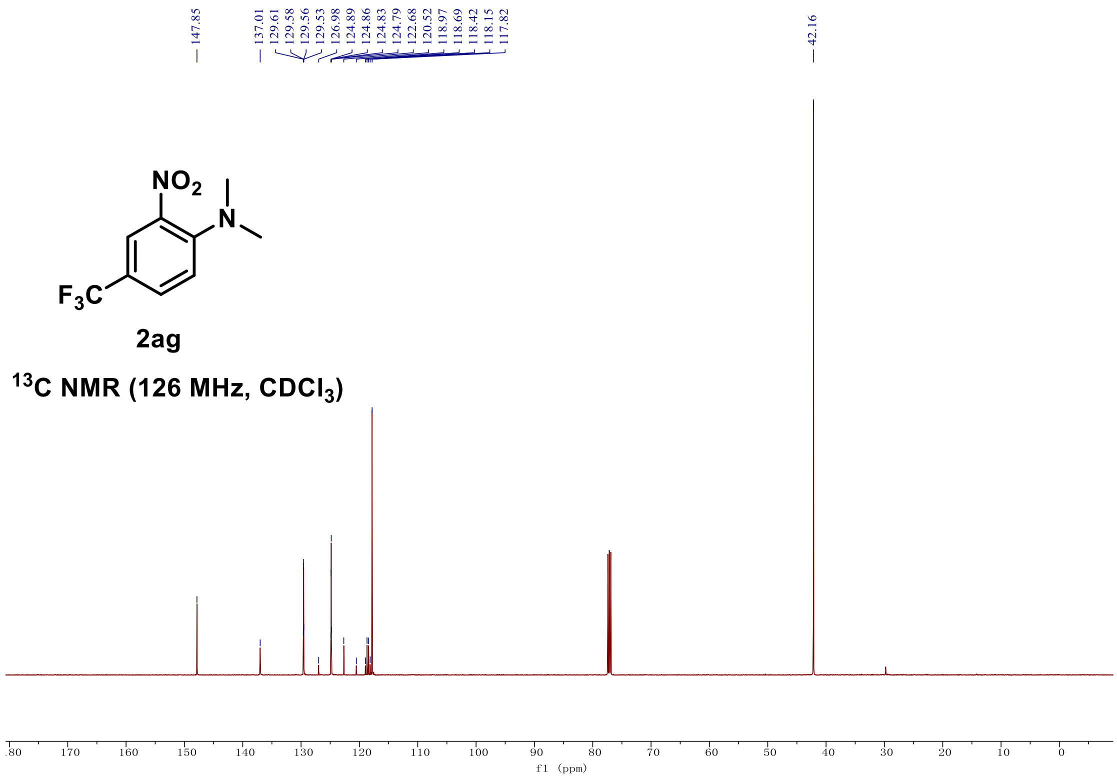
147.85
137.01
129.61
129.58
129.56
129.53
126.98
124.89
124.86
124.83
124.79
122.68
120.52
118.97
118.69
118.42
118.15
117.82
42.16
NO2
N
F3C
2ag
13C NMR (126 MHz, CDCl3)
f1 (ppm)

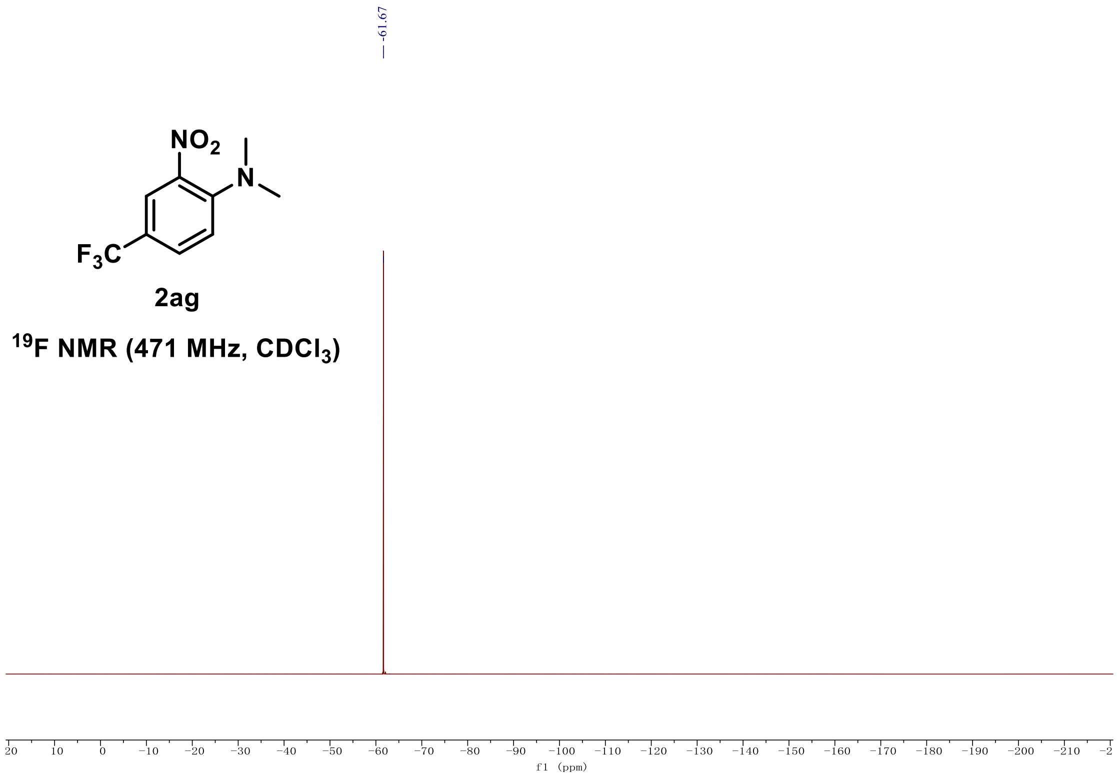
-61.67
NO2
N
F3C
2ag
19F NMR (471 MHz, CDCl3)
f1 (ppm)

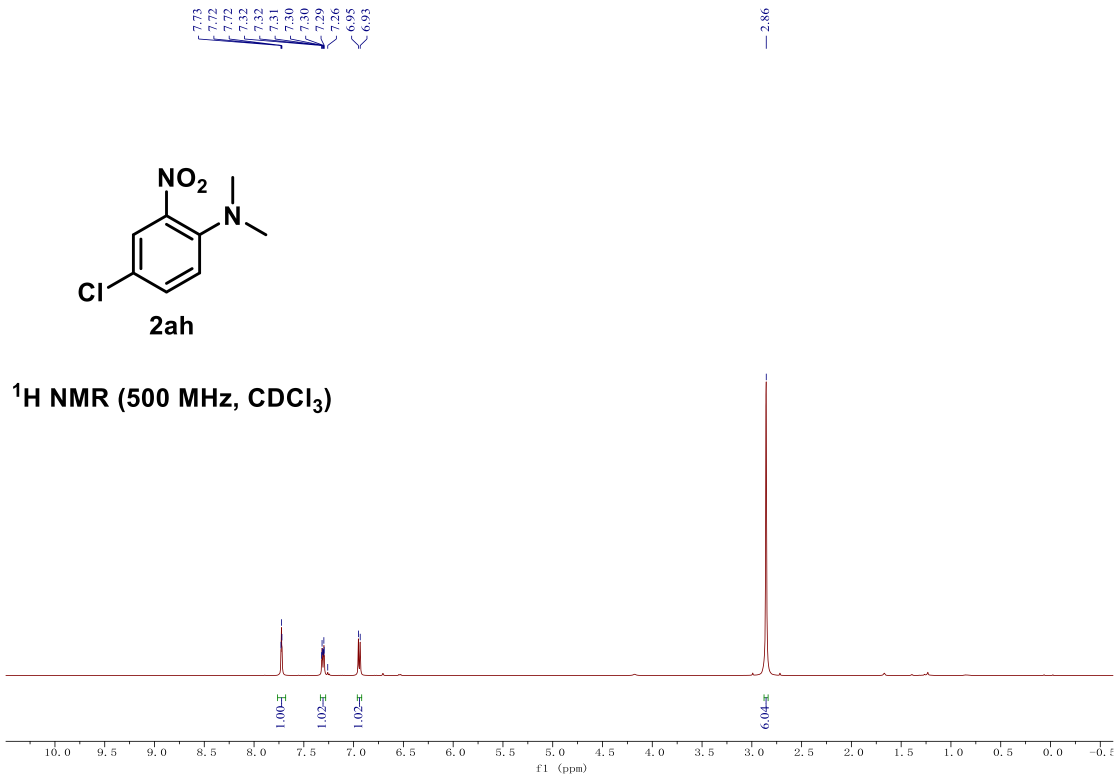


**2ah**

**$^{1}$H NMR (500 MHz, $CDCl_3$)**

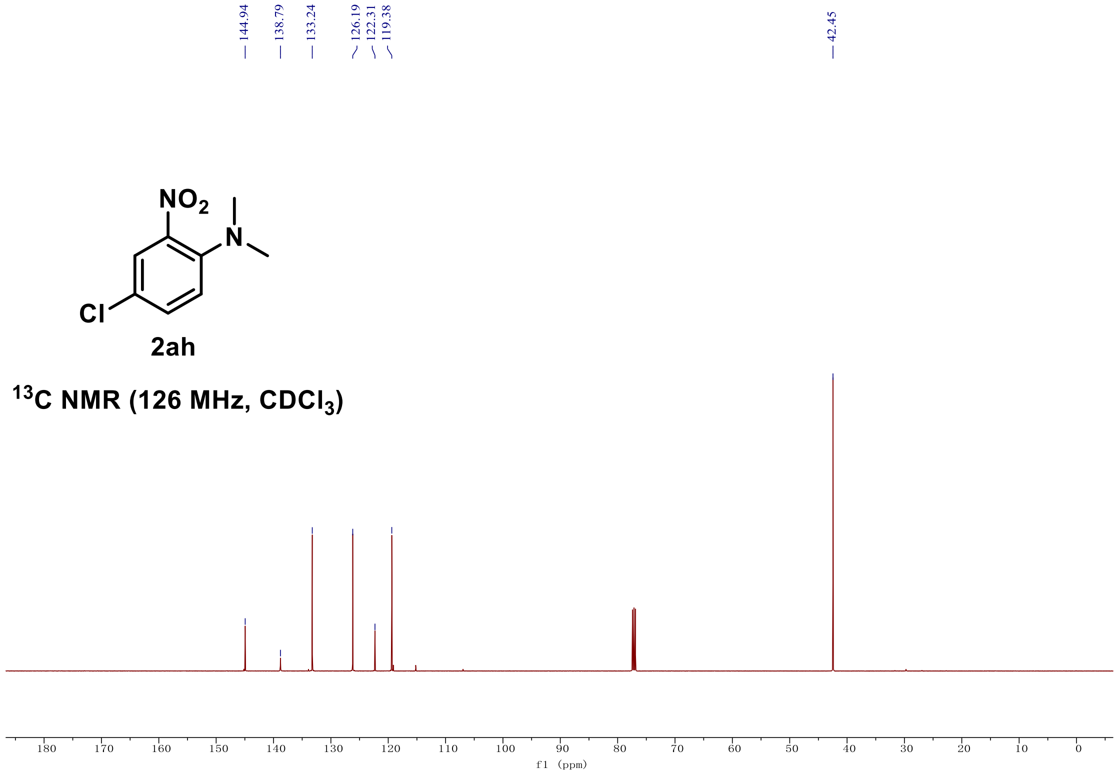


**2ah**

**$^{13}$C NMR (126 MHz, $CDCl_3$)**

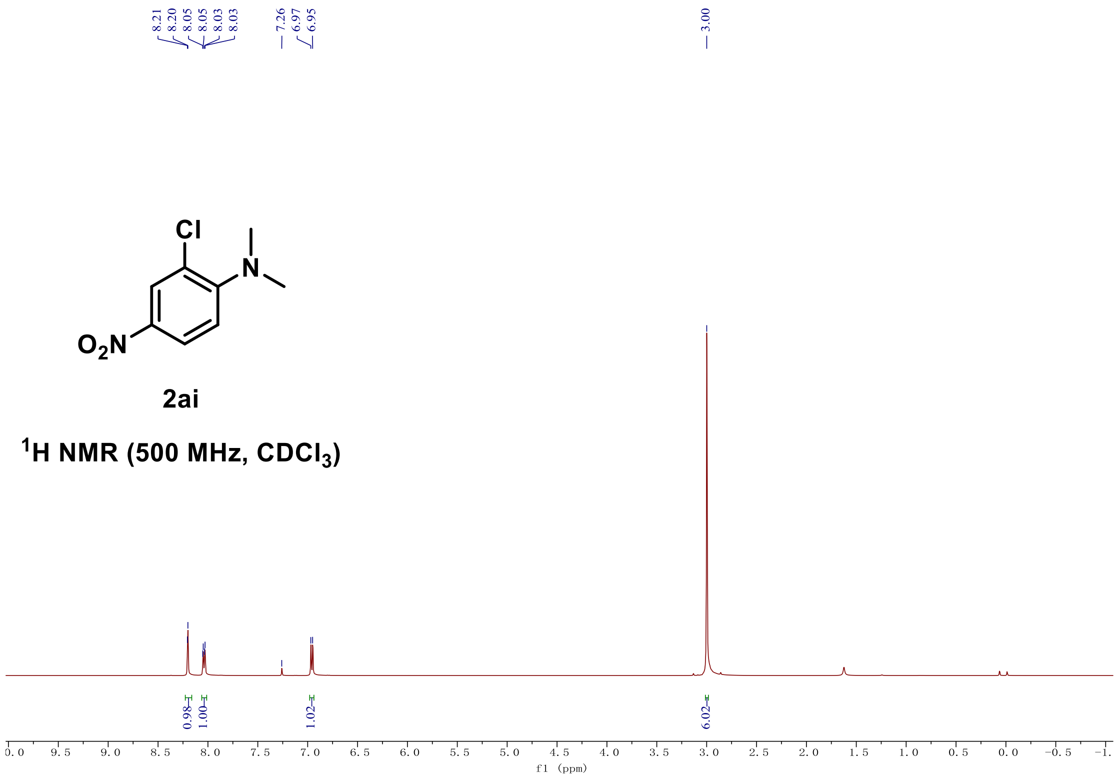


**2ai**

$^{1}$H NMR (500 MHz, CDCl$_3$)

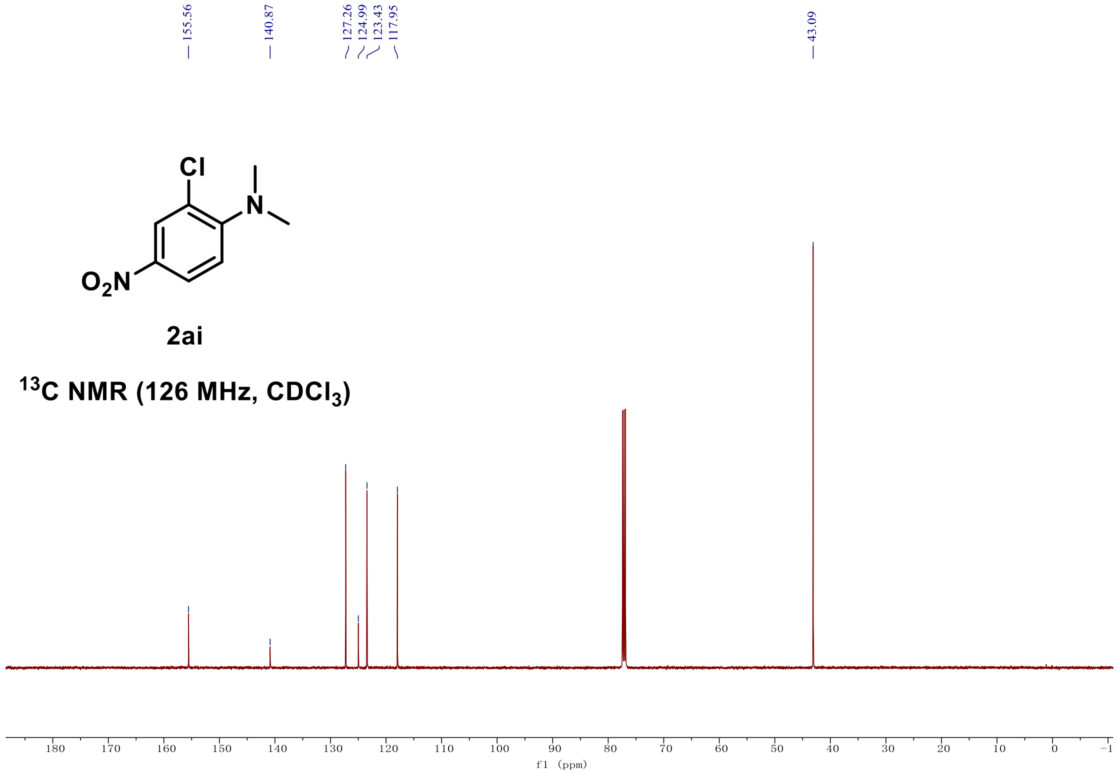


**2ai**

$^{13}$C NMR (126 MHz, CDCl$_3$)

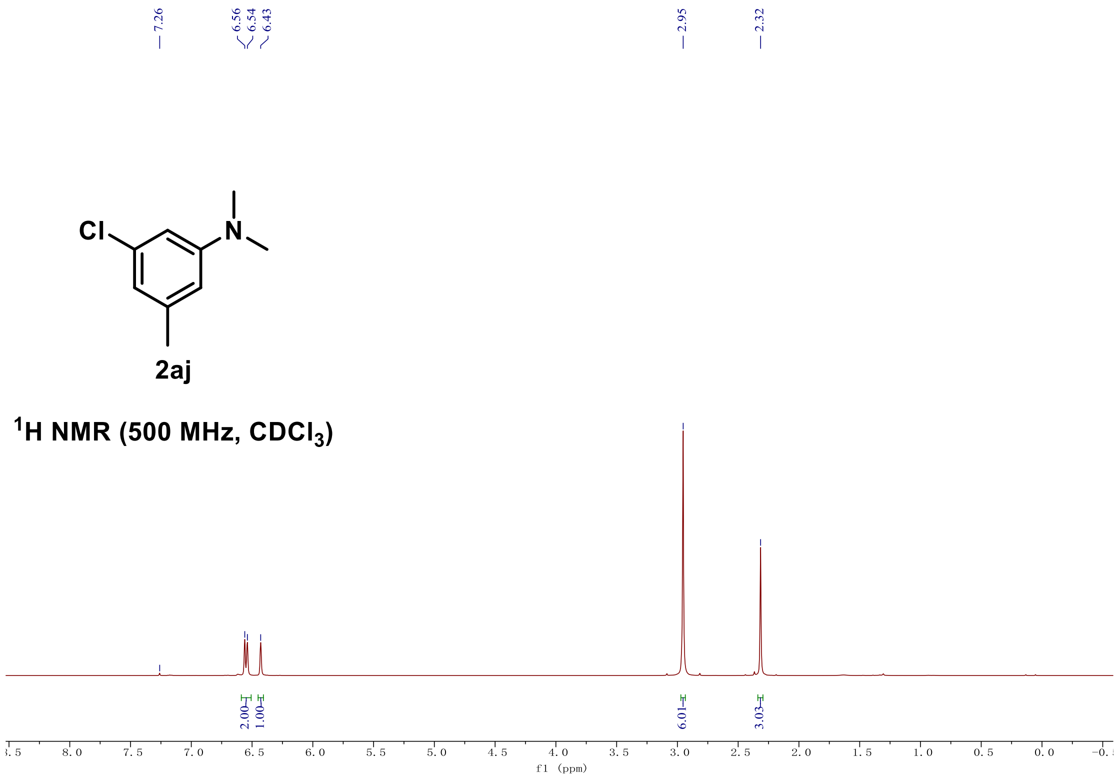
7.26
6.56
6.54
6.43
2.95
2.32
Cl
N
2aj
1H NMR (500 MHz, CDCl3)
2.00
1.00
6.01
3.03
f1 (ppm)

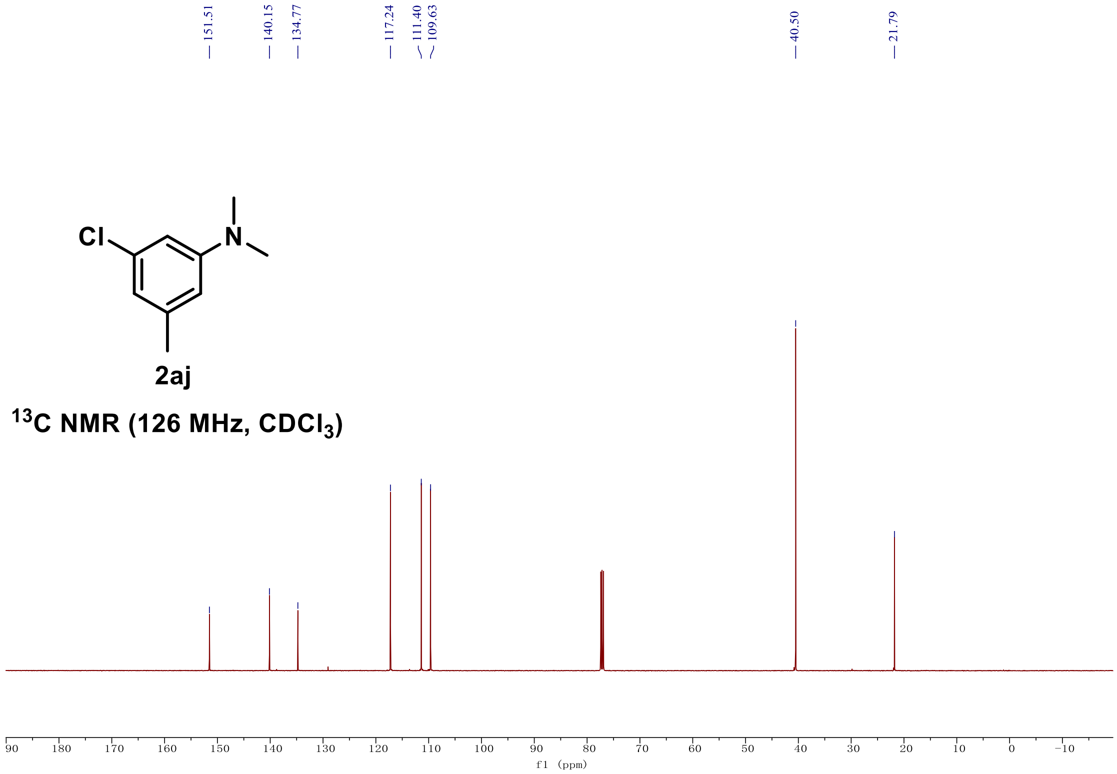
151.51
140.15
134.77
117.24
111.40
109.63
40.50
21.79
Cl
N
2aj
13C NMR (126 MHz, CDCl3)
f1 (ppm)

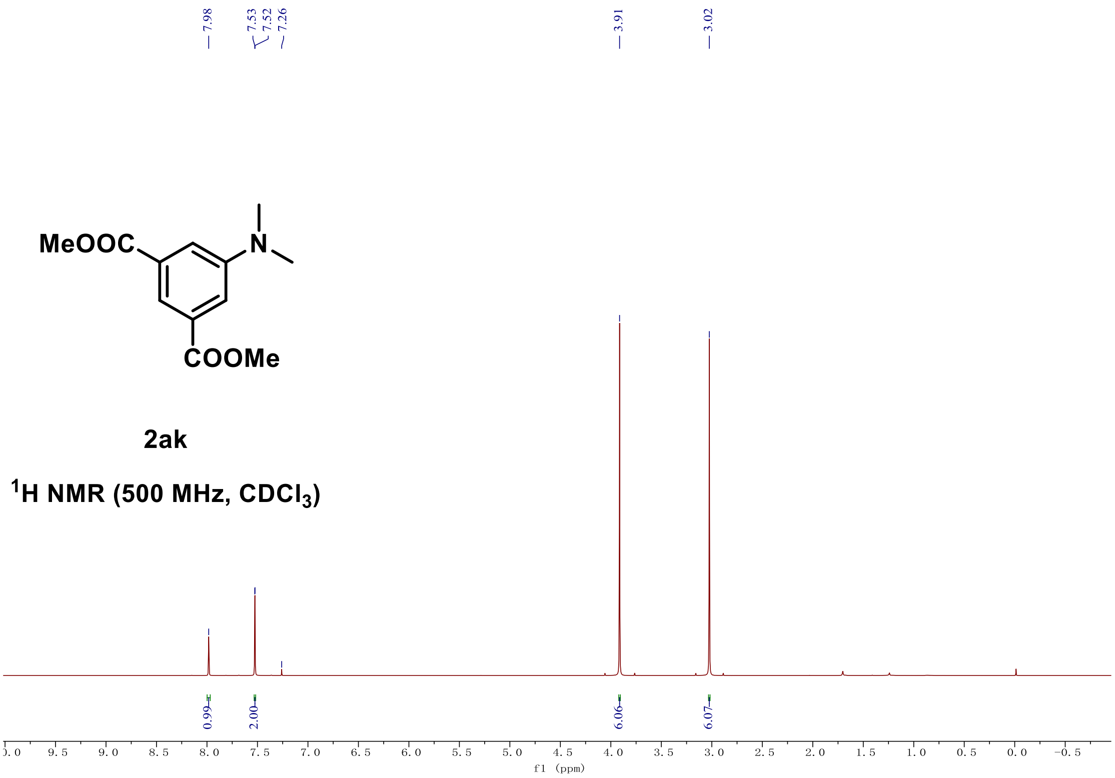


**2ak**

**$^{1}H$ NMR (500 MHz, $CDCl_3$)**

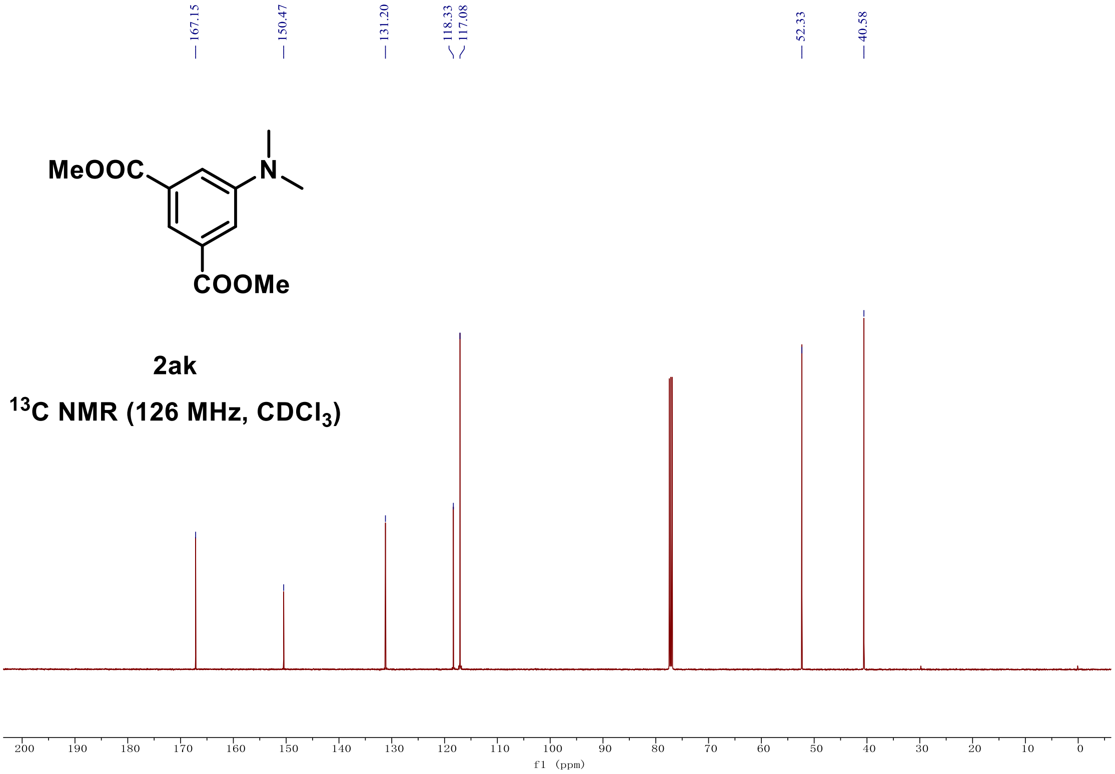


**2ak**

**$^{13}C$ NMR (126 MHz, $CDCl_3$)**

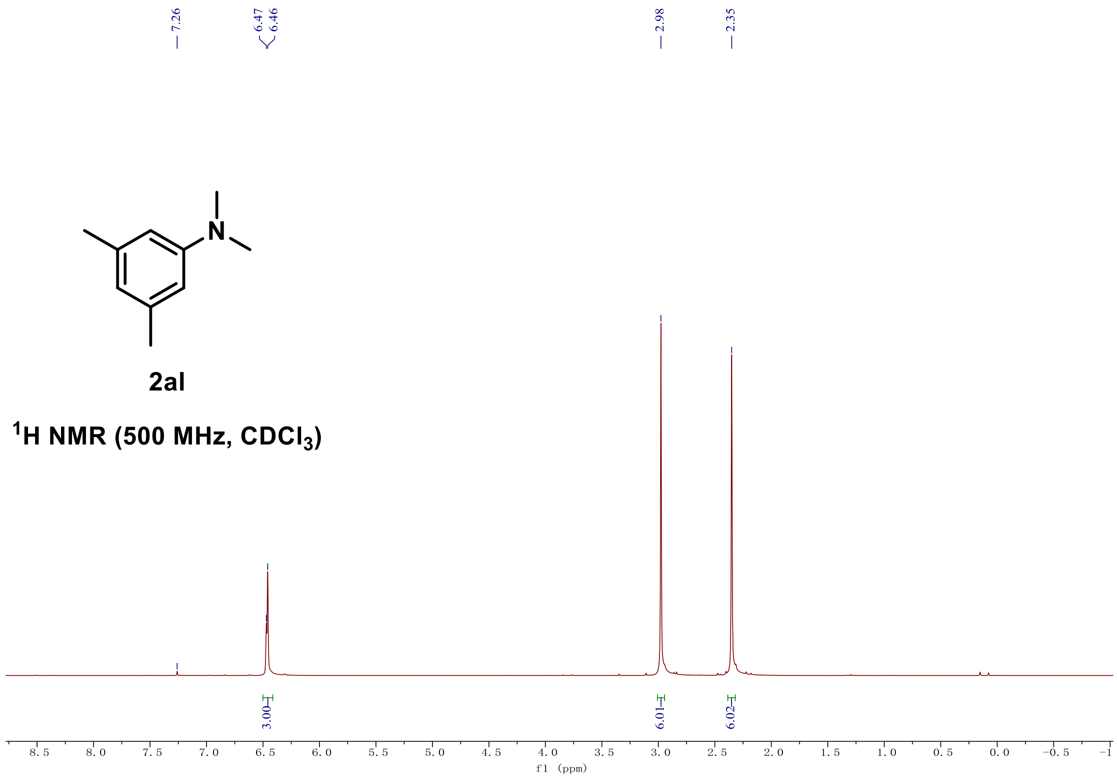
7.26
6.47
6.46
2.98
2.35
2al
$^1$H NMR (500 MHz, CDCl$_3$)
3.00
6.01
6.02
f1 (ppm)

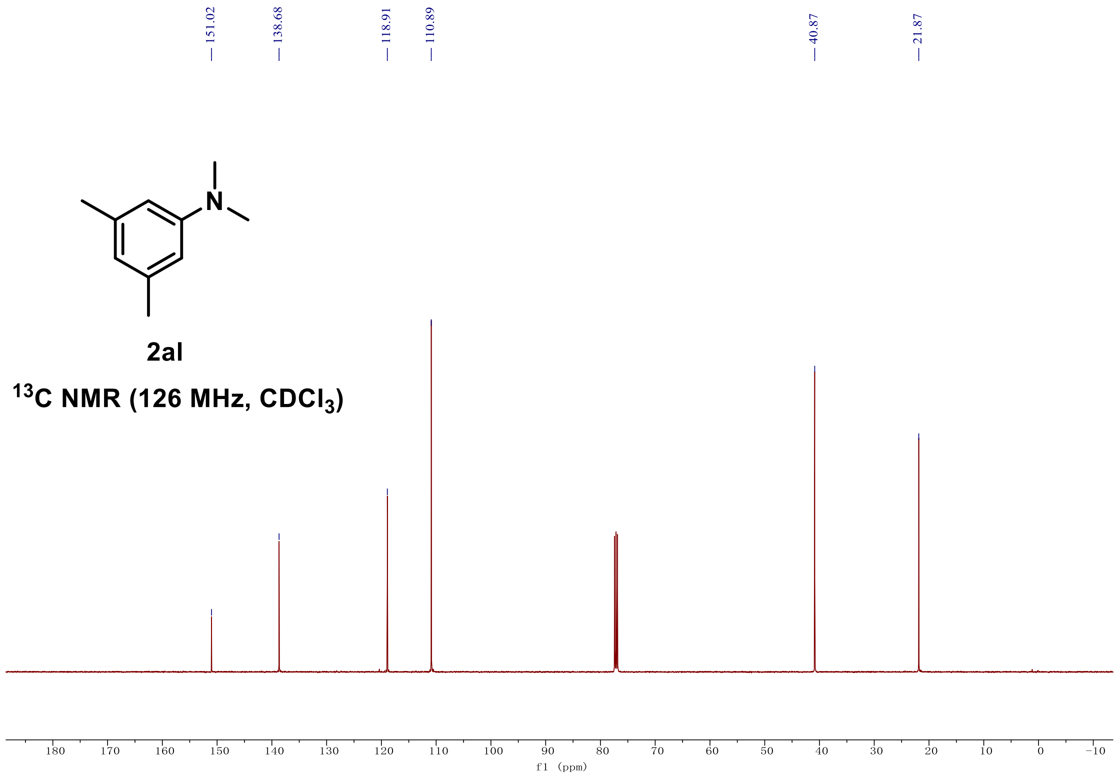
151.02
138.68
118.91
110.89
40.87
21.87
2al
$^{13}$C NMR (126 MHz, CDCl$_3$)
f1 (ppm)

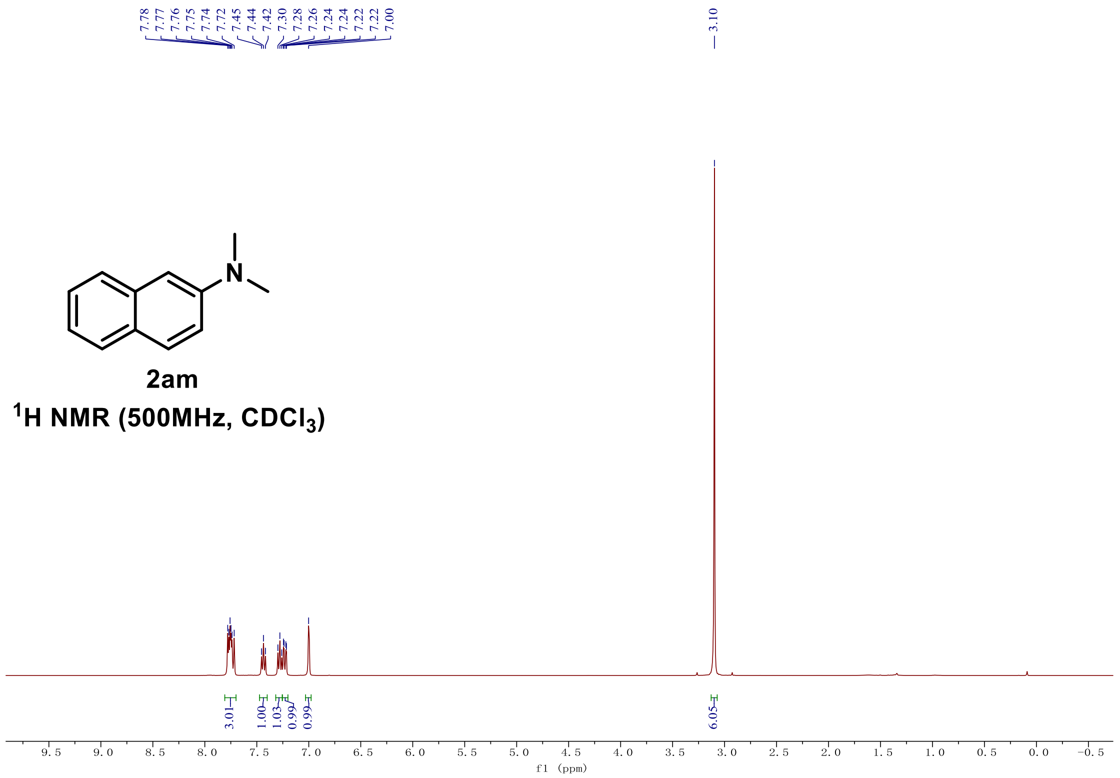


**2am**

$^1$H NMR (500MHz, $CDCl_3$)

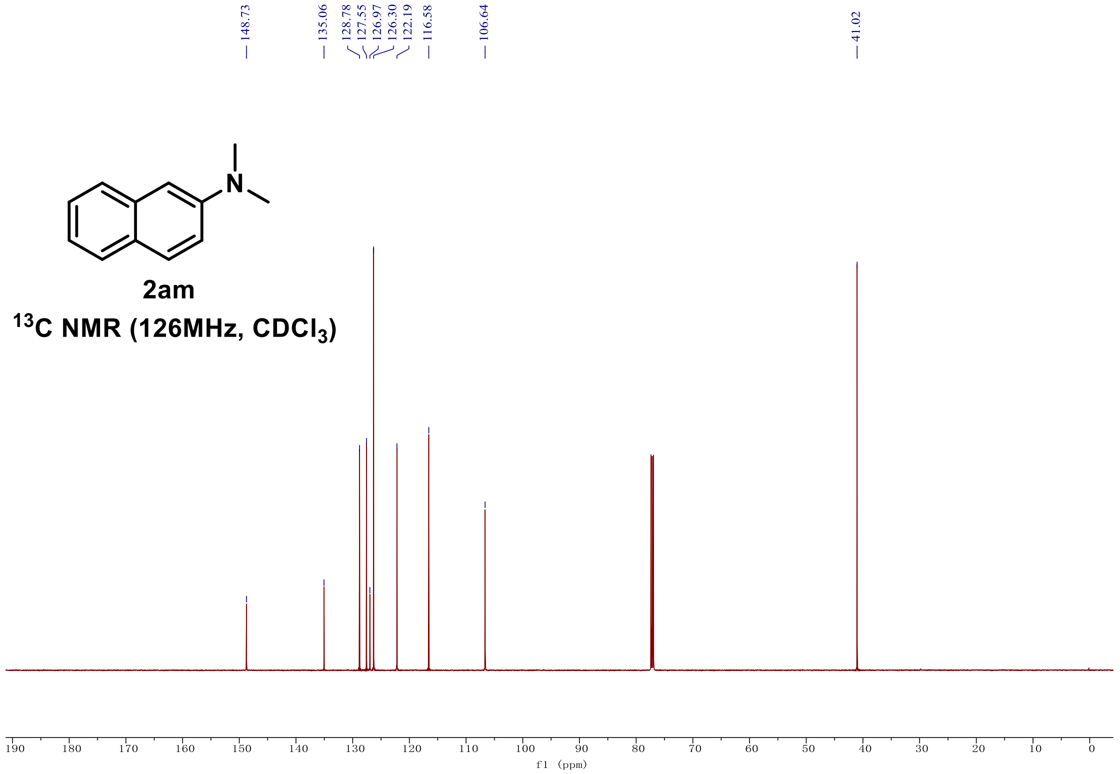


**2am**

$^{13}$C NMR (126MHz, $CDCl_3$)

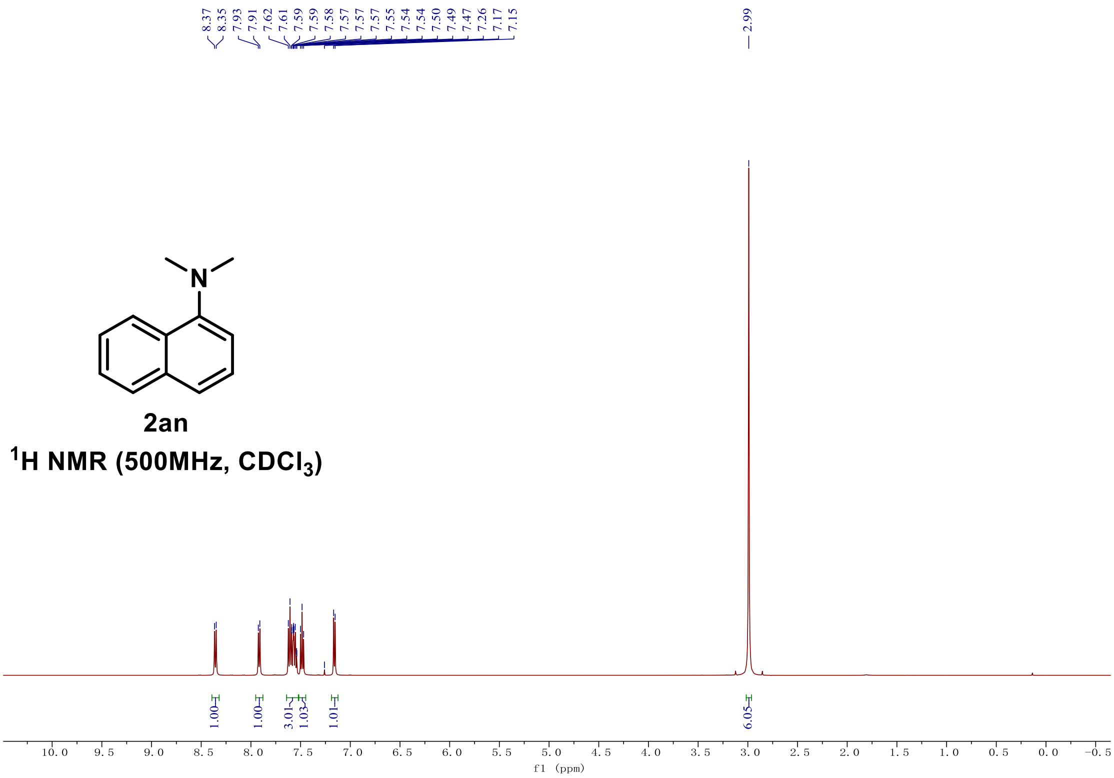


$^1H$ NMR (500MHz, $CDCl_3$)

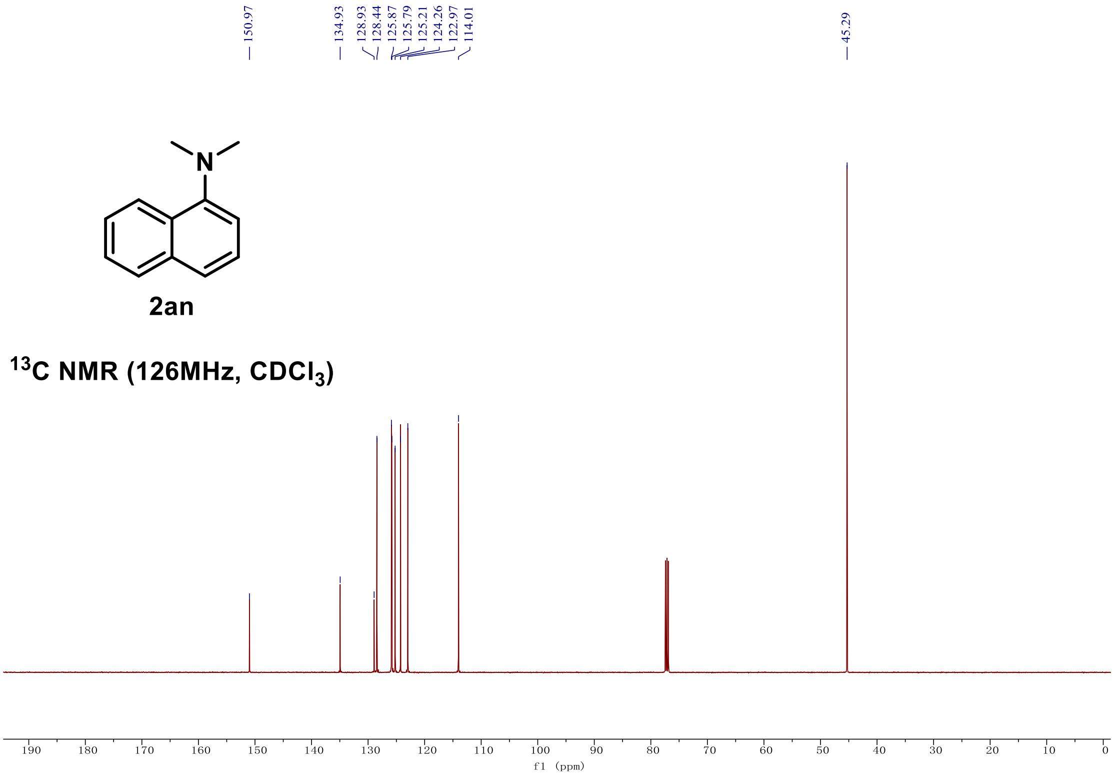


$^{13}C$ NMR (126MHz, $CDCl_3$)

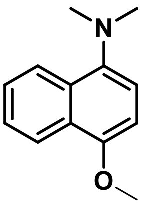

**2ao**

**$^{1}$H NMR (500 MHz, $CDCl_3$)**

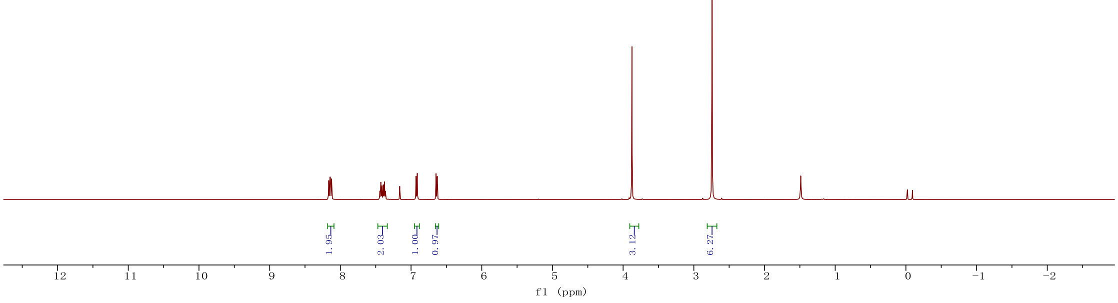


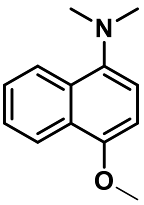

**2ao**

**$^{13}$C NMR (126 MHz, $CDCl_3$)**

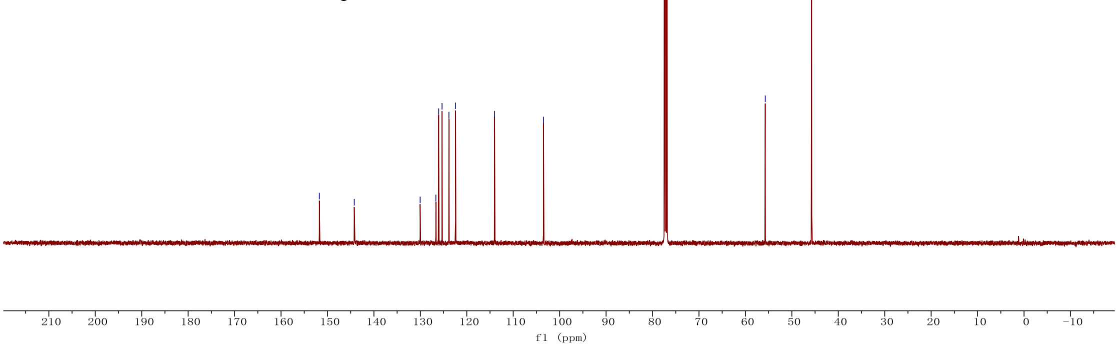

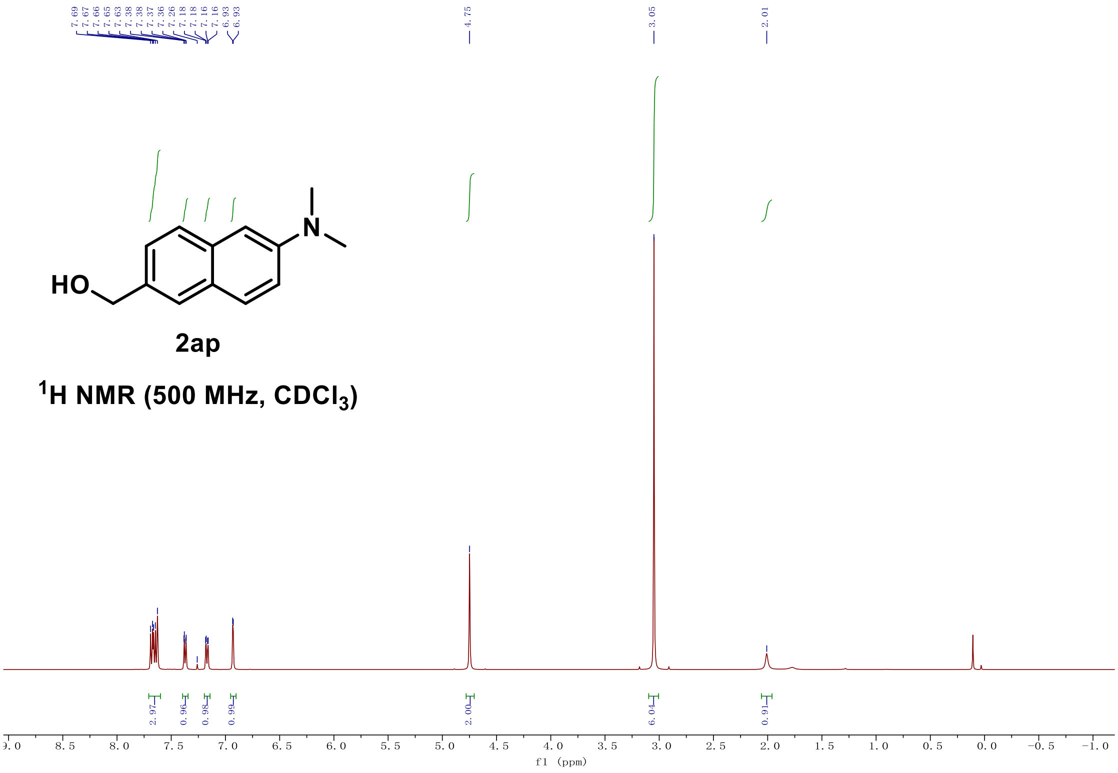
7.69 7.67 7.66 7.65 7.63 7.38 7.38 7.37 7.36 7.26 7.18 7.16 7.16 6.93 6.93
4.75
3.05
2.01
HO
N
2ap
1H NMR (500 MHz, CDCl3)
2.97 0.96 0.98 0.99 2.00 6.04 0.91
f1 (ppm)

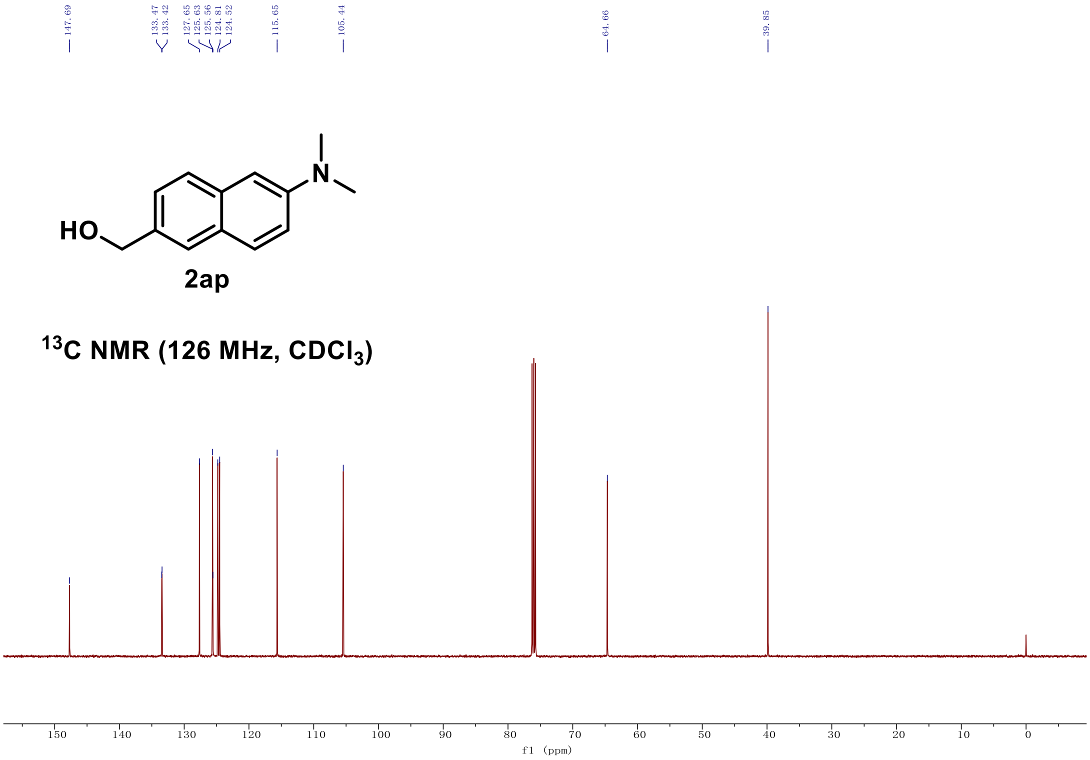
147.69
133.47 133.42 127.65 125.63 125.56 124.81 124.52
115.65
105.44
64.66
39.85
HO
N
2ap
13C NMR (126 MHz, CDCl3)
f1 (ppm)

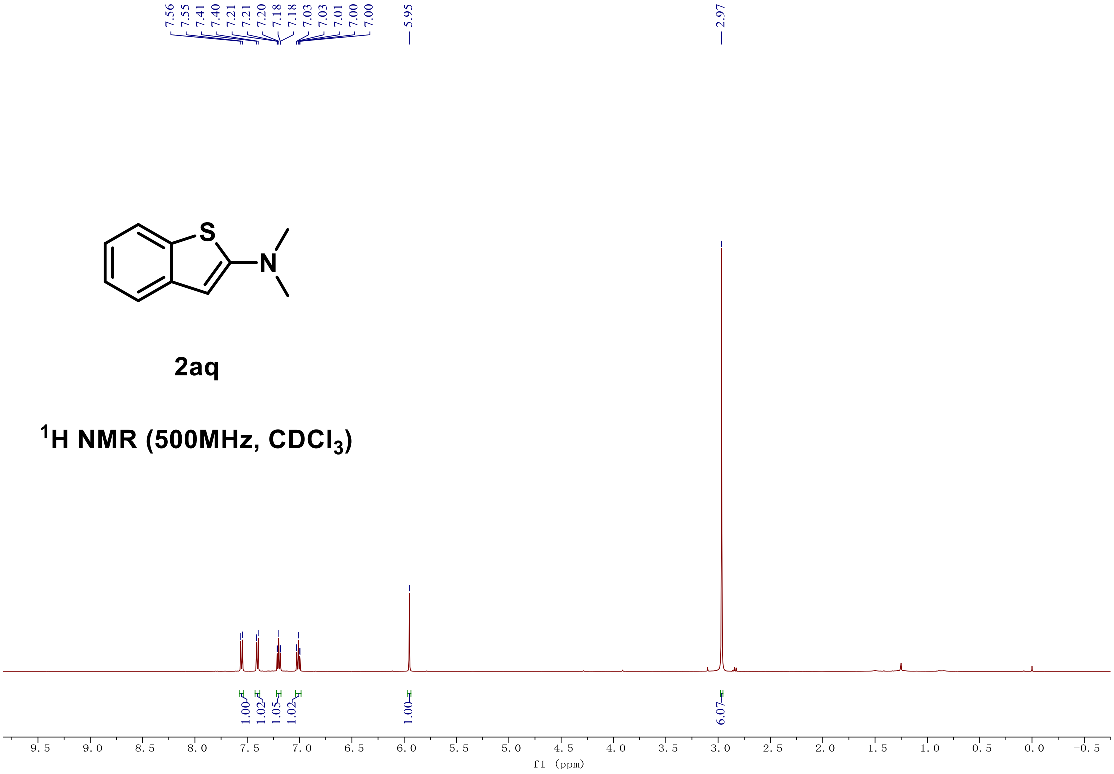
7.56
7.55
7.41
7.40
7.21
7.21
7.20
7.18
7.18
7.03
7.03
7.01
7.00
7.00
5.95
2.97
2aq
1H NMR (500MHz, CDCl3)
1.00
1.02
1.05
1.02
1.00
6.07
f1 (ppm)

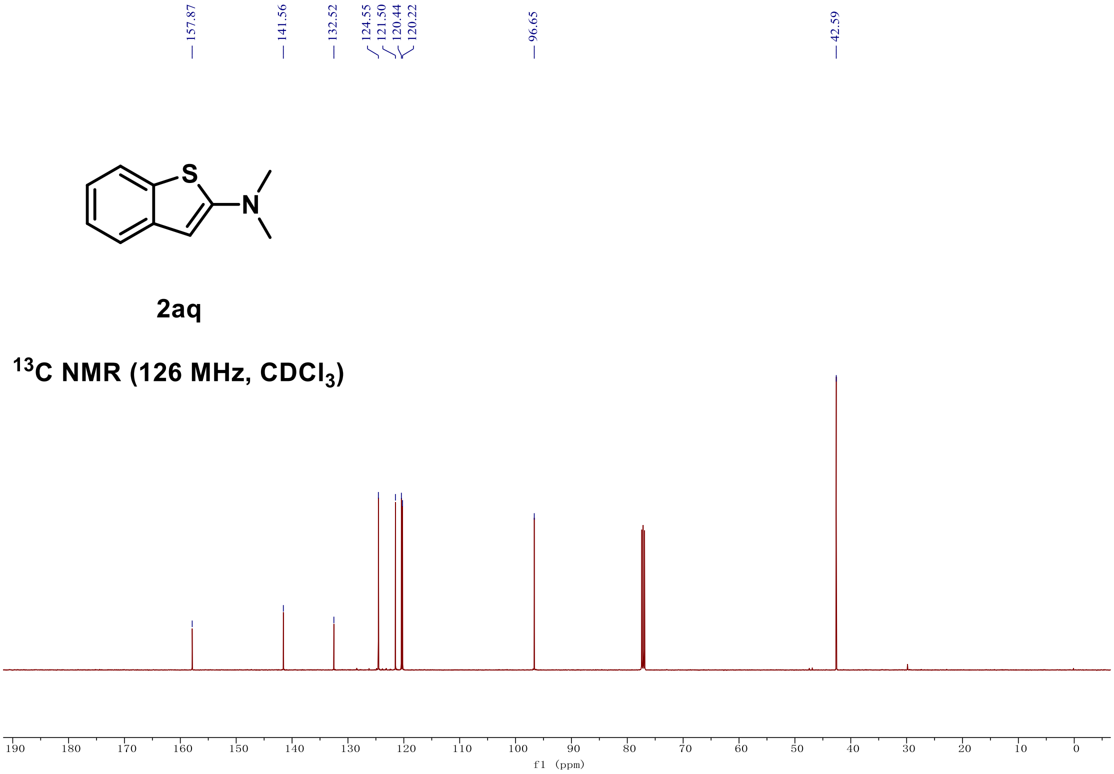
157.87
141.56
132.52
124.55
121.50
120.44
120.22
96.65
42.59
2aq
13C NMR (126 MHz, CDCl3)
f1 (ppm)

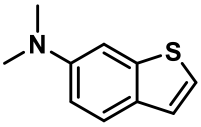

**2ar**

$^{1}$H NMR (500MHz, $CDCl_3$)

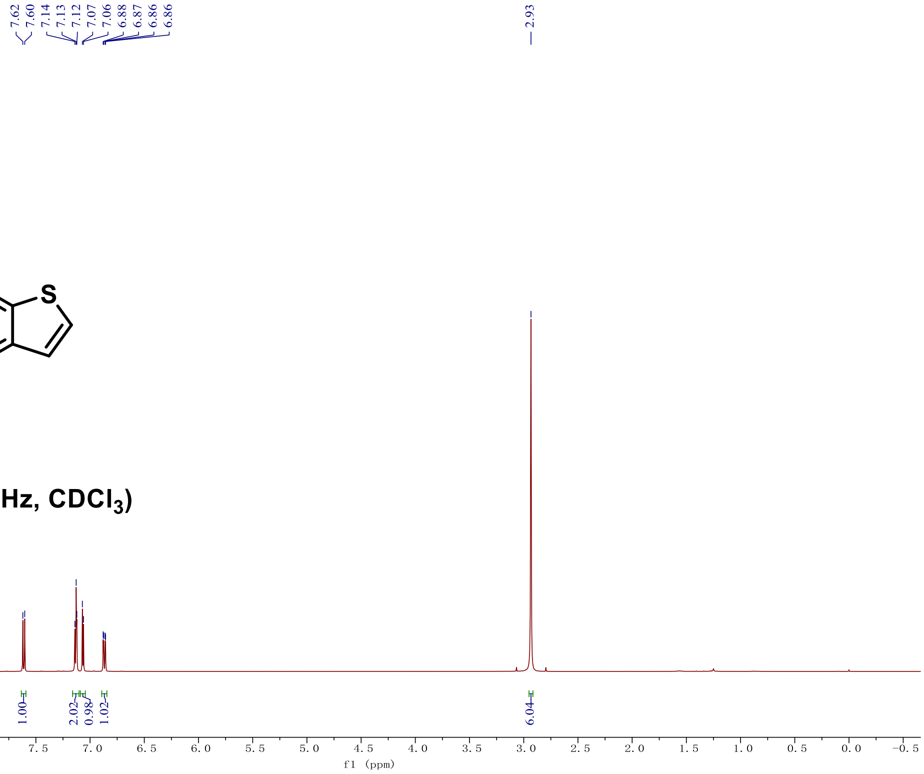


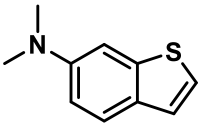

**2ar**

$^{13}$C NMR (126 MHz, $CDCl_3$)

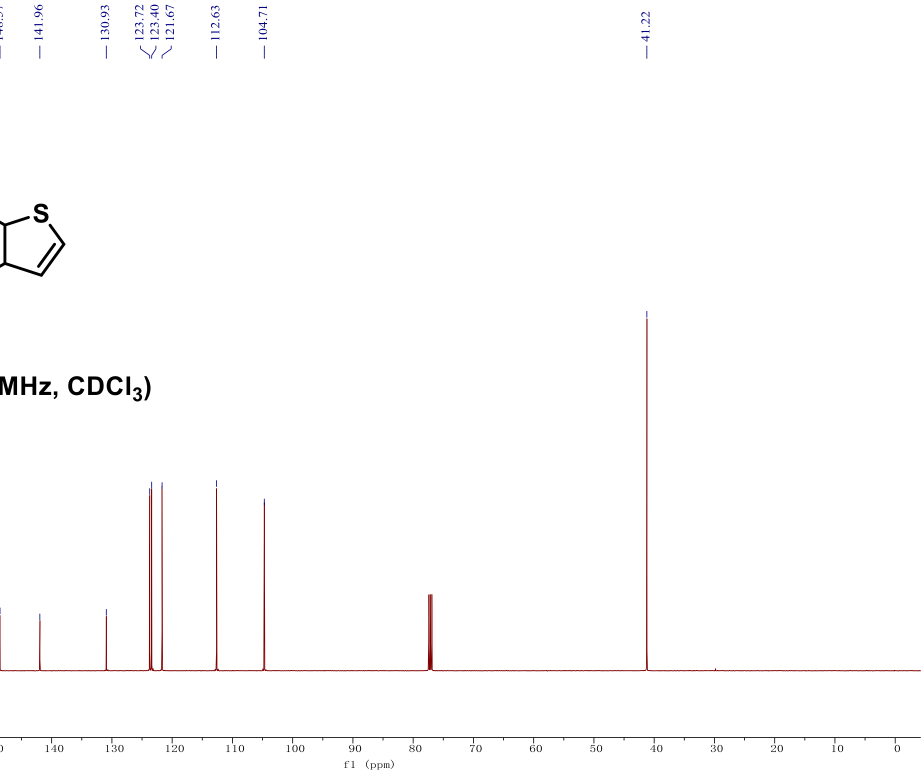

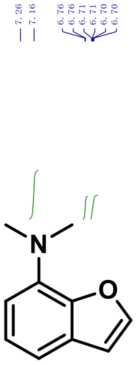

2as

$^1$H NMR (500 MHz, $CDCl_3$)

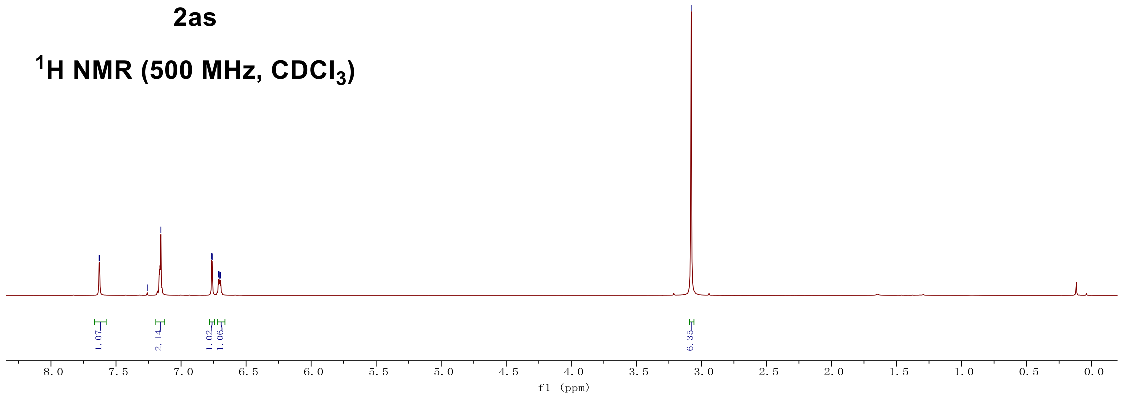


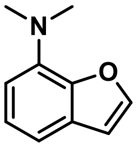

2as

$^{13}$C NMR (126 MHz, $CDCl_3$)

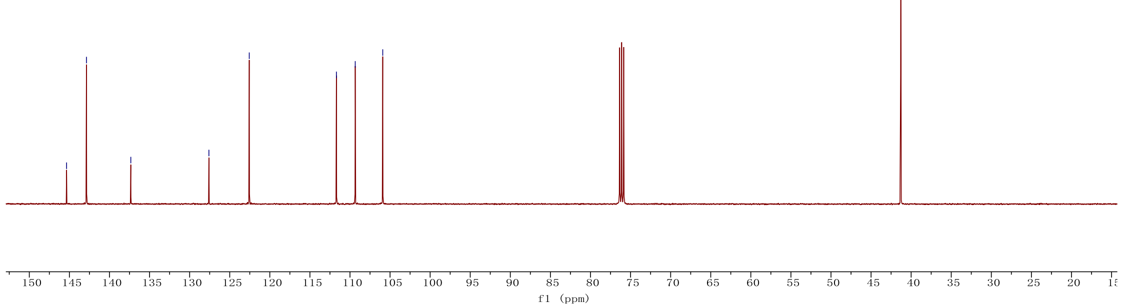

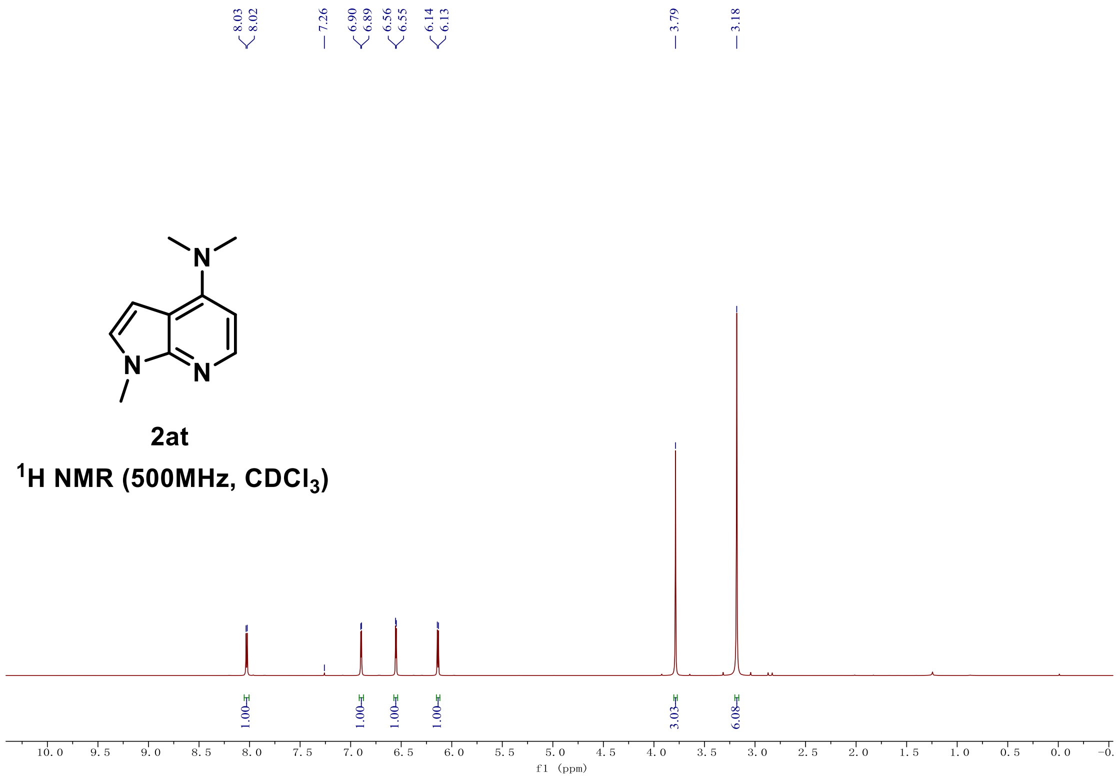
8.03
8.02
7.26
6.90
6.89
6.56
6.55
6.14
6.13
3.79
3.18
2at
1H NMR (500MHz, CDCl3)
1.00
1.00
1.00
1.00
3.03
6.08
f1 (ppm)

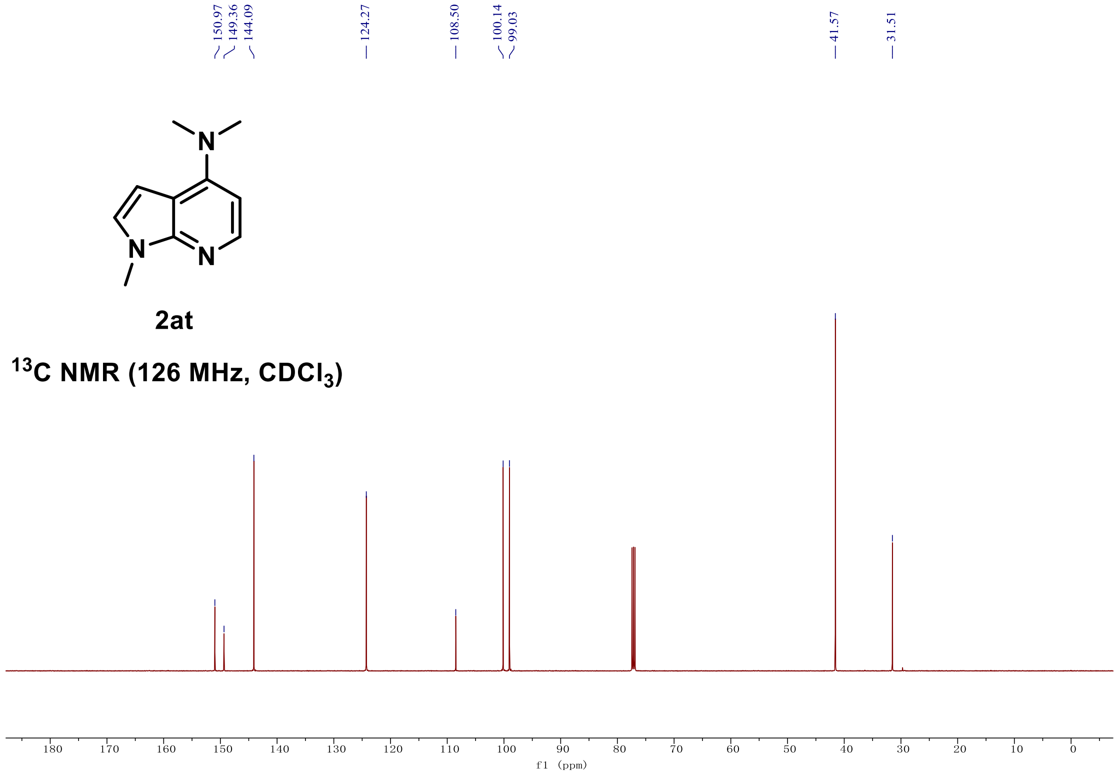
150.97
149.36
144.09
124.27
108.50
100.14
99.03
41.57
31.51
2at
13C NMR (126 MHz, CDCl3)
f1 (ppm)

2au

$^{1}H$ NMR (500MHz, $CDCl_3$)

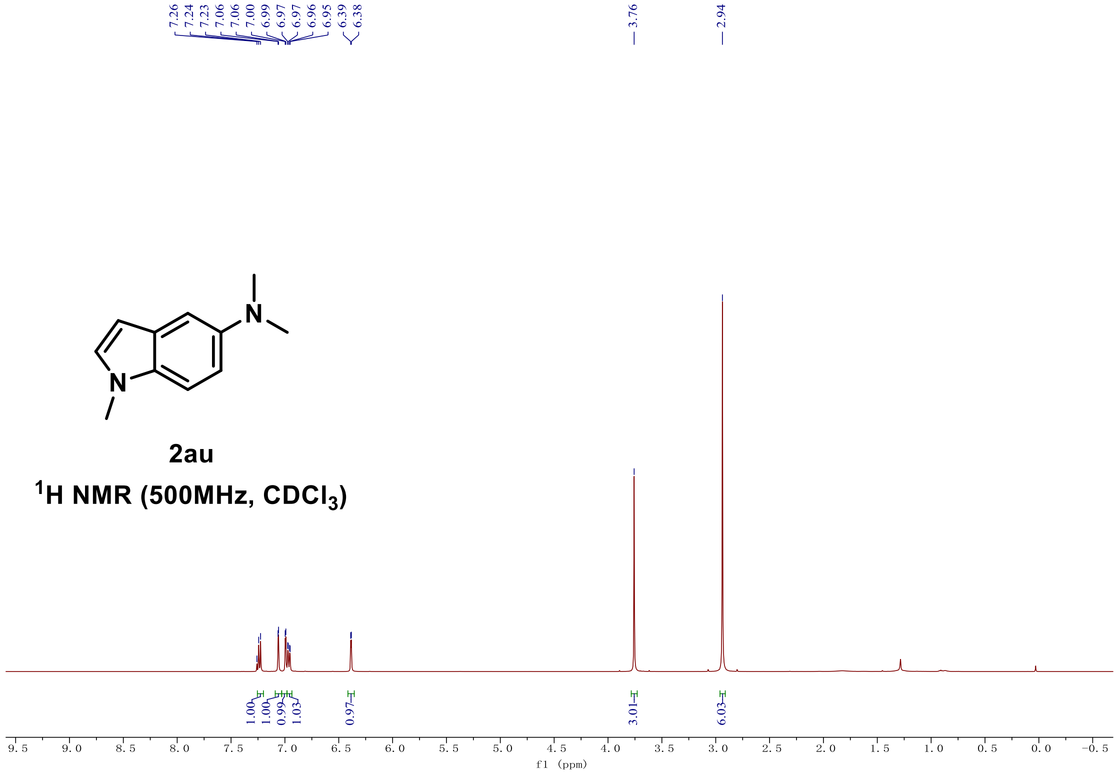


2au

$^{13}C$ NMR (126 MHz, $CDCl_3$)

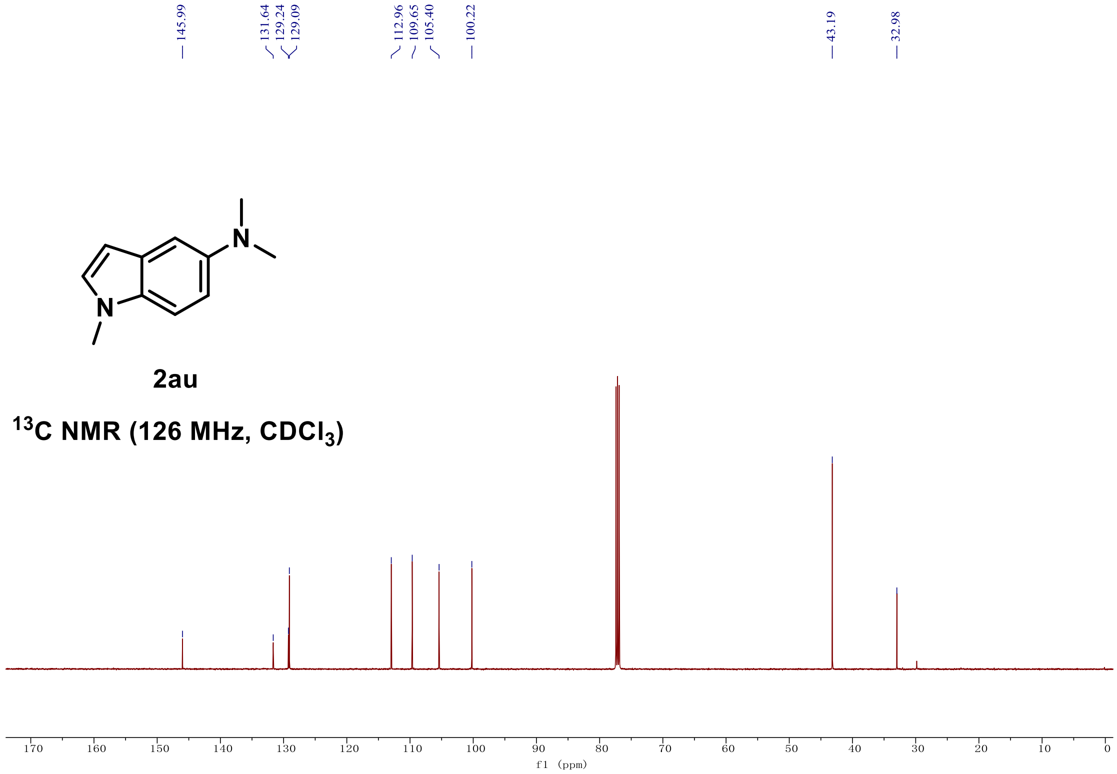

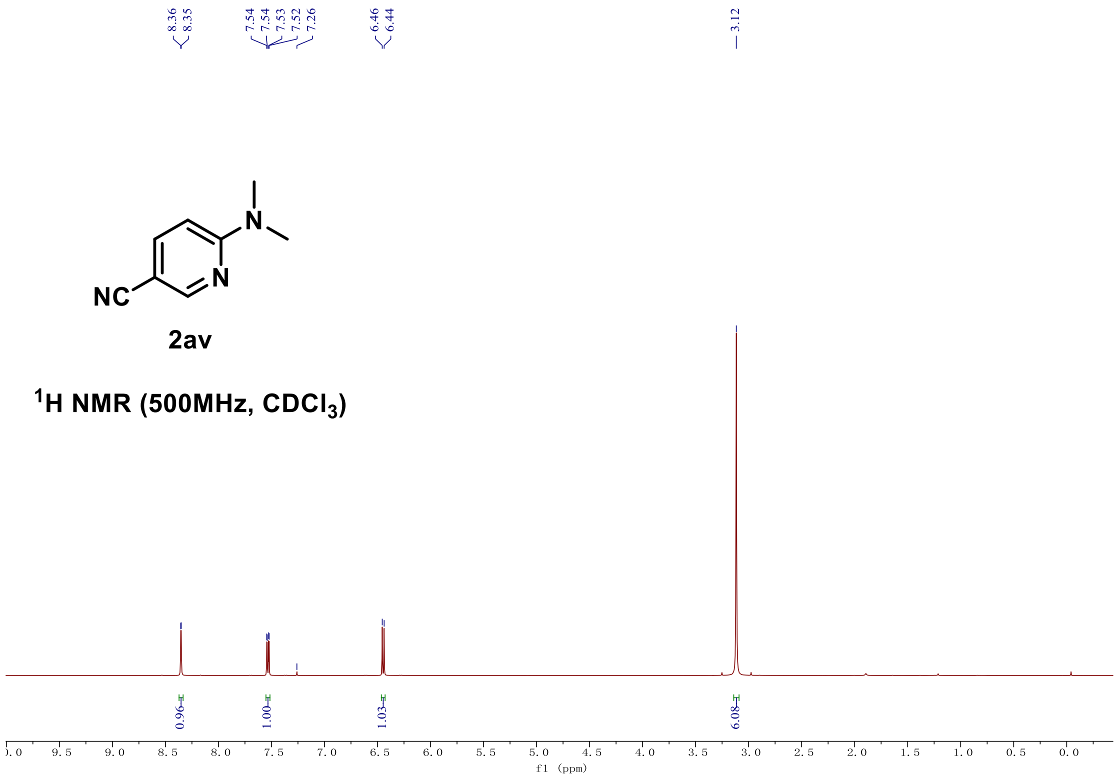
8.36
8.35
7.54
7.54
7.53
7.52
7.26
6.46
6.44
3.12
NC
N
N
2av
1H NMR (500MHz, CDCl3)
0.96
1.00
1.03
6.08
f1 (ppm)

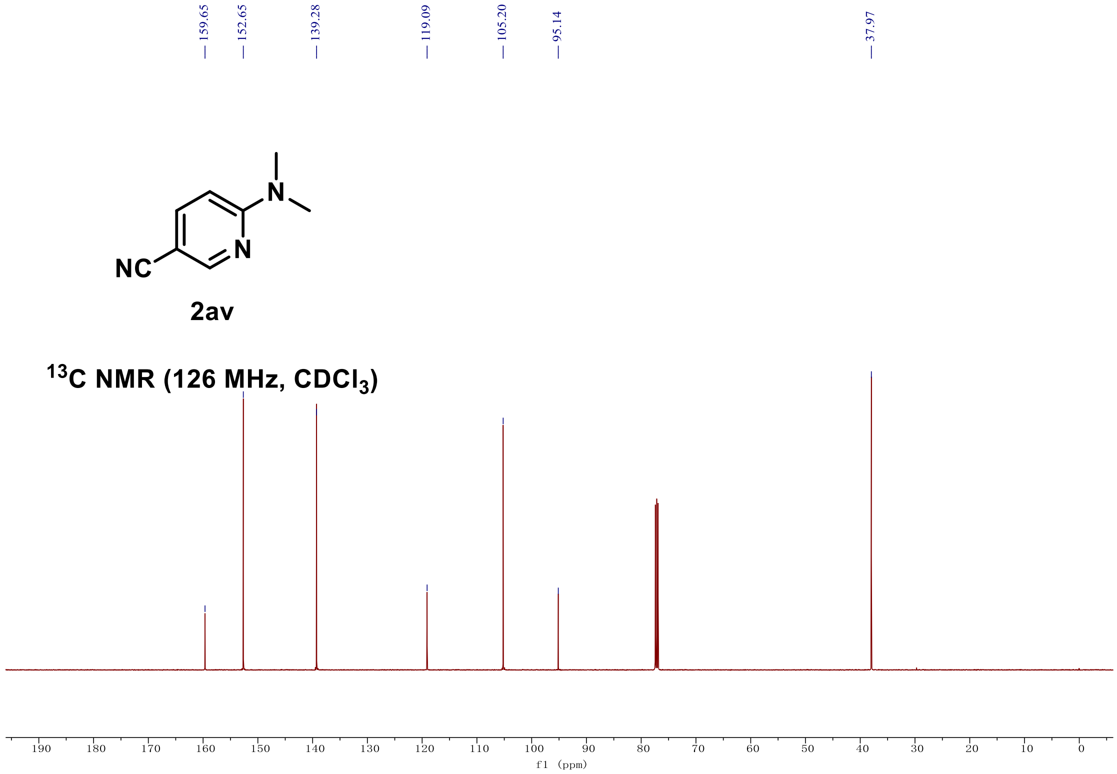
159.65
152.65
139.28
119.09
105.20
95.14
37.97
NC
N
N
2av
13C NMR (126 MHz, CDCl3)
f1 (ppm)

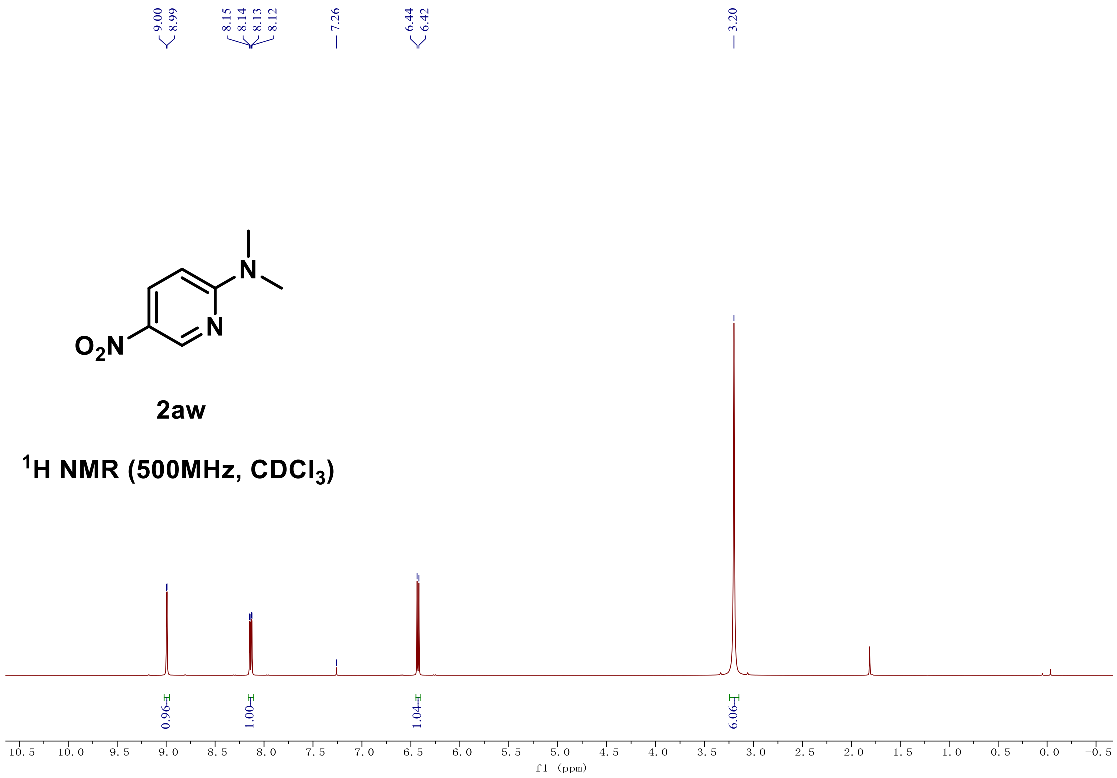
9.00
8.99
8.15
8.14
8.13
8.12
7.26
6.44
6.42
3.20
2aw
1H NMR (500MHz, CDCl3)
0.96
1.00
1.04
6.06
10.5 10.0 9.5 9.0 8.5 8.0 7.5 7.0 6.5 6.0 5.5 5.0 4.5 4.0 3.5 3.0 2.5 2.0 1.5 1.0 0.5 0.0 -0.5
f1 (ppm)

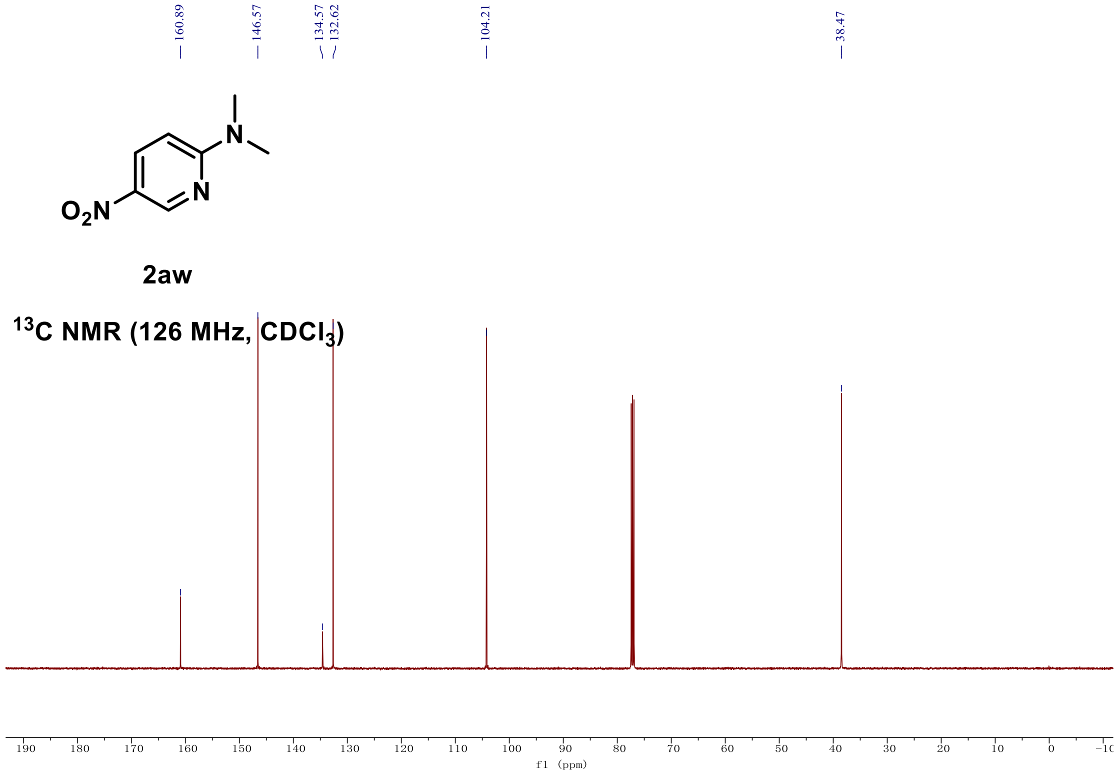
160.89
146.57
134.57
132.62
104.21
38.47
2aw
13C NMR (126 MHz, CDCl3)
190 180 170 160 150 140 130 120 110 100 90 80 70 60 50 40 30 20 10 0 -10
f1 (ppm)

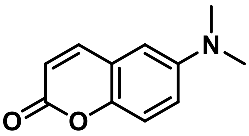

**2ax**

**$^{1}H$ NMR (500MHz, $CDCl_3$)**

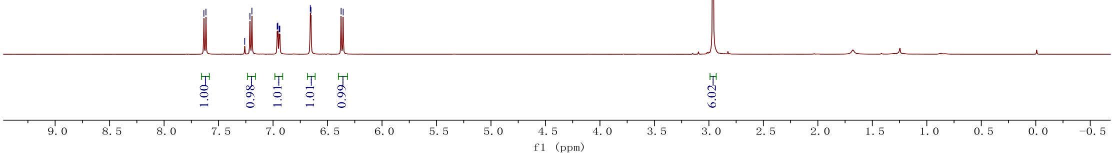


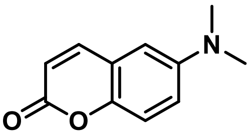

**2ax**

**$^{13}C$ NMR (126 MHz, $CDCl_3$)**

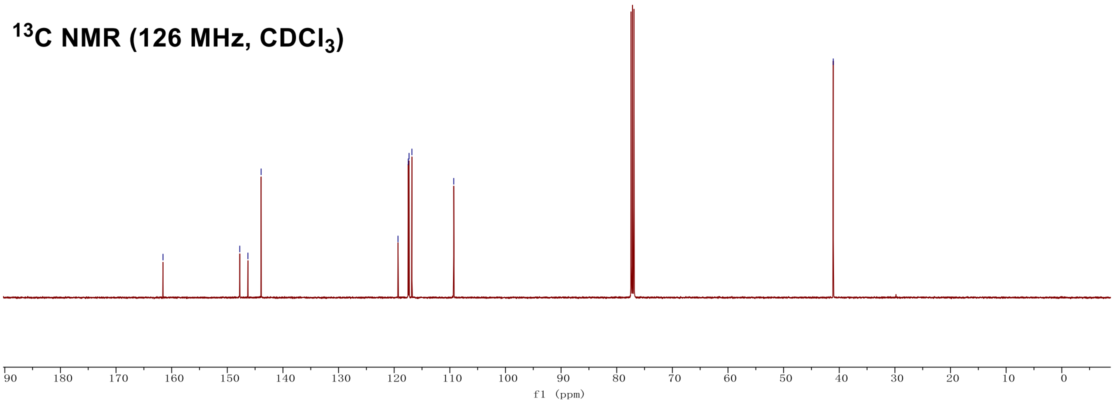

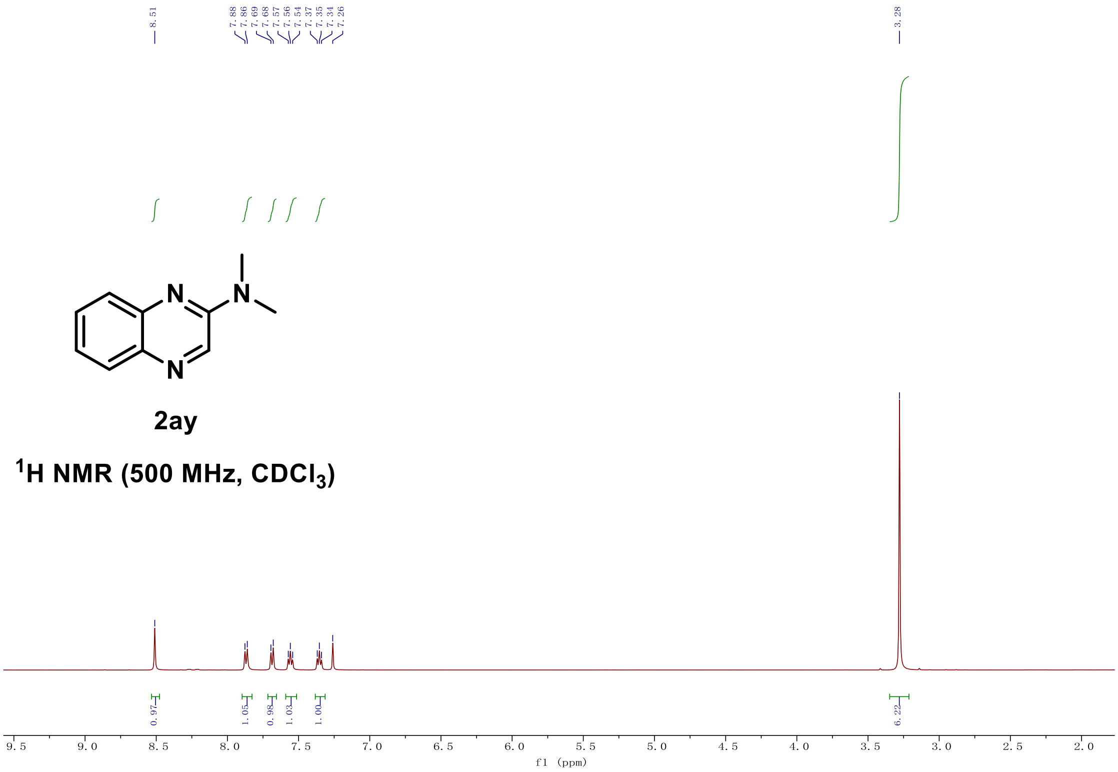


**2ay**

**$^{1}$H NMR (500 MHz, CDCl$_3$)**

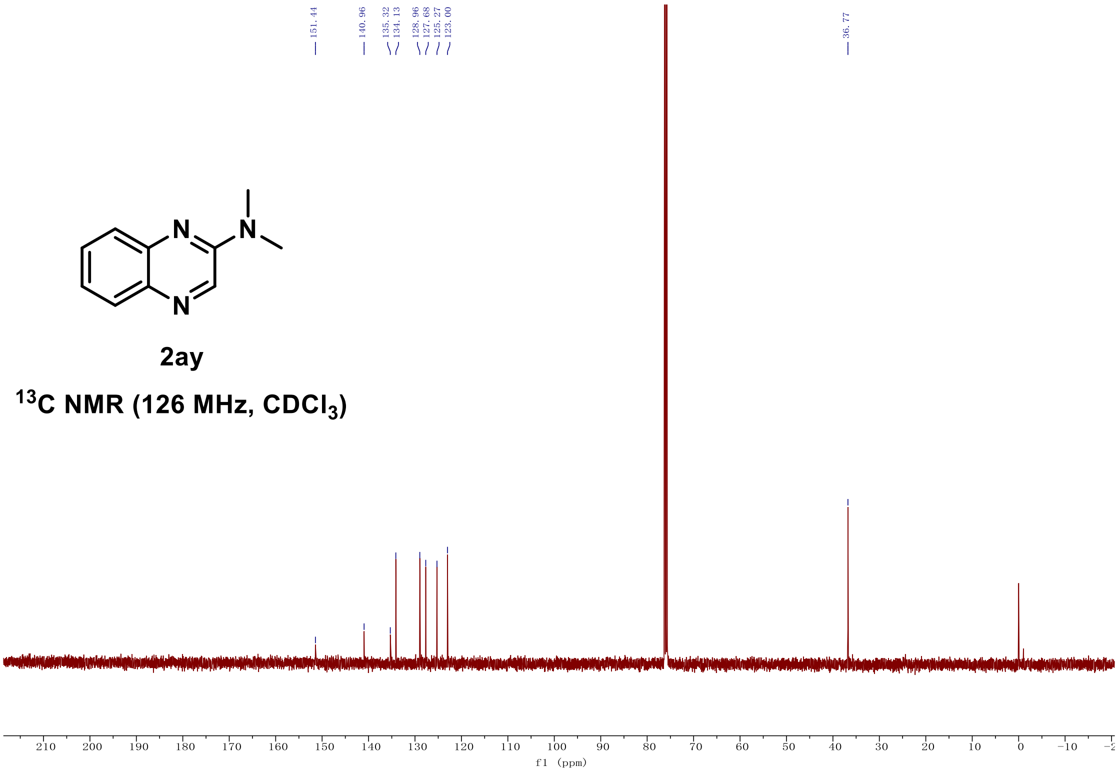


**2ay**

**$^{13}$C NMR (126 MHz, CDCl$_3$)**

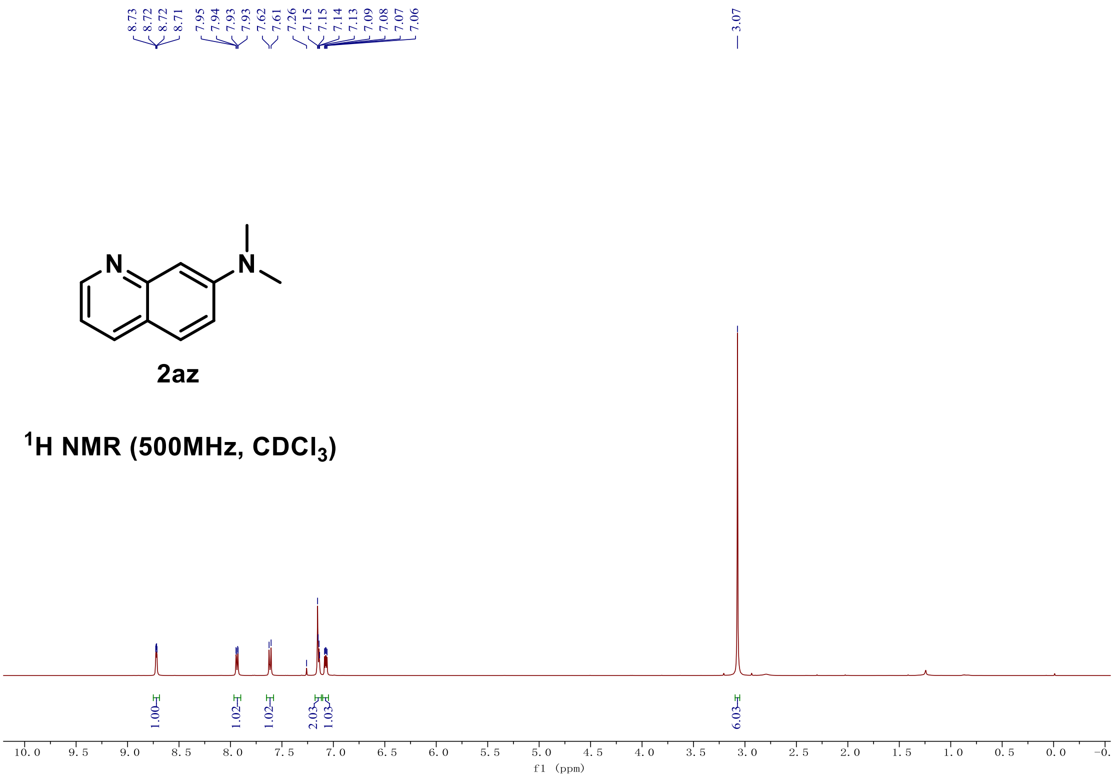

8.73 8.72 8.72 8.71 7.95 7.94 7.93 7.93 7.62 7.61 7.26 7.15 7.15 7.14 7.13 7.09 7.08 7.07 7.06
3.07
2az
$^{1}$H NMR (500MHz, CDCl$_3$)
1.00 1.02 1.02 2.03 1.03 6.03
f1 (ppm)


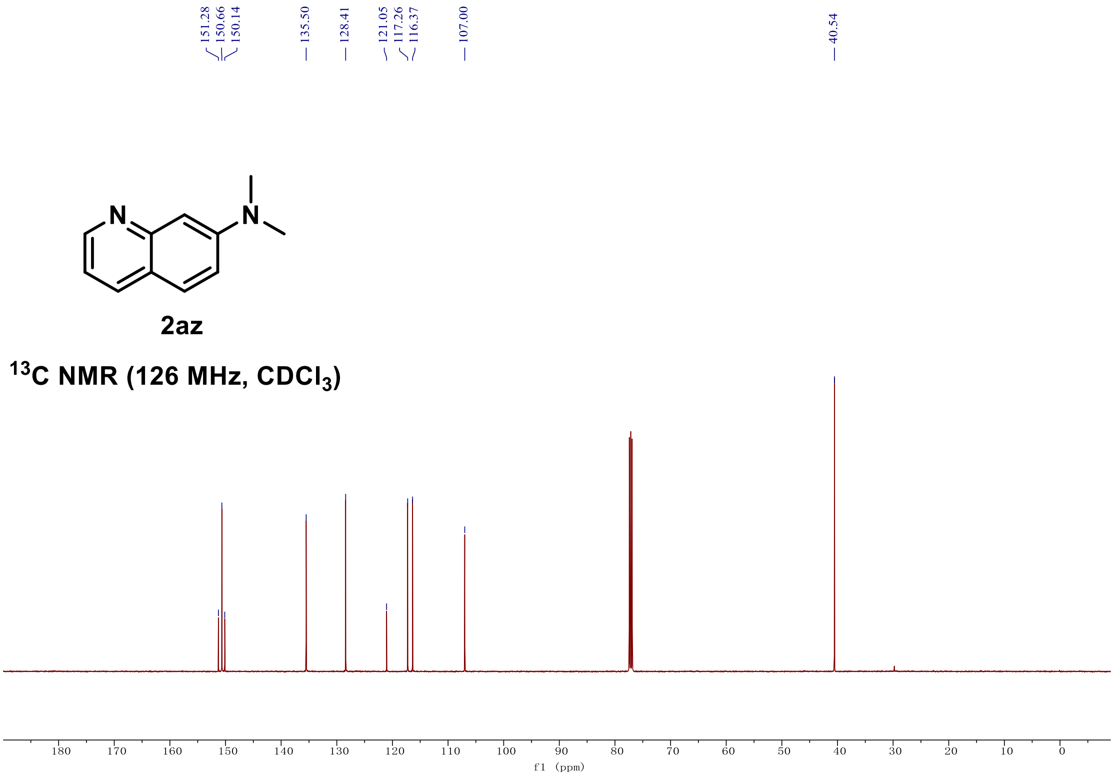

151.28 150.66 150.14 135.50 128.41 121.05 117.26 116.37 107.00
40.54
2az
$^{13}$C NMR (126 MHz, CDCl$_3$)
f1 (ppm)

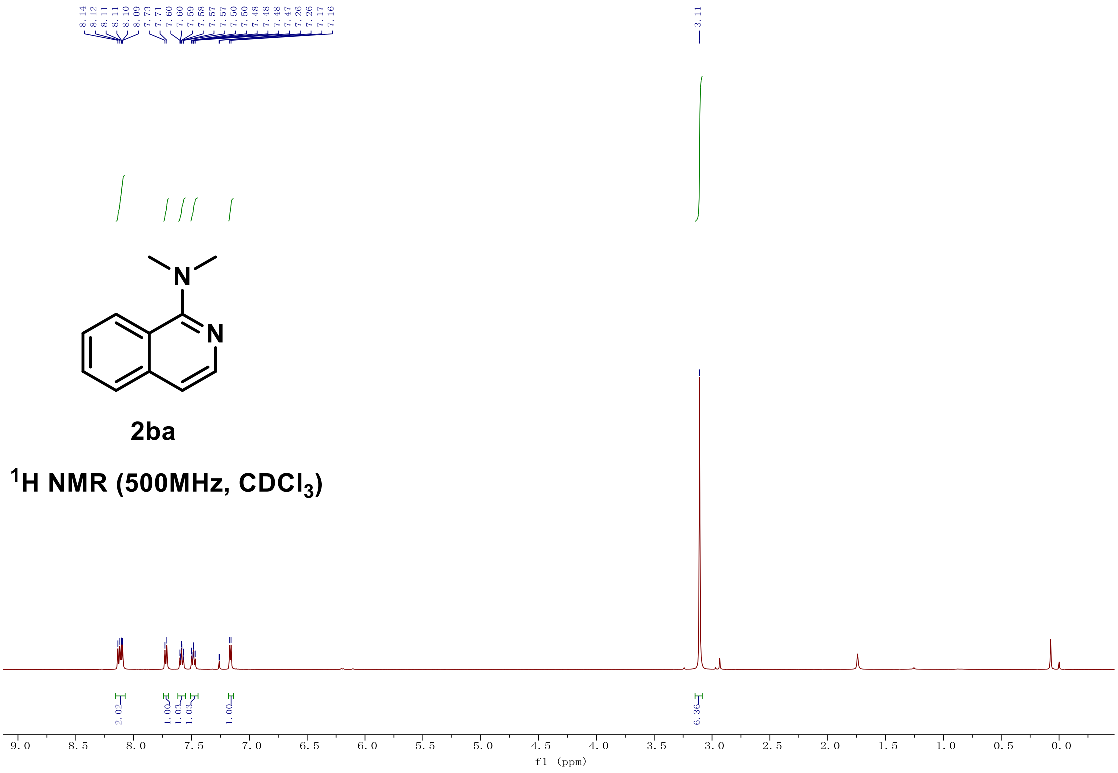
8.14
8.12
8.11
8.11
8.10
8.09
7.73
7.71
7.60
7.60
7.59
7.58
7.57
7.57
7.50
7.50
7.48
7.48
7.48
7.47
7.26
7.26
7.17
7.16
3.11
N
N
2ba
1H NMR (500MHz, CDCl3)
2.02
1.00
1.03
1.03
1.00
6.36
9.0
8.5
8.0
7.5
7.0
6.5
6.0
5.5
5.0
4.5
4.0
3.5
3.0
2.5
2.0
1.5
1.0
0.5
0.0
f1 (ppm)

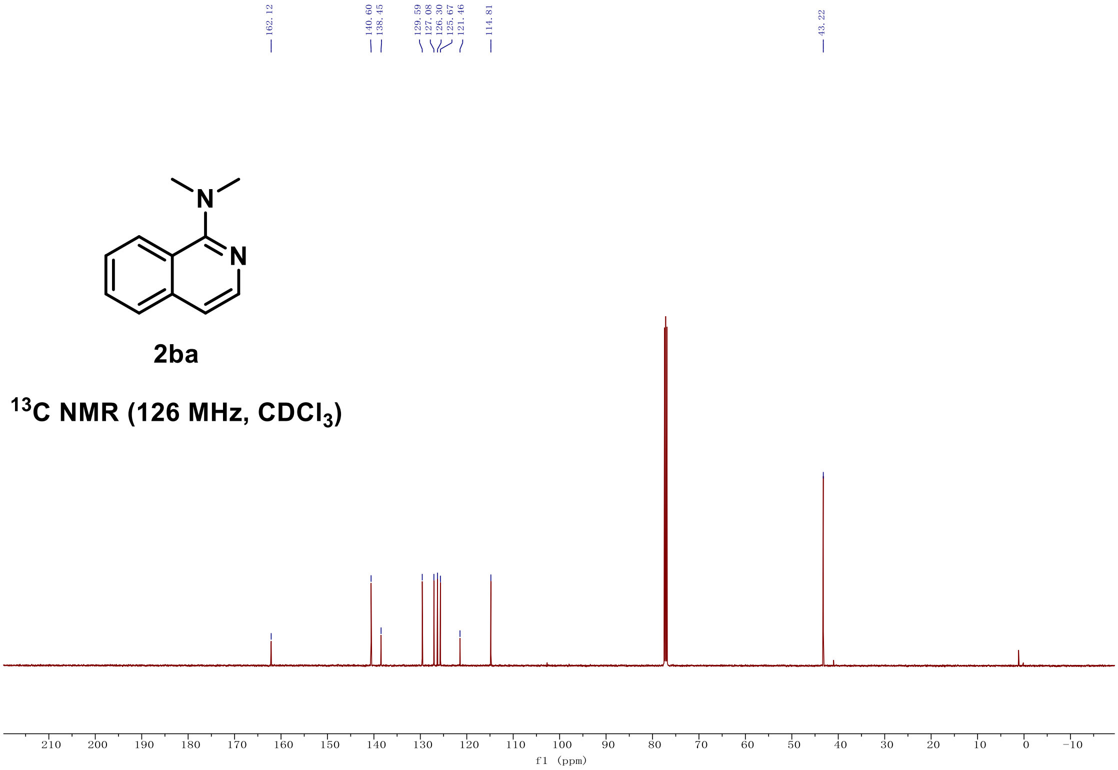
162.12
140.60
138.45
129.59
127.08
126.30
125.67
121.46
114.81
43.22
N
N
2ba
13C NMR (126 MHz, CDCl3)
210
200
190
180
170
160
150
140
130
120
110
100
90
80
70
60
50
40
30
20
10
0
-10
f1 (ppm)

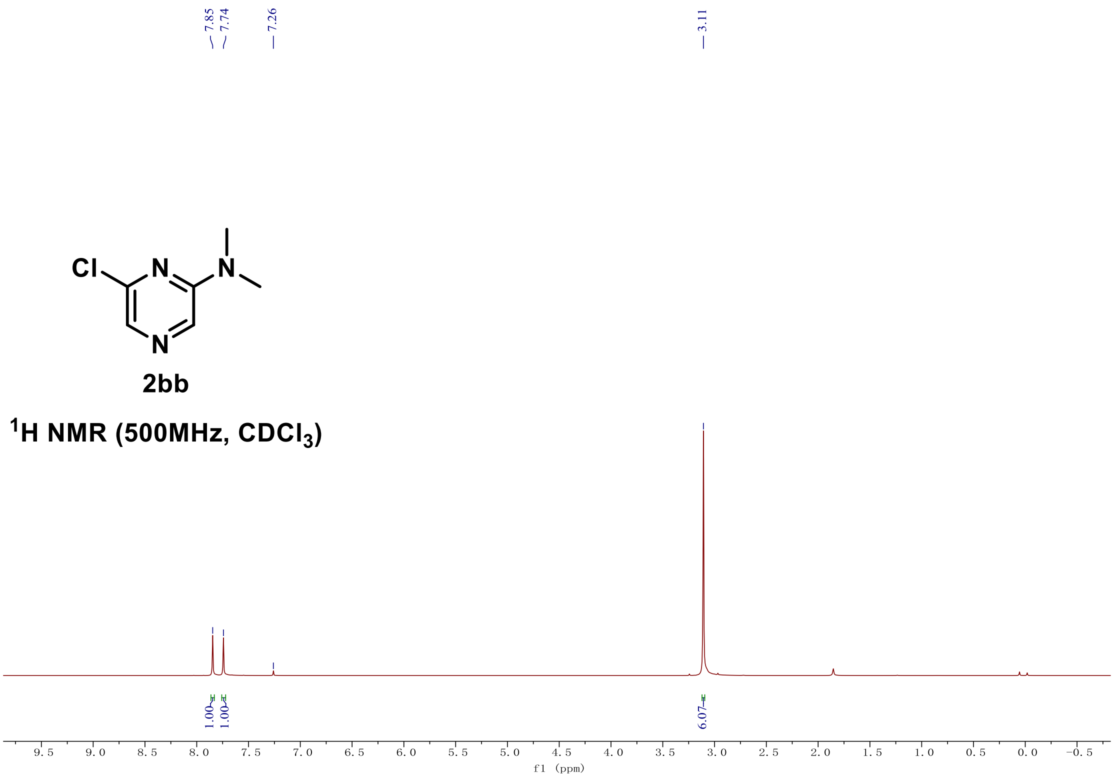
7.85
7.74
7.26
3.11
Cl
N
N
N
2bb
1H NMR (500MHz, CDCl3)
1.00
1.00
6.07
9.5
9.0
8.5
8.0
7.5
7.0
6.5
6.0
5.5
5.0
4.5
4.0
3.5
3.0
2.5
2.0
1.5
1.0
0.5
0.0
-0.5
f1 (ppm)

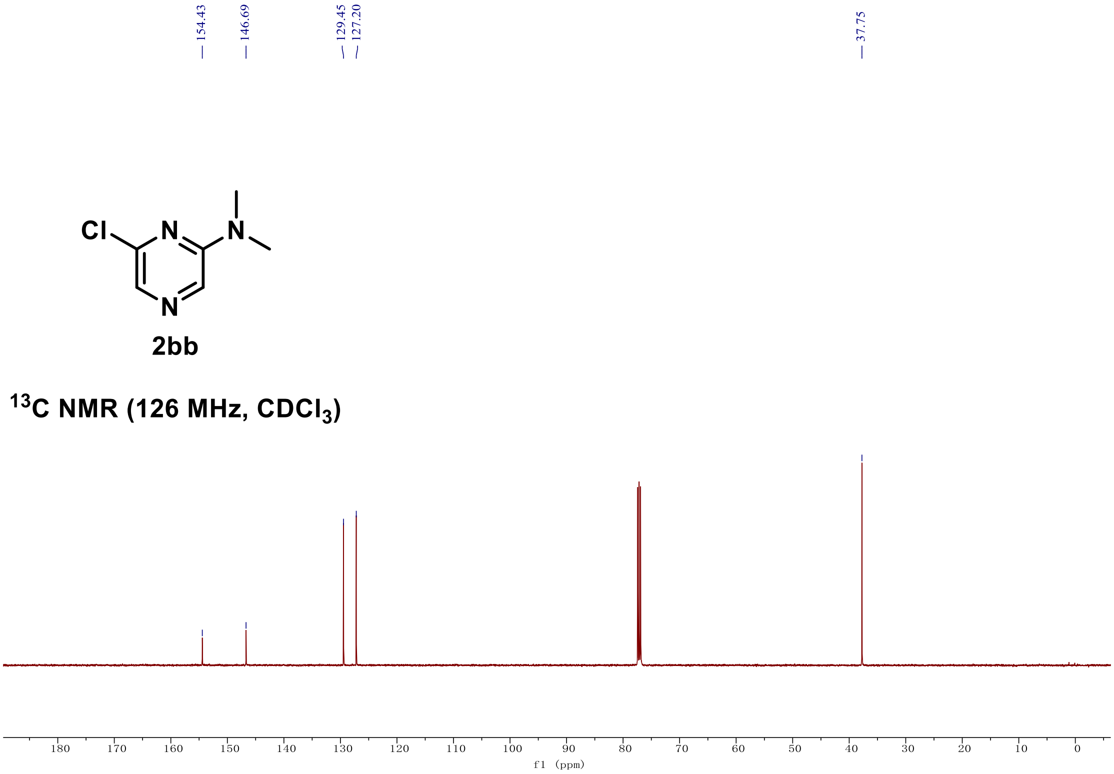
154.43
146.69
129.45
127.20
37.75
Cl
N
N
N
2bb
13C NMR (126 MHz, CDCl3)
180
170
160
150
140
130
120
110
100
90
80
70
60
50
40
30
20
10
0
f1 (ppm)

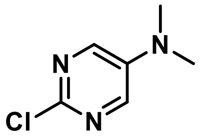

**2bc**

**$^1$H NMR (500MHz, $CDCl_3$)**

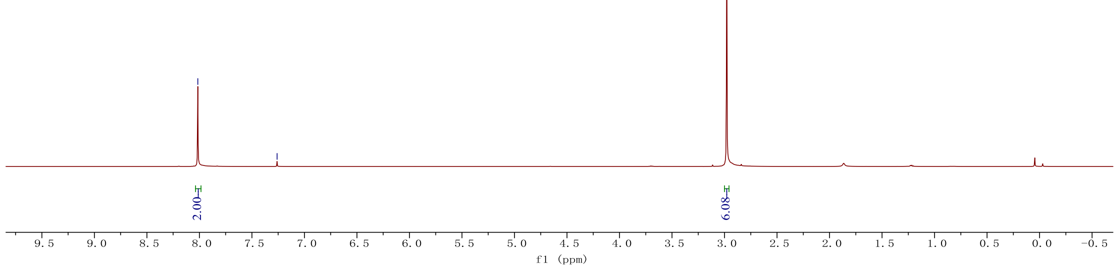


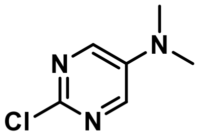

**2bc**

**$^{13}$C NMR (126 MHz, $CDCl_3$)**

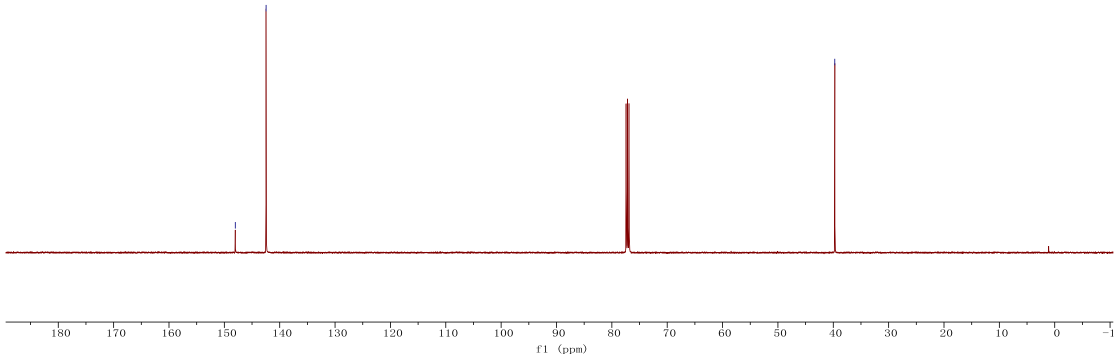

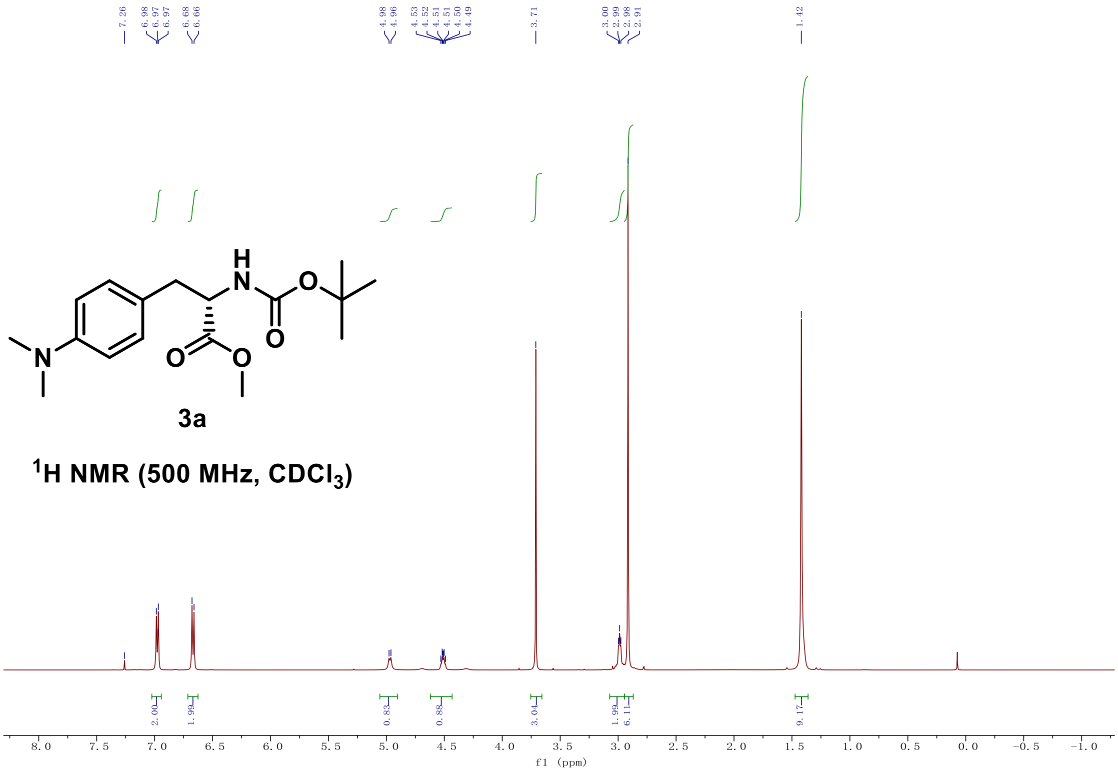

3a

$^{1}$H NMR (500 MHz, $CDCl_3$)

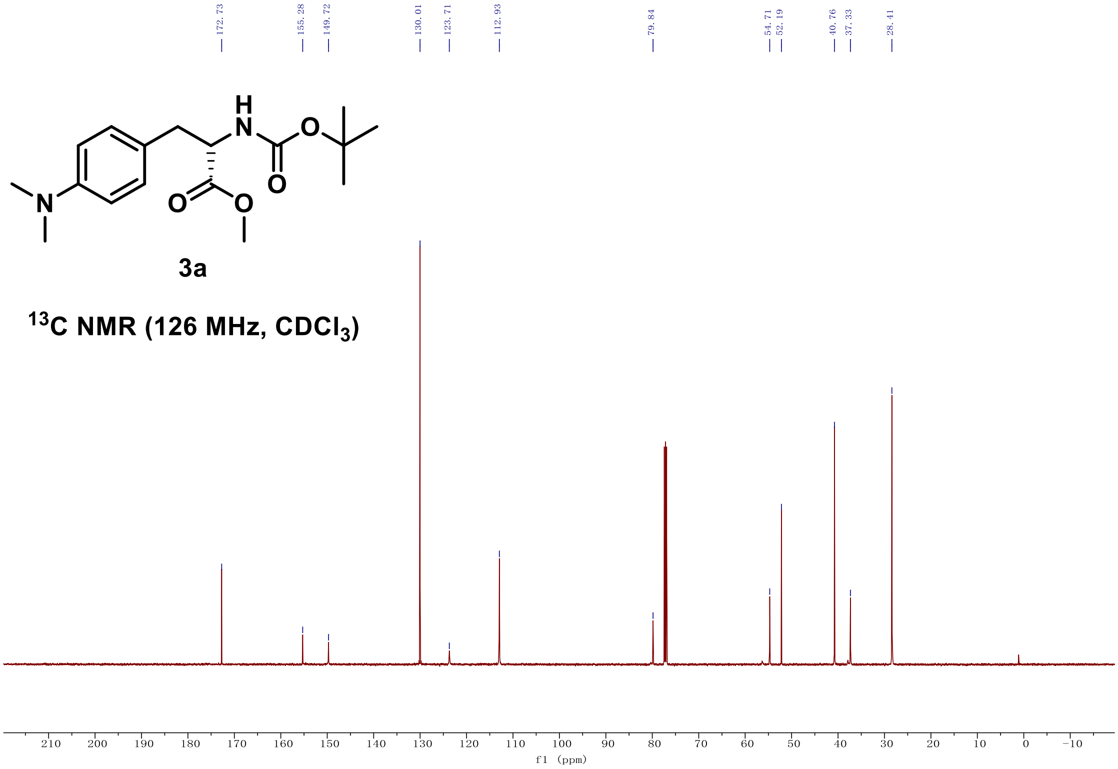

3a

$^{13}$C NMR (126 MHz, $CDCl_3$)

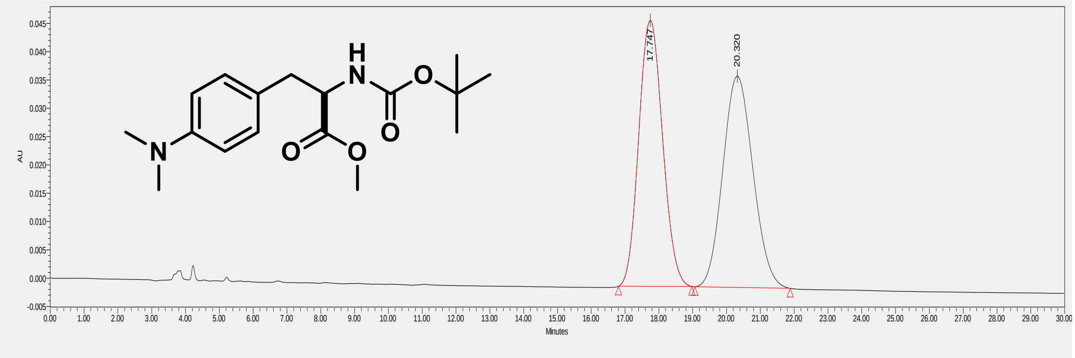


| Name | Retention Time | Area | % Area | Height |
|---|---|---|---|---|
| 1 | 17.747 | 2305947 | 49.91 | 46977 |
| 2 | 20.320 | 2314539 | 50.09 | 37334 |

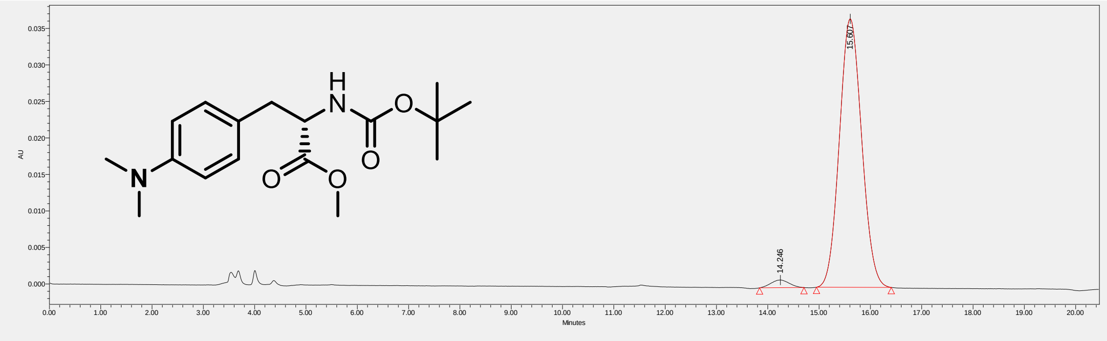


| Name | Retention Time | Area | % Area | Height |
|---|---|---|---|---|
| 1 | 14.246 | 25953 | 2.28 | 1052 |
| 2 | 15.607 | 1110758 | 97.72 | 36760 |

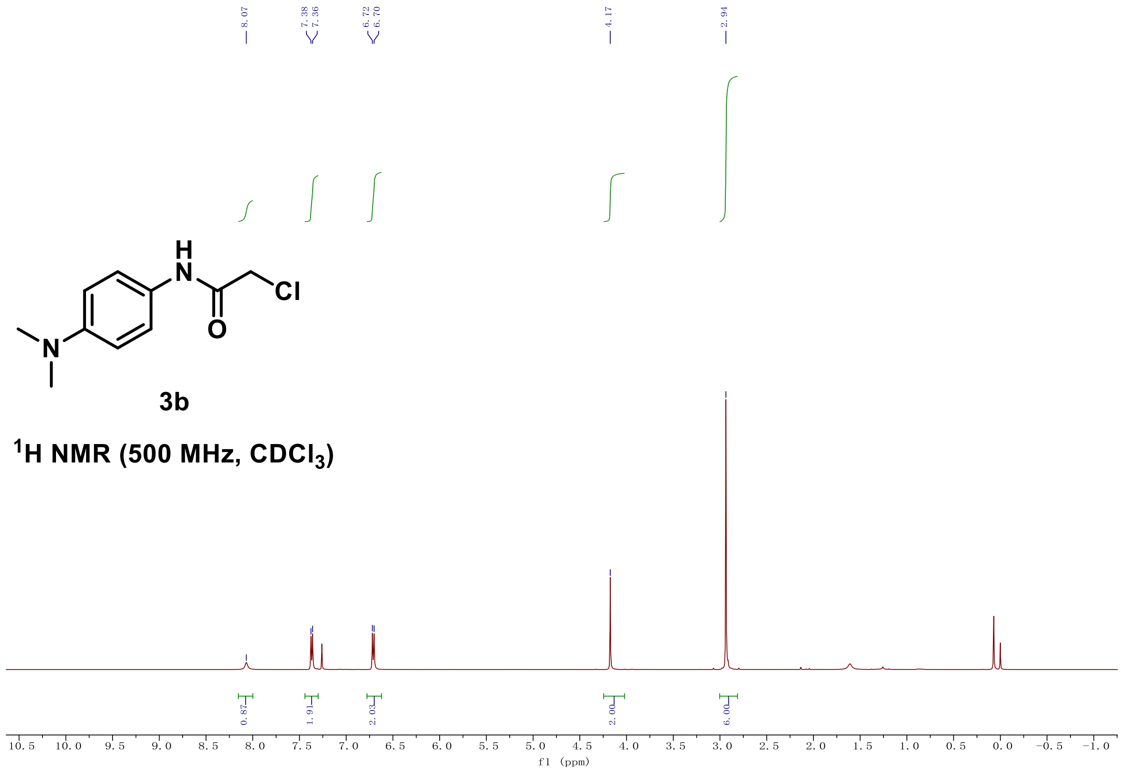
8.07
7.38
7.36
6.72
6.70
4.17
2.94
H
N
Cl
O
N
3b
$^{1}$H NMR (500 MHz, $CDCl_3$)
0.87
1.91
2.03
2.00
6.00
10.5 10.0 9.5 9.0 8.5 8.0 7.5 7.0 6.5 6.0 5.5 5.0 4.5 4.0 3.5 3.0 2.5 2.0 1.5 1.0 0.5 0.0 -0.5 -1.0
f1 (ppm)

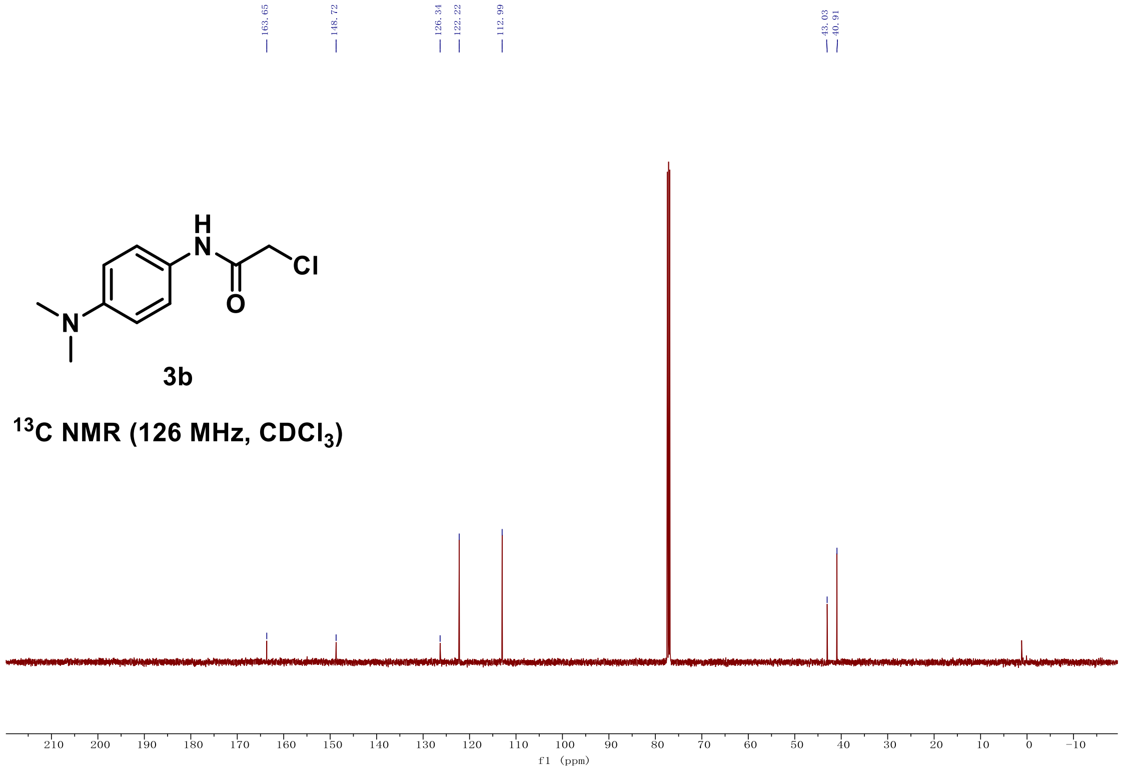
163.65
148.72
126.34
122.22
112.99
43.03
40.91
H
N
Cl
O
N
3b
$^{13}$C NMR (126 MHz, $CDCl_3$)
210 200 190 180 170 160 150 140 130 120 110 100 90 80 70 60 50 40 30 20 10 0 -10
f1 (ppm)

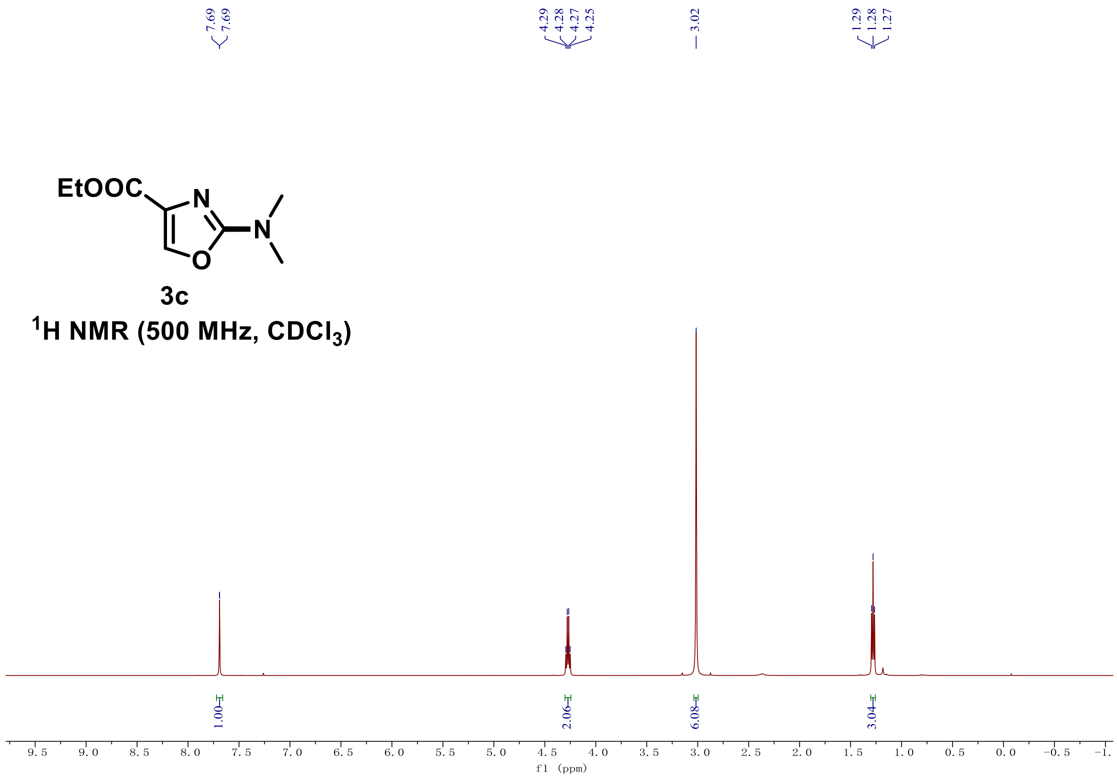
7.69
7.69
4.29
4.28
4.27
4.25
3.02
1.29
1.28
1.27
EtOOC
N
N
O
3c
1H NMR (500 MHz, CDCl3)
1.00
2.06
6.08
3.04
9.5 9.0 8.5 8.0 7.5 7.0 6.5 6.0 5.5 5.0 4.5 4.0 3.5 3.0 2.5 2.0 1.5 1.0 0.5 0.0 −0.5
f1 (ppm)

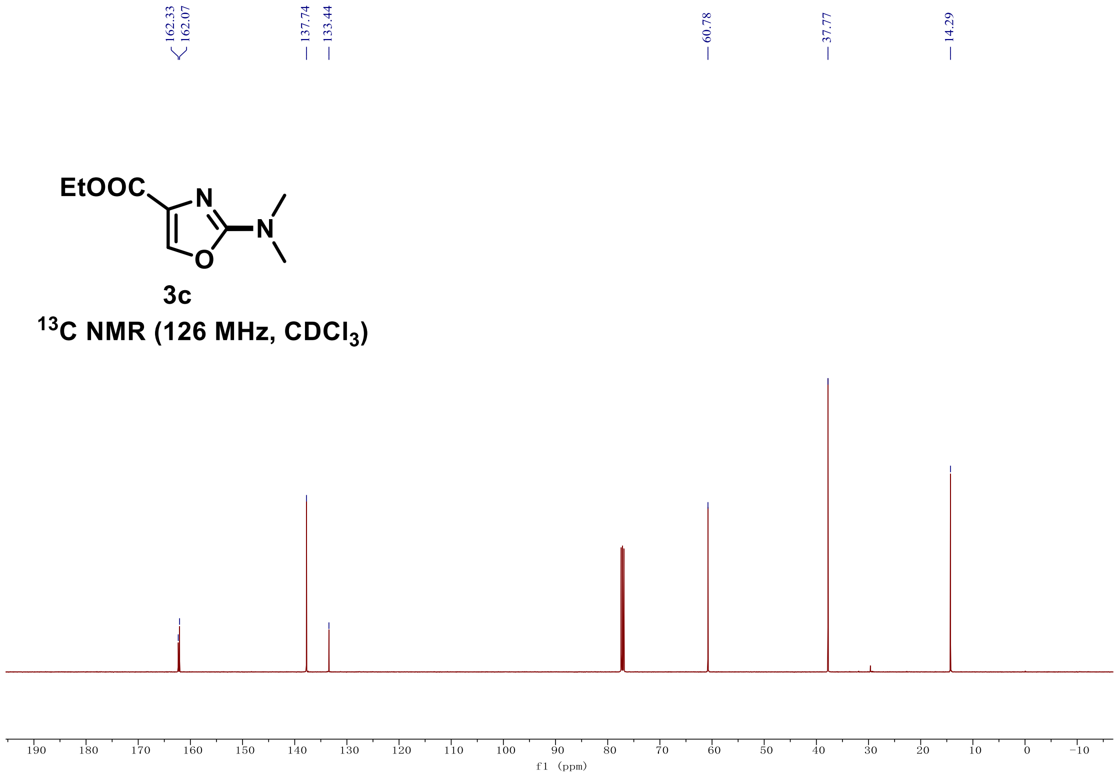
162.33
162.07
137.74
133.44
60.78
37.77
14.29
EtOOC
N
N
O
3c
13C NMR (126 MHz, CDCl3)
190 180 170 160 150 140 130 120 110 100 90 80 70 60 50 40 30 20 10 0 −10
f1 (ppm)

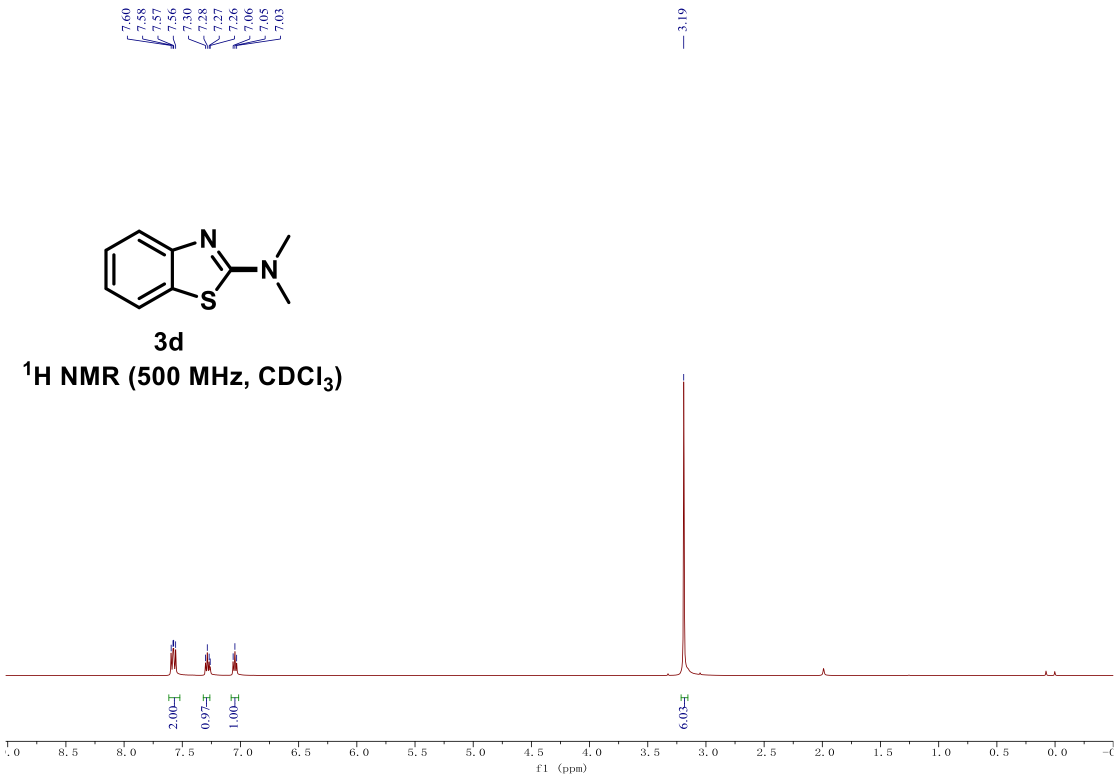
7.60
7.58
7.57
7.56
7.30
7.28
7.27
7.26
7.06
7.05
7.03
3.19
3d
$^{1}$H NMR (500 MHz, $CDCl_3$)
2.00
0.97
1.00
6.03
f1 (ppm)

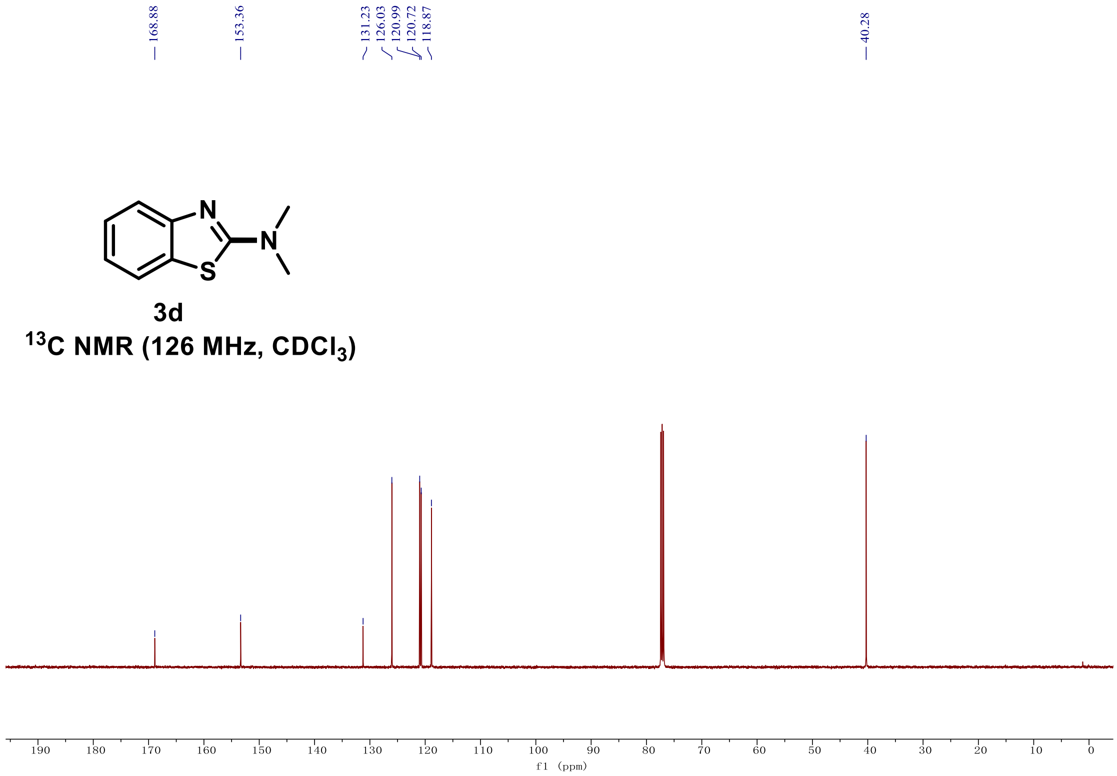
168.88
153.36
131.23
126.03
120.99
120.72
118.87
40.28
3d
$^{13}$C NMR (126 MHz, $CDCl_3$)
f1 (ppm)

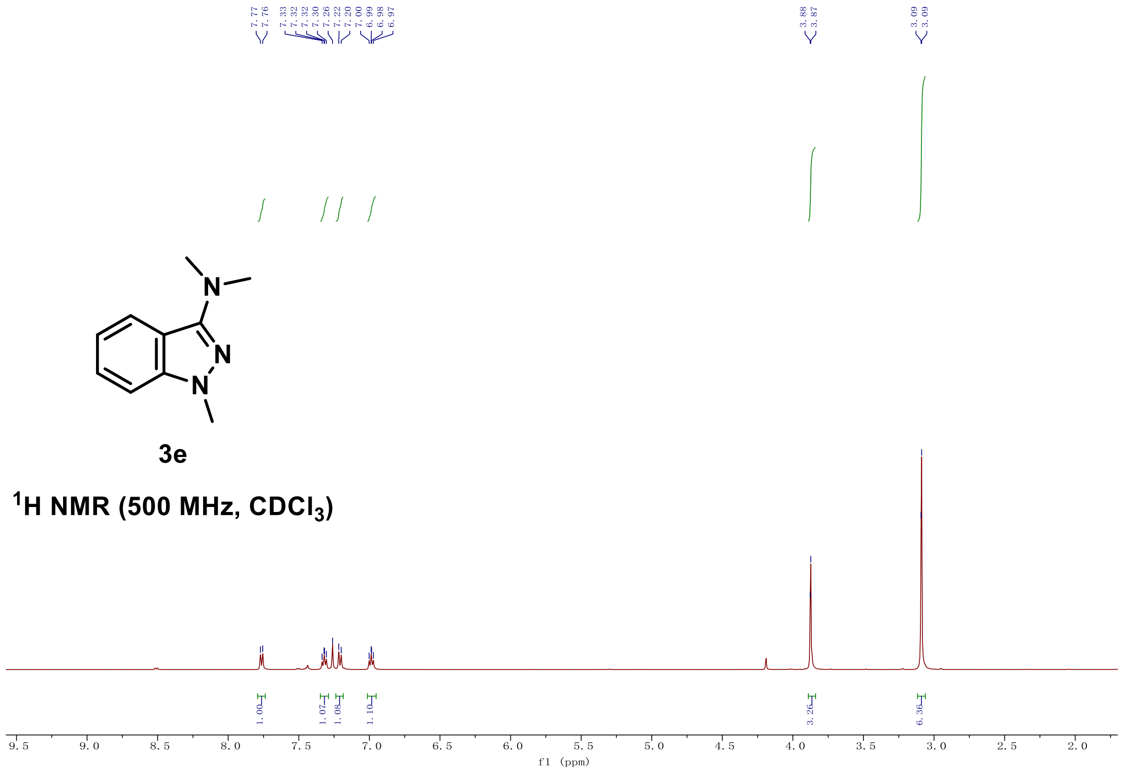
7.77
7.76
7.33
7.32
7.32
7.30
7.26
7.22
7.20
7.00
6.99
6.98
6.97
3.88
3.87
3.09
3.09
3e
$^1$H NMR (500 MHz, $CDCl_3$)
1.00
1.07
1.08
1.10
3.26
6.36
f1 (ppm)

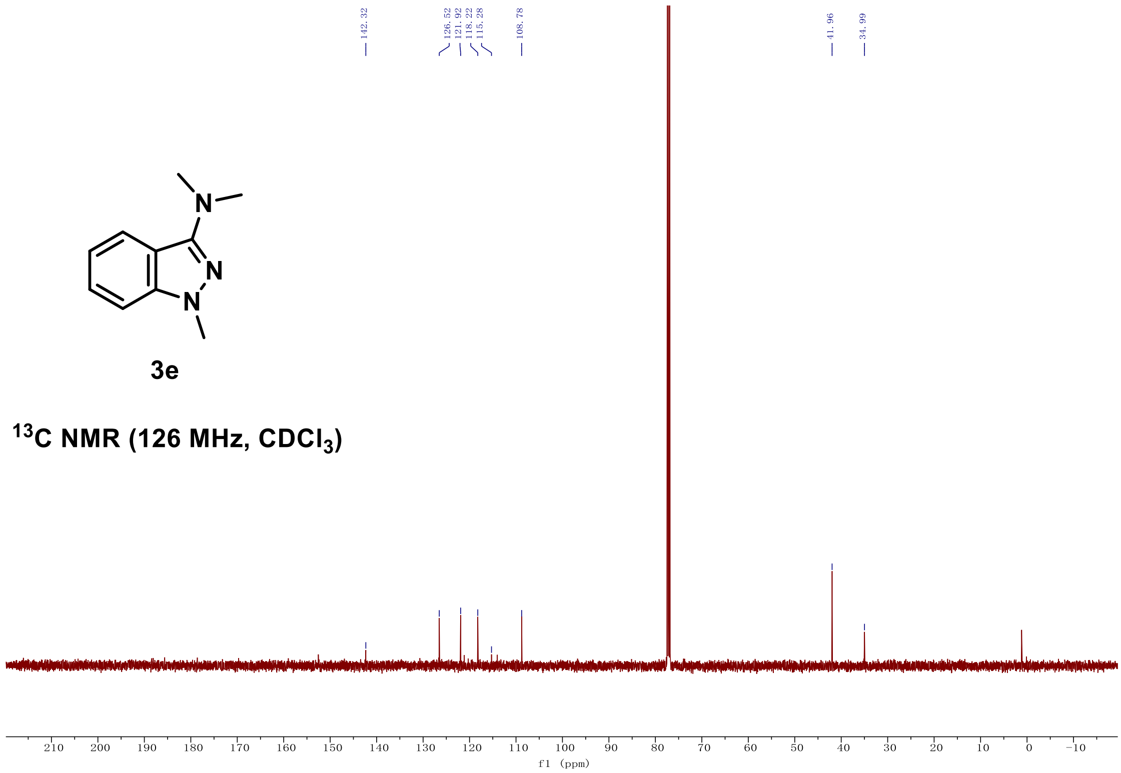
142.32
126.52
121.92
118.22
115.28
108.78
41.96
34.99
3e
$^{13}$C NMR (126 MHz, $CDCl_3$)
f1 (ppm)

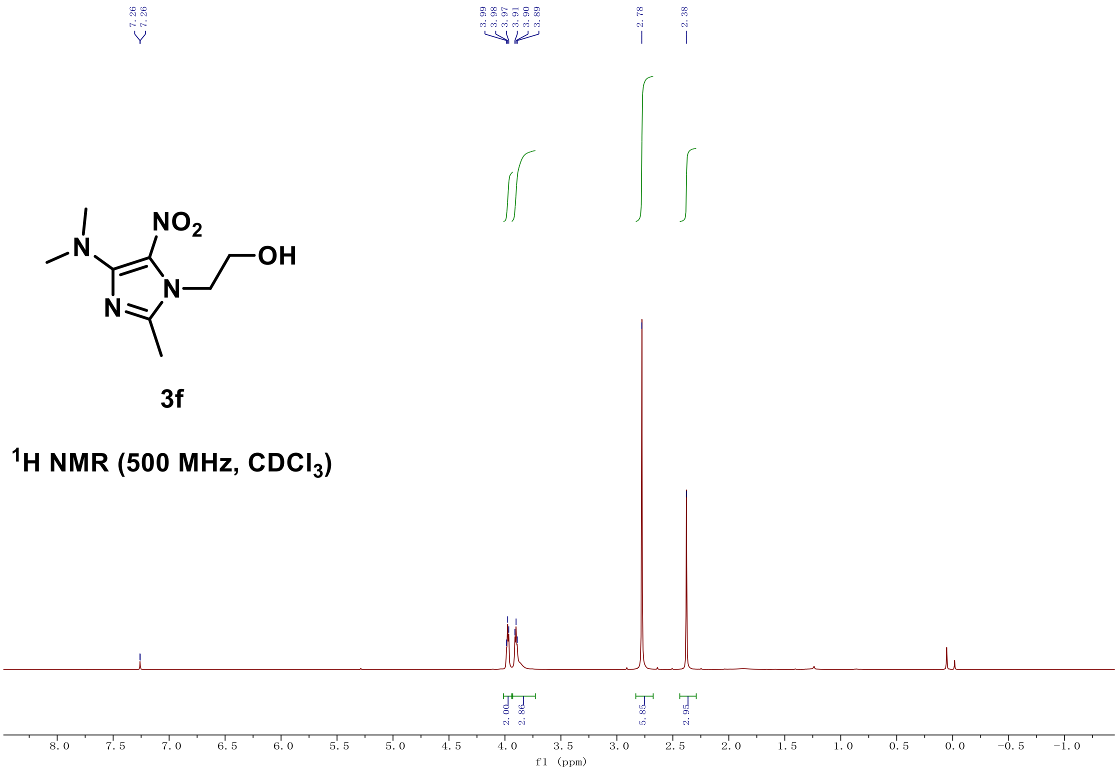
7.26
7.26
3.99
3.98
3.97
3.91
3.90
3.89
2.78
2.38
NO2
N
OH
3f
1H NMR (500 MHz, CDCl3)
2.00
2.86
5.85
2.95
f1 (ppm)

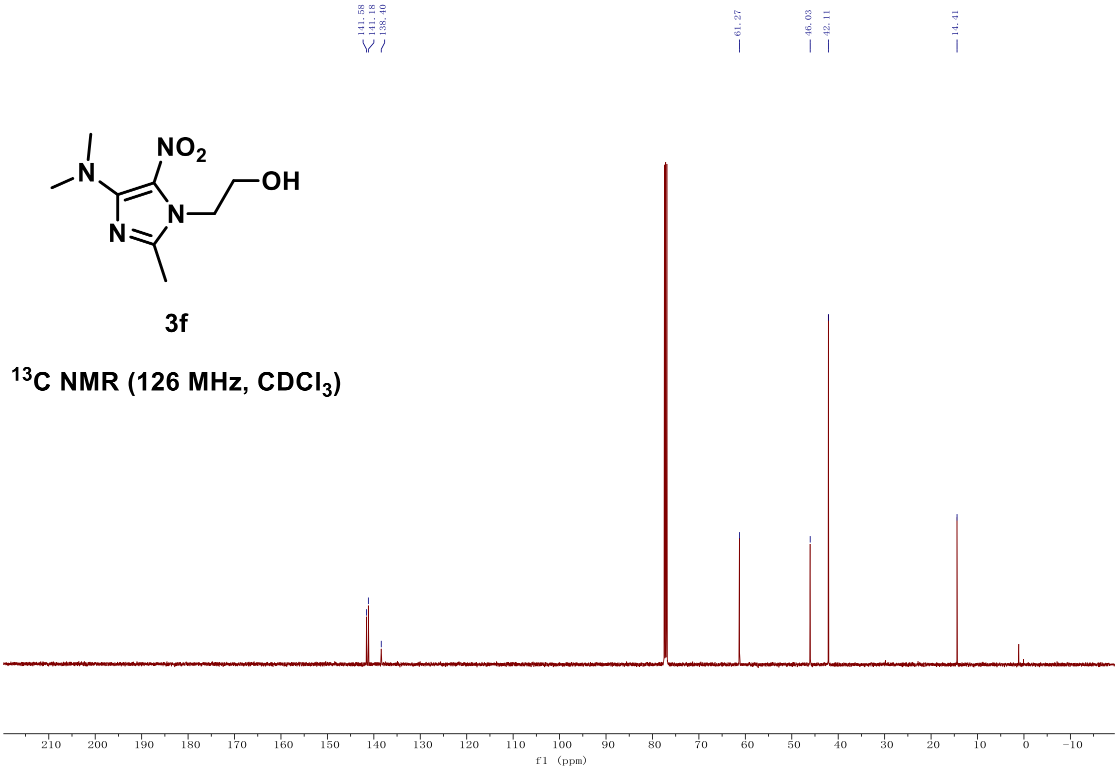
141.58
141.18
138.40
61.27
46.03
42.11
14.41
NO2
N
OH
3f
13C NMR (126 MHz, CDCl3)
f1 (ppm)

**s3b**

**$^{1}$H NMR (500MHz, $CDCl_3$)**

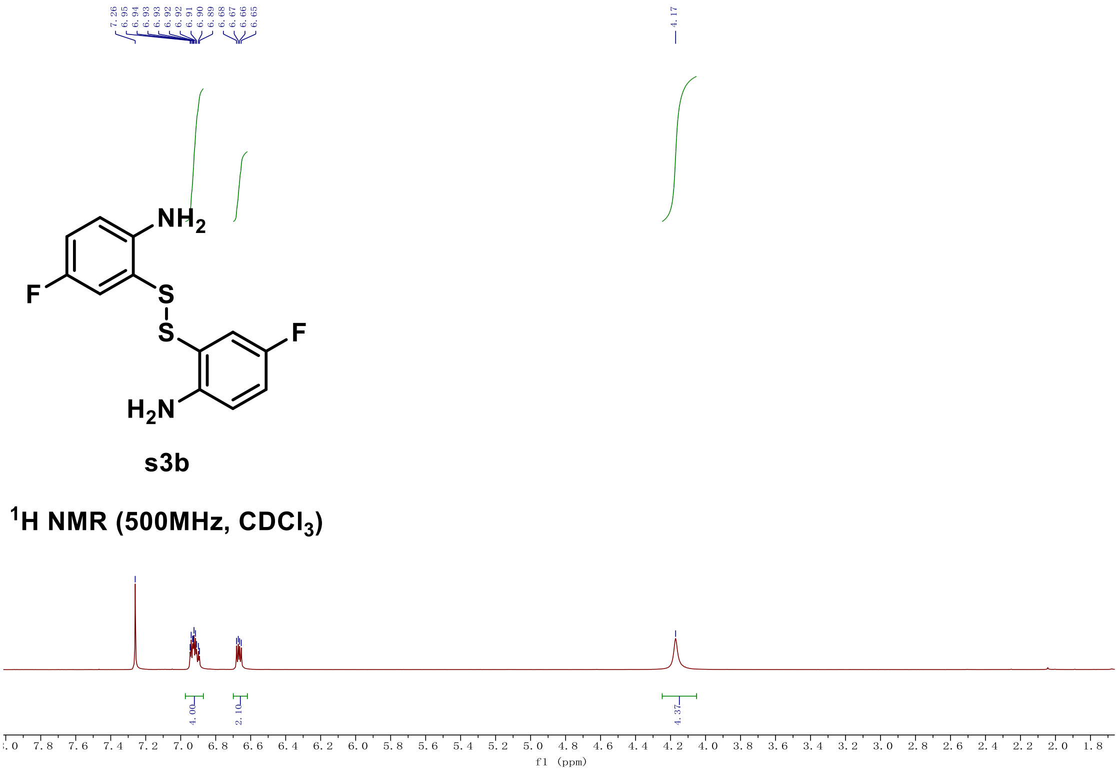


**s3c**

**$^{1}$H NMR (500MHz, $CDCl_3$)**

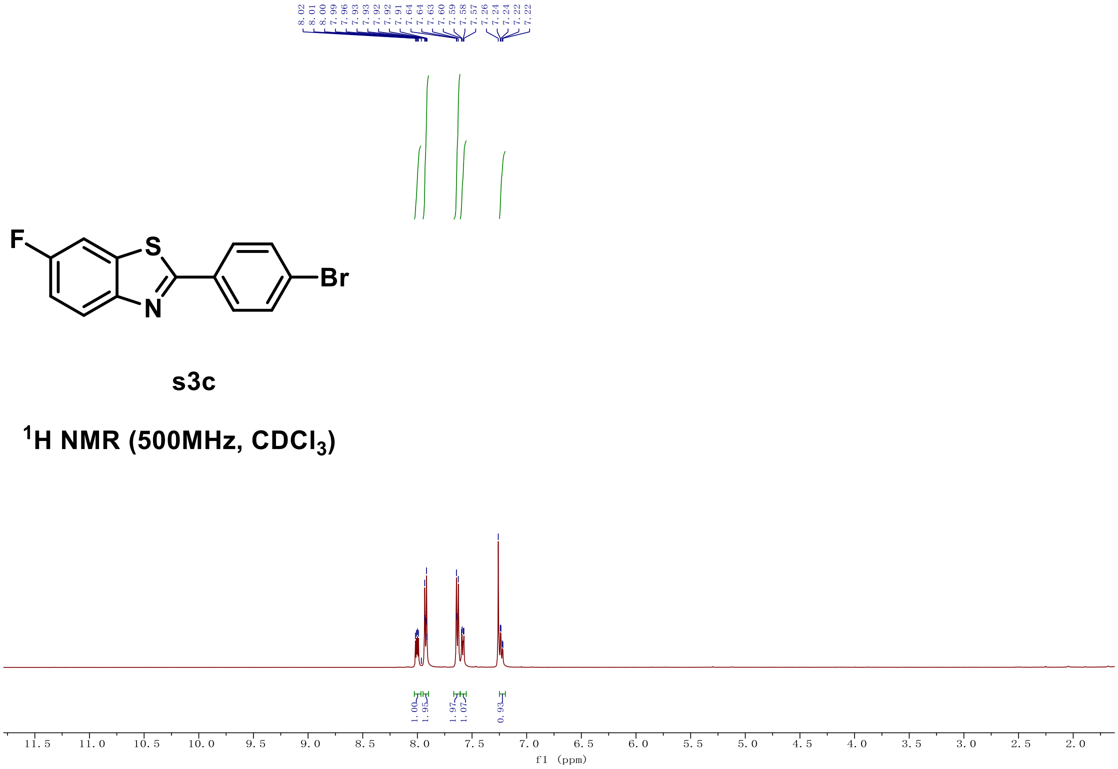

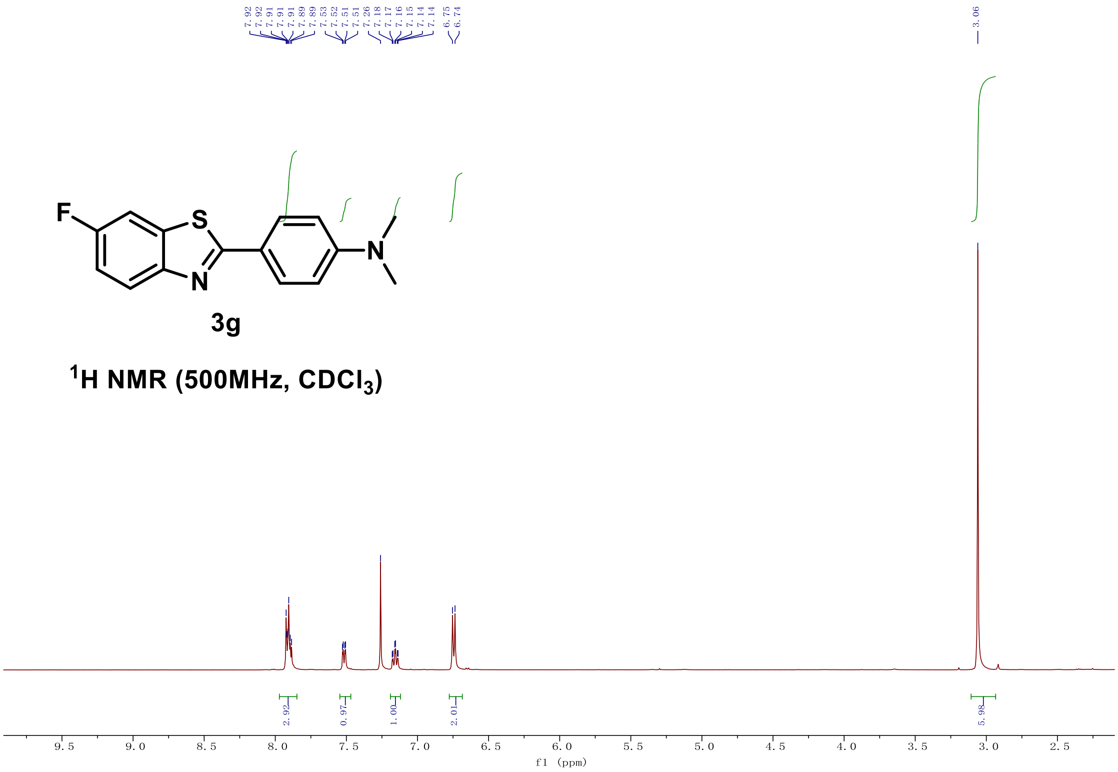


**3g**

**$^{1}H$ NMR (500MHz, $CDCl_3$)**

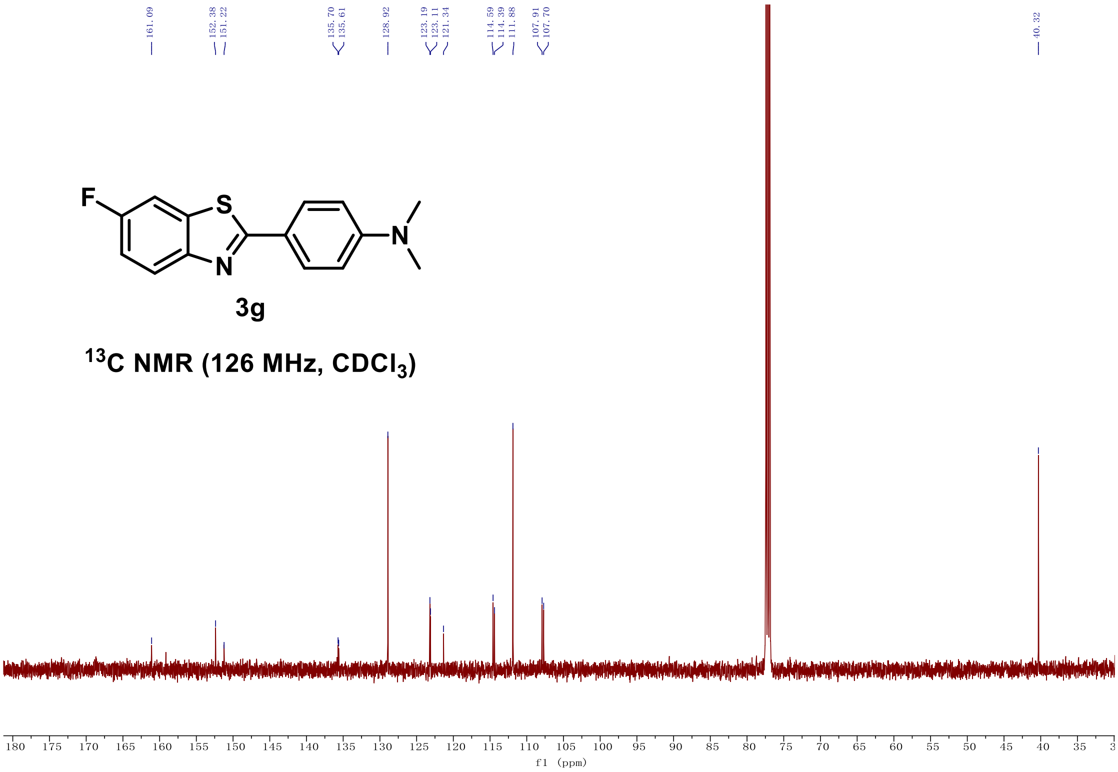


**3g**

**$^{13}C$ NMR (126 MHz, $CDCl_3$)**

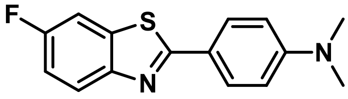


**3g**

**$^{19}$F NMR (471 MHz, $CDCl_3$)**

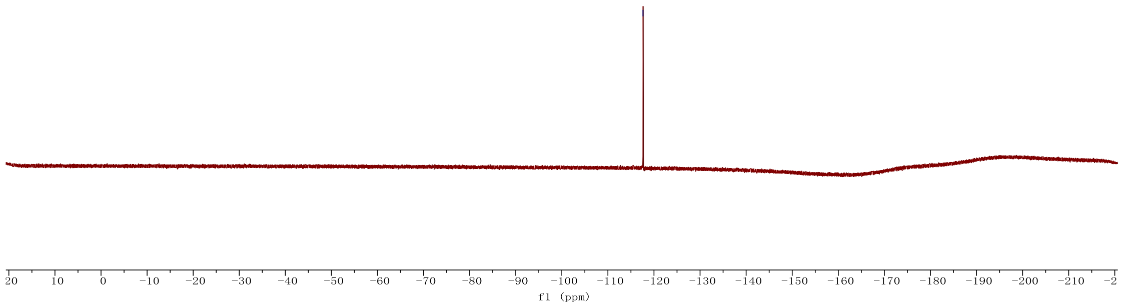


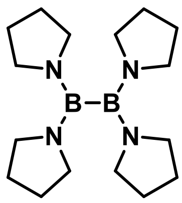


**s4a**

**$^{1}$H NMR (500MHz, $CDCl_3$)**

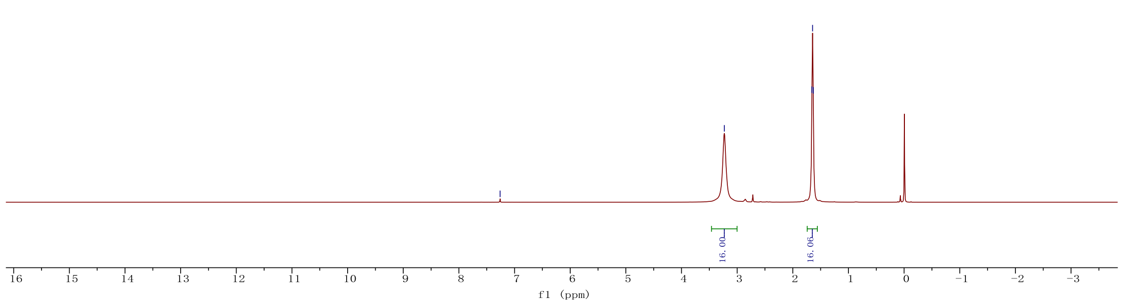

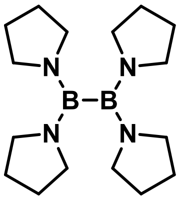


**s4a**

**$^{13}$C NMR (126 MHz, $CDCl_3$)**

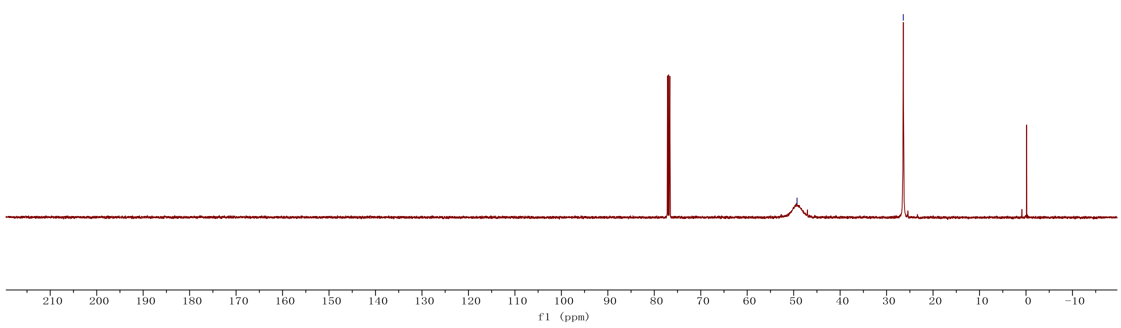


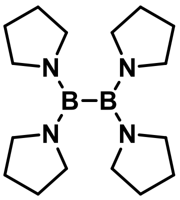


**s4a**

**$^{11}$B NMR (161 MHz, $CDCl_3$)**

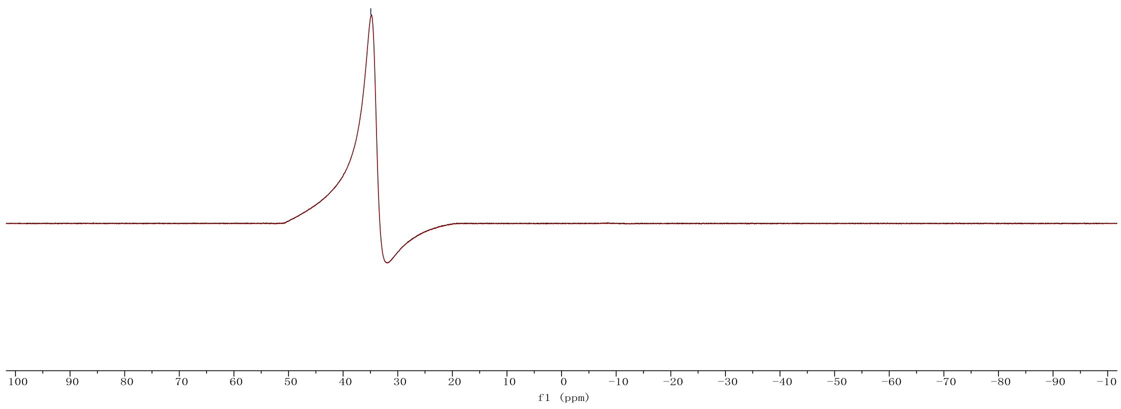

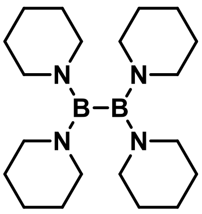

**s4b**

**$^{1}H$ NMR (500MHz, $CDCl_3$)**

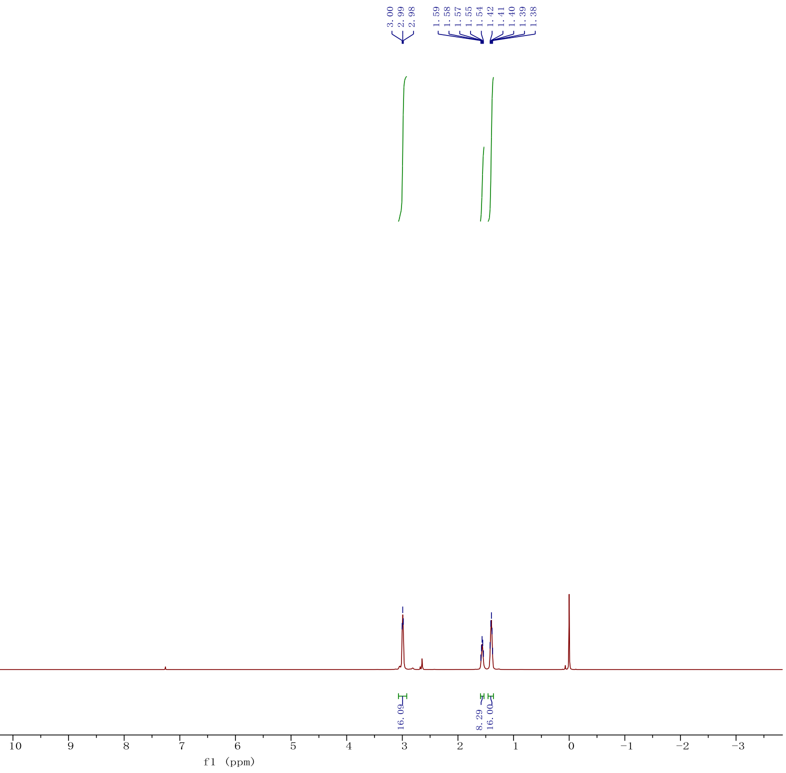


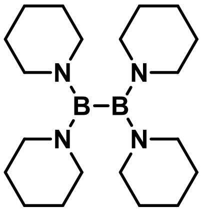

**s4b**

**$^{13}C$ NMR (126 MHz, $CDCl_3$)**

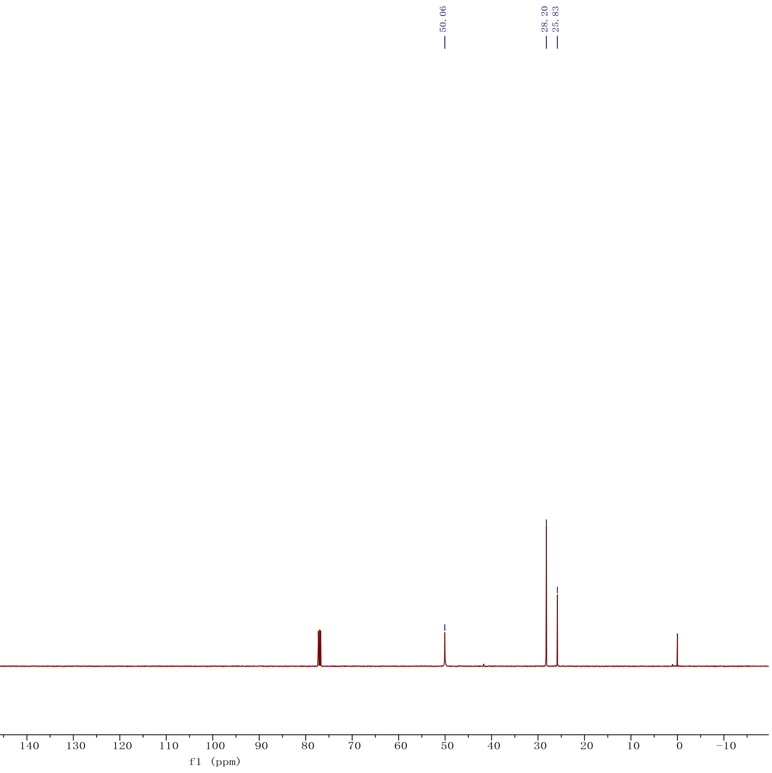

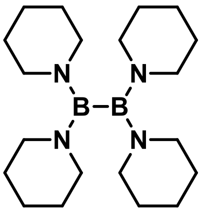

**s4b**

**$^{11}$B NMR (161 MHz, $CDCl_3$)**

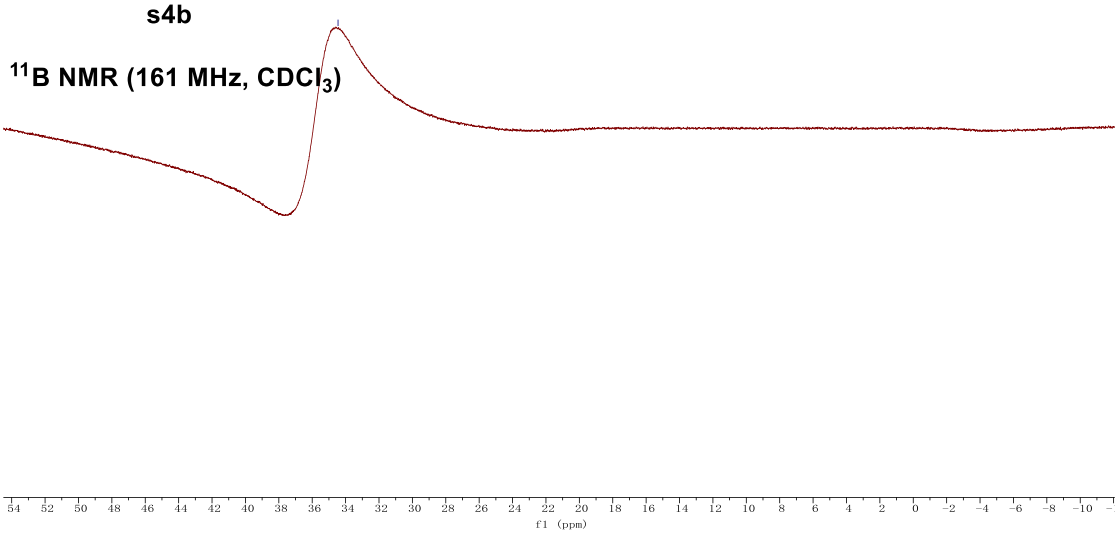

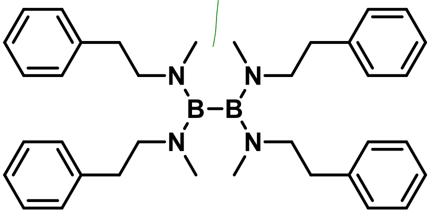

**s4c**

**$^{1}$H NMR (500MHz, DMSO-$d_6$)**

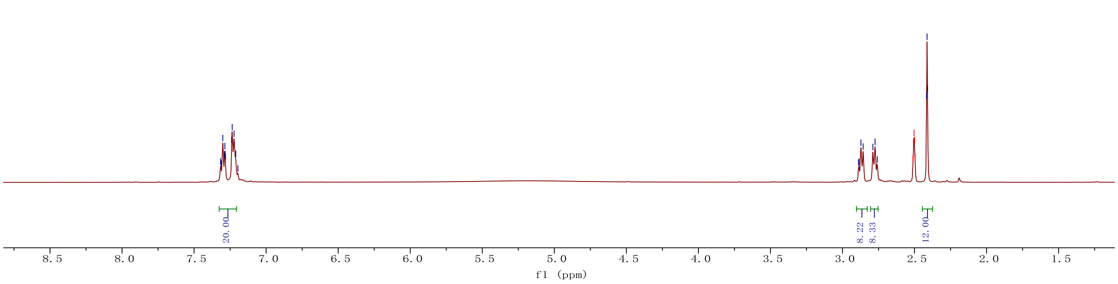

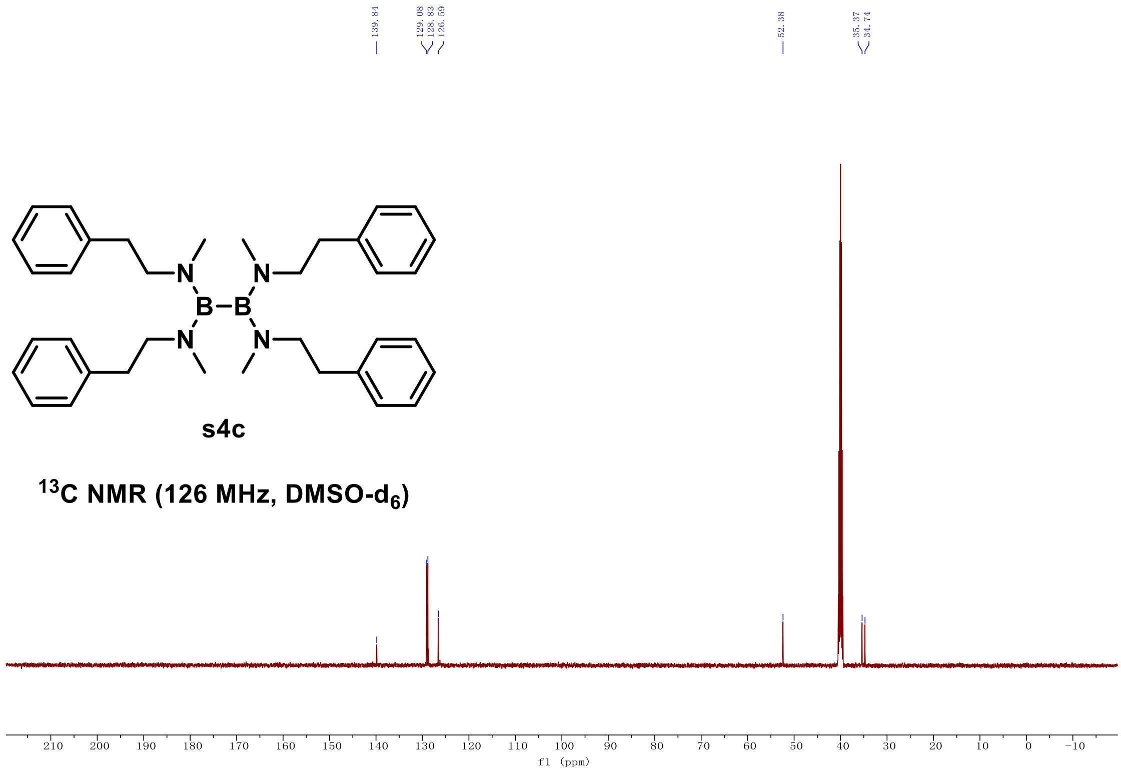
139.84
129.08
128.83
126.59
52.38
35.37
34.74
s4c
$^{13}$C NMR (126 MHz, DMSO-$d_6$)
f1 (ppm)

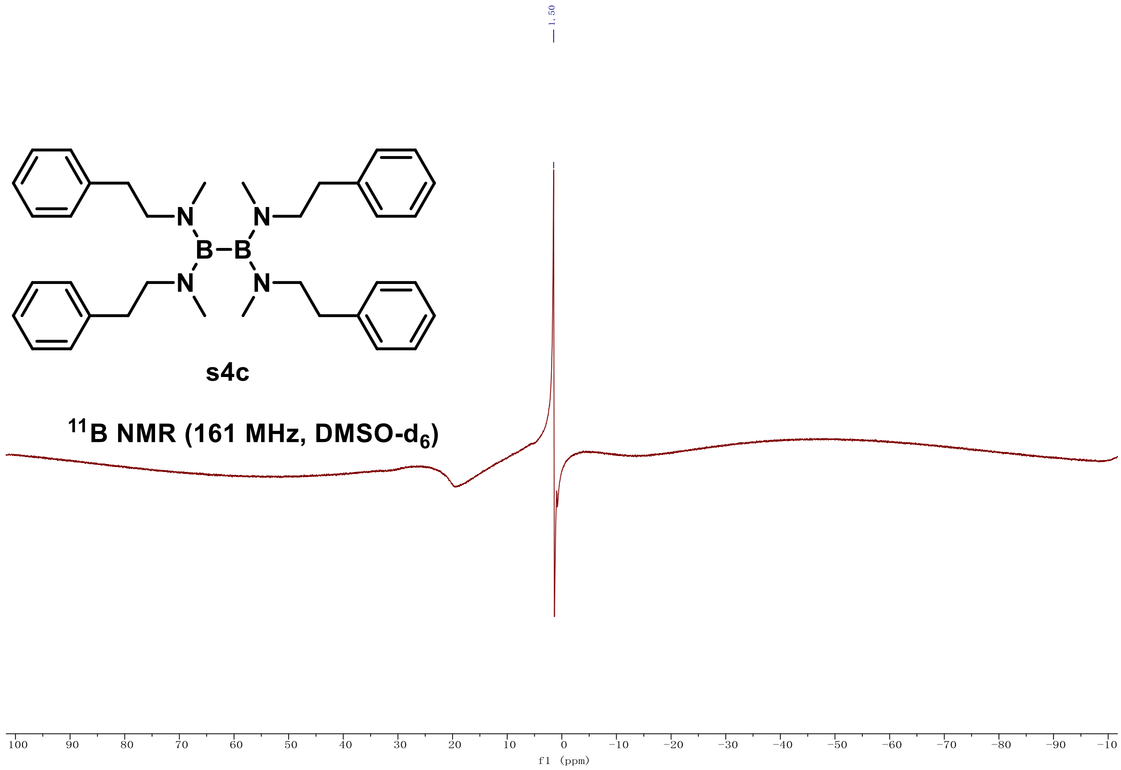
1.50
s4c
$^{11}$B NMR (161 MHz, DMSO-$d_6$)
f1 (ppm)

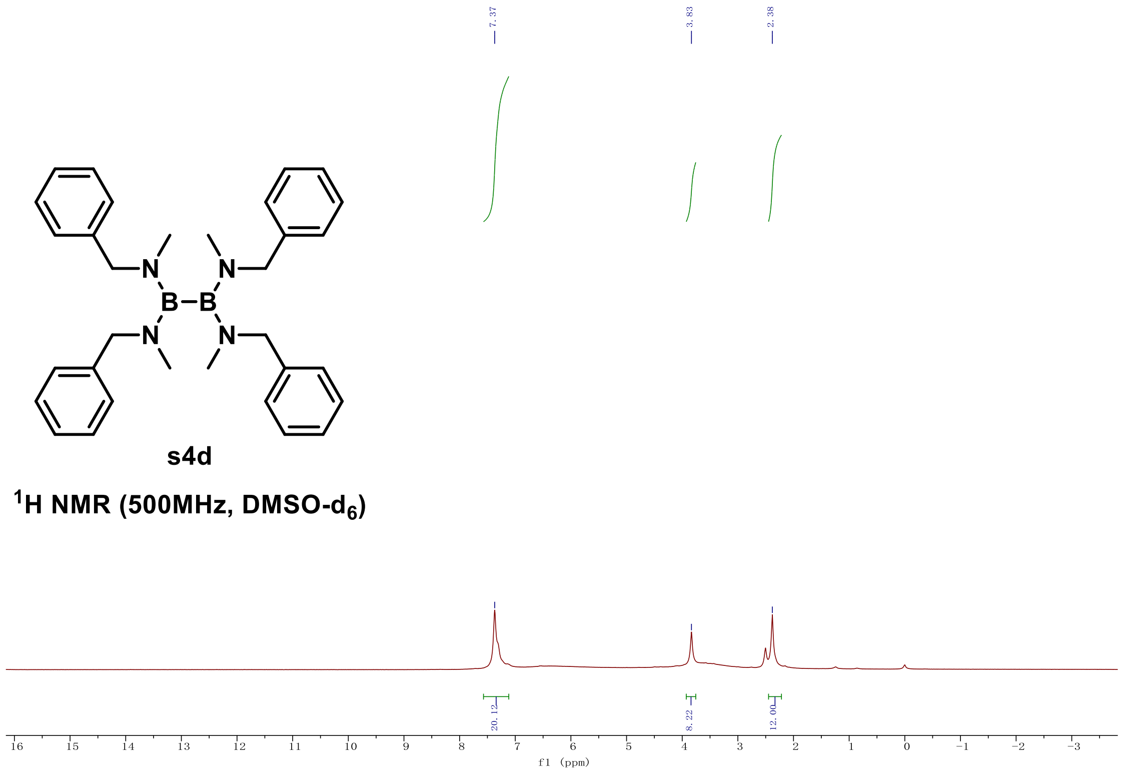


**s4d**

**$^{1}$H NMR (500MHz, DMSO-$d_6$)**

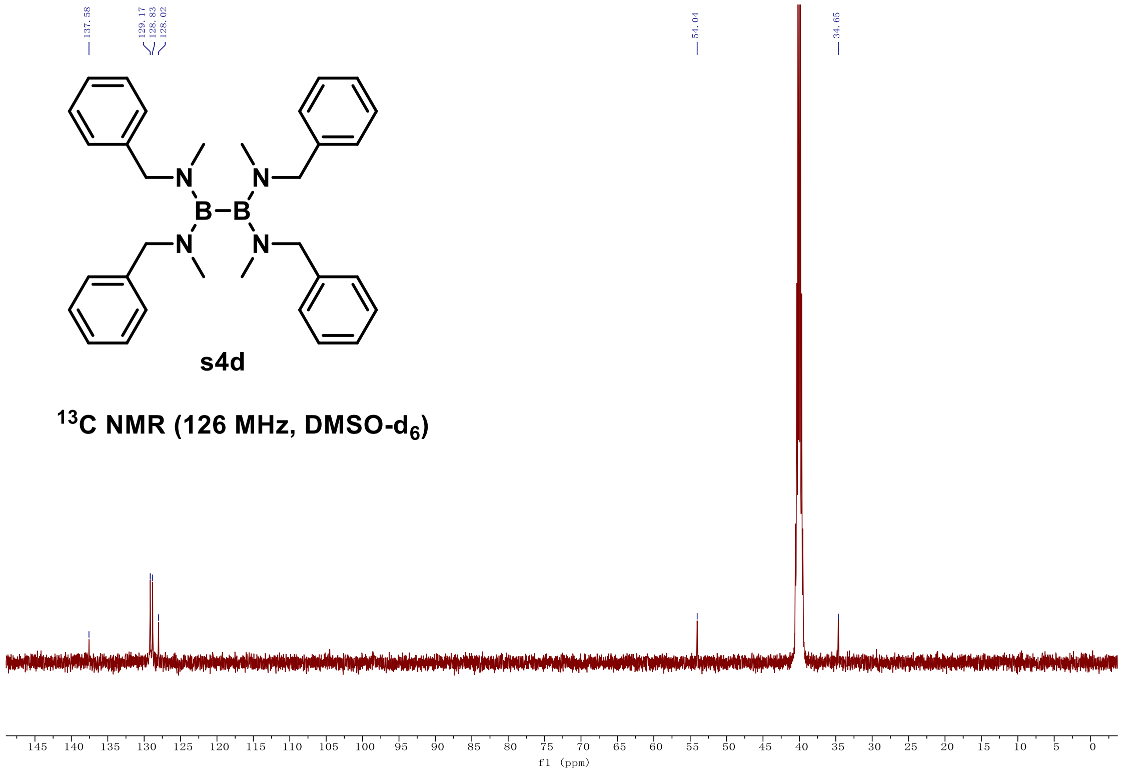


**s4d**

**$^{13}$C NMR (126 MHz, DMSO-$d_6$)**

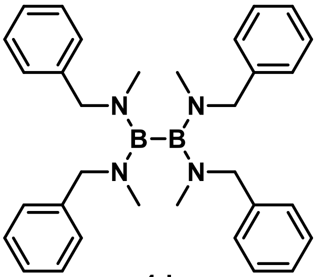

s4d

$^{11}$B NMR (161 MHz, DMSO-$d_6$)

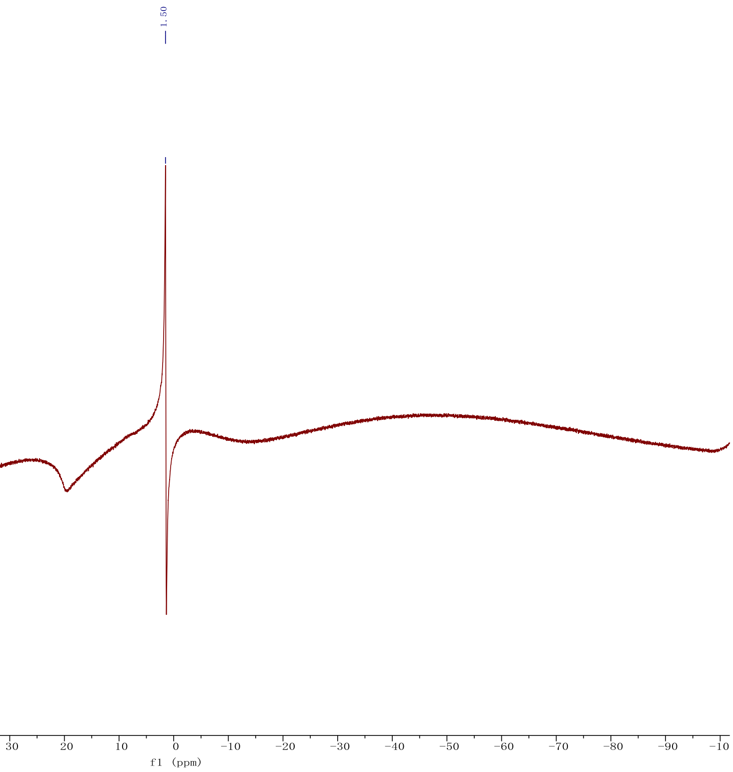


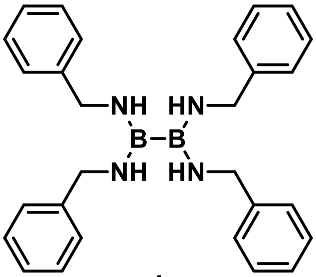

s4e

$^{1}$H NMR (500MHz, DMSO-$d_6$)

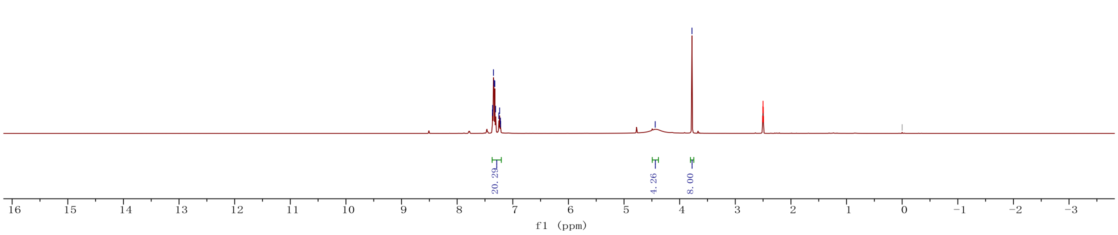

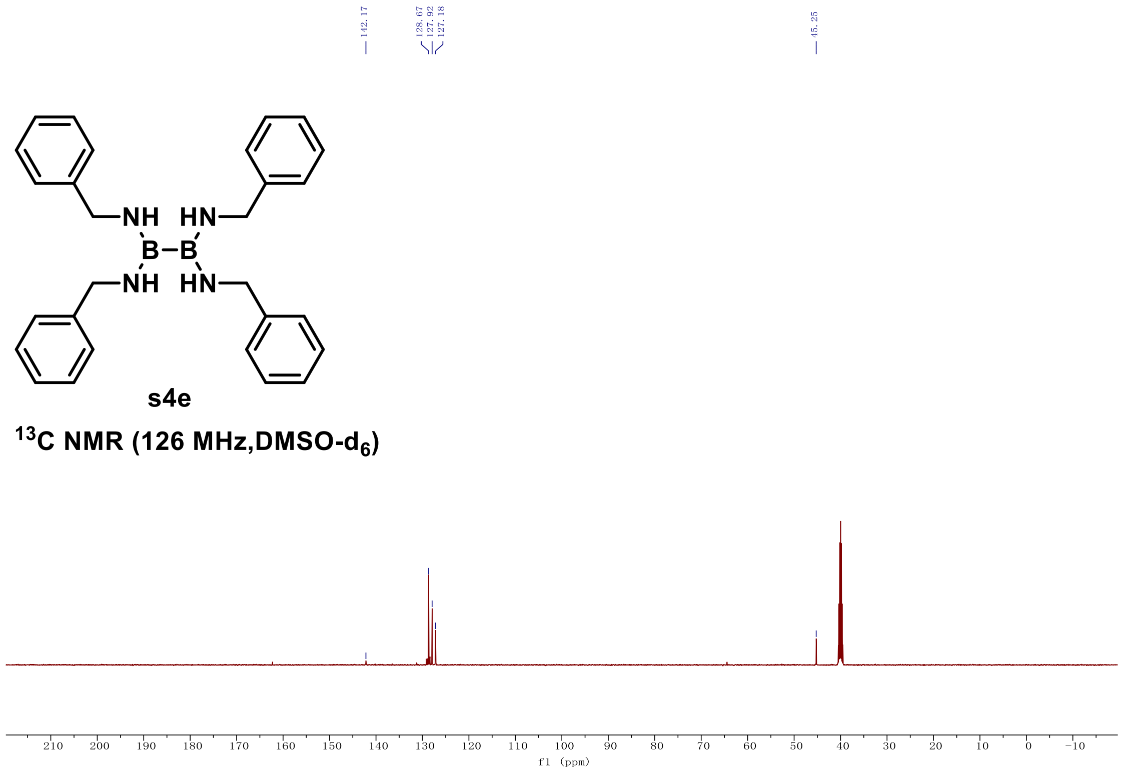

142.17
128.67
127.92
127.18
45.25
s4e
$^{13}$C NMR (126 MHz,DMSO-d$_6$)
f1 (ppm)


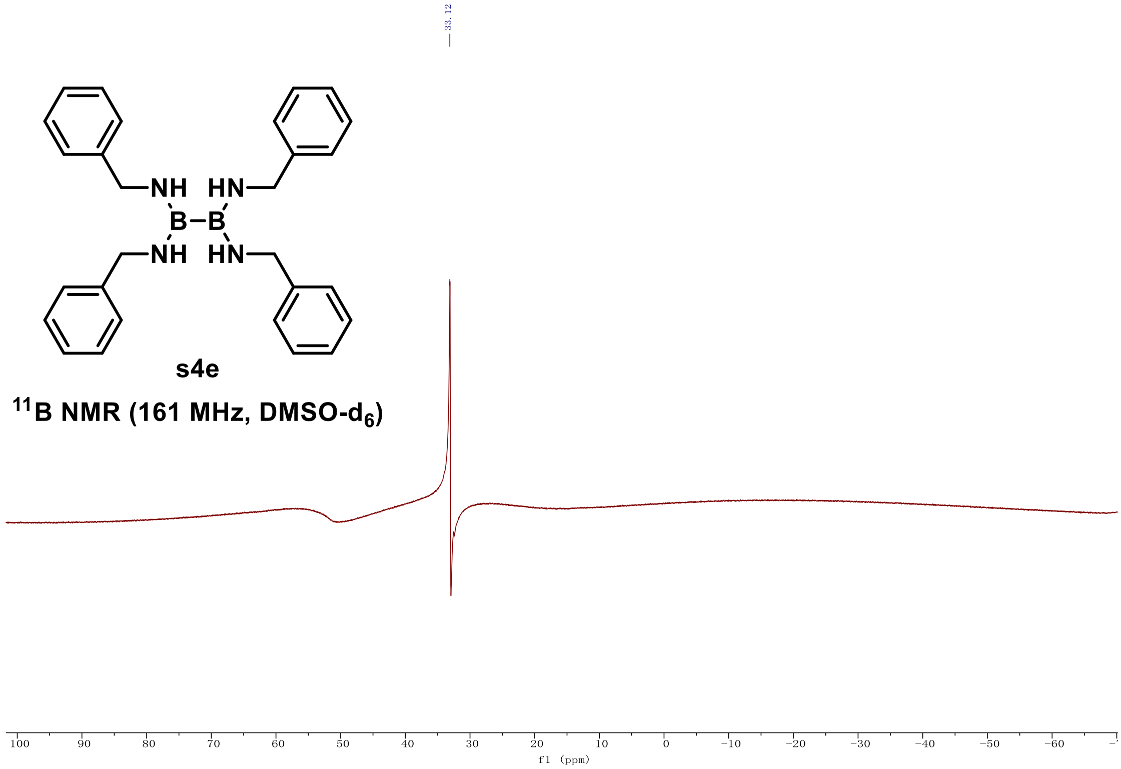

33.12
s4e
$^{11}$B NMR (161 MHz, DMSO-d$_6$)
f1 (ppm)

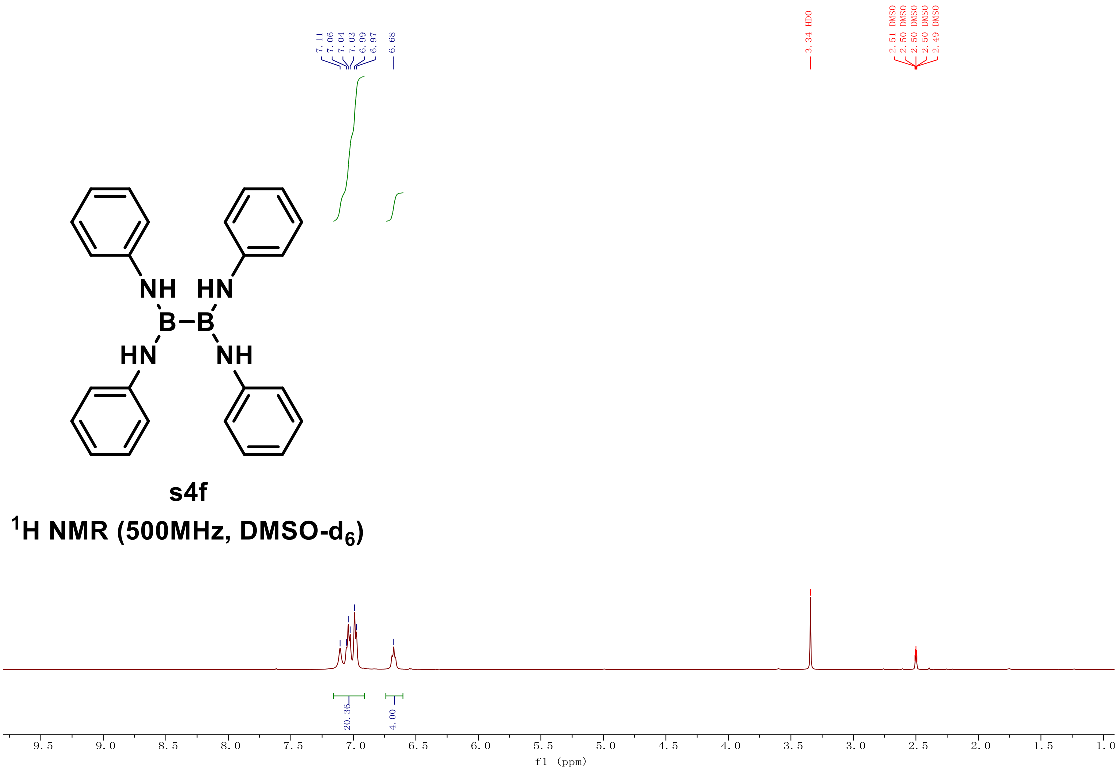


**s4f**

**$^{1}H$ NMR (500MHz, DMSO-$d_6$)**

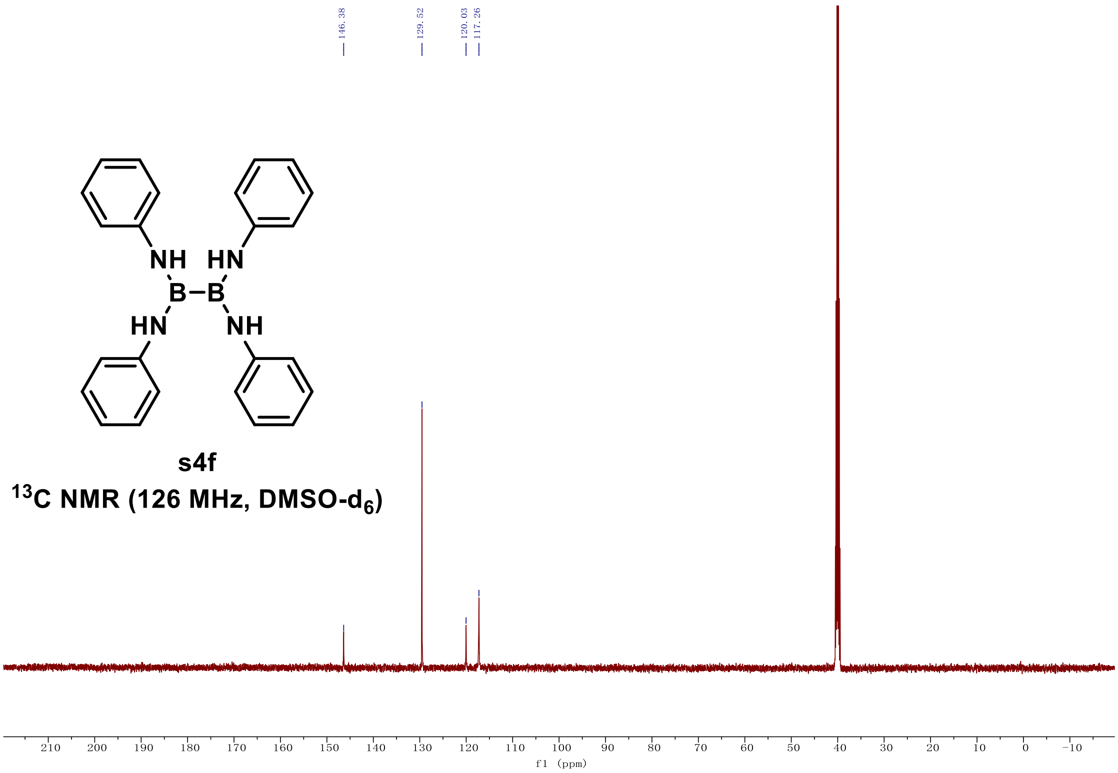


**s4f**

**$^{13}C$ NMR (126 MHz, DMSO-$d_6$)**

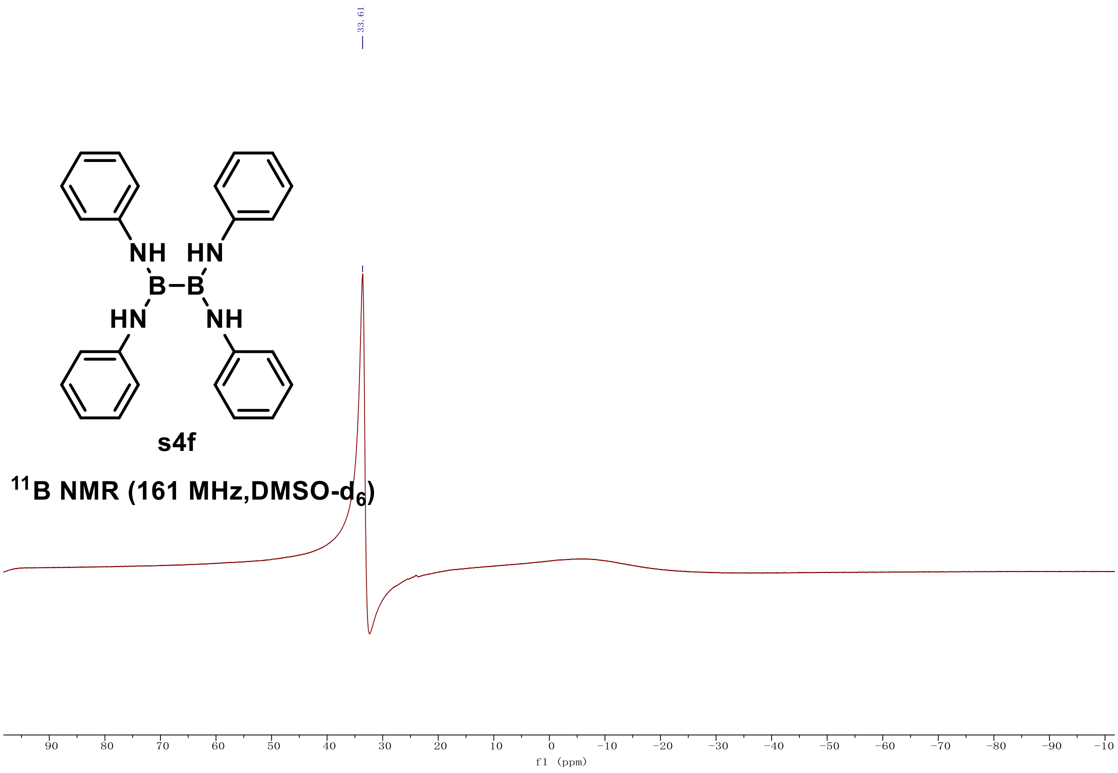
33.61
NH
HN
B–B
HN
NH
s4f
$^{11}$B NMR (161 MHz,DMSO-d$_6$)
f1 (ppm)

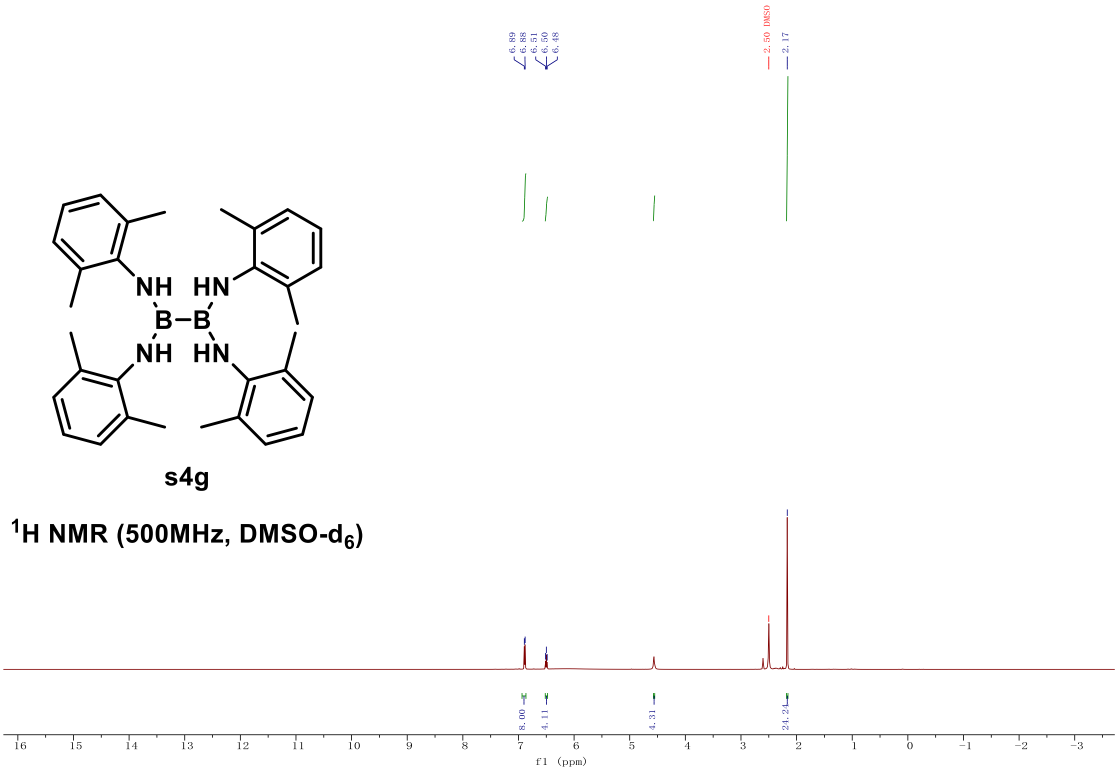
6.89
6.88
6.51
6.50
6.48
2.50 DMSO
2.17
NH
HN
B–B
NH
HN
s4g
$^1$H NMR (500MHz, DMSO-d$_6$)
8.00
4.11
4.31
24.24
f1 (ppm)

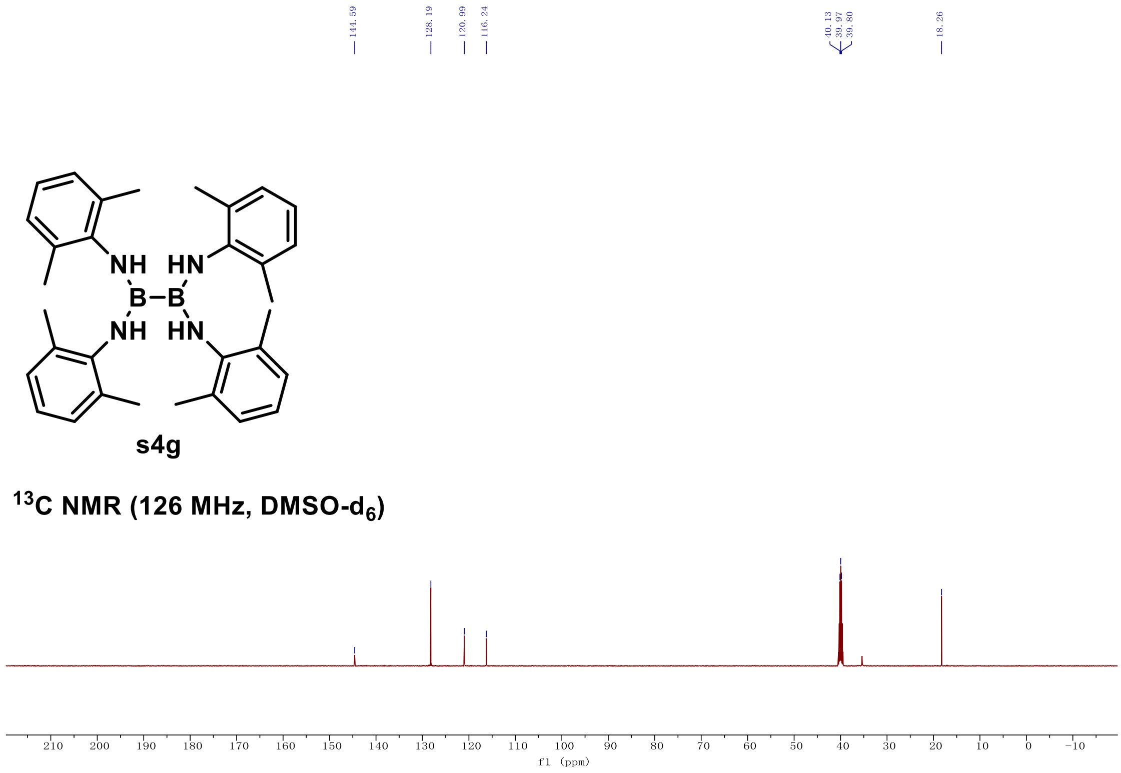
144.59
128.19
120.99
116.24
40.13
39.97
39.80
18.26
NH HN
B–B
NH HN
s4g
$^{13}$C NMR (126 MHz, DMSO-d$_6$)
210 200 190 180 170 160 150 140 130 120 110 100 90 80 70 60 50 40 30 20 10 0 −10
f1 (ppm)

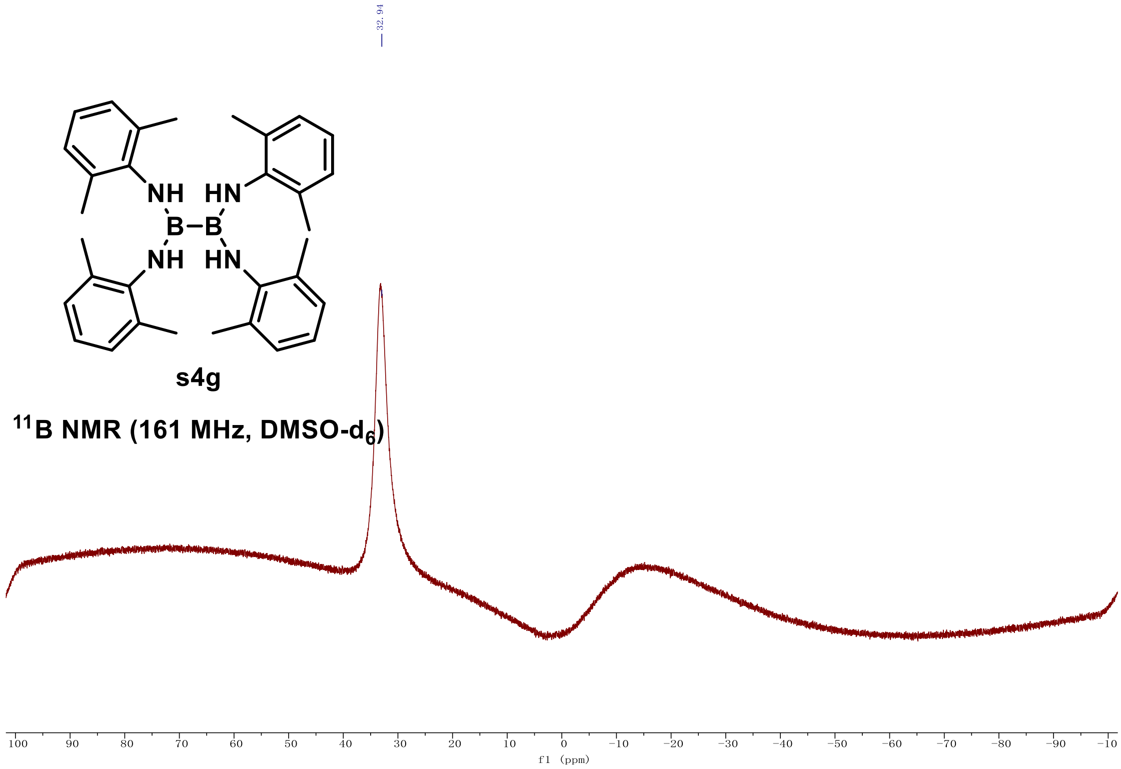
32.94
NH HN
B–B
NH HN
s4g
$^{11}$B NMR (161 MHz, DMSO-d$_6$)
100 90 80 70 60 50 40 30 20 10 0 −10 −20 −30 −40 −50 −60 −70 −80 −90 −10
f1 (ppm)

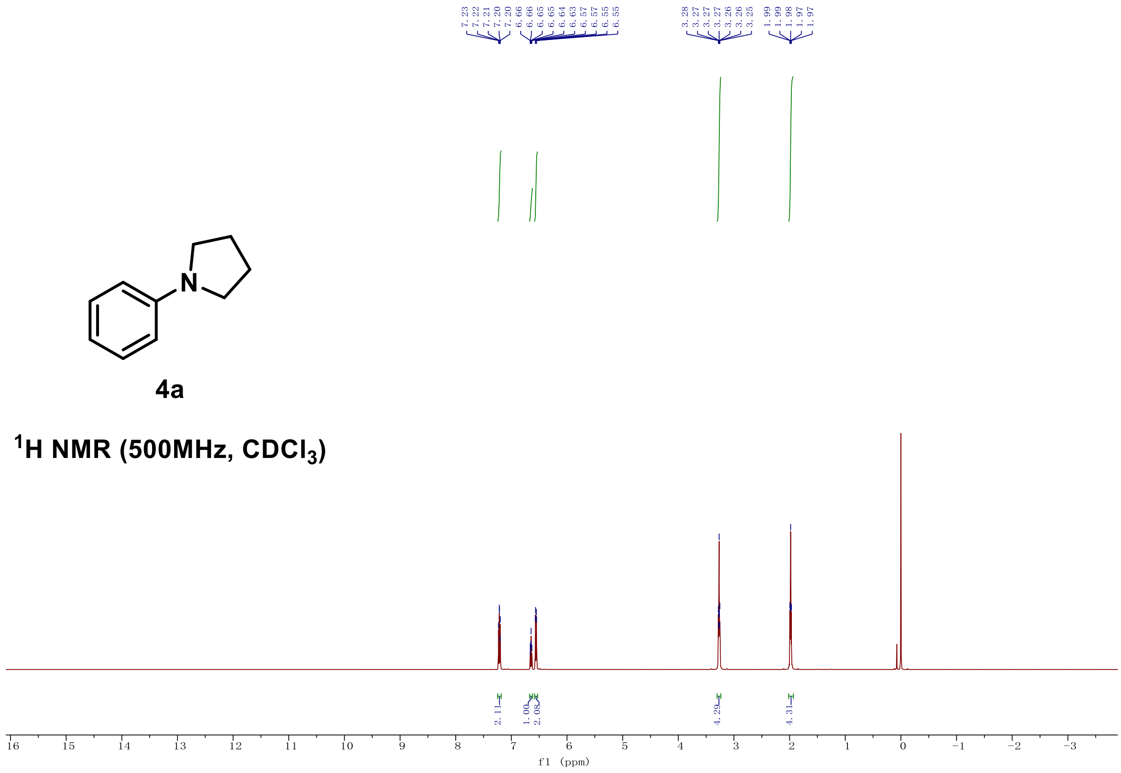
4a
$^{1}$H NMR (500MHz, CDCl$_3$)

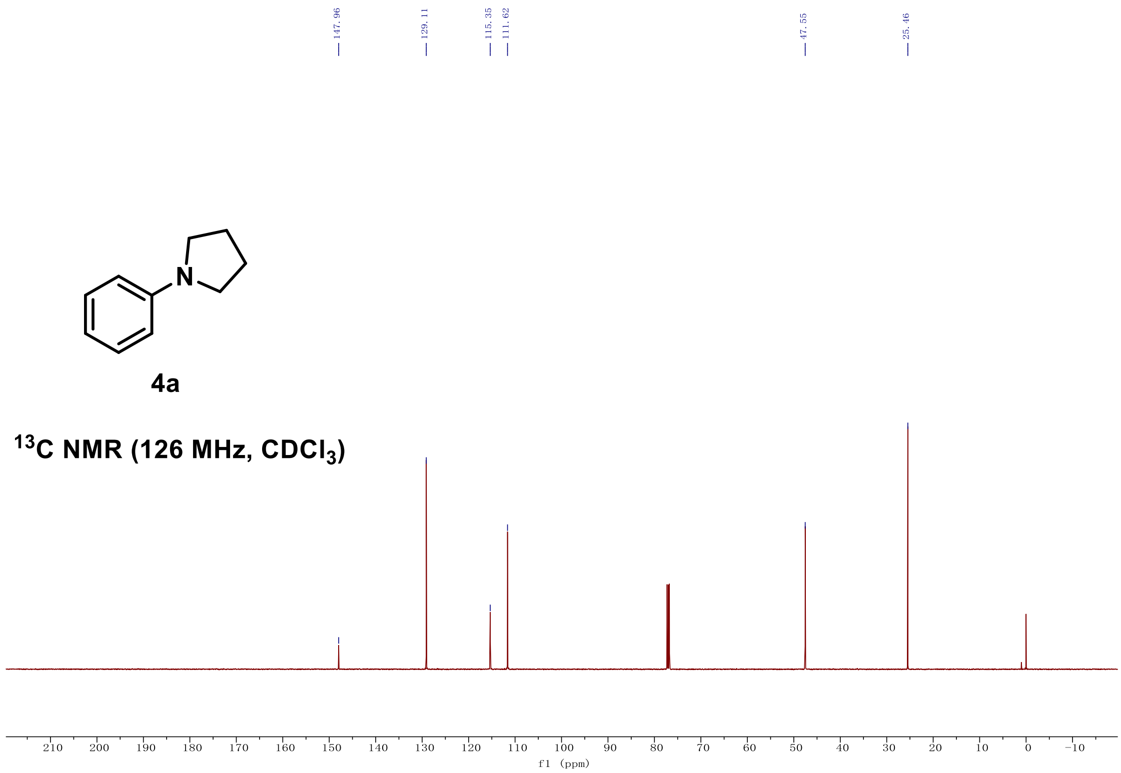
4a
$^{13}$C NMR (126 MHz, CDCl$_3$)

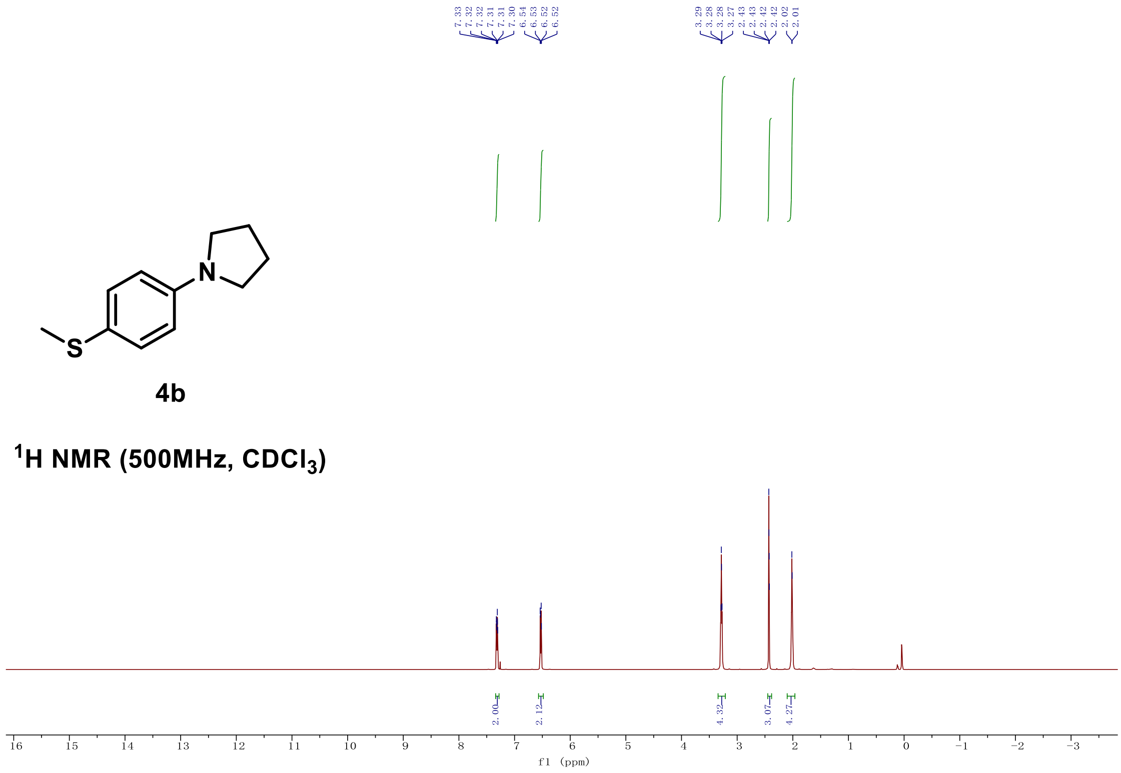


**4b**

**$^{1}$H NMR (500MHz, $CDCl_3$)**

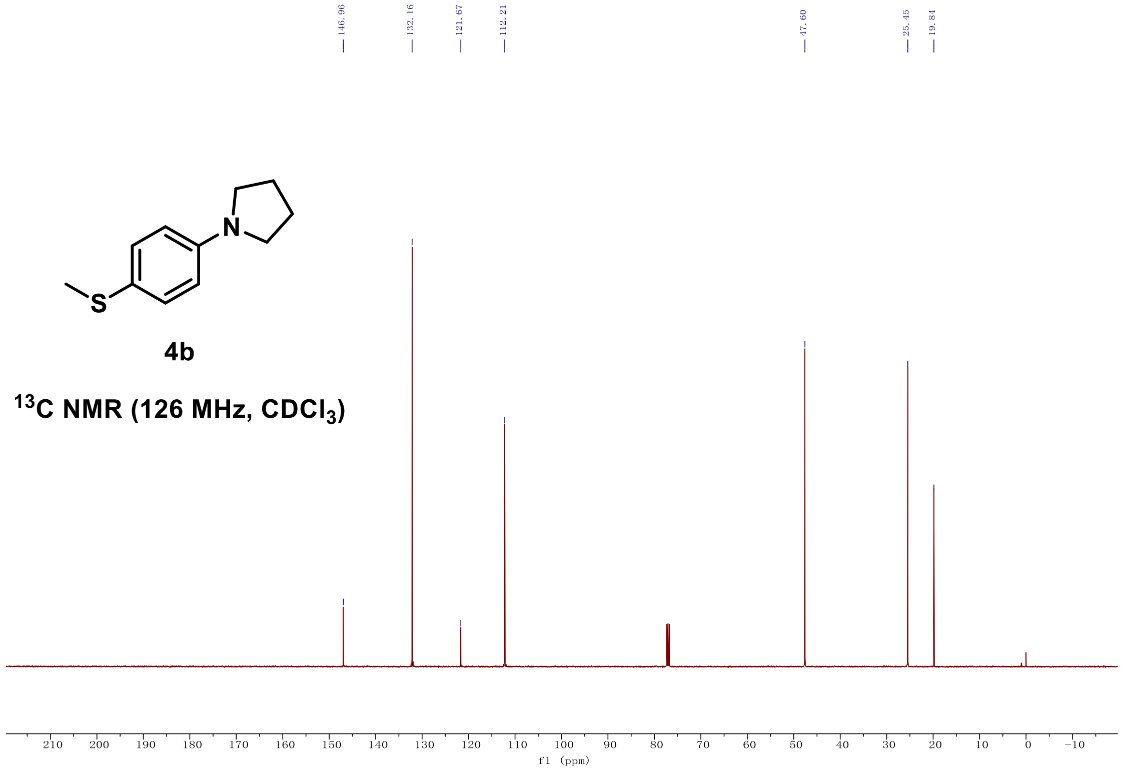


**4b**

**$^{13}$C NMR (126 MHz, $CDCl_3$)**

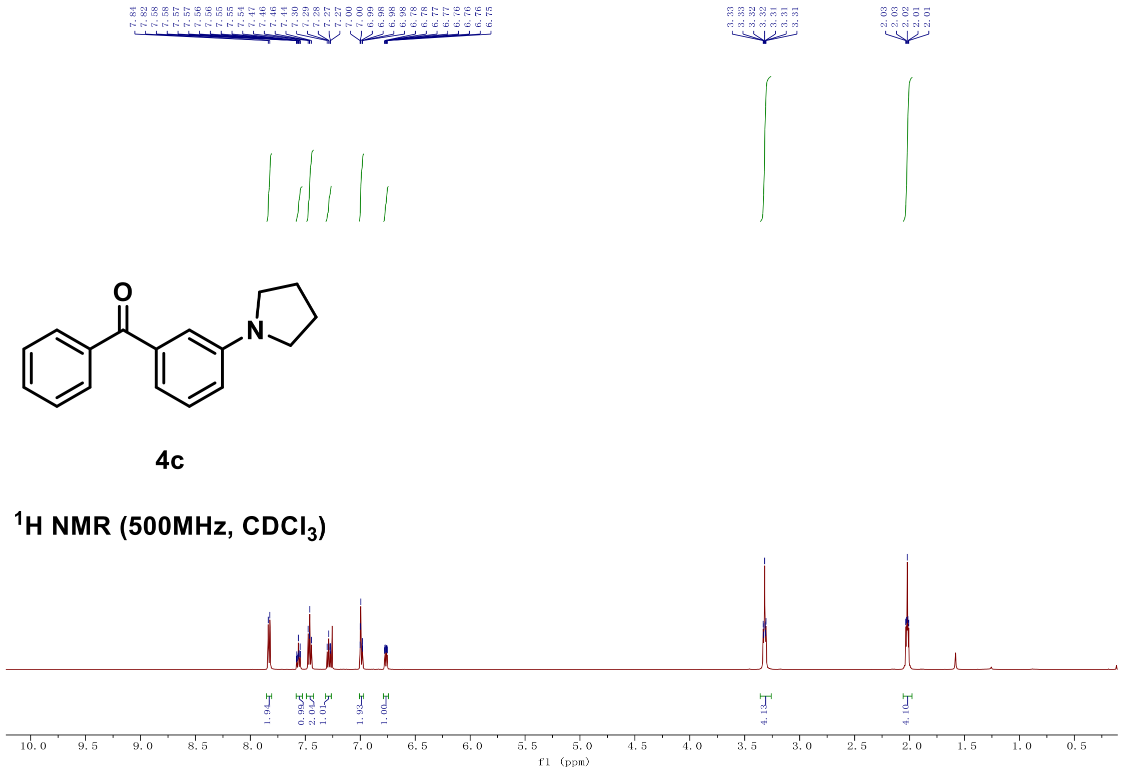


4c

$^{1}$H NMR (500MHz, $CDCl_3$)

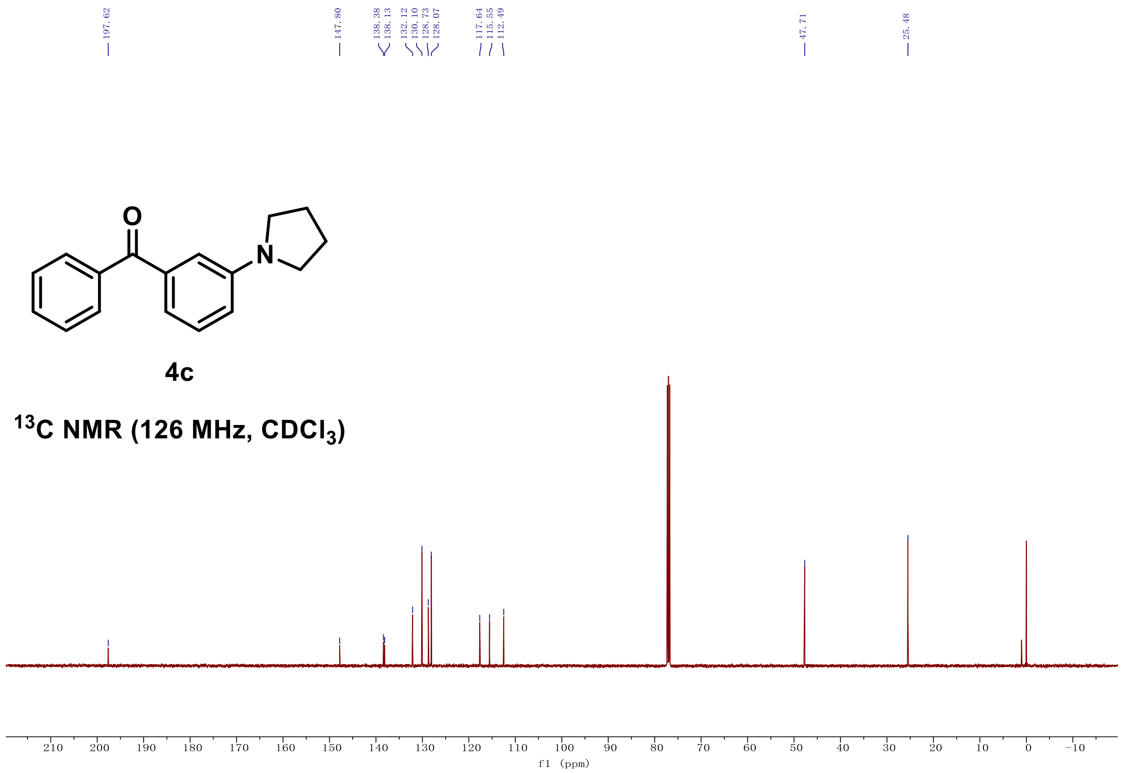


4c

$^{13}$C NMR (126 MHz, $CDCl_3$)

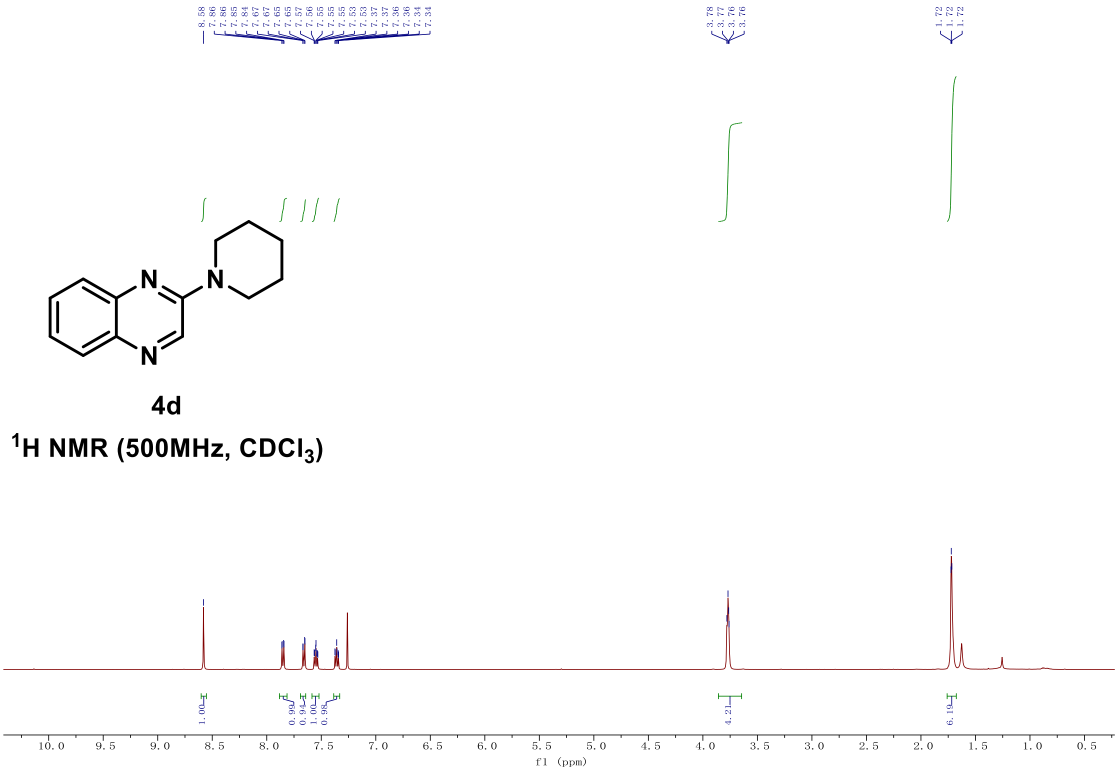

4d

$^{1}H$ NMR (500MHz, $CDCl_3$)

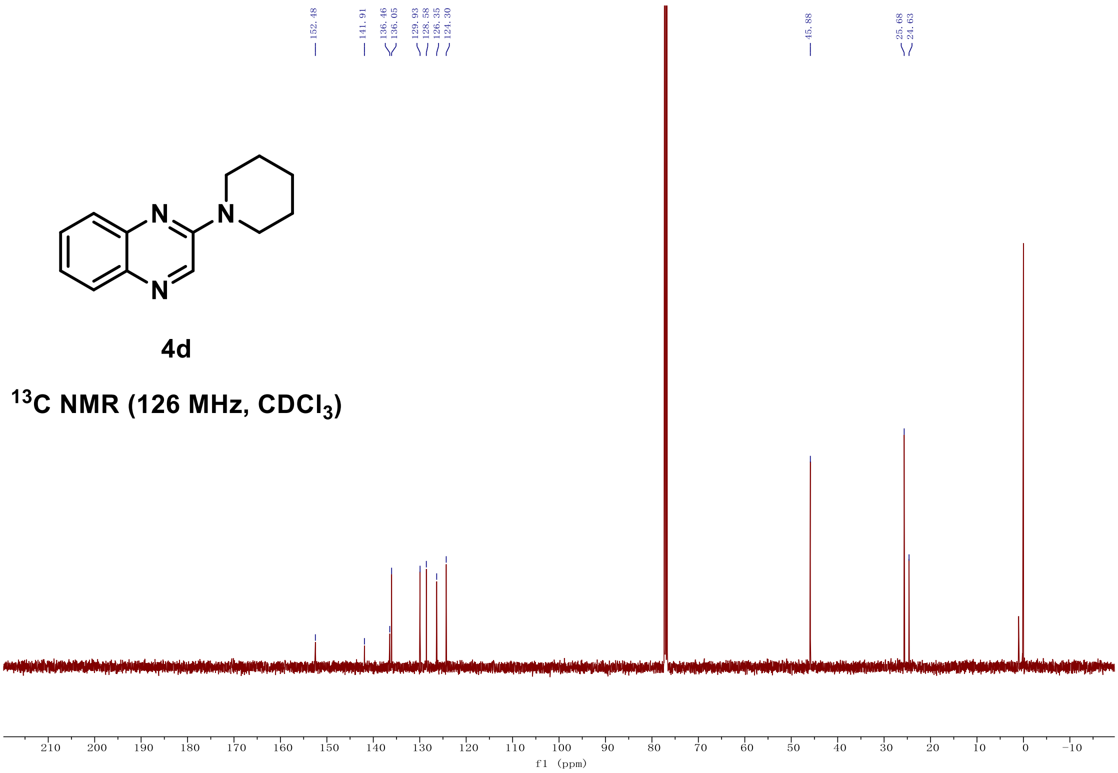

4d

$^{13}C$ NMR (126 MHz, $CDCl_3$)

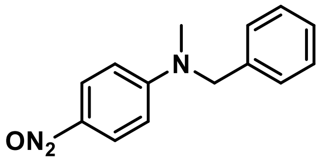

**4e**

**$^{1}H$ NMR (500MHz, $CDCl_3$)**

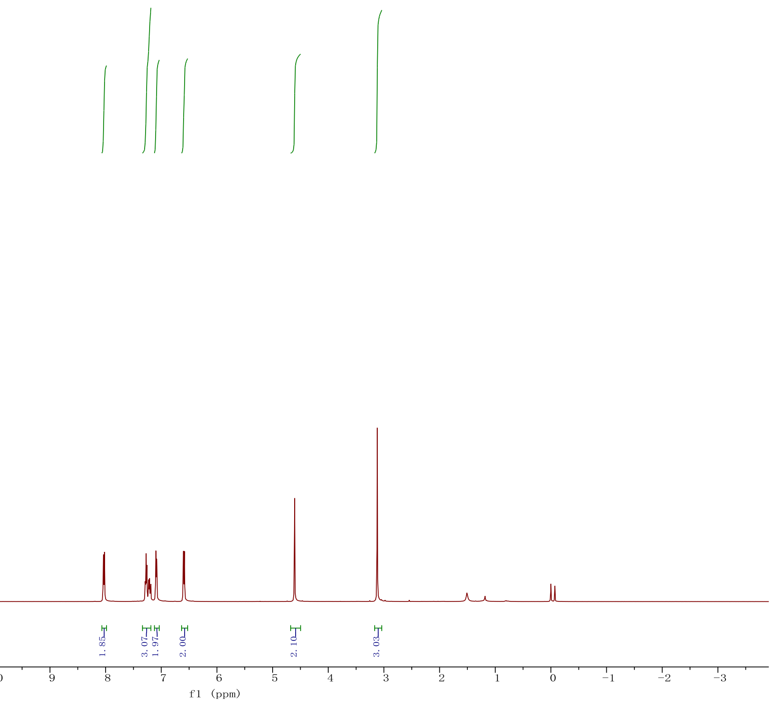


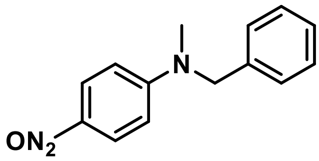

**4e**

**$^{13}C$ NMR (126 MHz, $CDCl_3$)**

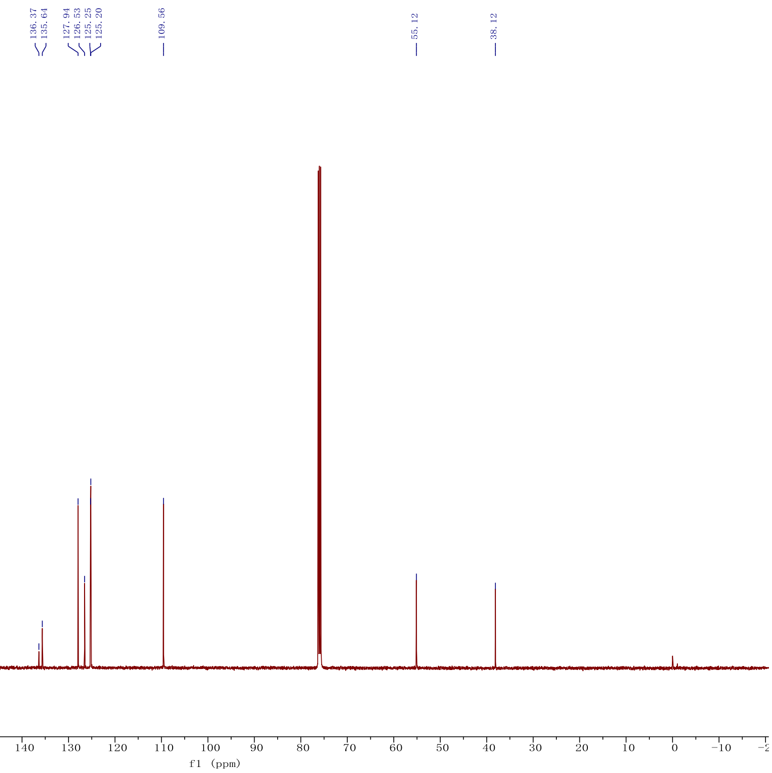

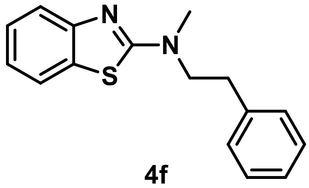

**4f**

**$^{1}$H NMR (500MHz, $CDCl_3$)**

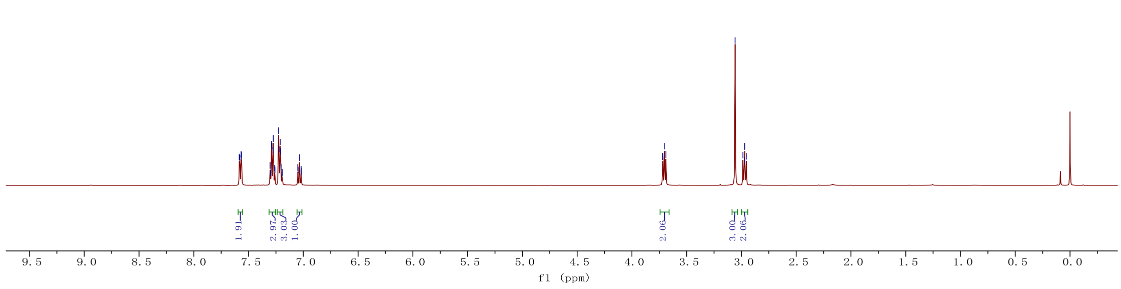


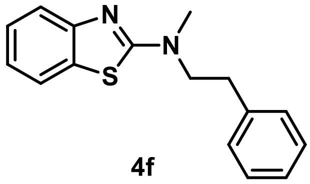

**4f**

**$^{13}$C NMR (126 MHz, $CDCl_3$)**

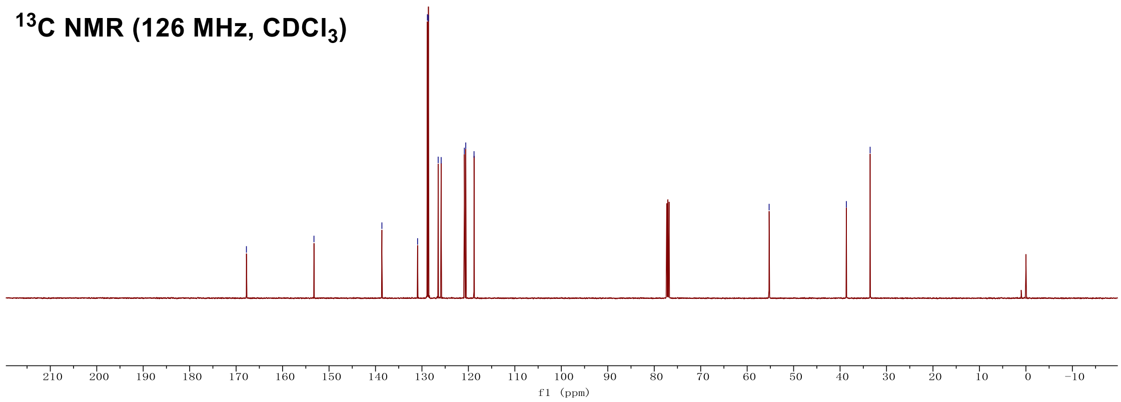

**4g**

**$^{1}$H NMR (500MHz, $CDCl_3$)**

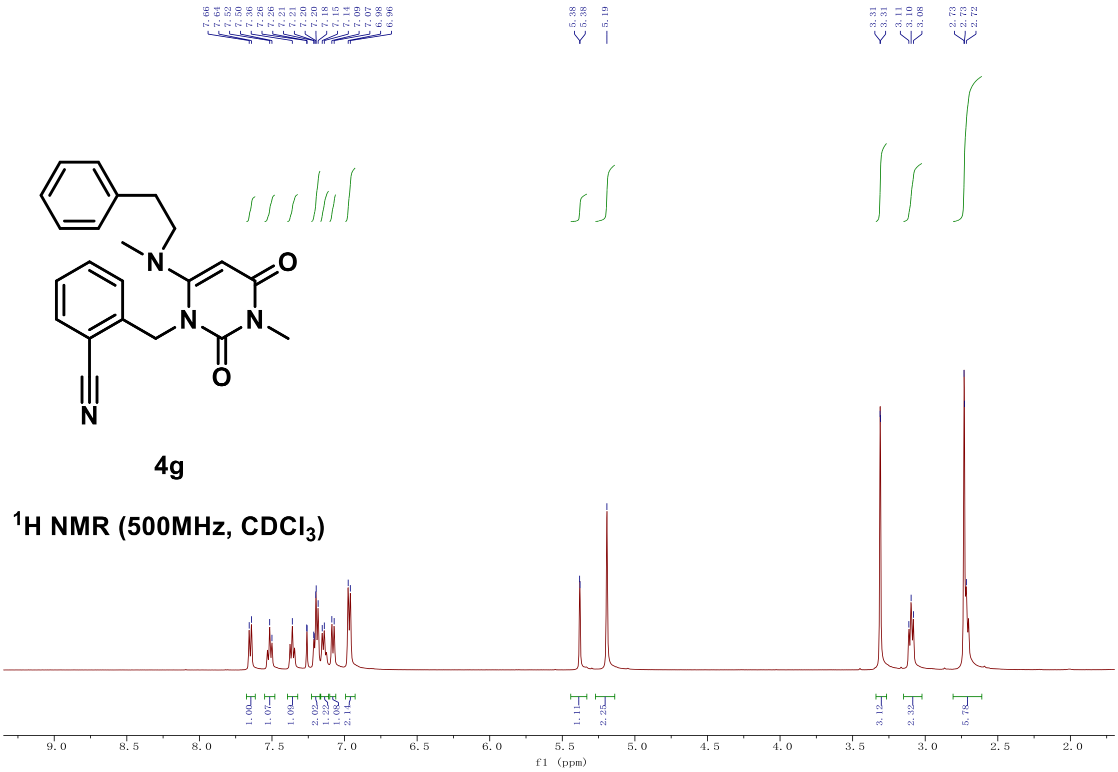


**4g**

**$^{13}$C NMR (126 MHz, $CDCl_3$)**

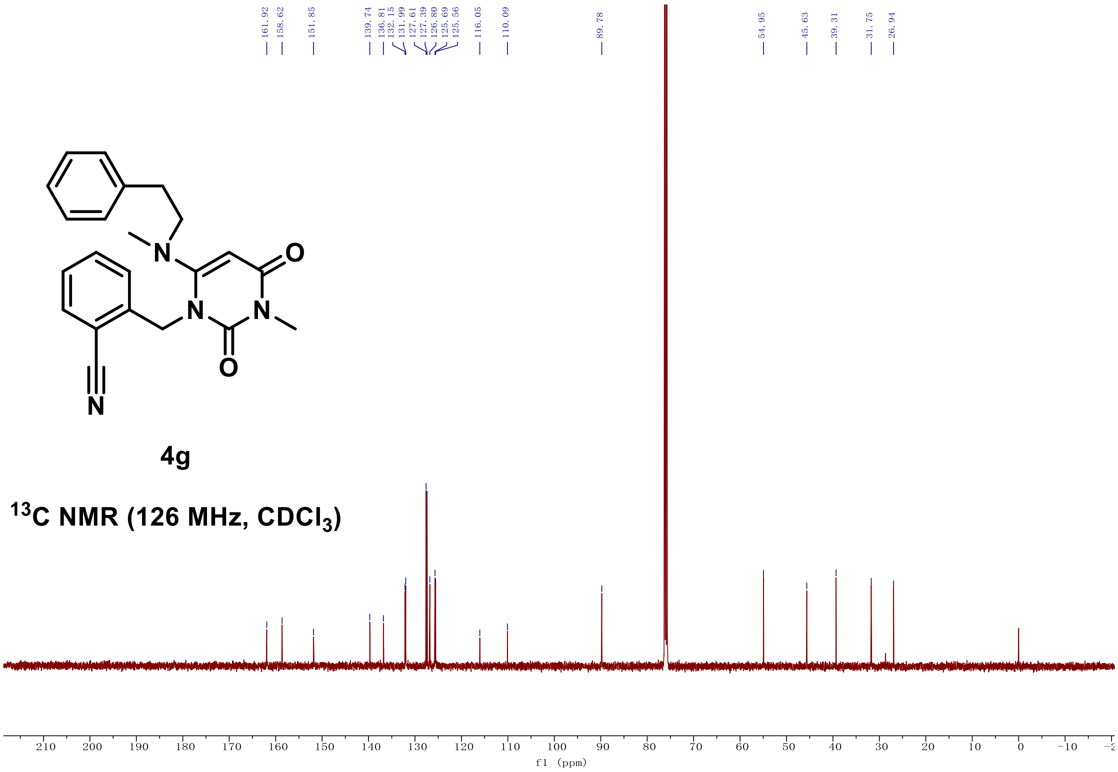

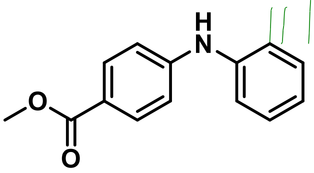

**4h**

## $^{1}$H NMR (500MHz, $CDCl_3$)

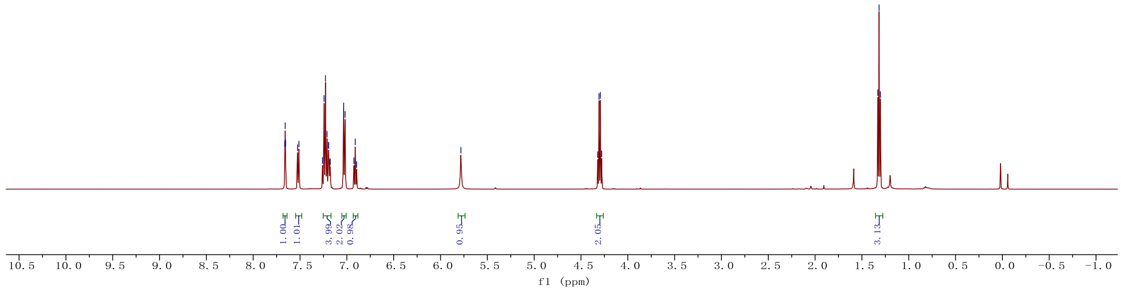


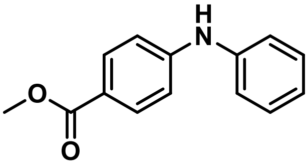

**4h**

## $^{13}$C NMR (126 MHz, $CDCl_3$)

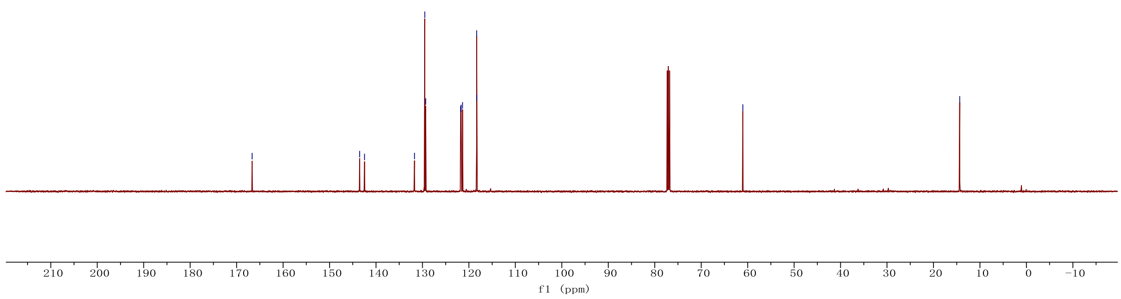

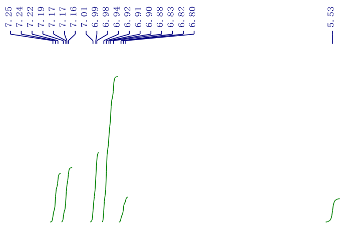


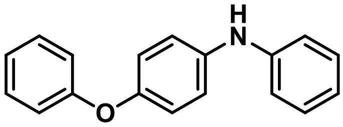


**4i**

## $^1$H NMR (500MHz, $CDCl_3$)

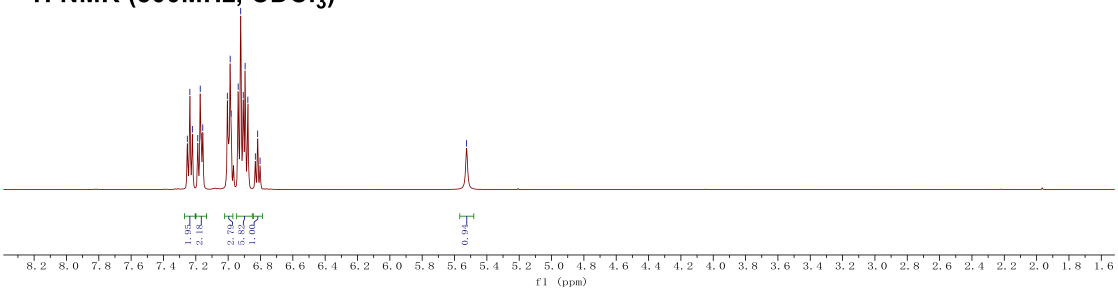


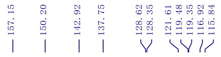


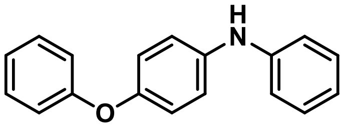


**4i**

## $^{13}$C NMR (126 MHz, $CDCl_3$)

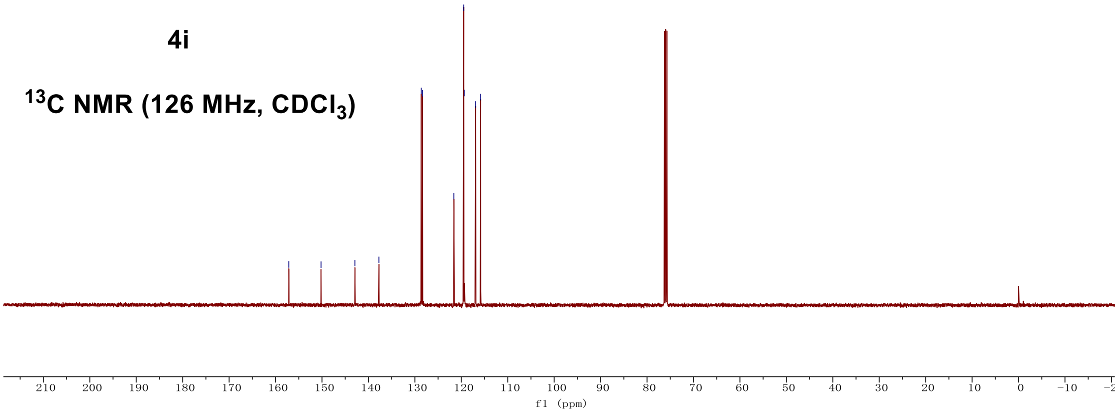

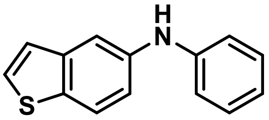

**4j**

**$^{1}$H NMR (500MHz, $CDCl_3$)**

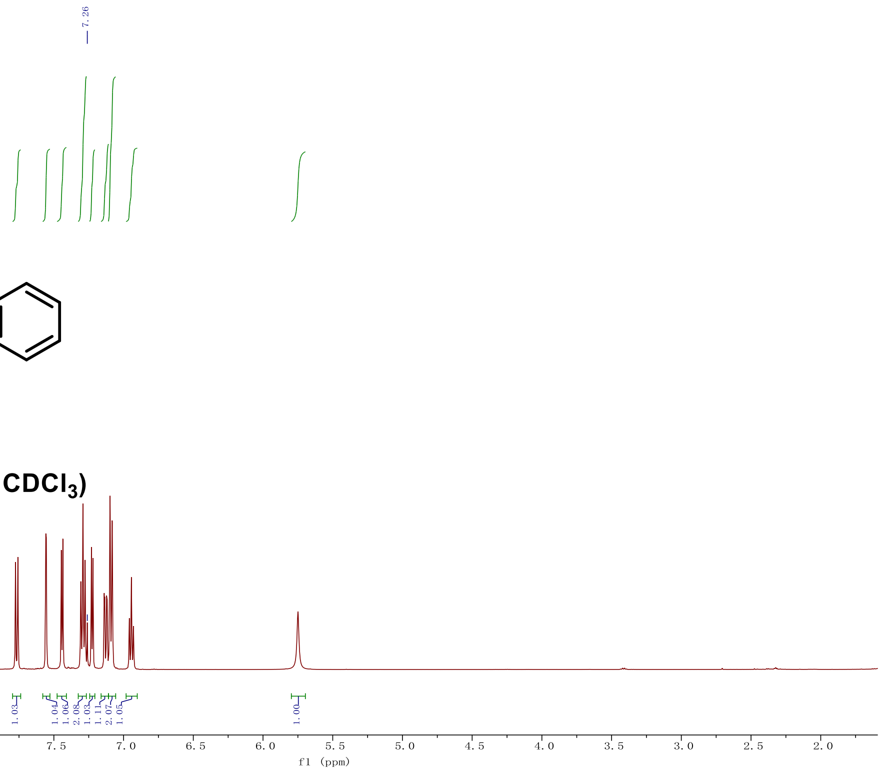


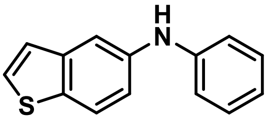

**4j**

**$^{13}$C NMR (126 MHz, $CDCl_3$)**

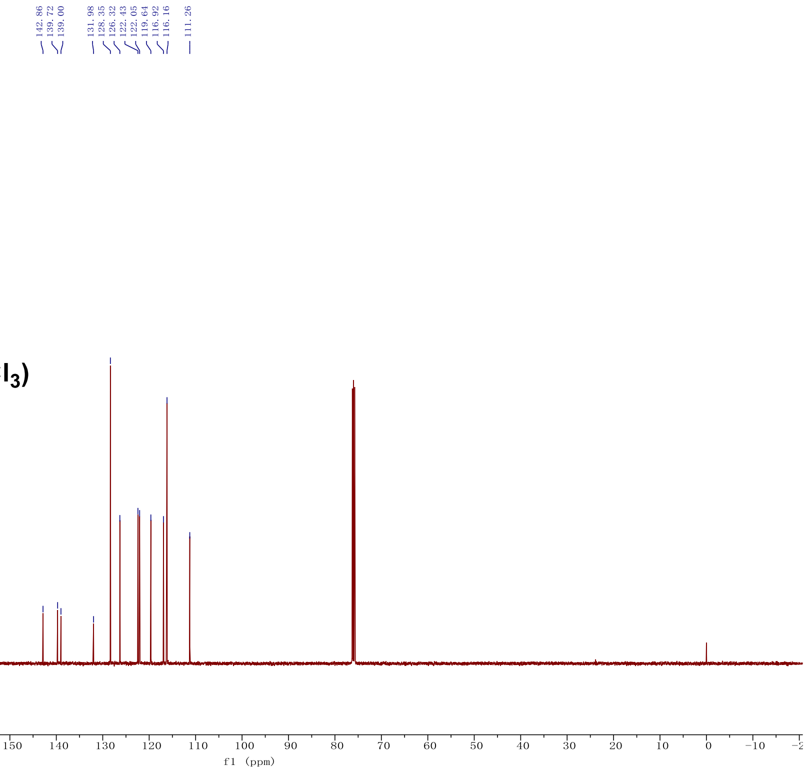

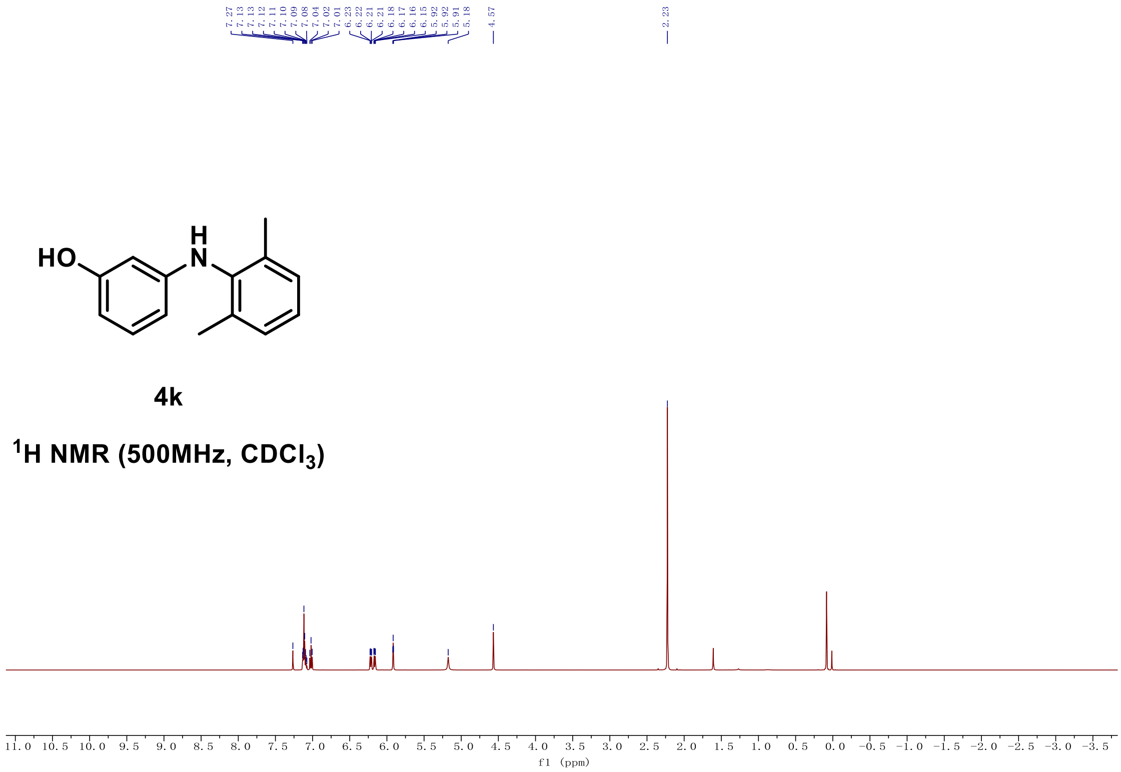


**4k**

**$^1$H NMR (500MHz, CDCl$_3$)**

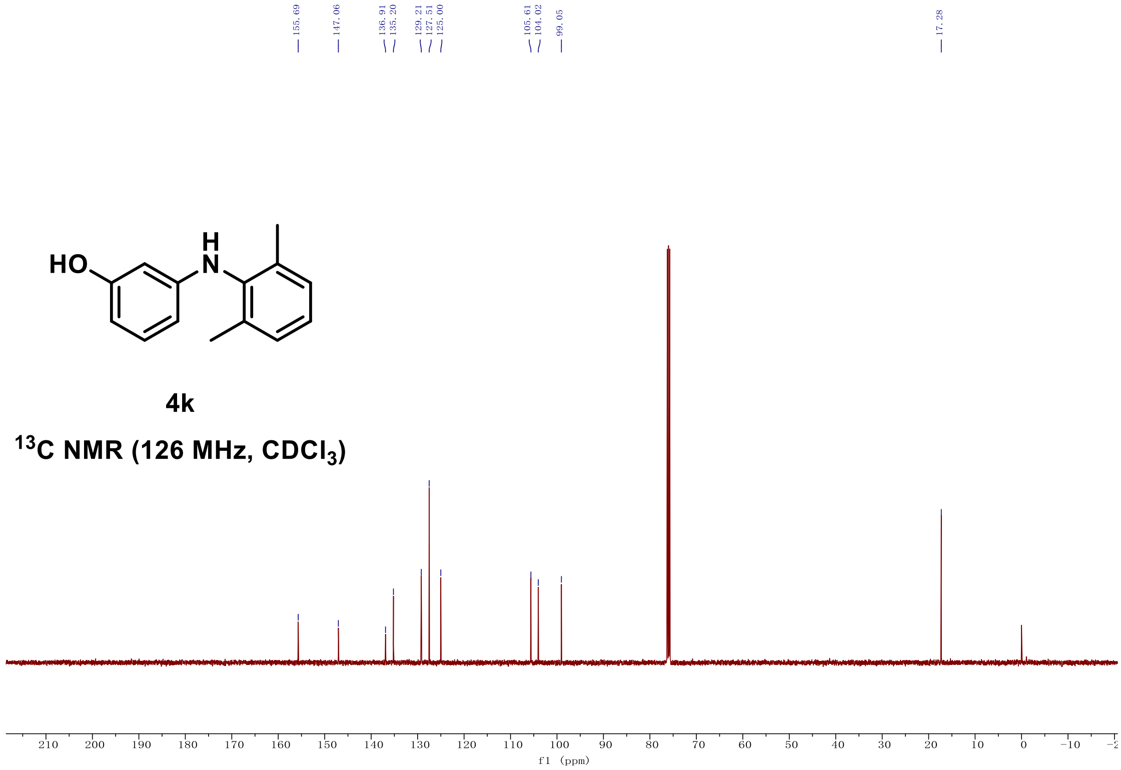


**4k**

**$^{13}$C NMR (126 MHz, CDCl$_3$)**

**5a**

**$^{1}$H NMR (500 MHz, $CDCl_3$)**

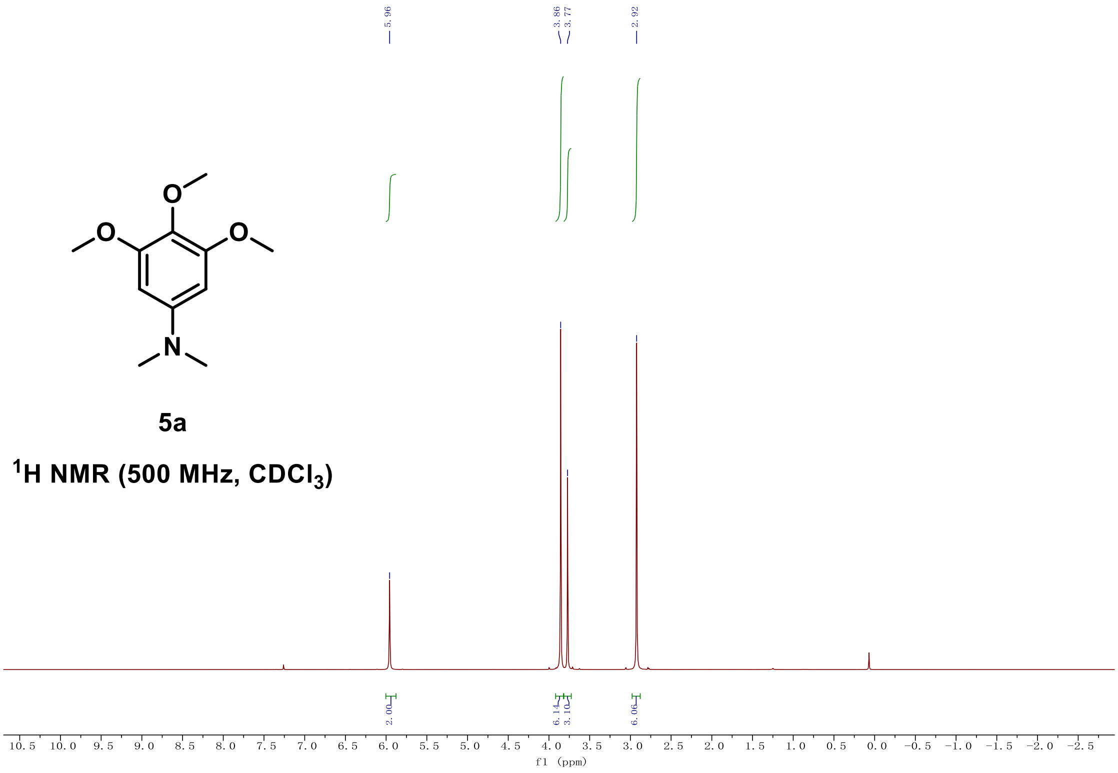


**5a**

**$^{13}$C NMR (126 MHz, $CDCl_3$)**

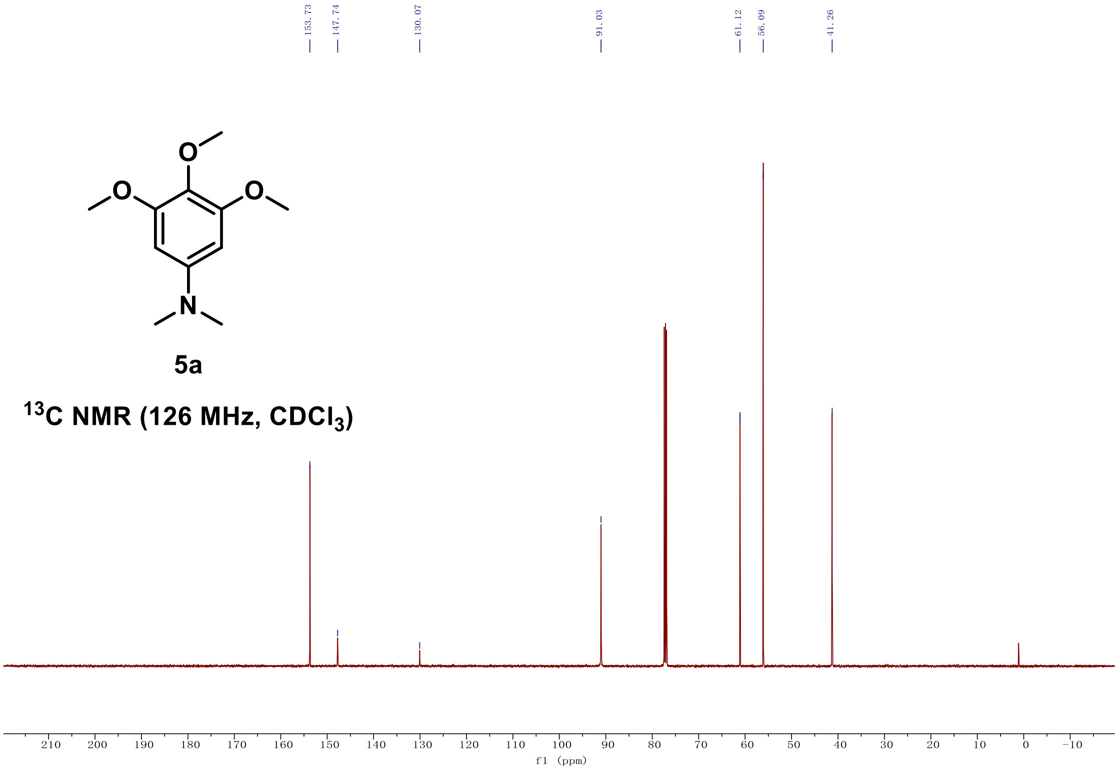

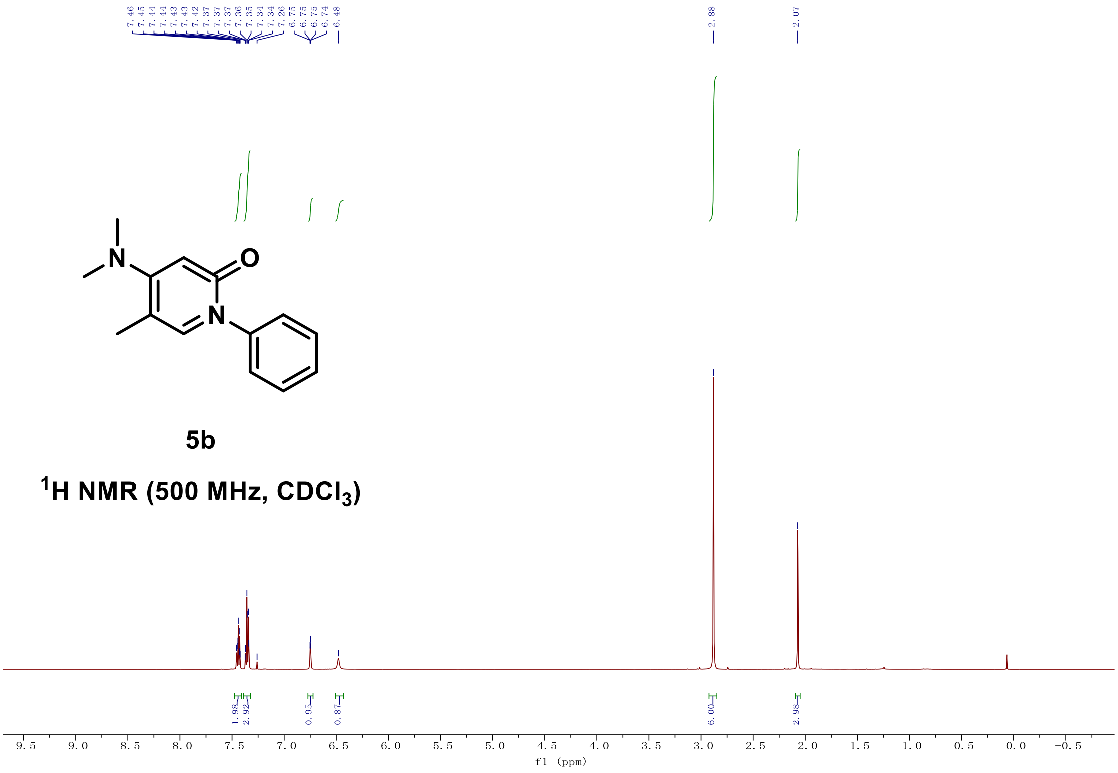


**5b**

$^{1}H$ NMR (500 MHz, $CDCl_3$)

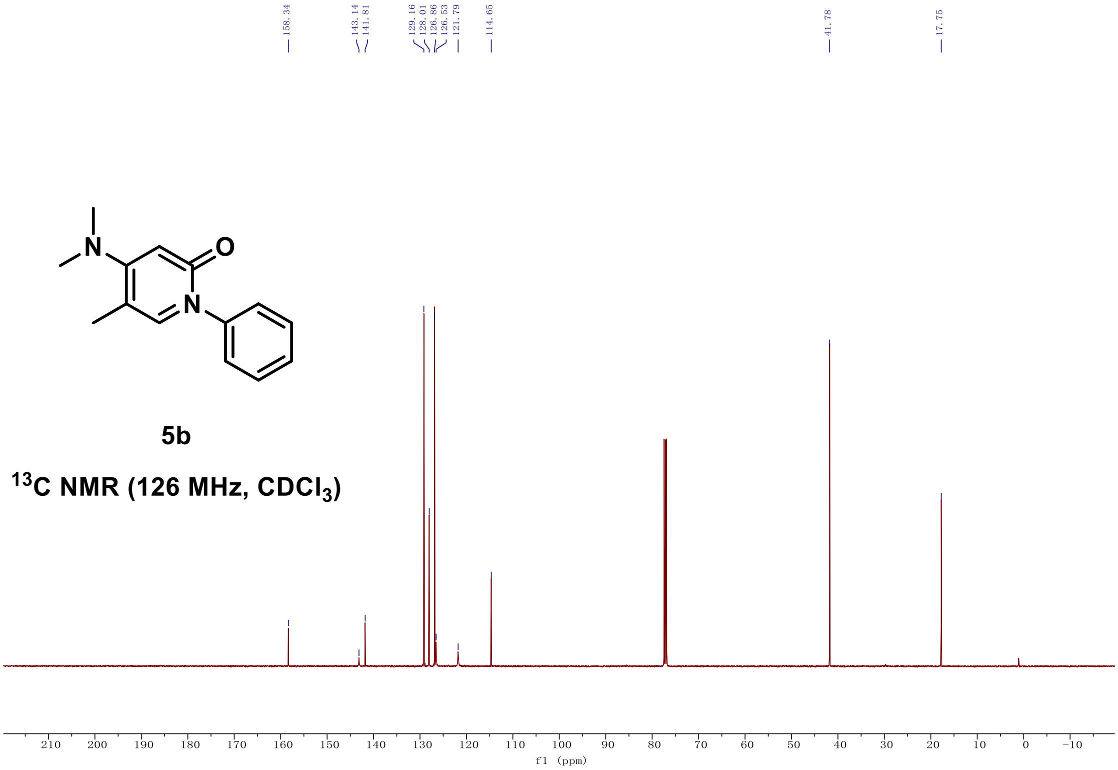


**5b**

$^{13}C$ NMR (126 MHz, $CDCl_3$)

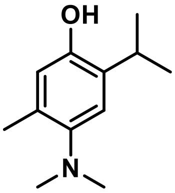

5c

$^1$H NMR (500 MHz, $CDCl_3$)

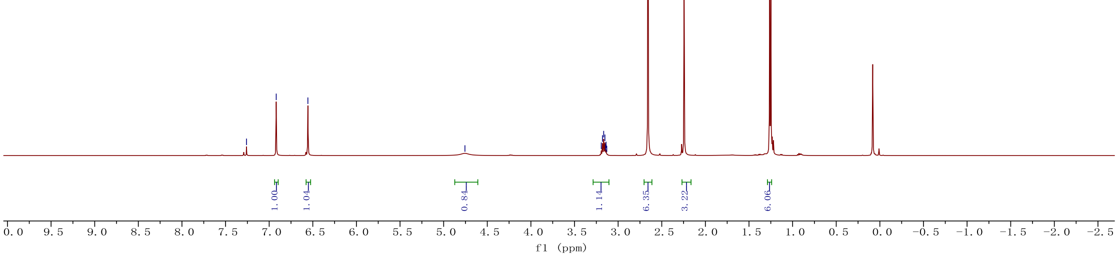


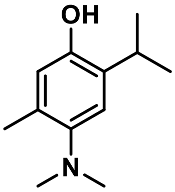

5c

$^{13}$C NMR (126 MHz, $CDCl_3$)

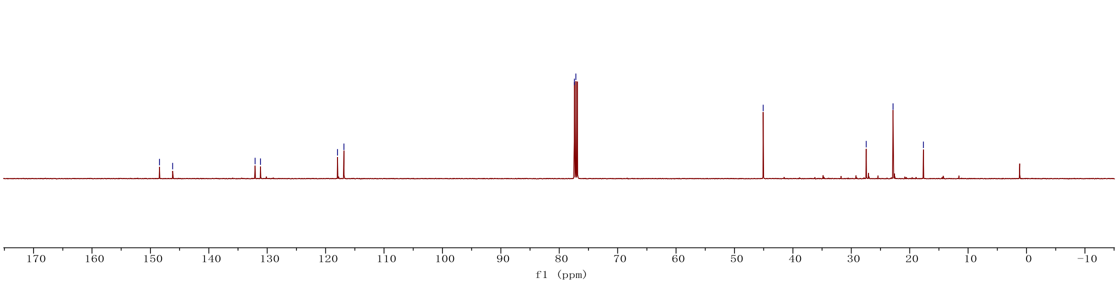

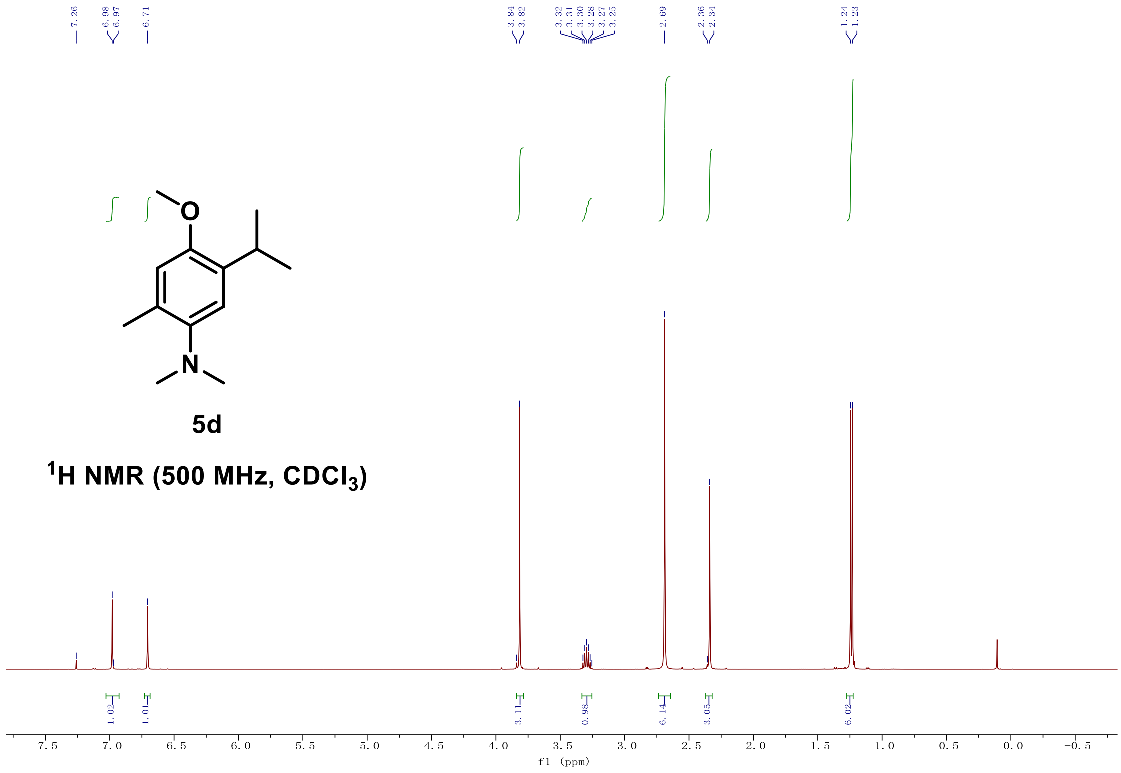
5d
1H NMR (500 MHz, CDCl3)
f1 (ppm)

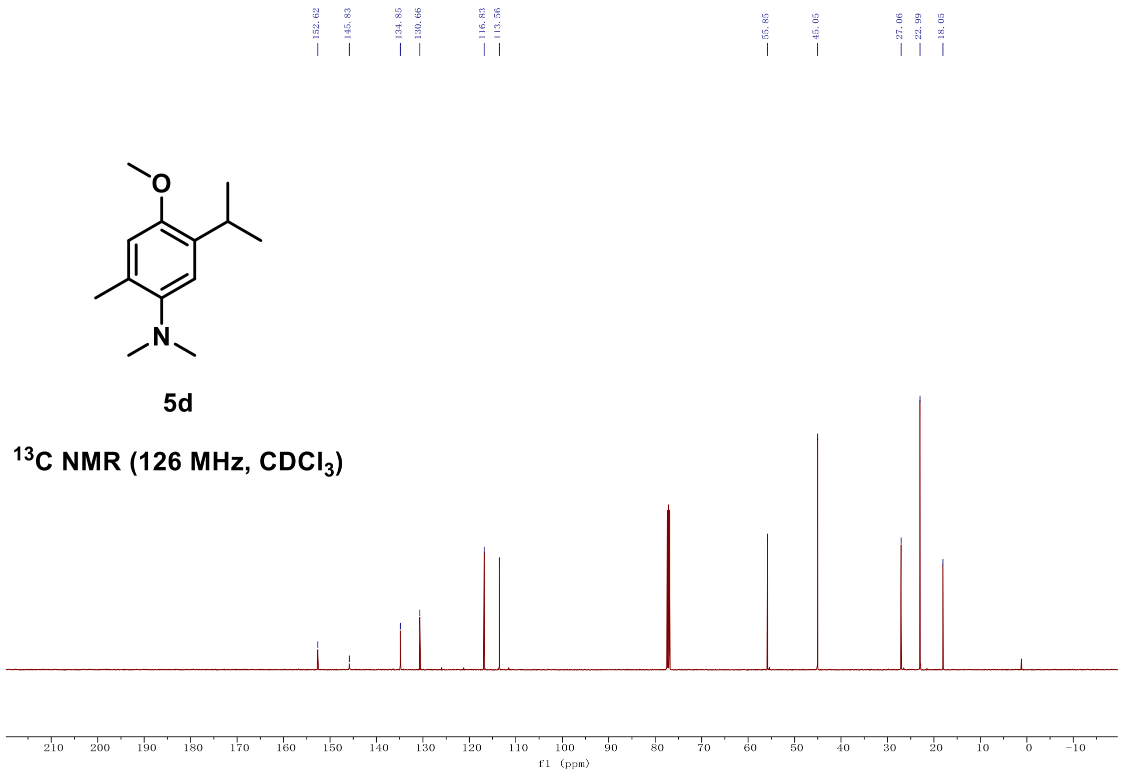
5d
13C NMR (126 MHz, CDCl3)
f1 (ppm)

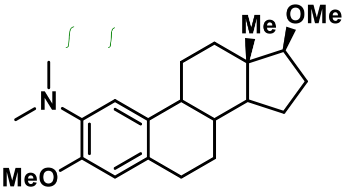


**5e**

**$^{1}$H NMR (500 MHz, CDCl$_3$)**

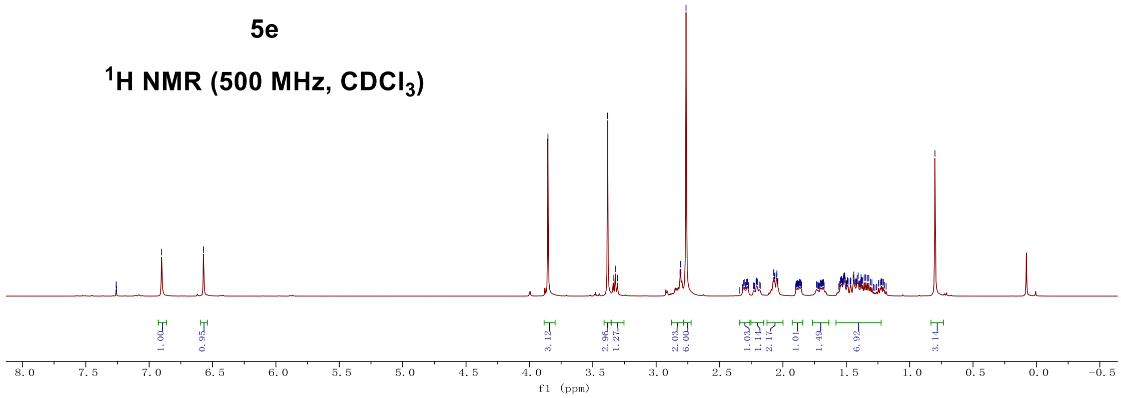


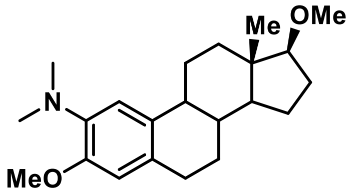


**5e**

**$^{13}$C NMR (126 MHz, CDCl$_3$)**

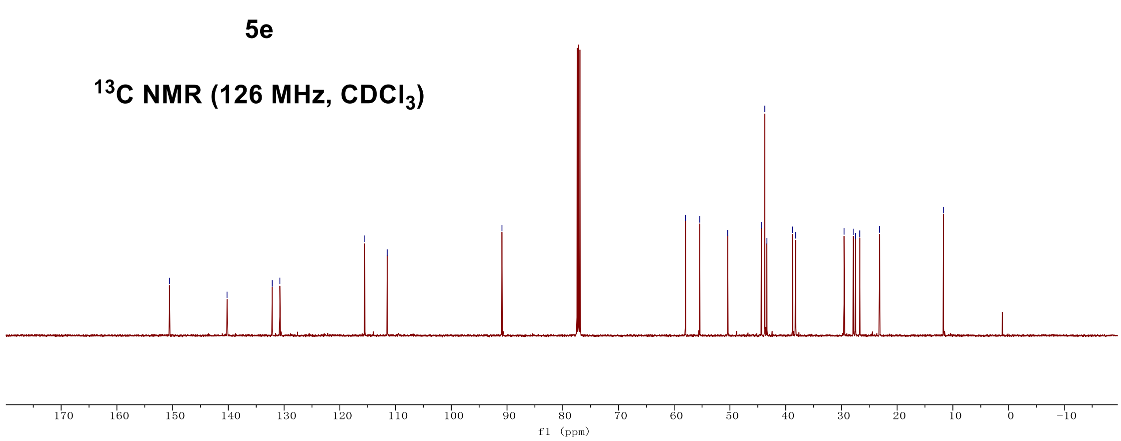

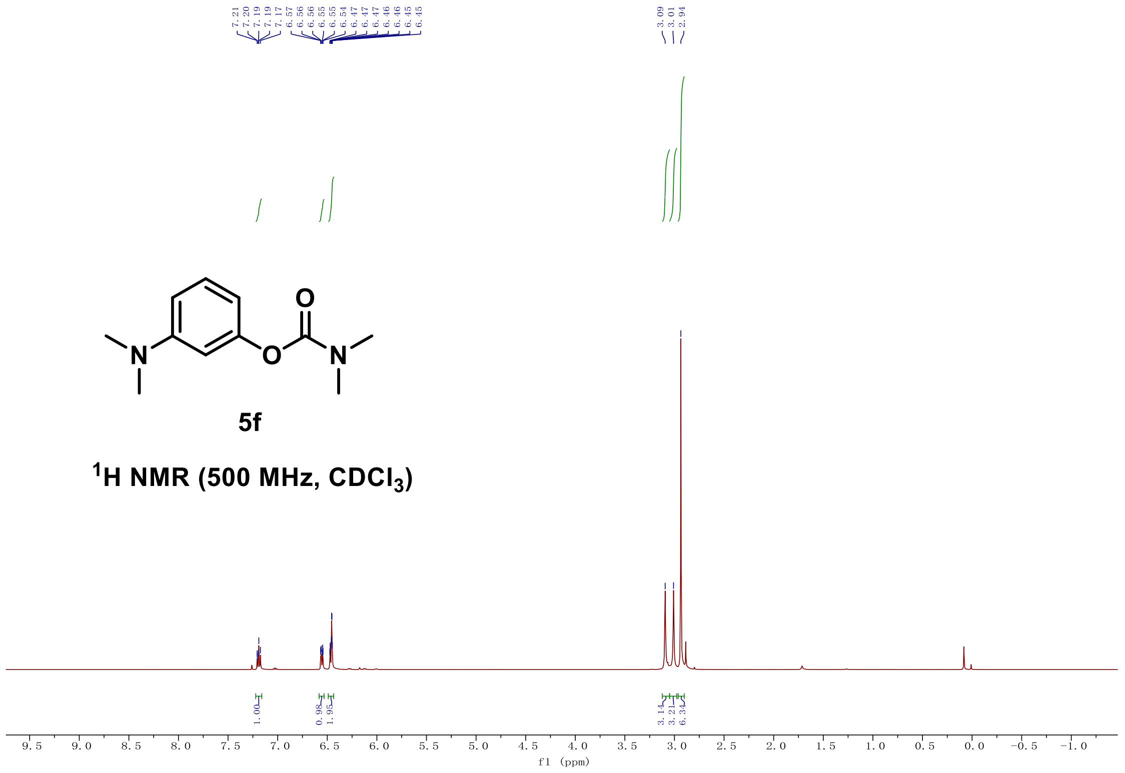
5f
$^1$H NMR (500 MHz, CDCl$_3$)
f1 (ppm)

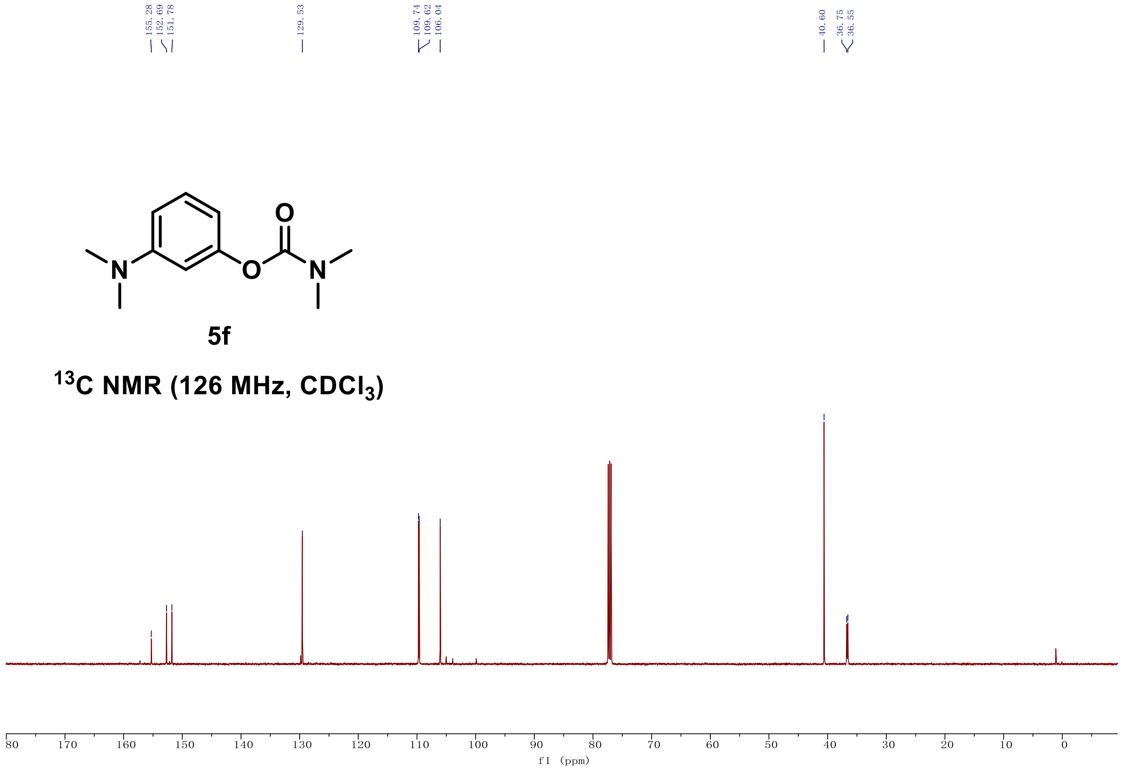
5f
$^{13}$C NMR (126 MHz, CDCl$_3$)
f1 (ppm)

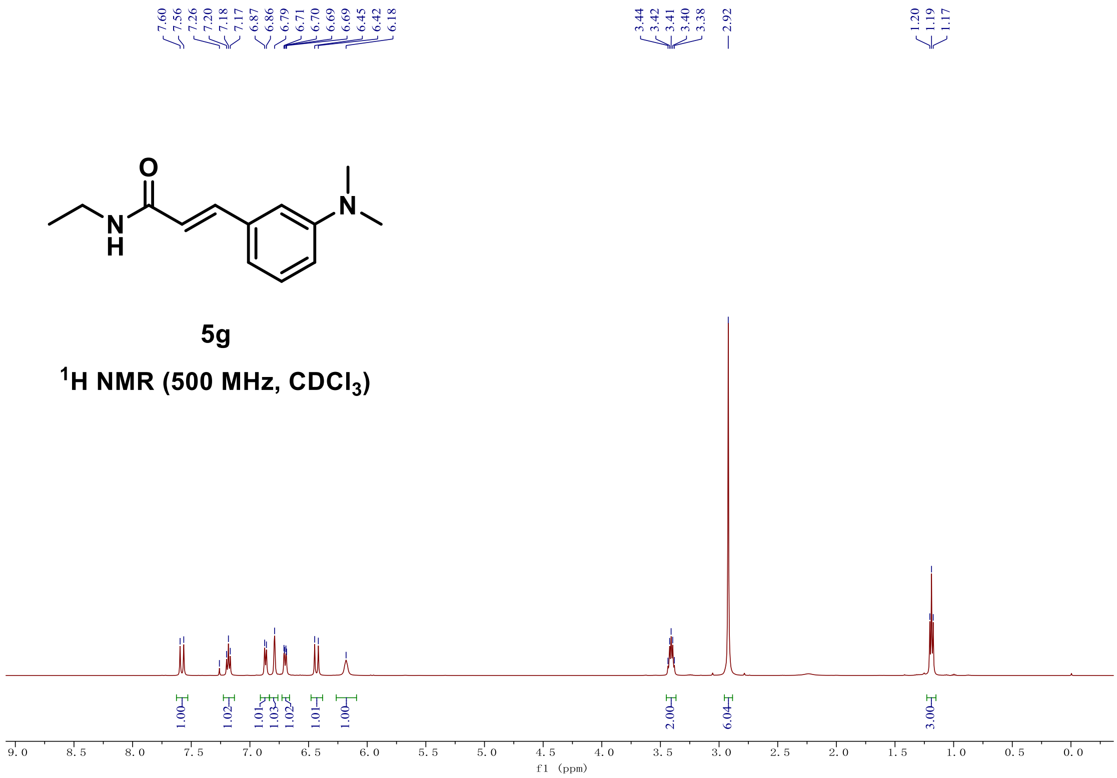


**5g**

**$^{1}H$ NMR (500 MHz, $CDCl_3$)**

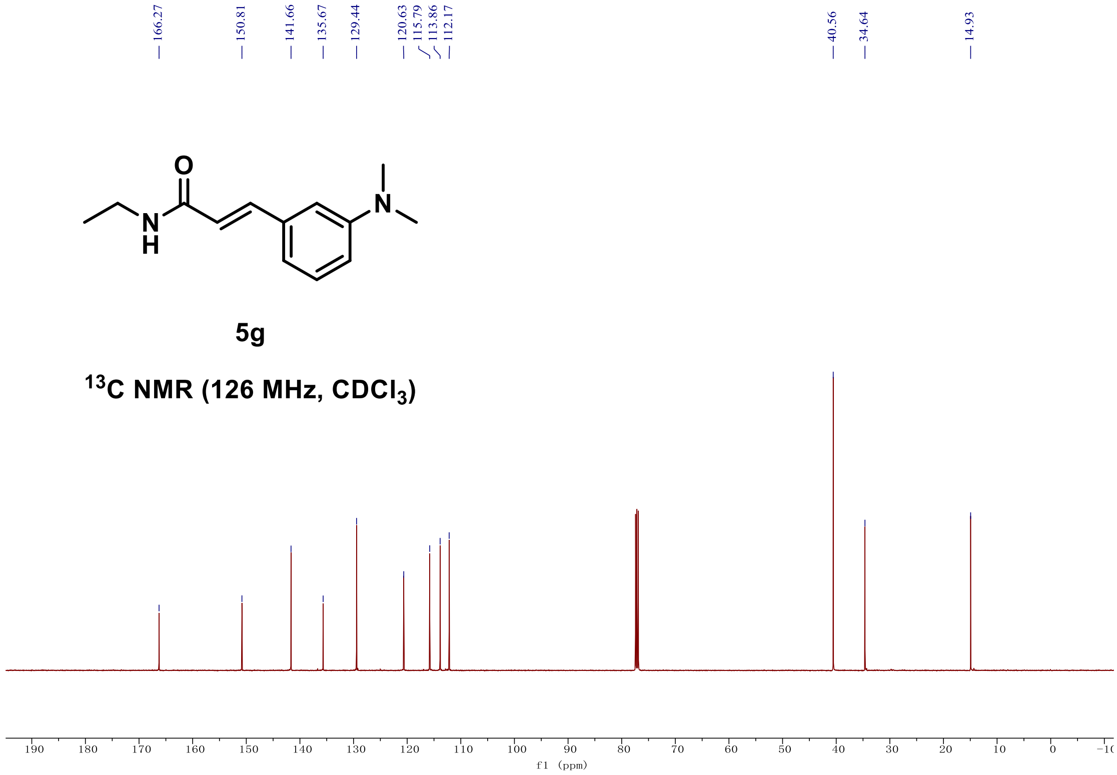


**5g**

**$^{13}C$ NMR (126 MHz, $CDCl_3$)**

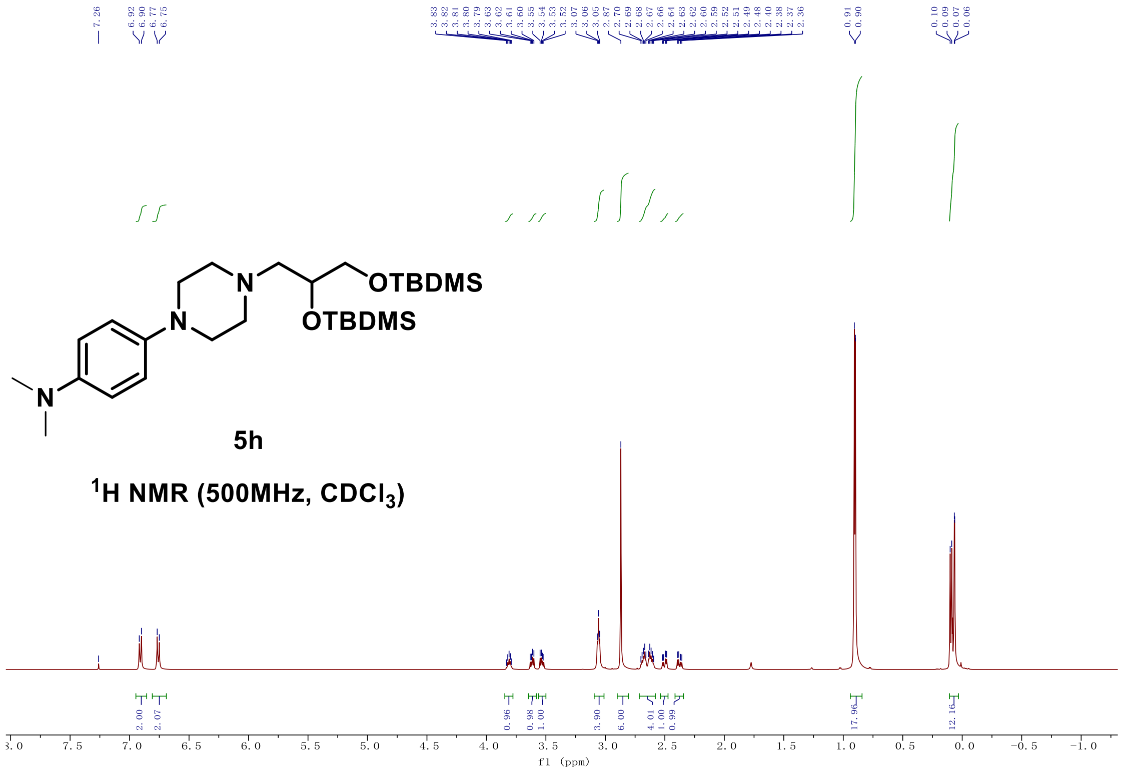

5h

$^{1}$H NMR (500MHz, $CDCl_3$)

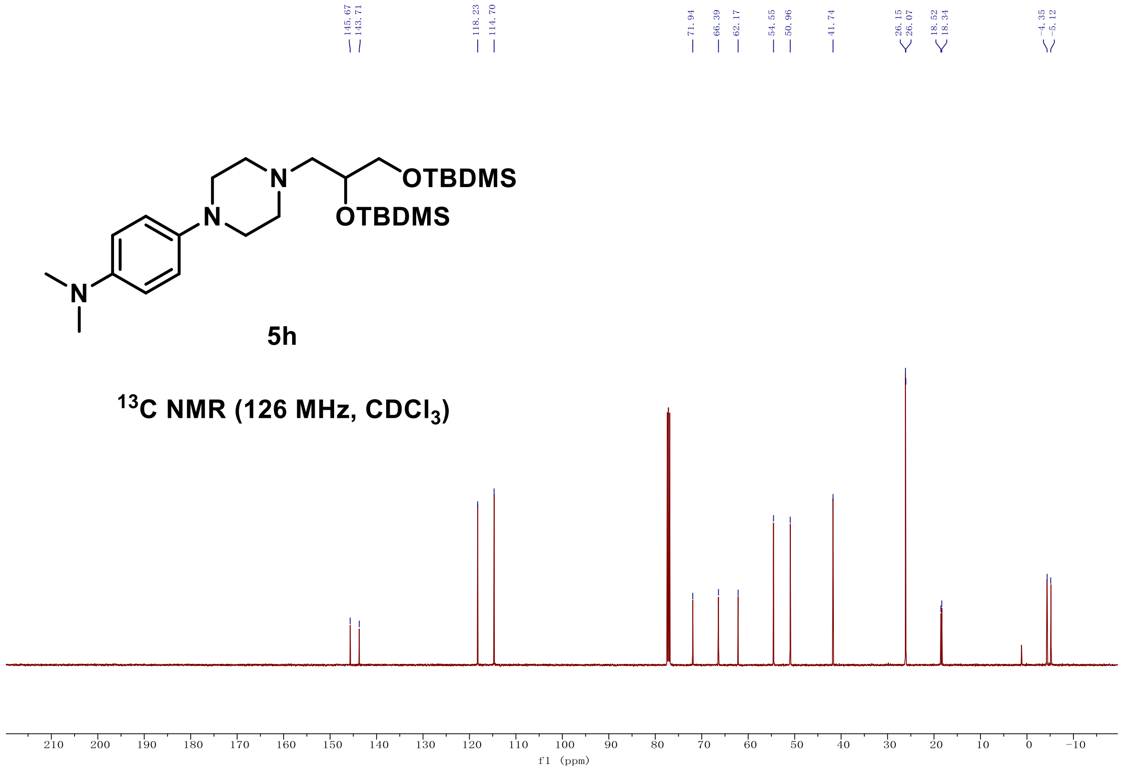

5h

$^{13}$C NMR (126 MHz, $CDCl_3$)

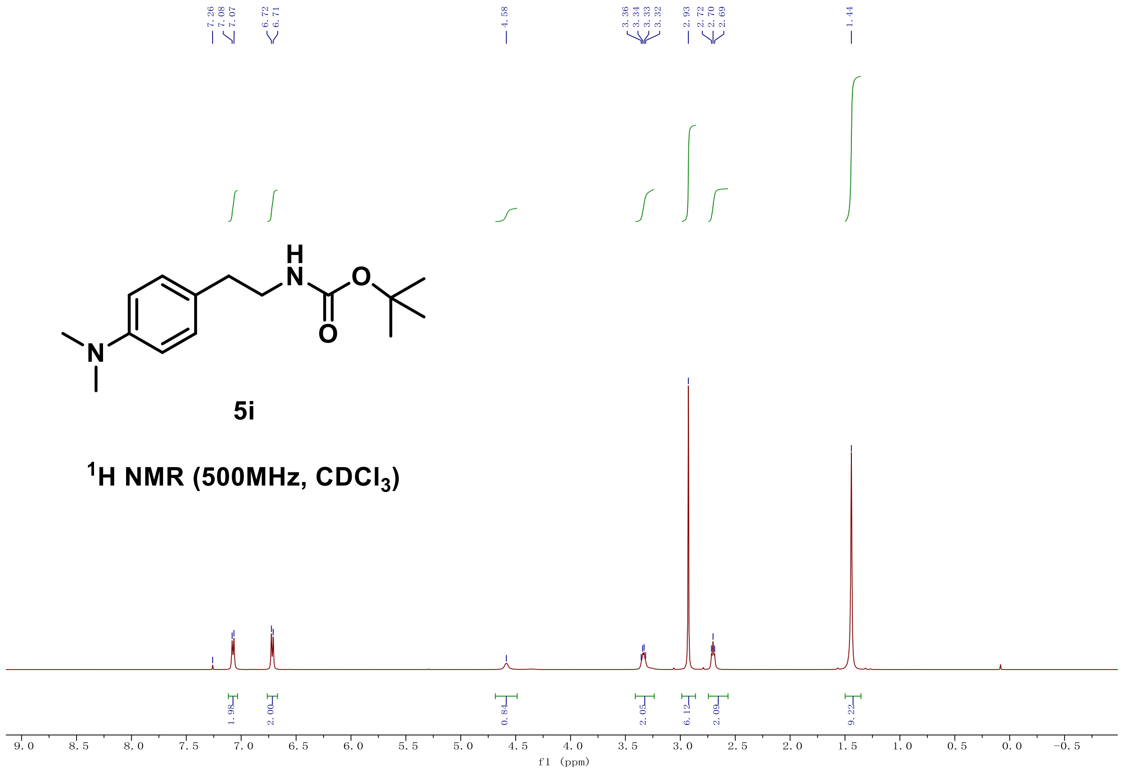


5i

$^{1}H$ NMR (500MHz, $CDCl_3$)

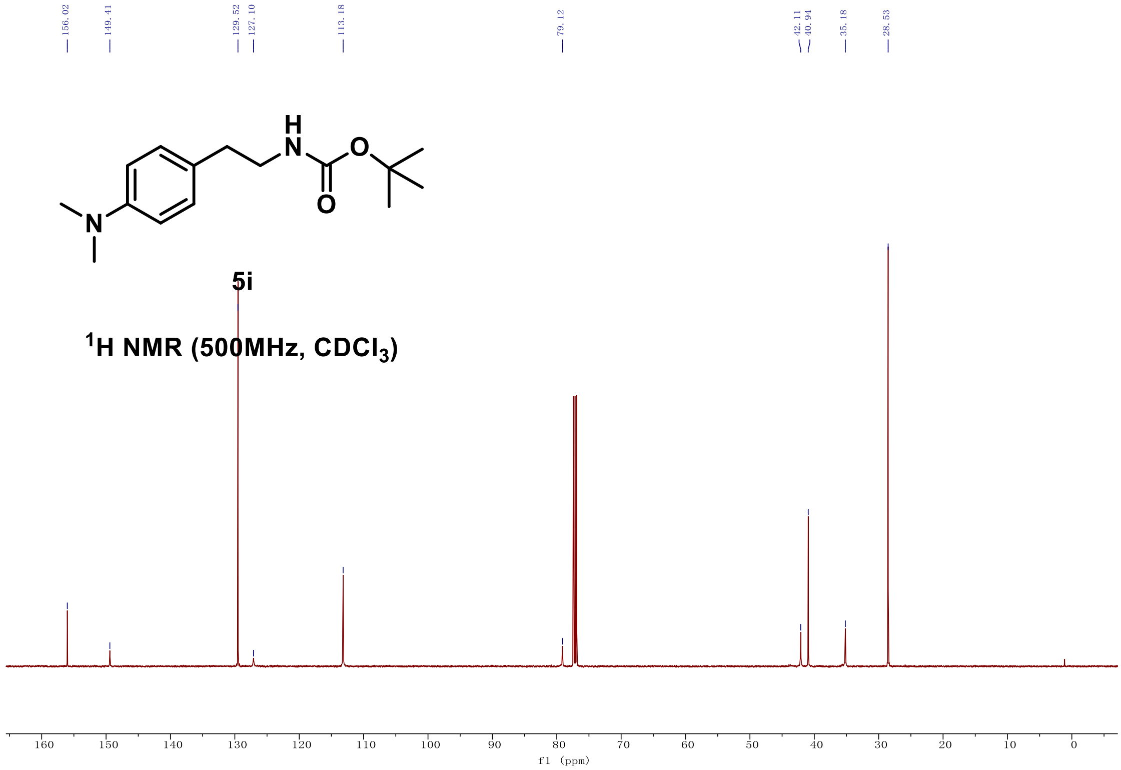


5i

$^{1}H$ NMR (500MHz, $CDCl_3$)

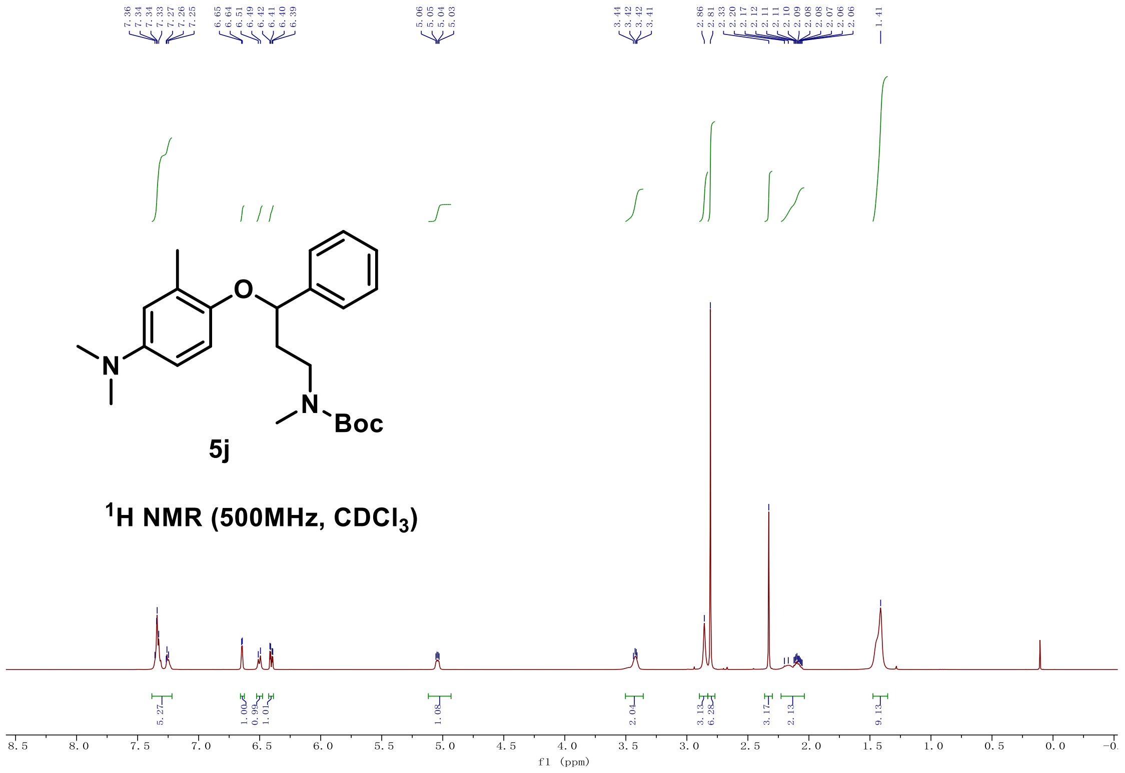
7.36 7.34 7.34 7.33 7.27 7.26 7.25 6.65 6.64 6.51 6.49 6.42 6.41 6.40 6.39 5.06 5.05 5.04 5.03 3.44 3.42 3.42 3.41 2.86 2.81 2.33 2.20 2.17 2.12 2.11 2.10 2.09 2.08 2.08 2.07 2.06 2.06 1.41
N
O
N
Boc
5j
1H NMR (500MHz, CDCl3)
5.27 1.00 0.99 1.01 1.08 2.04 3.13 6.28 3.17 2.13 9.13
f1 (ppm)

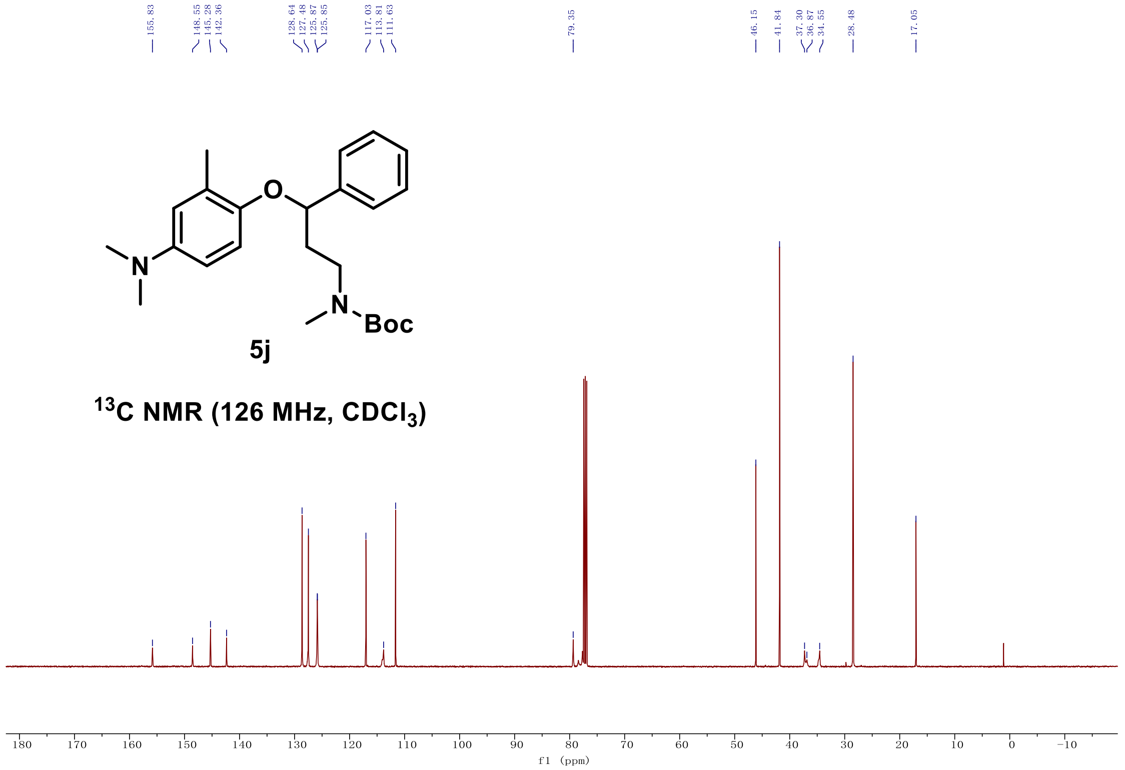
155.83 148.55 145.28 142.36 128.64 127.48 125.87 125.85 117.03 113.81 111.63 79.35 46.15 41.84 37.30 36.87 34.55 28.48 17.05
N
O
N
Boc
5j
13C NMR (126 MHz, CDCl3)
f1 (ppm)

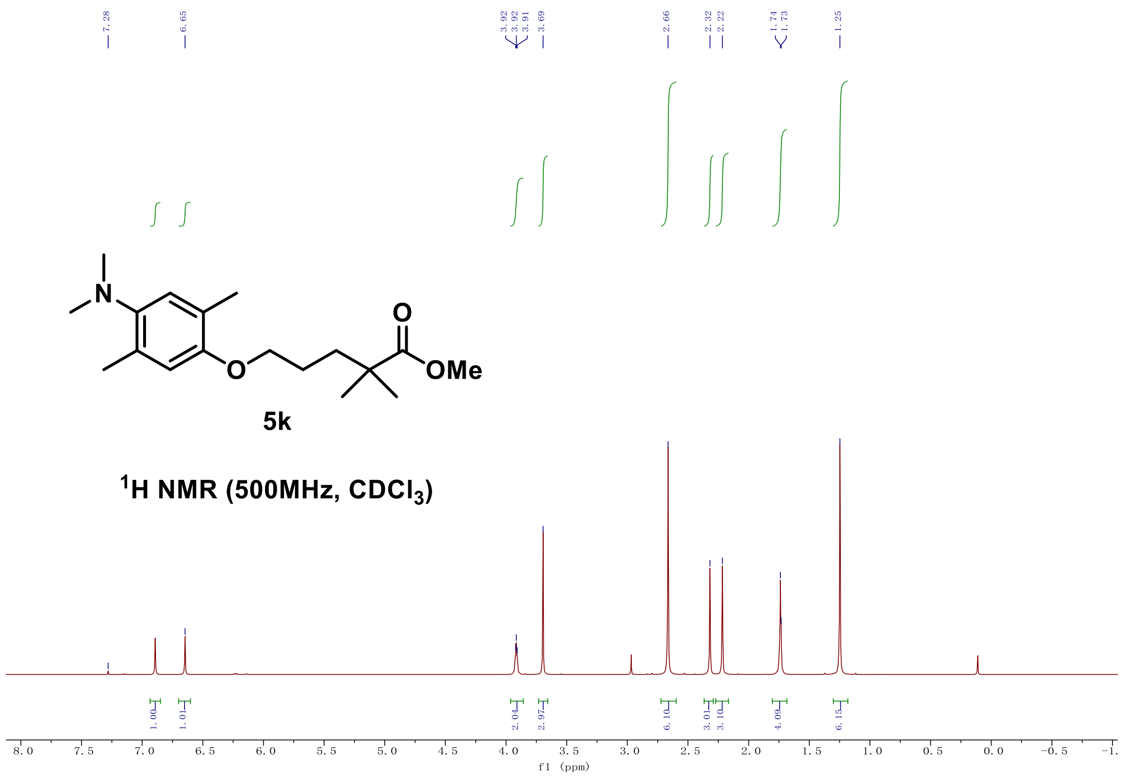


**5k**

$^{1}$H NMR (500MHz, $CDCl_3$)

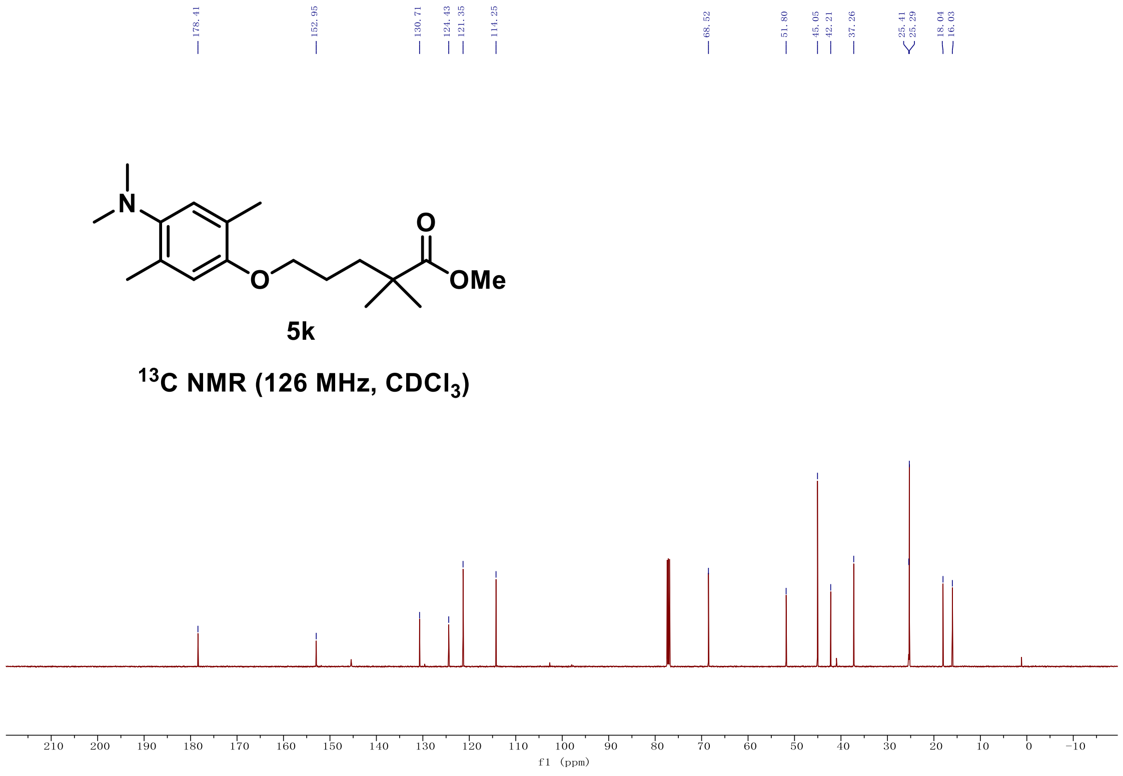


**5k**

$^{13}$C NMR (126 MHz, $CDCl_3$)

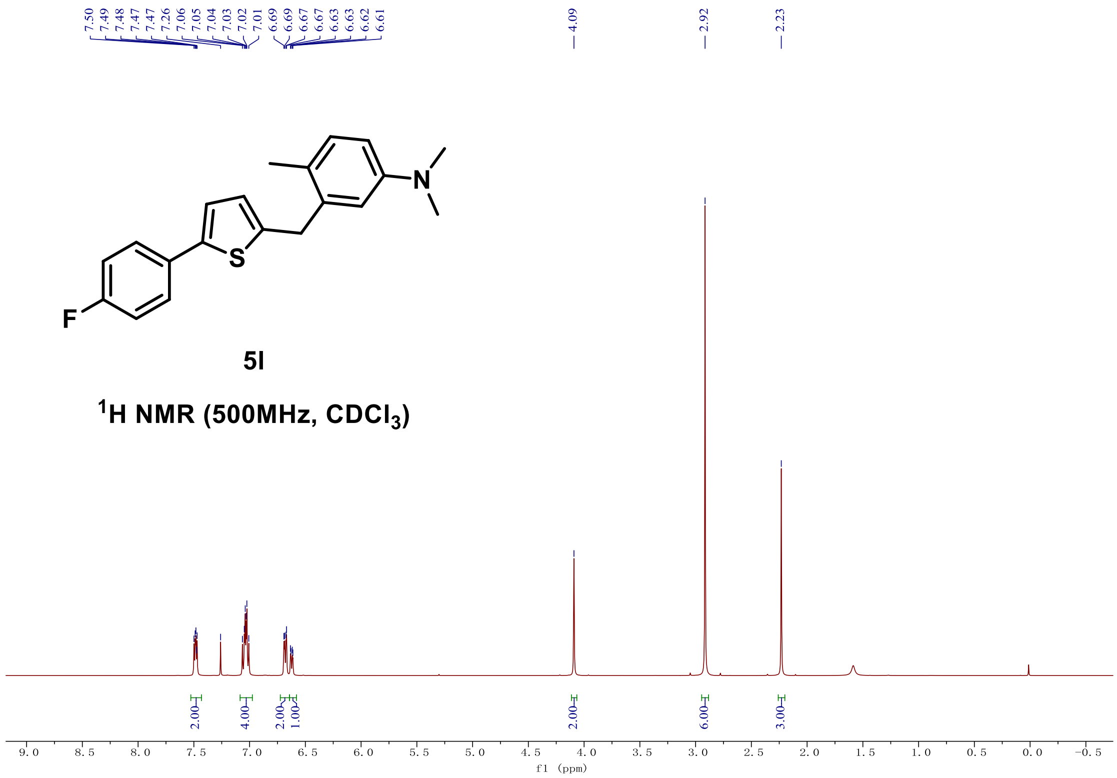
7.50
7.49
7.48
7.47
7.47
7.26
7.06
7.05
7.04
7.03
7.02
7.01
6.69
6.69
6.67
6.67
6.63
6.63
6.62
6.61
4.09
2.92
2.23
F
S
N
5l
$^{1}$H NMR (500MHz, CDCl$_3$)
2.00
4.00
2.00
1.00
2.00
6.00
3.00
f1 (ppm)

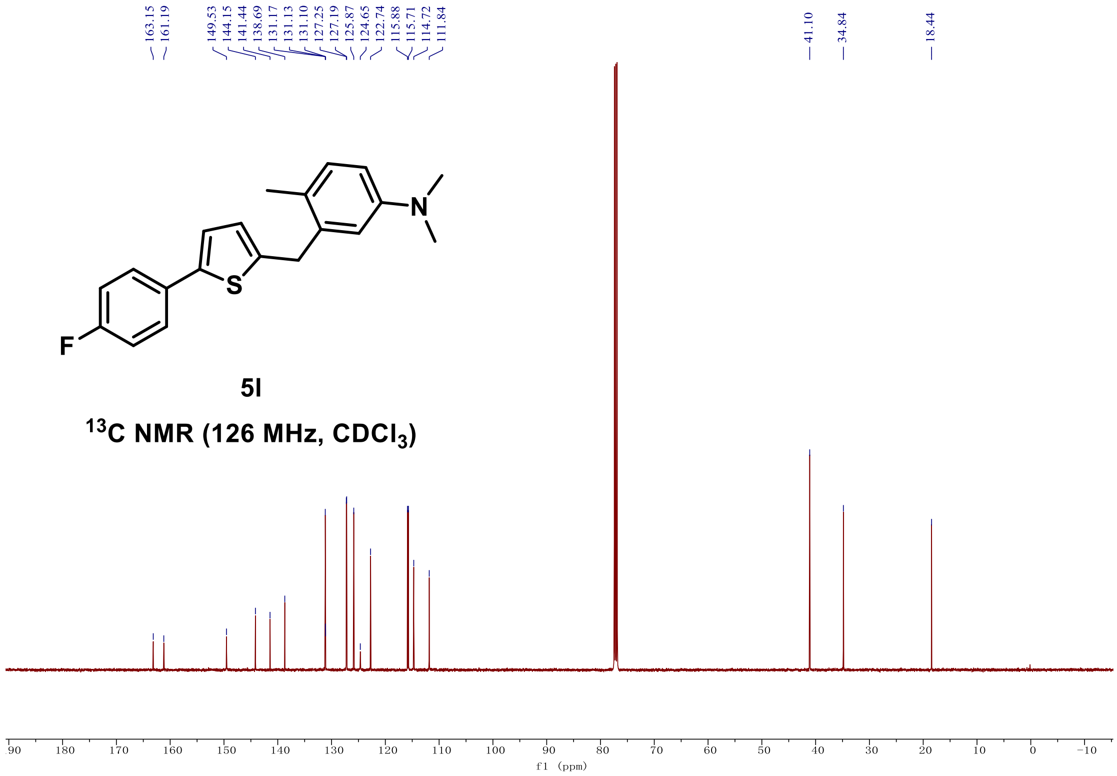
163.15
161.19
149.53
144.15
141.44
138.69
131.17
131.13
131.10
127.25
127.19
125.87
124.65
122.74
115.88
115.71
114.72
111.84
41.10
34.84
18.44
F
S
N
5l
$^{13}$C NMR (126 MHz, CDCl$_3$)
f1 (ppm)

**5l**

**$^{19}$F NMR (471 MHz, CDCl$_3$)**

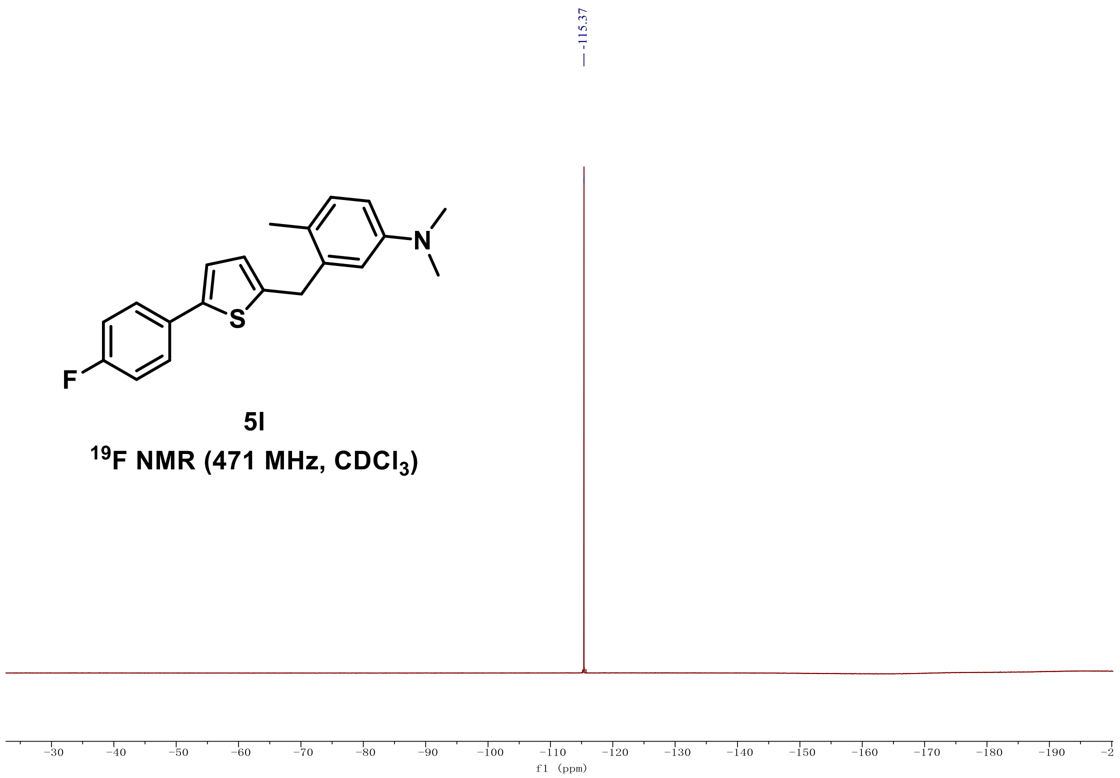


**s5a**

**$^{1}$H NMR (500 MHz, CDCl$_3$)**

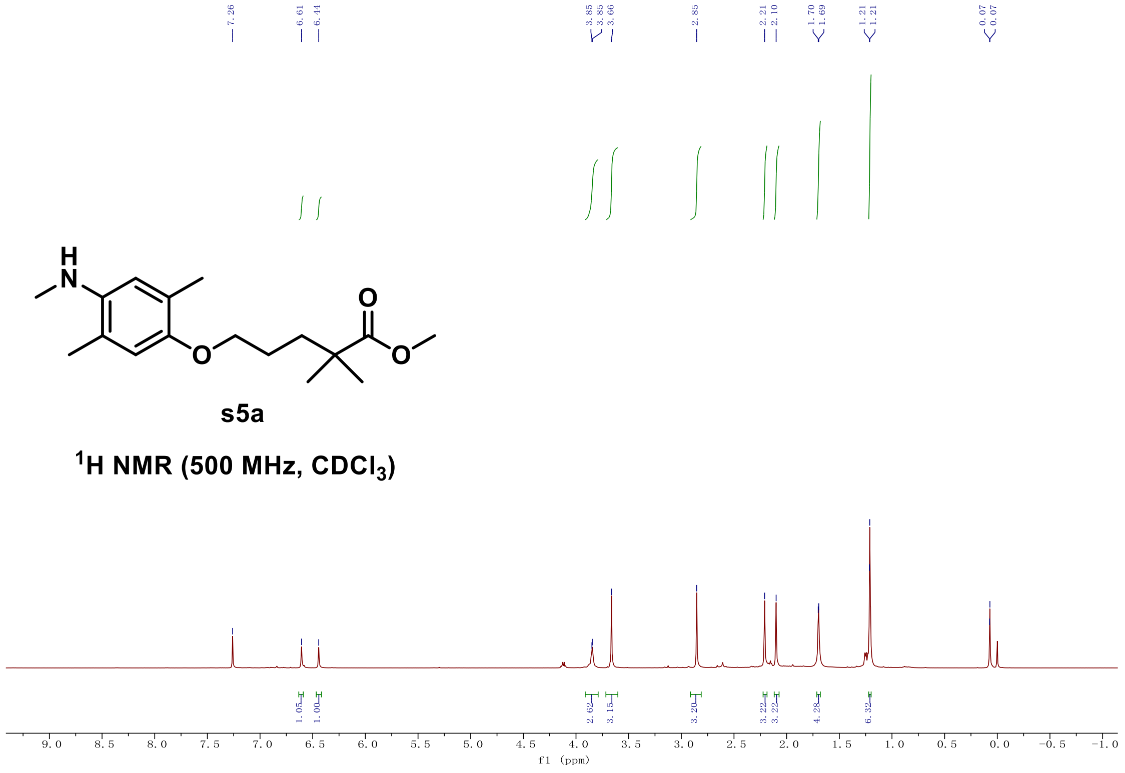

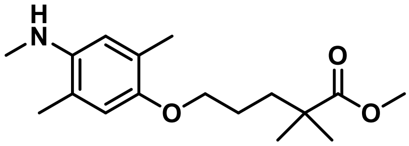

**s5a**

**$^{13}$C NMR (126 MHz, CDCl$_3$)**

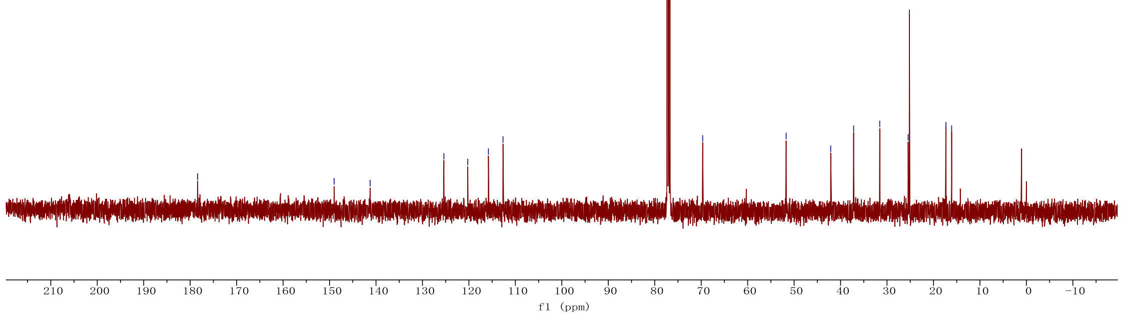


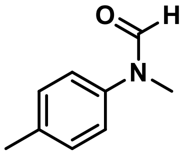

**s5b**

**$^{1}$H NMR (500 MHz, CDCl$_3$)**

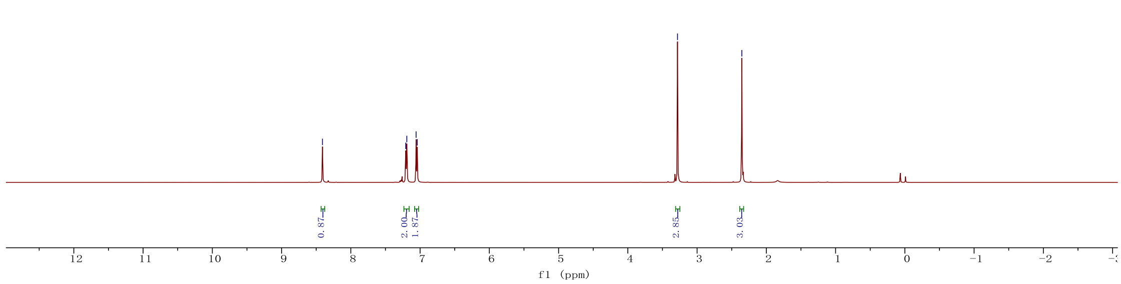

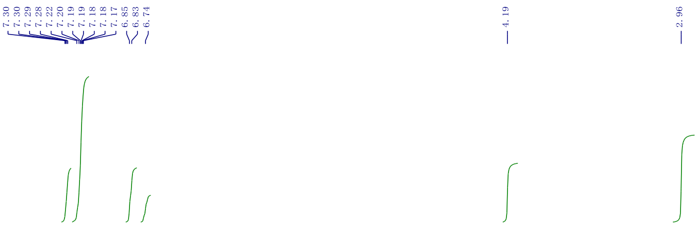


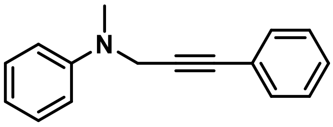

**s5c**

**$^{1}$H NMR (500 MHz, $CDCl_3$)**

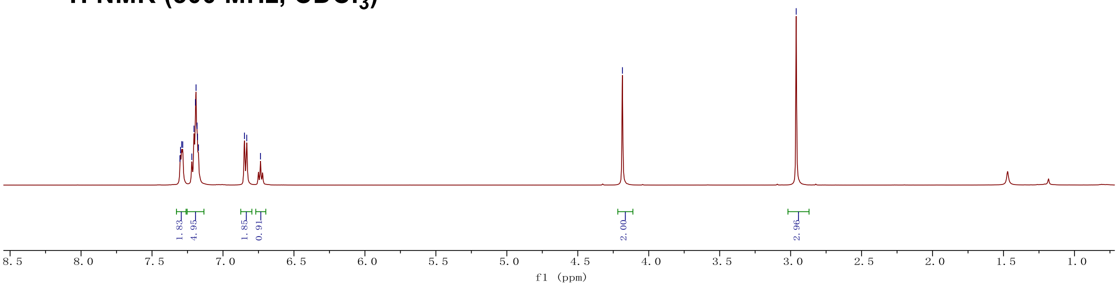


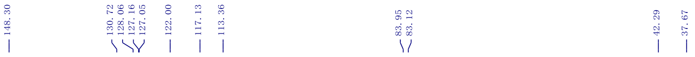


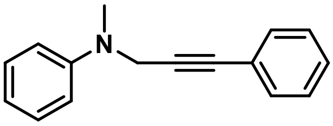

**s5c**

**$^{13}$C NMR (126 MHz, $CDCl_3$)**

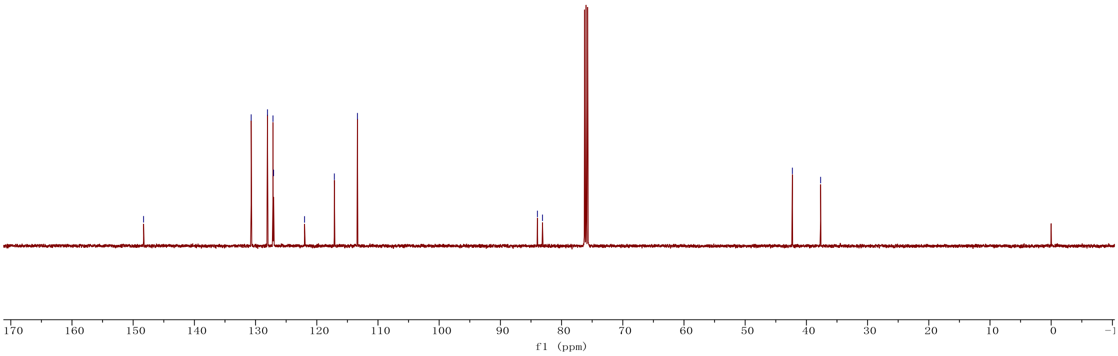

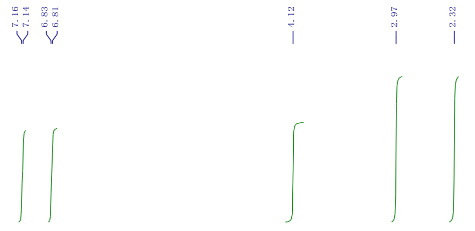


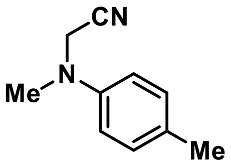


s5d

$^{1}$H NMR (500MHz, $CDCl_3$)

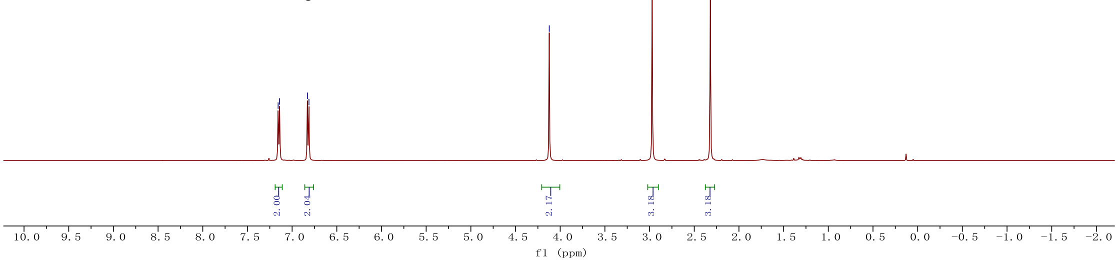


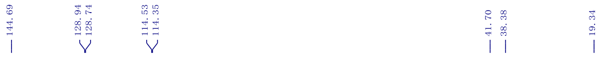


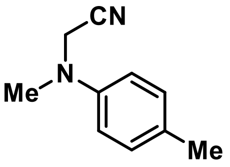


s5d

$^{13}$C NMR (126 MHz, $CDCl_3$)

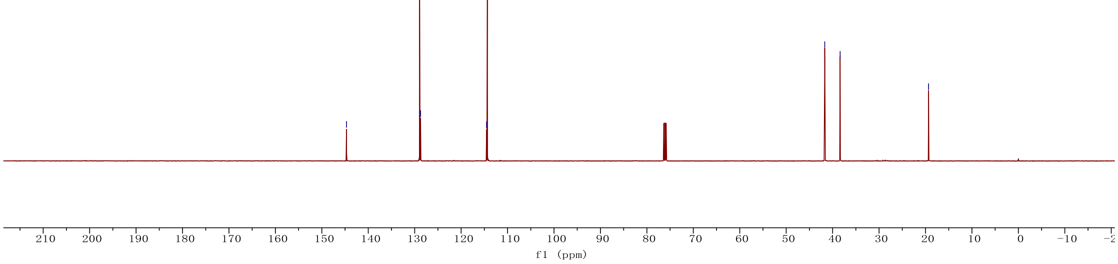

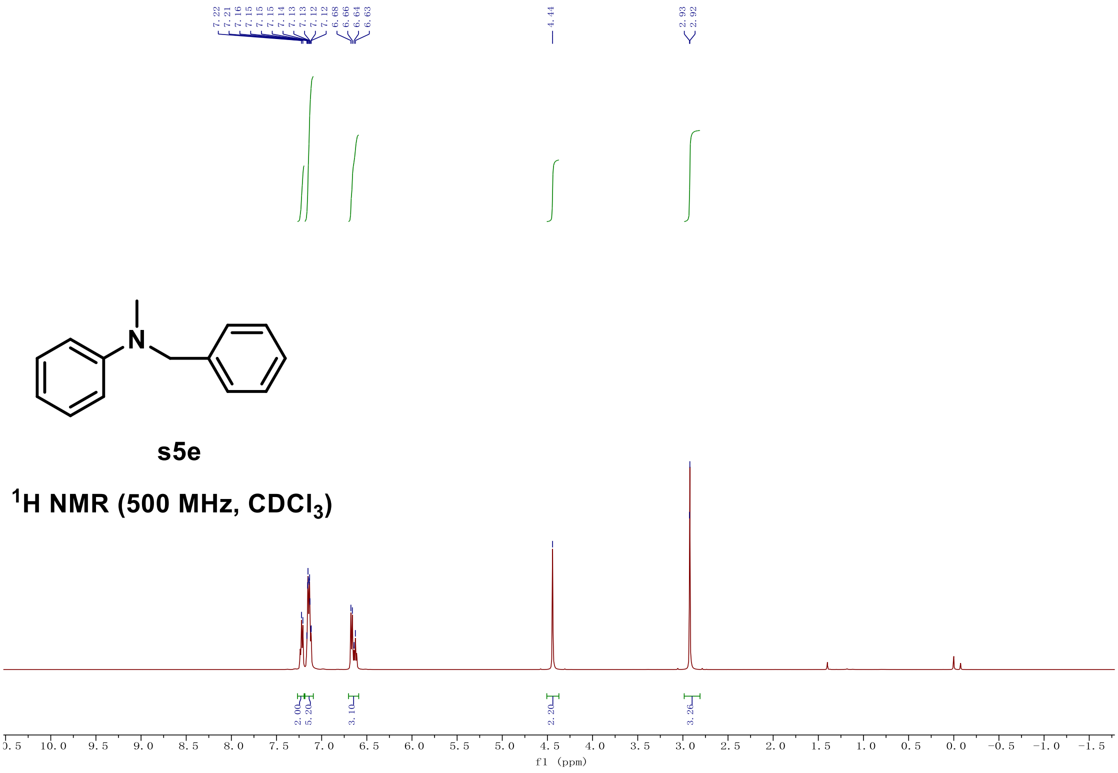
N
s5e
¹H NMR (500 MHz, CDCl₃)
7.22
7.21
7.16
7.15
7.15
7.15
7.14
7.13
7.12
7.12
6.68
6.66
6.64
6.63
4.44
2.93
2.92
2.00
5.20
3.10
2.20
3.26
f1 (ppm)

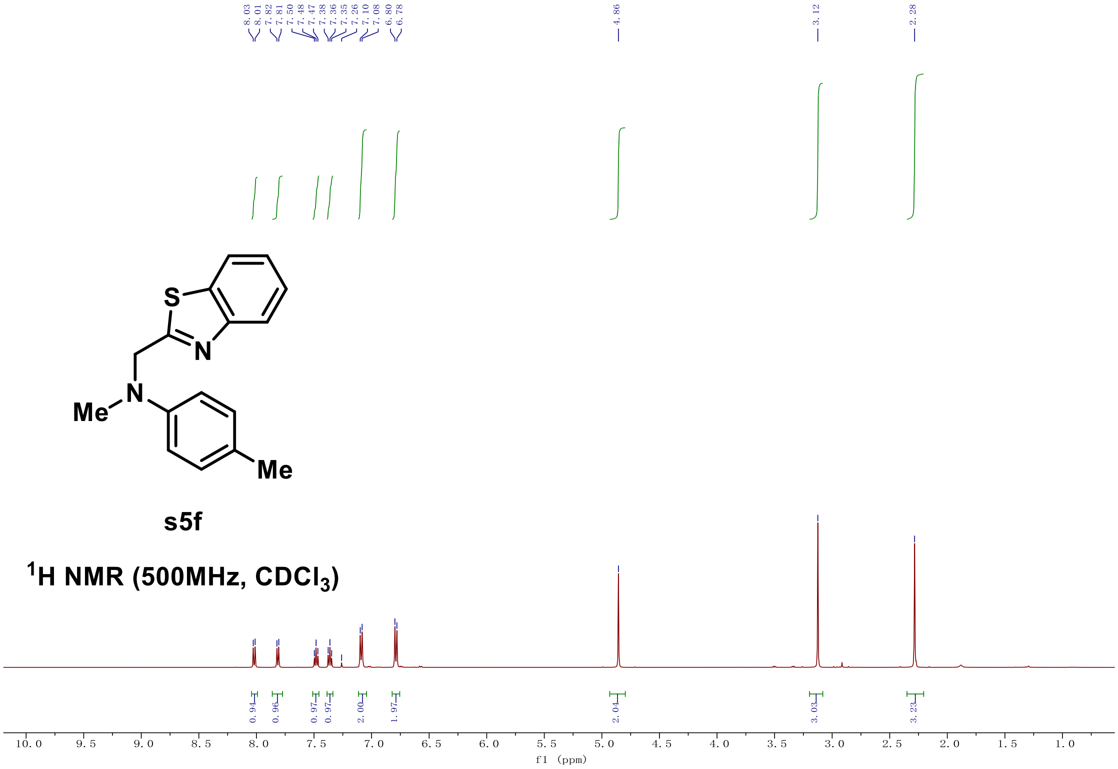
S
N
N
Me
Me
s5f
¹H NMR (500MHz, CDCl₃)
8.03
8.01
7.82
7.81
7.50
7.48
7.47
7.38
7.36
7.35
7.26
7.10
7.08
6.80
6.78
4.86
3.12
2.28
0.94
0.96
0.97
0.97
2.00
1.97
2.04
3.03
3.23
f1 (ppm)

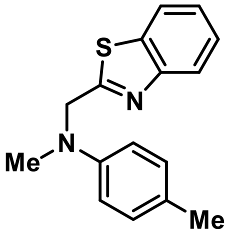

s5f

$^{13}$C NMR (126 MHz, CDCl$_3$)

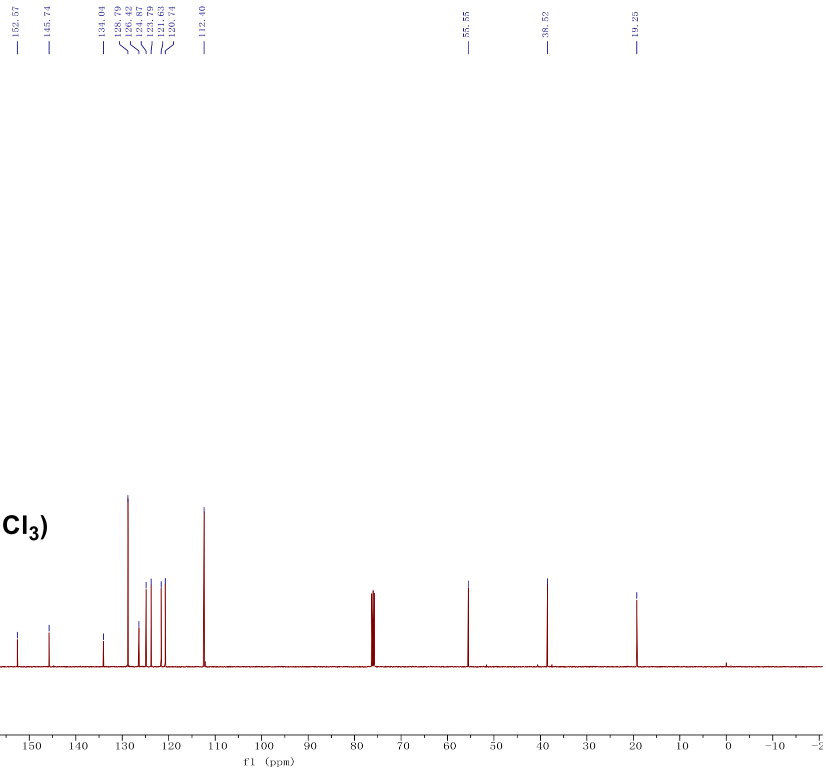


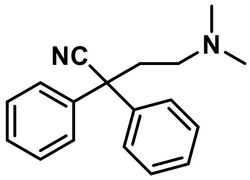

s6a

$^{1}$H NMR (500MHz, CDCl$_3$)

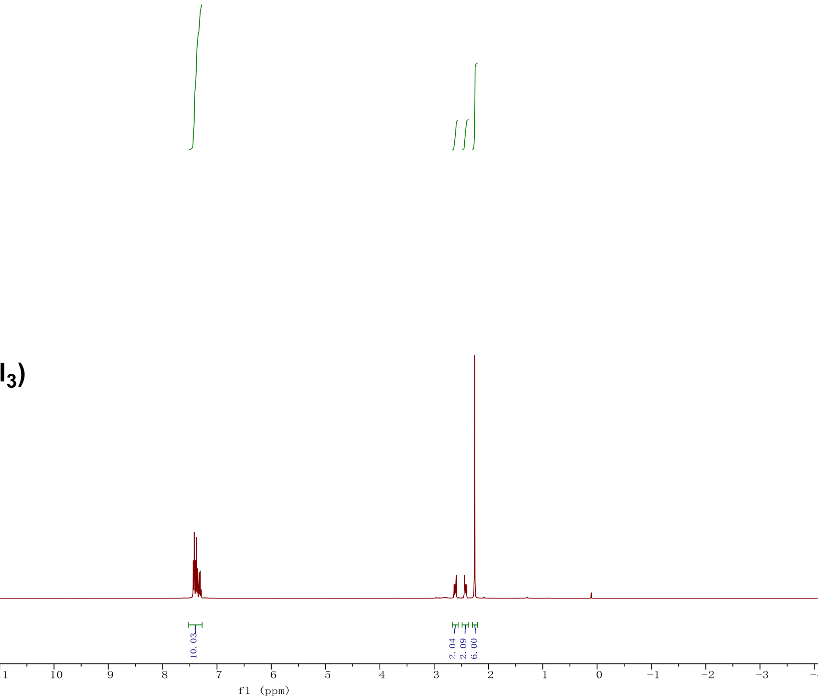

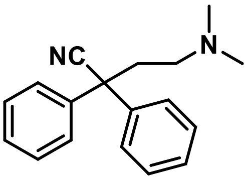

**s6a**

**$^{13}$C NMR (126 MHz, CDCl$_3$)**

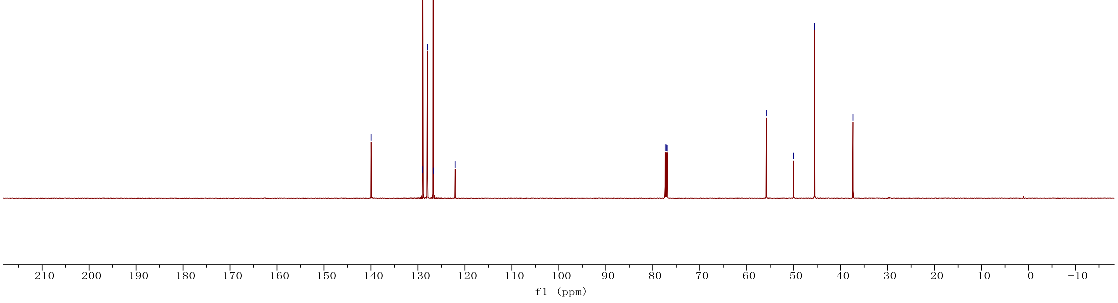


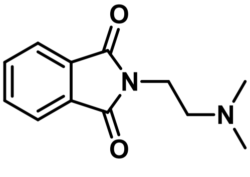

**s6b**

**$^{1}$H NMR (500MHz, CDCl$_3$)**

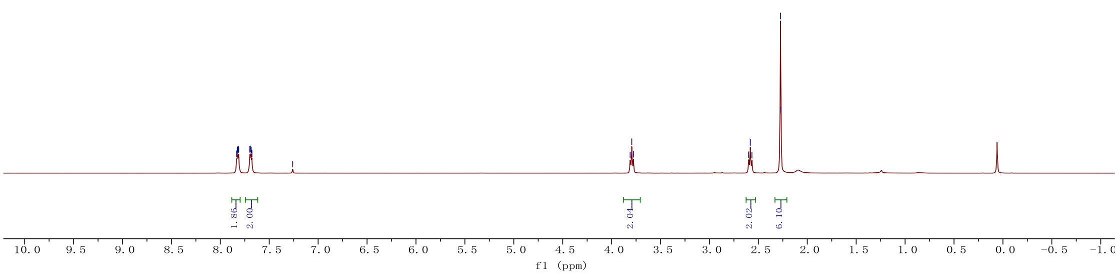

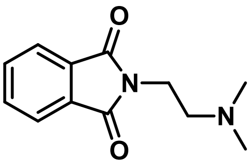

**s6b**

**$^{13}$C NMR (126 MHz, CDCl$_3$)**

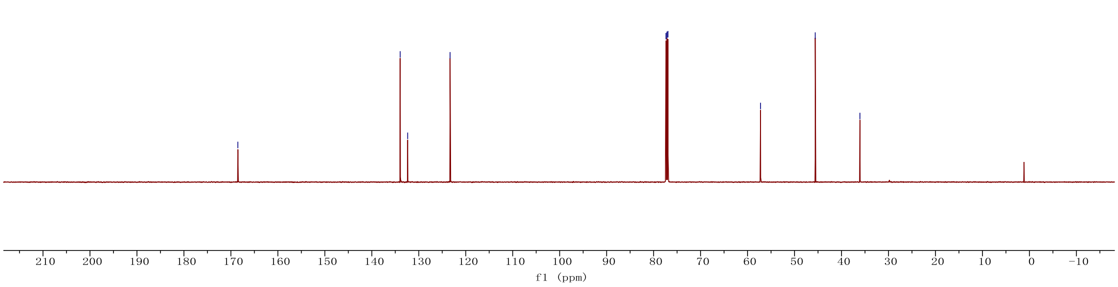


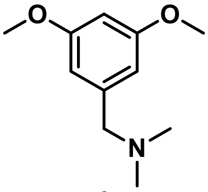

**s6c**

**$^1$H NMR (500MHz, CDCl$_3$)**

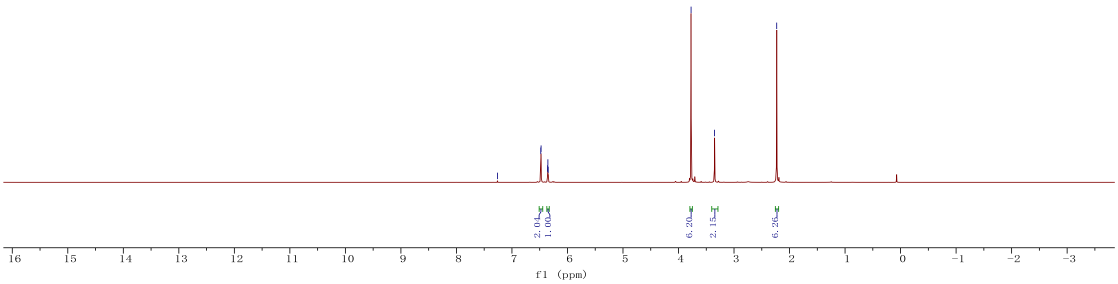

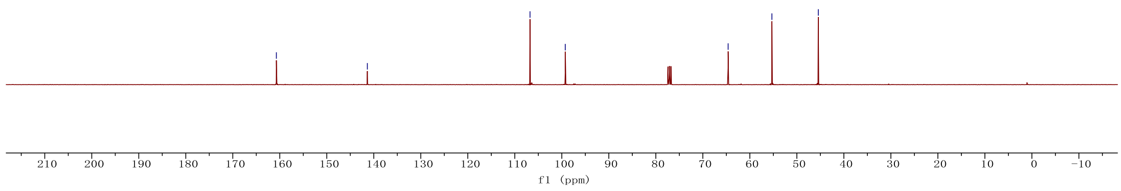


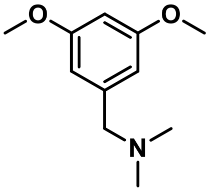

**s6c**

**$^{13}$C NMR (126 MHz, $CDCl_3$)**

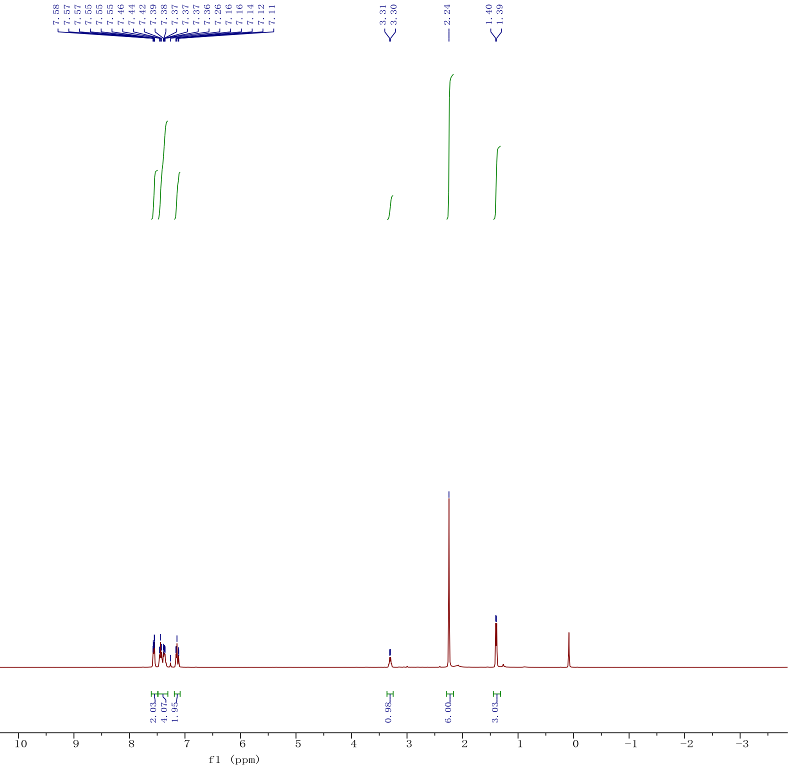


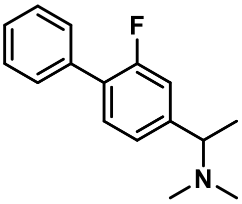

**s6d**

**$^{1}$H NMR (500MHz, $CDCl_3$)**

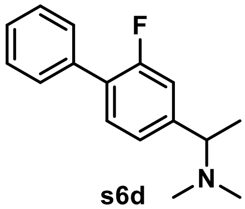


s6d

**$^{13}$C NMR (126 MHz, $CDCl_3$)**

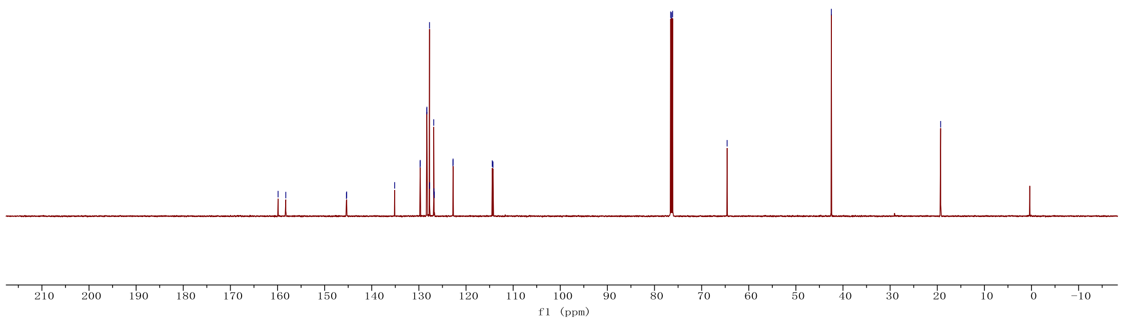


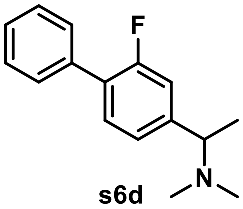


s6d

**$^{19}$F NMR (471 MHz, $CDCl_3$)**

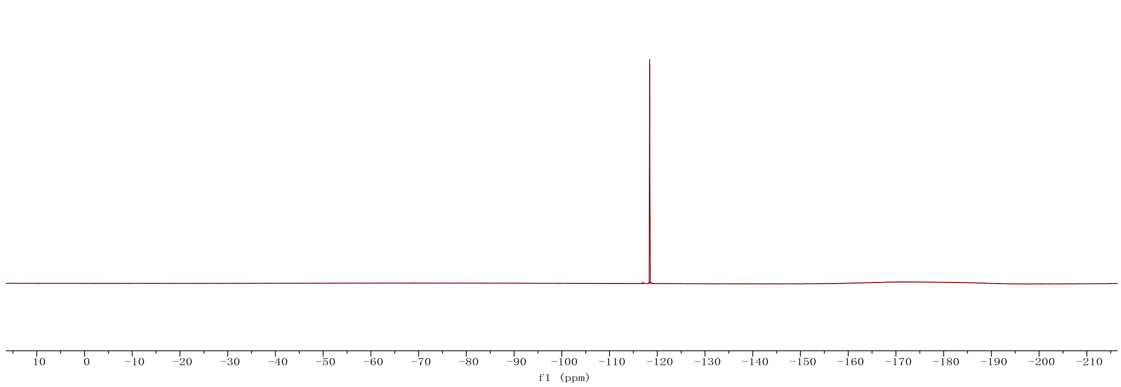

# 6. References

(1) Ma, S.-S.; Sun, R.; Zhang, Z.-H.; Guan, P.-X.; Lin, J.-Q.; Li, C.-S.; Xu, B.-H. Co(Dppbsa)-Catalyzed Reductive *N,N*-Dimethylation of Nitroaromatics with $CO_2$ and Hydrosilane. *Green Chem.* **25**, 8625-8632 (2023).

(2) Zheng, J.; Darcel, C.; Sortais, J.-B. Methylation of Secondary Amines with Dialkyl Carbonates and Hydrosilanes Catalysed by Iron Complexes. *Chem. Commun*. **50**, 14229-14232 (2014).

(3) Kuhlmann, J. H.; Uygur, M.; García Mancheño, O. Protodesilylation of Arylsilanes by Visible-Light Photocatalysis. *Org. Lett.* **24**, 1689-1694 (2022).

(4) Sorribes, I.; Junge, K.; Beller, M. General Catalytic Methylation of Amines with Formic Acid under Mild Reaction Conditions. *Chem. - Eur. J.* **20**, 7878-7883 (2014).

(5) Jiang, X.; Wang, C.; Wei, Y.; Xue, D.; Liu, Z.; Xiao, J. A General Method for N-Methylation of Amines and Nitro Compounds with Dimethylsulfoxide. *Chem. - Eur. J.* **20**, 58-63 (2014).

(6) Zhang, Y.; César, V.; Storch, G.; Lugan, N.; Lavigne, G. Skeleton Decoration of NHCs by Amino Groups and Its Sequential Booster Effect on the Palladium-Catalyzed Buchwald-Hartwig Amination. *Angew. Chem. Int. Ed.* **53**, 6482-6486 (2014**)**.

(7) Zhang, J.; Wei, R.; Ren, C.; Liu, L. L.; Wu, L. Si-B Functional Group Exchange Reaction Enabled by a Catalytic Amount of $BH_3$ : Scope, Mechanism, and Application. *J. Am. Chem. Soc.* **145**, 15619-15629 (2023).

(8) Nikitas, N. F.; Theodoropoulou, M. A.; Kokotos, C. G. Photochemical Reaction of *N,N*-Dimethylanilines with *N*-Substituted Maleimides Utilizing Benzaldehyde as the Photoinitiator. *Eur. J. Org. Chem.* **2021**, 1168-1173 (2021).

(9) Li, J.; Huang, C.; Wen, D.; Zheng, Q.; Tu, B.; Tu, T. Nickel-Catalyzed Amination of Aryl Chlorides with Amides. *Org. Lett.* **23**, 687-691 (2021).

(10) Schmidt, A.; Habeck, T.; Snovydovych, B.; Eisfeld, W. Addition Reactions and Redox Esterifications of Carbonyl Compounds by *N*-Heterocyclic Carbenes of Indazole. *Org. Lett.* **9**, 3515-3518 (2007**)**.

(11) Wang, W.; Liu, J.; Yang, L.; Song, S.; Jiao, N. A Catalytic Method to Activate Nitromethane by the Cooperation of Homo- and Heterogeneous Catalysis. *Angew. Chem. Int. Ed.* 2024, e202312354.

(12) Shet, H.; Patel, M.; Waikar, J. M.; More, P. M.; Sanghvi, Y. S.; Kapdi, A. R. Room-Temperature Dialkylamination of Chloroheteroarenes Using a Cu(II)/PTABS Catalytic System. *Chem.- Asian J.* **18**, e202201006, (2023).

(13) Yang, B.; Wang, Z.-X. Transition-Metal-Free Cross-Coupling of Aryl and Heteroaryl Thiols with Arylzinc Reagents. *Org. Lett.* **19**, 6220-6223 (2017).

(14) Conte, S.; Rodríguez-Calero, G. G.; Burkhardt, S. E.; Lowe, M. A.; Abruña, H. D. Designing Conducting Polymer Films for Electrochemical Energy Storage Technologies. *RSC Adv*. **3**, 1957-1964 (2013).

(15) Towards Sustainable Catalysis-Highly Efficient Olefin Metathesis in Protic Media Using Phase

Labelled Cyclic Alkyl Amino Carbene (CAAC) Ruthenium Catalysts. *Chem. Cat. Chem.* **12**, 1953-1957 (2020).

(16) Li, X.; Li, F.; Xu, Y.; Xiao, L.; Xie, J.; Zhou, Q. Hydrogenation of Esters by Manganese Catalysts. *Adv. Synth. Catal.* **364**, 744-749 (2022).

(17) Hou, C.; He, Q.; Yang, C. Direct Synthesis of Diverse 2-Aminobenzo[*b*]Thiophenes via Palladium-Catalyzed Carbon-Sulfur Bond Formation Using $Na_2S_2O_3$ as the Sulfur Source. *Org. Lett.* **16**, 5040-5043 (2014).

(18) Shunmughanathan, M.; Puthiaraj, P.; Pitchumani, K. Melamine-Based Microporous Network Polymer Supported Palladium Nanoparticles: A Stable and Efficient Catalyst for the Sonogashira Coupling Reaction in Water. *Chem. Cat. Chem.* **7**, 666-673 (2015).

(19) Pandey, G.; Tiwari, S. K.; Singh, B.; Vanka, K.; Jain, S. *P* Selective ($Sp^2$)-C-H Functionalization for an Acylation/Alkylation Reaction Using Organic Photoredox Catalysis. *Chem. Commun.* **53**, 12337-12340 (2017).

(20) Tran, C.; Gallavardin, T.; Petit, M.; Slimi, R.; Dhimane, H.; Blanchard-Desce, M.; Acher, F. C.; Ogden, D.; Dalko, P. I. Two-Photon "Caging" Groups: Effect of Position Isomery on the Photorelease Properties of Aminoquinoline-Derived Photolabile Protecting Groups. *Org. Lett.* **17**, 402-405 (2015).

(21) Torigoe, T.; Ohmura, T.; Suginome, M. Asymmetric Cycloisomerization of *o*-Alkenyl-*N*-Methylanilines to Indolines by Iridium-Catalyzed C($Sp^3$)-H Addition to Carbon-Carbon Double Bonds. *Angew. Chem. Int. Ed.* **56**, 14272-14276 (2017).

(22) Abe, M.; Nitta, S.; Miura, E.; Kimachi, T.; Inamoto, K. Nitrile Synthesis via Desulfonylative-Smiles Rearrangement. *J. Org. Chem.* **87**, 4460-4467 (2022).

(23) Salvi, L.; Davis, N. R.; Ali, S. Z.; Buchwald, S. L. A New Biarylphosphine Ligand for the Pd-Catalyzed Synthesis of Diaryl Ethers under Mild Conditions. *Org. Lett.* **14**, 170-173 (2012).

(24) Guo, X.; Lv, C.; Mahmood, Q.; Zhou, L.; Xu, G.; Wang, Q. Solvent-Controlled Chemoselective *N*-Dealkylation-*N* -Nitrosation or *C*-Nitration of *N*-Alkyl Anilines with *Tert*-Butyl Nitrite. *Org. Chem. Front.* **6**, 3401-3407 (2019).

(25) Nishizawa, A.; Takahira, T.; Yasui, K.; Fujimoto, H.; Iwai, T.; Sawamura, M.; Chatani, N.; Tobisu, M. Nickel-Catalyzed Decarboxylation of Aryl Carbamates for Converting Phenols into Aromatic Amines. *J. Am. Chem. Soc.* **141**, 7261-7265 (2019).

(26) Senthamarai, T.; Murugesan, K.; Natte, K.; Kalevaru, N. V.; Neumann, H.; Kamer, P. C. J.; Jagadeesh, R. V. Expedient Synthesis of *N*-Methyl- and *N*-Alkylamines by Reductive Amination Using Reusable Cobalt Oxide Nanoparticles. *Chem. Cat. Chem.* **10**, 1235-1240 (2018).

(27) Buchynskyy, A.; Gillespie, J. R.; Herbst, Z. M.; Ranade, R. M.; Buckner, F. S.; Gelb, M. H. 1-Benzyl-3-Aryl-2-Thiohydantoin Derivatives as New Anti- *Trypanosoma Brucei* Agents: SAR and in Vivo Efficacy. *ACS Med. Chem. Lett.* **8***,* 886-891 (2017).

(28) Zhang, B.-S.; Jia, W.-Y.; Wang, Y.-M.; Oliveira, J. C. A.; Warratz, S.; Zhang, Z.-Q.; Gou, X.-Y.; Liang, Y.-M.; Wang, X.-C.; Quan, Z.-J.; Ackermann, L. Template Synthesis to Solve the

Unreachable *Ortho* C-H Functionalization Reaction of Aryl Iodide. *J. Org. Chem.* **88**, 16539-16546 (2023).

(29) Li, H.; Gonçalves, T. P.; Zhao, Q.; Gong, D.; Lai, Z.; Wang, Z.; Zheng, J.; Huang, K.-W. Diverse Catalytic Reactivity of a Dearomatized $PN^3$ P*-Nickel Hydride Pincer Complex towards $CO_2$ Reduction. *Chem. Commun.* **54**, 11395-11398 (2018).

(30) Zhu, F.; Tao, J.-L.; Wang, Z.-X. Palladium-Catalyzed C-H Arylation of (Benzo)Oxazoles or (Benzo)Thiazoles with Aryltrimethylammonium Triflates. *Org. Lett.* **17**, 4926-4929 (2015).

(31) Liu, X.; Xiang, M.; Tong, Z.; Luo, F.; Chen, W.; Liu, F.; Wang, F.; Yu, R.-Q.; Jiang, J.-H. Activatable Fluorescence Probe via Self-Immolative Intramolecular Cyclization for Histone Deacetylase Imaging in Live Cells and Tissues. *Anal. Chem.* **90**, 5534-5539 (2018).

(32) Mao, W.; Tang, J.; Dai, L.; He, X.; Li, J.; Cai, L.; Liao, P.; Jiang, R.; Zhou, J.; Wu, H. A General Strategy to Design Highly Fluorogenic Far-Red and Near-Infrared Tetrazine Bioorthogonal Probes. *Angew. Chem. Int. Ed.* **60**, 2393-2397 (2021).

(33) Cheng, C.-C.; Chang, C.-P.; Yu, W.-S.; Hung, F.-T.; Liu, Y.-I.; Wu, G.-R.; Chou, P.-T. Comprehensive Studies on Dual Excitation Behavior of Double Proton versus Charge Transfer in 4-(*N*-Substituted Amino)-1*H*-Pyrrolo[2,3-*b*]Pyridines. *J. Phys. Chem. A*. **107**, 1459-1471 (2003).

(34) Stahl, T.; Müther, K.; Ohki, Y.; Tatsumi, K.; Oestreich, M. Catalytic Generation of Borenium Ions by Cooperative B-H Bond Activation: The Elusive Direct Electrophilic Borylation of Nitrogen Heterocycles with Pinacolborane. *J. Am. Chem. Soc.* **135**, 10978-10981 (2013).

(35) Reeves, J. T.; Malapit, C. A.; Buono, F. G.; Sidhu, K. P.; Marsini, M. A.; Sader, C. A.; Fandrick, K. R.; Busacca, C. A.; Senanayake, C. H. Transnitrilation from Dimethylmalononitrile to Aryl Grignard and Lithium Reagents: A Practical Method for Aryl Nitrile Synthesis. *J. Am. Chem. Soc.* **137**, 9481-9488 (2015).

(36) Osuský, P.; Smolíček, M.; Nociarová, J.; Rakovský, E.; Hrobárik, P. One-Pot Reductive Methylation of Nitro- and Amino-Substituted (Hetero)Aromatics with DMSO/HCOOH: Concise Synthesis of Fluorescent Dimethylamino-Functionalized Bibenzothiazole Ligands with Tunable Emission Color upon Complexation. *J. Org. Chem.* **87**, 10613-10629 (2022).

(37) Lee, B. K.; Biscoe, M. R.; Buchwald, S. L. Simple, Efficient Protocols for the Pd-Catalyzed Cross-Coupling Reaction of Aryl Chlorides and Dimethylamine. *Tetrahedron Lett.* **50**, 3672-3674 (2009).

(38) Chang, K.; Liu, Y.; Liu, J.; Peng, Y.; Yang, Y.; Li, Z.; Jheng, R.; Chao, C.; Liu, K.; Chou, P. Catalytic-Type Excited-State N-H Proton-Transfer Reaction in 7-Aminoquinoline and Its Derivatives. *Chem.-Eur. J.* **25**, 14972-14982 (2019).

(39) Lee, B. K.; Biscoe, M. R.; Buchwald, S. L. Simple, Efficient Protocols for the Pd-Catalyzed Cross-Coupling Reaction of Aryl Chlorides and Dimethylamine. *Tetrahedron Lett.* **50**, 3672-3674 (2009).

(40) Luo, M.-J.; Lv, G.-F.; Li, Y.; Li, J.-H. Metal-Free Amino-Controlled Electrochemical Intramolecular C-O and C-N Couplings by Site-Selective Activation of Aryl C-N and C-O Bonds.

*Green Chem.* **23**, 2044-2048 (2021).
(41) Dai, W.-C.; Yang, B.; Xu, S.-H.; Wang, Z.-X. Nickel-Catalyzed Cross-Coupling of Aryl 2-Pyridyl Ethers with Organozinc Reagents: Removal of the Directing Group via Cleavage of the Carbon-Oxygen Bonds. *J. Org. Chem.* **86**, 2235-2243 (2021).
(42) Petersen, T. P.; Larsen, A. F.; Ritzén, A.; Ulven, T. Continuous Flow Nucleophilic Aromatic Substitution with Dimethylamine Generated in Situ by Decomposition of DMF. *J. Org. Chem.* **78**, 4190-4195 (2013).
(43) Wang, W.; Yang, X.; Dai, R.; Yan, Z.; Wei, J.; Dou, X.; Qiu, X.; Zhang, H.; Wang, C.; Liu, Y.; Song, S.; Jiao, N. Catalytic Electrophilic Halogenation of Arenes with Electron-Withdrawing Substituents. *J. Am. Chem. Soc.* **144**, 13415-13425 (2022).
(44) Hirose, W.; Sato, K.; Matsuda, A. Fluorescence Properties of 5-(5,6-Dimethoxybenzothiazol-2-yl)-2′-deoxyuridine ($d^{bt}$ U) and Oligodeoxyribonucleotides Containing $d^{bt}$ U. *Eur. J. Org. Chem.* **2011**, 6206-6217 (2011).
(45) Weekes, A. A.; Bagley, M. C.; Westwell, A. D. An Efficient Synthetic Route to Biologically Relevant 2-Phenylbenzothiazoles Substituted on the Benzothiazole Ring. *Tetrahedron*, **67**, 7743-7747 (2011).
(46) Lee, B. C.; Kim, J. S.; Kim, B. S.; Son, J. Y.; Hong, S. K.; Park, H. S.; Moon, B. S.; Jung, J. H.; Jeong, J. M.; Kim, S. E. Aromatic Radiofluorination and Biological Evaluation of 2-Aryl-6-[$18^{F}$]Fluorobenzothiazoles as a Potential Positron Emission Tomography Imaging Probe for β-Amyloid Plaques. *Bioorg. Med. Chem.* **19**, 2980-2990 (2011).
(47) Loderer, D.; Nöth, H.; Pommerening, H.; Rattay, W.; Schick, H. Chemistry of Diborane(4) Derivatives: Mixed Tetraaminodiboranes(4) and Additions of Diborane(4) Derivatives to an Amino-imino-borane. *Chem. Ber.* **127**, 1605-1611 (1994).
(48) Baber, R. A.; Charmant, J. P. H.; Cook, A. J. R.; Farthing, N. E.; Haddow, M. F.; Norman, N. C.; Orpen, A. G.; Russell, C. A.; Slattery, J. M. Primary Amido Substituted Diborane(4) Compounds and Imidodiborate(4) Anions. *Dalton Trans.* 3137-3139 (2005).
(49) Lin, Y.; Li, D.; Zhang, J.; Tang, Z.; Liu, L.; Huang, T.; Li, C.; Chen, T. $I_2$ /$NaH_2PO_2$ -Mediated Deoxyamination of Cyclic Ethers for the Synthesis of *N*-Aryl-Substituted Azacycles. *New J. Chem.* **45**, 21011-21014 (2021).
(50) Zhang, Y.; Yang, X.; Yao, Q.; Ma, D. CuI/DMPAO-Catalyzed *N*-Arylation of Acyclic Secondary Amines. *Org. Lett.* **14,** 3056-3059 (2012).
(51) Murthy Bandaru, S. S.; Bhilare, S.; Chrysochos, N.; Gayakhe, V.; Trentin, I.; Schulzke, C.; Kapdi, A. R. Pd/PTABS: Catalyst for Room Temperature Amination of Heteroarenes. *Org. Lett.* **20**, 473-476 (2018).
(52) Roy, S.; Sarma, M. J.; Kashyap, B.; Phukan, P. A Quick Chan-Lam C-N and C-S Cross Coupling at Room Temperature in the Presence of Square Pyramidal [$Cu(DMAP)_4$ I]I as a Catalyst. *Chem. Commun.* **52**, 1170-1173 (2016).
(53) Dong, J.; Hu, J.; Liu, X.; Sun, S.; Bao, L.; Jia, M.; Xu, X. Ionic Reactivity of 2-Isocyanoaryl

Thioethers: Access to 2-Halo and 2-Aminobenzothia/Selenazoles. *J. Org. Chem.* **87**, 2845-2852 (2022).

(54) Villatoro, R. S.; Belfield, J. R.; Arman, H. D.; Hernandez, L. W.; Simmons, E. M.; Garlets, Z. J.; Wisniewski, S. R.; Coombs, J. R.; Frantz, D. E. General Method for Ni-Catalyzed C-N Cross-Couplings of (Hetero)Aryl Chlorides with Anilines and Aliphatic Amines under Homogeneous Conditions Using a Dual-Base Strategy. *Organometallics*, **42**, 3164-3172 (2023).

(55) Duparc, V. H.; Bano, G. L.; Schaper, F. Chan-Evans-Lam Couplings with Copper Iminoarylsulfonate Complexes: Scope and Mechanism. *ACS Catal.* **8***,* 7308-7325 (2018).

(56) Vantourout, J. C.; Miras, H. N.; Isidro-Llobet, A.; Sproules, S.; Watson, A. J. B. Spectroscopic Studies of the Chan-Lam Amination: A Mechanism-Inspired Solution to Boronic Ester Reactivity. *J. Am. Chem. Soc.* **139**, 4769-4779 (2017).

(57) Santoro, O.; Lazreg, F.; Minenkov, Y.; Cavallo, L.; Cazin, C. S. J. N-Heterocyclic Carbene Copper (I) Catalysed *N*-Methylation of Amines Using $CO_2$. *Dalton Trans.* **44**, 18138-18144 (2015).

(58) Muto, K.; Hatakeyama, T.; Yamaguchi, J.; Itami, K. C-H Arylation and Alkenylation of Imidazoles by Nickel Catalysis: Solvent-Accelerated Imidazole C-H Activation. *Chem. Sci.* **6**, 6792-6798 (2015).

(59) Song, G.; Nong, D.-Z.; Li, J.-S.; Li, G.; Zhang, W.; Cao, R.; Wang, C.; Xiao, J.; Xue, D. General Method for the Amination of Aryl Halides with Primary and Secondary Alkyl Amines via Nickel Photocatalysis. *J. Org. Chem.* **87**, 10285-10297 (2022).

(60) Yang, J.-F.; Liu, Y.-F.; Wei, L.-L.; Zhao, Y.-Q.; Shi, L. Visible Light-Mediated Photocatalyst-Free *N*-Demethylation of Aryl Tertiary Amines. *Tetrahedron Lett.* **124**, 154585 (2023).

(61) Yang, S.; Li, P.; Wang, Z.; Wang, L. Photoinduced Oxidative Formylation of *N,N*-Dimethylanilines with Molecular Oxygen without External Photocatalyst. *Org. Lett.* **19**, 3386-3389 (2017).

(62) Li, Z.; Li, C.-J. CuBr-Catalyzed Efficient Alkynylation of $Sp^3$ C-H Bonds Adjacent to a Nitrogen Atom. *J. Am. Chem. Soc.* **126**, 11810-11811 (2004).

(63) Han, W.; Ofial, A. R. Iron Catalyzed Oxidative Cyanation of Tertiary Amines. *Chem. Commun.* **33**, 5024-5026 (2009).

(64) Liu, D.; Liu, C.; Li, H.; Lei, A. Direct Functionalization of Tetrahydrofuran and 1,4-Dioxane: Nickel-Catalyzed Oxidative $C(Sp^3)$-H Arylation. *Angew. Chem. Int. Ed.* **52**, 4453-4456 (2013).

(65) Bergamaschi, E.; Weike, C.; Mayerhofer, V. J.; Funes-Ardoiz, I.; Teskey, C. J. Dual Photoredox/Cobaloxime Catalysis for Cross-Dehydrogenative α-Heteroarylation of Amines. *Org. Lett.* **23***,* 5378-5382 (2021).

(66) Bonifazi, A.; Battiti, F. O.; Sanchez, J.; Zaidi, S. A.; Bow, E.; Makarova, M.; Cao, J.; Shaik, A. B.; Sulima, A.; Rice, K. C.; Katritch, V.; Canals, M.; Lane, J. R.; Newman, A. H. Novel Dual-Target $\mu$-Opioid Receptor and Dopamine $D_3$ Receptor Ligands as Potential Nonaddictive Pharmacotherapeutics for Pain Management. *J. Med. Chem.* ***64***, 7778-7808 (2021).

(67) Jelali, H.; Mansour, L.; Deniau, E.; Sauthier, M.; Hamdi, N. An Efficient Synthesis of

Phthalimides and Their Biological Activities. *Polycycl. Aromat. Compd.* **42**, 1806-1813 (2022).

(68) Li, H.; Cai, G.-X.; Shi, Z.-J. LiCl-Promoted Pd(II)-Catalyzed Ortho Carbonylation of *N,N*-Dimethylbenzylamines. *Dalton Trans.* **39**, 10442 (2010).

(69) Lutovsky, G. A.; Gockel, S. N.; Bundesmann, M. W.; Bagley, S. W.; Yoon, T. P. Iron-Mediated Modular Decarboxylative Cross-Nucleophile Coupling. *Chem*, ***9***, 1610-1621 (2023).

(70) Gaussian 16, Revision A.03, M. J. Frisch, G. W. Trucks, H. B. Schlegel, G. E. Scuseria, M. A. Robb, J. R. Cheeseman, G. Scalmani, V. Barone, G. A. Petersson, H. Nakatsuji, X. Li, M. Caricato, A. V. Marenich, J. Bloino, B. G. Janesko, R. Gomperts, B. Mennucci, H. P. Hratchian, J. V. Ortiz, A. F. Izmaylov, J. L. Sonnenberg, D. Williams-Young, F. Ding, F. Lipparini, F. Egidi, J. Goings, B. Peng, A. Petrone, T. Henderson, D. Ranasinghe, V. G. Zakrzewski, J. Gao, N. Rega, G. Zheng, W. Liang, M. Hada, M. Ehara, K. Toyota, R. Fukuda, J. Hasegawa, M. Ishida, T. Nakajima, Y. Honda, O. Kitao, H. Nakai, T. Vreven, K. Throssell, J. A. Montgomery, Jr., J. E. Peralta, F. Ogliaro, M. J. Bearpark, J. J. Heyd, E. N. Brothers, K. N. Kudin, V. N. Staroverov, T. A. Keith,R. Kobayashi, J. Normand, K. Raghavachari, A. P. Rendell, J. C. Burant, S. S. Iyengar, J. Tomasi, M. Cossi, J. M. Millam, M. Klene, C. Adamo, R. Cammi, J. W. Ochterski, R. L. Martin, K. Morokuma, O. Farkas, J. B. Foresman, and D. J. Fox, Gaussian, Inc., Wallingford CT, 2016.

(71) a) Grimme, S.; Ehrlich, S.; Goerigk, L. Effect of the damping function in dispersion corrected density functional theory. *J. Comput. Chem.* **32**, 1456-1465 (2011).; b) Zhao, Y.; Truhlar, D. G. A new local density functional for main-group thermochemistry, transition metal bonding, thermochemical kinetics, and noncovalent interactions. *J. Chem. Phys.* **125**, 194101 (2006).

(72) M. Dolg, U. Wedig, H. Stoll, H. Preuss, Energy-adjusted ab initio pseudopotentials for the first row transition elements. *J. Chem. Phys.* **86**, 866-872 (1987).

(73) Marenich, A. V.; Cramer, C. J.; Truhlar, D. G. Universal Solvation Model Based on Solute Electron Density and on a Continuum Model of the Solvent Defined by the Bulk Dielectric Constant and Atomic Surface Tensions. *J. Phys. Chem. B,* **113**, 6378-6396 (2009).
