# Supplementary material for: Nickel catalyzed C-N coupling of haloarenes with B2N4 reagents
Source: Nat Commun. 2025 Apr 3;16:3202. doi: 10.1038/s41467-025-58438-6 (PMC11968942; doi:10.1038/s41467-025-58438-6)
Supplement: Supplementary file 2 — Description of Additional Supplementary Files [file 41467_2025_58438_MOESM2_ESM.pdf]

Description of Additional Supplementary Files

File Name: Supplementary Data 1

Description: Cartesian coordinates of the calculated structures.
